# Development and Validation of an Explainable Machine Learning Model to Assess the Prevalence Probability of Gastrointestinal Heat Retention Syndrome in Children: Cross-Sectional Study

**DOI:** 10.2196/94775

**Published:** 2026-07-02

**Authors:** Senlong Hou, Jiyu Jiang, Xue Li, Mingze Yang, Qilin Chen, Xueyan Ma, Xiaohong Gu

**Affiliations:** 1 School of Traditional Chinese Medicine Beijing University of Chinese Medicine Beijing, Beijing China; 2 Institute of Epidemic Diseases School of Traditional Chinese Medicine Beijing University of Chinese Medicine Beijing China

**Keywords:** energy metabolism, gastrointestinal heat retention syndrome, machine learning, explainability, prevalence probability assessment model

## Abstract

**Background:**

Health problems associated with energy metabolism imbalance have received increasing attention. Our team was the first to propose the concept of gastrointestinal heat retention syndrome (GHRS). Triggered by high-calorie diets and excessive energy intake, GHRS is strongly associated with recurrent respiratory infections and other pediatric conditions in children. Existing research on GHRS has primarily focused on risk factor analyses, and no interpretable exploratory self-assessment model for estimating prevalence probability is currently available for routine household use.

**Objective:**

This study aims to develop and validate an interpretable machine learning model to help caregivers conduct exploratory self-assessments of the probability that children meet the criteria for pediatric GHRS and to provide a practical assessment tool for household use.

**Methods:**

This study conducted a questionnaire survey of kindergarten children in Longgang District, Shenzhen, China. Samples with missing information on GHRS-related symptoms and signs were excluded. Independent correlates were identified using univariate logistic analysis, collinearity testing, Least Absolute Shrinkage and Selection Operator regression, and multivariable logistic regression. After handling missing data and preprocessing, the dataset was randomly divided into training and test sets in an 8:2 ratio. SMOTETomek (Synthetic Minority Oversampling Technique Tomek Links) was included as a conservative resampling step in the training pipeline, and optimal features were selected using a combination of Pearson correlation analysis and recursive feature elimination. In addition, a Random Forest (RF) sensitivity analysis without SMOTETomek was performed, and the minimum required sample size was estimated according to the events per variable (EPV) rule. Seven machine learning models were developed and evaluated in terms of discrimination, calibration, and clinical utility. The SHAP (Shapley Additive Explanations) method was used to interpret the optimal model, which was subsequently deployed online using the Streamlit framework.

**Results:**

A total of 120,198 questionnaires were collected, of which 108,447 were deemed valid and included in the analysis. The ratio of GHRS-positive to GHRS-negative cases was nearly balanced. The study identified 59 independent correlates of GHRS, including 10 protective factors and 49 risk factors. After data cleaning, a complete-case dataset of 49,798 participants was obtained, exceeding the minimum sample size requirement of 3747 cases based on the EPV rule. During internal validation, the RF model demonstrated acceptable discriminatory performance, stable calibration, and high net benefit in decision curve analysis, and was therefore selected as the primary analytical model. SHAP analysis identified 5 key predictive features. The resulting online tool collects information through 75 single-choice questions and automatically provides an estimated probability of GHRS together with lifestyle recommendations.

**Conclusions:**

Using cross-sectional data, this study developed and validated an interpretable model that enables caregivers to perform exploratory self-assessments of the probability that children meet the study-specific GHRS scale criteria. As a household self-screening tool, the model helps caregivers estimate this probability based on readily obtainable information and model-generated risk estimates.

## Introduction

Human energy metabolism follows an energy balance equation, where energy intake equals total energy expenditure [1]. Energy intake occurs through dietary consumption, while total energy expenditure represents the sum of the organism’s daily basal metabolism (or resting energy expenditure), the thermic effect of food, and heat production from physical activity [2]. Basal metabolism refers to the energy expended at rest, accounting for approximately 60% of total daily energy expenditure. The thermic effect of food denotes the postprandial increase in metabolic rate, encompassing energy consumed for food processing and storage, as well as the metabolic effects of nutrient intake, constituting about 10% of total energy expenditure. Heat production from physical activity comprises both exercise and nonexercise activity thermogenesis. Conscious activities such as physical exercise constitute exercise thermogenesis, accounting for 0%-10% of total energy expenditure. Unconscious activities, including fidgeting and posture maintenance, represent nonexercise activity thermogenesis, contributing approximately 20% of total energy expenditure [3,4]. Since the Industrial Revolution began approximately 250 years ago, significant changes have occurred in human dietary patterns and lifestyles [5]. Decreasing food costs, advancements in processing technology, and increased food availability have progressively elevated energy intake in human diets. Concurrently, sedentary lifestyles characterized by physical inactivity and sun avoidance have gradually reduced energy expenditure. This energy imbalance ultimately results in a widespread energy surplus across populations, leading to various health problems [6,7]. Currently, most scholars posit that when energy intake exceeds total energy expenditure, the surplus is converted into fat for storage, consequently leading to obesity [8,9]. Our research team observed clinically that when energy intake surpasses the combined demands of basal metabolism, the thermic effect of food, heat production from physical activity, and energy expenditure for fat conversion, excess energy may be consumed through mechanisms such as enhanced metabolism or triggered inflammatory responses, manifesting as specific digestive tract symptoms. Animal studies have demonstrated that high-calorie diets can aggravate lipopolysaccharide-induced pneumonia through acetate-mediated macrophage polarization via the histone deacetylase 9/histone deacetylase 10-hypoxia-inducible factor-1α glycolysis axis. They also worsen 2,4-dinitrochlorobenzene–induced eczema through T helper 1/2 and T helper 17/regulatory T-cell imbalances, increased inflammatory factor expression, heightened oxidative stress, and alterations in the gut microbiota. Additionally, such diets elevate murine serum levels of the proinflammatory cytokines tumor necrosis factor-α and interleukin (IL)-6 while reducing anti-inflammatory IL-10. They upregulate Toll-like receptor 4, tumor necrosis factor-α, and IL-6 transcripts in the small intestine, alter intestinal permeability, and modify rat gut microbiota structure, inducing significant inflammatory changes in digestive tissues, thereby further substantiating this phenomenon [10-13].

Our team has provisionally termed this specific digestive tract syndrome—induced by enhanced metabolism and inflammatory responses resulting from high-calorie diets—as gastrointestinal heat retention syndrome (GHRS). GHRS primarily presents with gastrointestinal symptoms, including dry stool, straining during defecation, and strong fecal odor, and may be accompanied by dry mouth, increased eye discharge, epistaxis, oral ulcers, and elevated skin temperature of the palms and soles. Research demonstrates that GHRS is closely associated with childhood conditions such as recurrent respiratory infections, eczema, allergic purpura, and recurrent functional abdominal pain. Interventions targeting GHRS may therefore positively affect the prevention of such pediatric diseases [14].

The diagnosis of GHRS primarily relies on the diagnostic model and testing scale developed by our research team. The model is based on the Extreme Gradient Boosting (XGBoost) algorithm and achieved an internal validation accuracy of 93.03% [15]. The etiology of GHRS is closely associated with sociodemographic factors and lifestyle habits. Animal studies indicate that high-protein, high-calorie diets can induce GHRS. The established rat model exhibits characteristics including a preference for cooler areas, reduced bowel sounds, increased fecal characteristic scores, and elevated anal temperature. These changes are accompanied by elevated levels of the inflammatory factors IL-6, Iba-1 (ionized calcium-binding adapter molecule 1), and nuclear factor-kappa B p65, along with reduced levels of the blood-brain barrier structural proteins Claudin-5 and Occludin [16]. Our cross-sectional study in Beijing revealed variations in GHRS prevalence among children with different sociodemographic characteristics, including age, gender, household income, and parenting style. Children aged 3-5 years, girls, long-term urban residents, those with grandparents as primary caregivers, monthly household incomes of 10,000-20,000 CNY (1 CNY=US $0.15, as of June 19, 2026), antibiotic use before age 1 year, preterm birth, nonbreastfeeding, dinner after 7:00 PM, consumption of roasted, fried, or pan-fried foods 2 or more times per week, and frequent snack or dessert intake were identified as having a higher risk of GHRS. Conversely, daily indoor exercise exceeding 30 minutes and higher consumption of vegetables, fish, and soy products were identified as protective factors against GHRS [17]. Currently, research on GHRS is limited to the exploration of associated factors, and no prevalence prediction model has been developed; therefore, its practical clinical utility remains limited.

In recent years, machine learning (ML) techniques have been widely applied in disease screening. Zhu et al [18] used 8 demographic and clinical characteristics, including age, education level, hypertension, and a history of diabetes, to construct a prediction model for metabolic dysfunction–associated steatotic liver disease using 10 ML models, such as logistic regression (LR) and multilayer perceptron [18]. To enhance the credibility of model behavior and outcomes, ML models require explainability analyses. For instance, when Li et al [19] used the XGBoost model to predict prediabetes risk, they used the SHAP (Shapley Additive Explanations) method for explainability analysis. Their findings indicated that age, hypertension, and white blood cell count may be risk factors for prediabetes. When dealing with a large number of features, feature selection becomes necessary. Fitriyani et al [20] constructed an integrated prediction framework for cardiovascular disease by combining information gain feature selection, local outlier detection, and Bagging-Histogram Gradient Boosting. Through comparative testing against multiple traditional ML models, they demonstrated that feature selection combined with ensemble learning effectively enhances model discriminatory power and generalization capability [20].

This cross-sectional questionnaire survey investigated the positive rate of GHRS and its potential sociodemographic and lifestyle correlates among preschool children in Longgang District, Shenzhen. Using the collected data, we developed and validated an interpretable ML model to enable caregivers to estimate the probability that a child meets the study-specific GHRS scale criteria and subsequently developed a convenient assessment tool.

## Methods

### Data Collection

Data were collected through questionnaires from children attending 442 kindergartens in Longgang District, Shenzhen, between May and July 2021. The collected information encompassed basic demographic data, clinical symptoms and signs of GHRS, other symptoms and signs, and dietary behaviors. The inclusion criteria were (1) children enrolled in kindergartens in Longgang District during the survey period, and (2) guardians who were willing and able to complete the survey. The exclusion criterion was missing data on GHRS-related symptoms and signs, rendering confirmation of a GHRS diagnosis impossible.

### Clinical Features

The variable data evaluated in this study were obtained through an online questionnaire survey completed by guardians based on the children’s actual circumstances. Children’s basic information encompassed 39 items, including kindergarten name, grade level, class, relationship to the informant, gender, date of birth, feeding method during the first 6 months of life, timing of animal protein complementary food introduction, and other relevant information. Clinical symptoms and accompanying signs of GHRS were 19 symptoms and signs observed within the preceding 2 weeks, such as increased palm and sole skin temperature, heat aversion, oral malodor, warm exhaled air from the mouth or nose, and thirst accompanied by a preference for cold drinks. Other symptoms and signs were derived from manifestations occurring within the preceding year and included listlessness, anorexia, sallow complexion, flaccid muscles, a preference for quietness, weak and feeble speech, reticence, excessive sweating, cold hands and feet, aversion to cold, dislike of raw and cold foods, and 51 additional symptoms or signs.

### Research Results Evaluation

Symptoms and signs related to GHRS diagnosis were all included in the questionnaire. The questionnaire was completed by the children’s legal guardians, who possessed basic literacy skills. Following the questionnaire instructions, guardians provided responses based on their observations and judgments regarding the children’s symptoms and signs. This study used a guardian-administered questionnaire survey and did not implement blinding of investigators or outcome assessors. GHRS diagnosis was conducted using the “Self-Rating Diagnostic Scale for Gastrointestinal Heat Retention Syndrome in Children” developed by our team in prior research, which has demonstrated good reliability and validity [21,22]. The scale comprises 10 items with different weighting coefficients. Individuals with a cumulative score of 133 points or more were classified as GHRS positive, whereas those with scores below this threshold were classified as non-GHRS, as detailed in Table 1.

**Table 1:** Items and weight coefficients of the “Self-Rating Diagnostic Scale for Gastrointestinal Heat Retention Syndrome in Children” questionnaire (GHRS^a^ diagnosis requires ≥133 points).

Item	Weight coefficient
Dry and hard stools	100
Abnormal appetite	55
Dark-colored urine	47
Restless sleep	33
Decreased defecation frequency	32
Halitosis	28
Heat sensation in palms and soles	24
Night sweating	21
Irritability	16
Foul-smelling stools	15

^a^GHRS: gastrointestinal heat retention syndrome.

### Data Preprocessing

Box plots were used to detect outliers in continuous variables. Outliers were defined as values above the upper quartile plus 1.5 times the IQR or below the lower quartile minus 1.5 times the IQR (Table S1 in Multimedia Appendix 1). Each outlier was replaced with the nearest boundary value to align with the main data distribution (Figure S1 in Multimedia Appendix 1). To maximize data validity, samples with missing values were excluded before model training. Categorical variables were predefined before model construction, and their data were processed using one-hot encoding. All sociodemographic subgroups were subjected to the same preprocessing procedures for outlier screening, missing value handling, and feature encoding, without differential treatment. Independent correlation analyses were performed to examine statistical associations between variables and GHRS and to assess the feasibility of model development. These analyses did not contribute to feature selection for the final model.

### Variable Selection

In this study, GHRS diagnosis required the use of assessment scales. To prevent the trained ML models from learning the scale scoring rules directly or indirectly, we initially excluded variables related to GHRS clinical symptoms and accompanying signs (including scale items), retaining 467 other variables (Table S2 in Multimedia Appendix 1). Subsequently, univariate correlation screening and recursive feature elimination (RFE) were used for feature selection to enhance model performance and stability. Univariate correlation screening utilized the Pearson correlation coefficient to calculate the absolute correlation between each feature and the GHRS diagnostic outcome. Features with an absolute correlation coefficient of 0.1 or more were retained, whereas those exhibiting weaker correlations with the target variable were excluded to minimize interference from redundant variables. RFE is a widely used feature selection method in ML that eliminates unimportant features and ultimately identifies the optimal feature combination for achieving peak model performance. During the RFE process, 10-fold stratified cross-validation was applied, and features contributing minimally to model performance were recursively eliminated to derive the optimal feature subset for each model.

### Sample Size Determination

According to the events per variable (EPV) rule, the minimum required sample size for the training set was calculated using the following formula:

Minimum required sample size for the training set = number of factors included × EPV/incidence of positive events in the training set

where the number of included factors refers to the number of features in the optimal feature subset obtained after univariate correlation screening and RFE. The positive event incidence rate in the training set represents the probability of a positive GHRS diagnosis. The EPV value was set at 10. According to existing research findings, the prevalence of GHRS among children is approximately 51.88% [17]. Therefore, the minimum required sample size for the training set should be at least 19.27 times the number of features in the optimal feature subset.

### Model Development and Validation

Using stratified random sampling, the preprocessed dataset was divided into training and test sets at an 8:2 ratio. Final feature selection was conducted exclusively within the training set and cross-validation process to avoid information leakage from the test set. The test set was used solely for final model performance evaluation and did not participate in feature screening, resampling, hyperparameter tuning, or model selection. This study included samples only from multiple kindergartens in Longgang District, Shenzhen, and did not contain multiregional subsets; therefore, cross-subset heterogeneity was not assessed. After retaining complete cases, and considering that complete-case screening may alter the distribution of the training set, SMOTETomek (Synthetic Minority Oversampling Technique Tomek Links) was prespecified as a conservative resampling step in the training pipeline. Given the nearly balanced ratio of GHRS-positive to GHRS-negative cases, this step was not considered necessary to correct severe class imbalance but was retained as part of the primary analysis pipeline. SMOTETomek, feature standardization, and model training were integrated into an ImbPipeline (Imbalanced Learning Pipeline) and fitted exclusively on the training set and within cross-validation training folds. Validation folds and the test set were used solely for performance evaluation and were not involved in resampling, feature screening, hyperparameter tuning, or model selection, thereby minimizing the risk of information leakage. Seven ML models—Decision Tree, K-Nearest Neighbors, Linear Support Vector Machine (LinearSVC), LR, naive Bayes (NB), Random Forest (RF), and XGBoost—were used to estimate the probability of GHRS positivity. To optimize model performance, hyperparameter tuning was performed using grid search based on 3 rounds of 3-fold cross-validation to determine the final hyperparameters for each model using the optimal feature subset. Finally, because the outputs of the Decision Tree and LinearSVC models do not represent reliable probability estimates, Platt scaling (sigmoid) was applied for probability calibration to all models. Specifically, a calibration function was fitted using 3-fold cross-validation to convert model outputs into calibrated probability values ranging from 0 to 1.

After model construction, internal validation was performed. We conducted 10-fold stratified cross-validation on the training set. In each cross-validation iteration, feature standardization, SMOTETomek resampling, model training, and probability calibration were performed exclusively within the training folds, whereas the validation folds were used solely for performance evaluation. Performance metrics, including the area under the receiver operating characteristic curve (AUC), sensitivity, specificity, and Brier score, were calculated for each fold. The optimal threshold for each fold was determined using the Youden Index. Results were then aggregated across all folds to estimate the average performance on the training set and to derive the global optimal threshold. Using the optimal feature subset, optimal hyperparameters, and global optimal threshold obtained from the training set, the final model was trained. The model was subsequently validated on the test set, and performance metrics were calculated.

### Model Performance Comparison

Model performance was compared across 3 dimensions: discriminatory power, calibration, and clinical utility. To evaluate discriminatory power, the AUC was primarily used to assess the model’s ability to distinguish between GHRS-positive and GHRS-negative samples. The AUC ranges from 0 to 1, with values closer to 1 indicating better discriminatory performance. Sensitivity and specificity were also calculated at both the fixed threshold (0.5) and the optimal threshold. Sensitivity reflects the model’s ability to identify positive samples, whereas specificity reflects its ability to identify negative samples. For calibration assessment, the Brier score was used to evaluate the accuracy of the model’s probability estimates. The Brier score ranges from 0 to 1, with values closer to 0 indicating better agreement between the predicted probabilities and the observed outcomes. Additionally, a calibration curve was plotted with the model-predicted probability on the x-axis and the observed positive rate on the y-axis to visually assess the model’s calibration performance. To further quantify calibration, the calibration slope and calibration intercept were calculated. The calibration slope typically approaches 1, with values closer to 1 indicating better agreement between the trend of the predicted probabilities and the observed positive rate. The calibration intercept typically approaches 0, with values closer to 0 indicating lower overall prediction bias. For clinical utility evaluation, decision curve analysis (DCA) was used to calculate the net benefit across different threshold probabilities. A higher net benefit indicates greater practical value of the model at a given clinical decision threshold. Decision curves were then plotted to visually compare the net benefits of each model with those of the “intervention for all” and “intervention for none” strategies. Finally, based on the performance of the above evaluation metrics in both the training and test sets, the optimal model was selected according to its overall practical utility.

### Model Explanation

This study used the SHAP method to interpret the optimal ML model. SHAP is a game theory–based technique that ranks the importance of input features and explains model outputs, thereby addressing the limited interpretability of ML models. By calculating each feature’s contribution to the model output, SHAP provides both global and local interpretability, enhancing model transparency and explainability. In addition, a predicted probability distribution plot for the optimal model was generated to illustrate the agreement between model-predicted probabilities and actual outcomes, thereby supporting the reliability of the model evaluation results.

### Network Calculator

To facilitate exploratory self-assessment at home, we integrated the optimized final model into a web application developed using the Streamlit framework. After users complete the online questionnaire and enter values for all predictive features, the application calculates and returns the probability that the child meets the study-defined GHRS scale criteria.

### Statistics

The aforementioned ML models were developed using the scikit-learn and Keras libraries in Python (version 3.11; Python Foundation). Continuous variables with normal distributions are presented as mean (SD) and were compared using the unpaired (2-tailed) *t* test. Categorical variables were represented using one-hot encoding. Independent correlates in the overall cohort were identified through univariate LR analysis, collinearity testing, stepwise Wald regression analysis, Least Absolute Shrinkage and Selection Operator (LASSO) regression analysis, and multivariable LR analysis.

### Ethical Considerations

This study protocol adhered to the principles of the Declaration of Helsinki and received approval from the Ethics Review Committee of Beijing University of Chinese Medicine, Shenzhen Hospital (Longgang; approval number SZLDH2021LSYM-03). As a cross-sectional study, this research was not registered in a clinical trial registry before data collection. This study was conducted with informed consent obtained from kindergarten administrators and the guardians of participating children. Data collection complied with the Ethical Review Measures for Life Science and Medical Research Involving Humans and the Personal Information Protection Law of the People’s Republic of China. The dataset was rigorously anonymized and deidentified to ensure that no participants could be identified, and no participant identities could be discerned from any images or multimedia appendices associated with this article. Participants received standardized compensation determined by the research team. Detailed compensation terms are not disclosed beyond the participants and members of the research team.

This study was reported in accordance with the TRIPOD+AI (Transparent Reporting of a Multivariable Prediction Model for Individual Prognosis or Diagnosis + Artificial Intelligence) guidelines. The complete TRIPOD+AI abstract and full reporting checklists are provided in Multimedia Appendices 2 and 3, respectively.

## Results

### Baseline Clinical Characteristics

**Figure 1:**
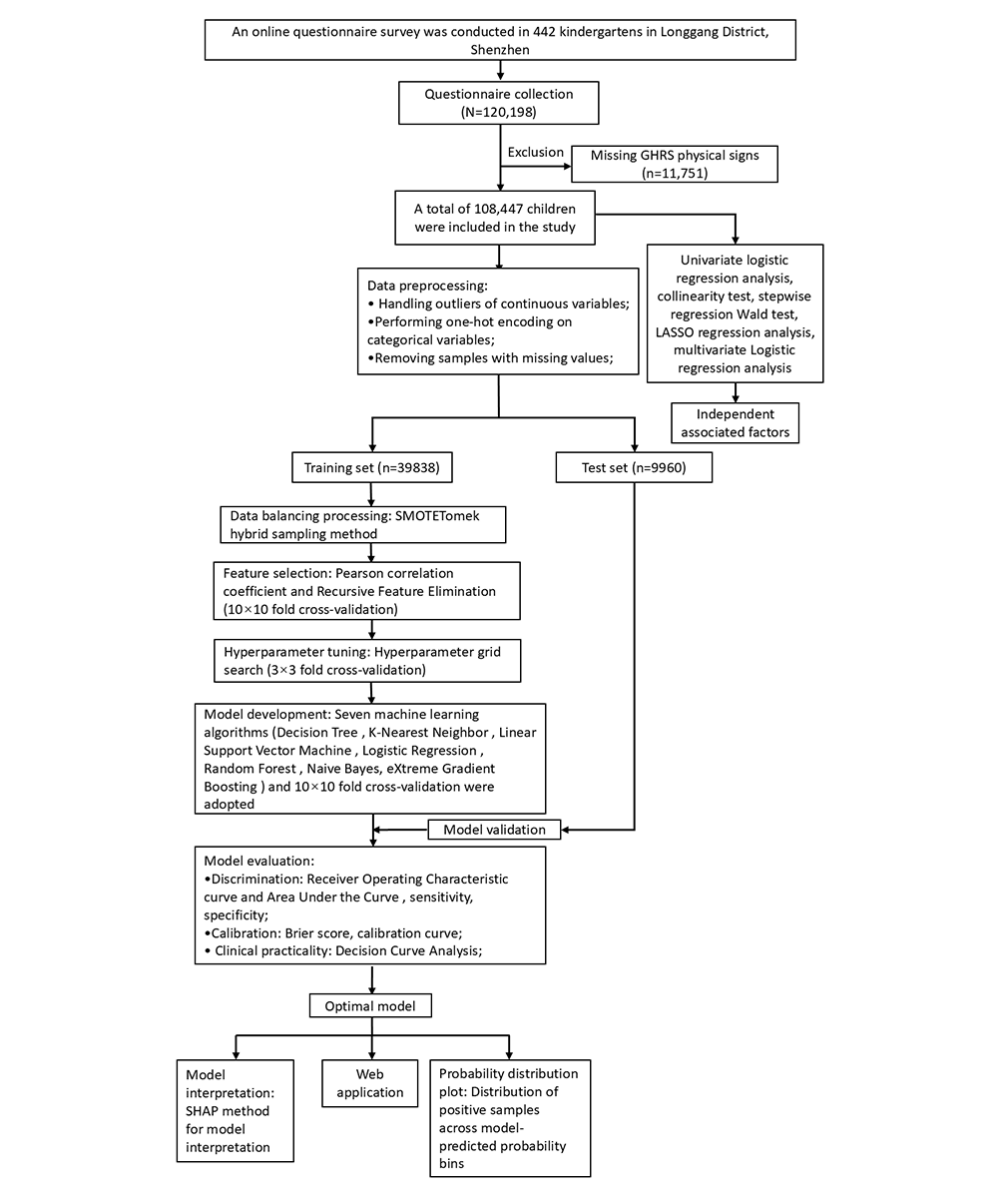
Research Process Design Diagram.

Following questionnaire distribution, 120,198 questionnaires were returned. Of these, 11,751 were excluded because of missing data on GHRS symptoms and signs, leaving 108,447 questionnaires for analysis. The demographic characteristics of the children are presented in Table 2. As shown in Table 2, significant differences (*P*<.001) were observed between the GHRS-positive and GHRS-negative groups with respect to gender, grade level, age, height, and body weight, indicating that these demographic variables may contribute to estimating the probability that a child meets the GHRS scale criteria. Details of the study design are presented in Figure 1.

**Table 2:** Comparison of demographic characteristics between GHRS^a^-positive and non-GHRS groups.

Characteristic (feature)			Overall (N=108,447)		GHRS0 (n=56,815)		GHRS1 (n=51,632)		*P* value			
**GHRS stage, n (%)**									.99			
	GHRS0	56,815 (52.39)		N/A^b^		N/A						
	GHRS1	51,632 (47.61)		N/A		N/A						
**Age (years), n (%)**												
	3	8828 (8.14)		4691 (8.26)		4137 (8.01)		<.001				
	4	38,258 (35.28)		19,404 (34.15)		18,854 (36.52)		<.001				
	5	33,804 (31.17)		15,755 (27.73)		18,049 (34.96)		<.001				
	6	26,774 (24.69)		11,483 (20.21)		15,291 (29.62)		<.001				
	7	783 (0.72)		299 (0.53)		484 (0.94)		<.001				
**Gender, n (%)**									<.001			
	Female	50,823 (46.86)		25,399 (44.70)		25,424 (49.24)						
	Male	57,624 (53.14)		31,416 (55.30)		26,208 (50.76)						
**Ethnic group, n (%)**									.29			
	Han Chinese	103,370 (95.32)		54,118 (95.25)		49,252 (95.39)						
	Other ethnic groups	5077 (4.68)		2697 (4.75)		2380 (4.61)						
**Grade, n (%)**												
	Junior class	33,691 (31.07)		16,126 (28.38)		17,565 (34.02)		<.001				
	Middle class	35,548 (32.78)		18,329 (32.26)		17,219 (33.35)		<.001				
	Senior class	38,949 (35.92)		22,223 (39.11)		16,726 (32.39)		<.001				
	Others	259 (0.24)		137 (0.24)		122 (0.24)		<.001				
**Only child, n (%)**										<.001		
	Yes	79,155 (72.99)		43,162 (75.97)		35,993 (69.71)						
	No	29,292 (27.01)		13,653 (24.03)		15,639 (30.29)						
Height (cm), mean (SD)			111.60 (8.02)		112.26 (8.08)		110.87 (7.89)		<.001			
Weight (kg), mean (SD)			19.24 (3.57)		19.56 (3.6)		18.9 (3.51)		<.001			
BMI (kg/m^2^), mean (SD)			15.37 (1.98)		15.45 (2.01)		15.28 (1.94)		<.001			

^a^GHRS: gastrointestinal heat retention syndrome.

^b^N/A: not applicable.

### Independent Correlative Factors

To determine the association between GHRS prevalence and questionnaire-collected variables while preliminarily assessing the feasibility of constructing a GHRS prevalence prediction model, we investigated independent correlates of GHRS in children using a dataset comprising 108,447 samples. After creating dummy variables and applying one-hot encoding to categorical variables, 469 variables were obtained. Variables such as “unclear,” which were designed to improve questionnaire completeness but lacked analytical significance, were excluded, leaving 467 variables. Univariate LR analysis identified 433 potential correlates associated with GHRS prevalence (*P*<.01; see Table S2 in Multimedia Appendix 1 for the complete list of *P* values). After collinearity testing, 335 potential correlates were retained (variance inflation factor [VIF]<10). Following stepwise regression with Wald tests, 170 potential correlates were identified (*P*<.001), and LASSO regression analysis identified 113 potential correlates (coefficient≠0). Finally, multivariable LR analysis identified 59 independent correlates associated with GHRS prevalence, including 10 protective factors and 49 risk factors (Table S2 in Multimedia Appendix 1 and Tables 3 and 4).

**Table 3:** Protective factors for GHRS^a^.

Features			Non-GHRS (n=56,815), n		GHRS (n=51,632), n		Univariate logistic regression, odds ratio (95% CI); *P* value^b^		Collinearity statistics VIF^c,d^		Stepwise regression Wald test (*P* value)^e^		LASSO^f^ regression analysis^g^		Multivariate logistic regression, odds ratio (95% CI); *P* value^h^		
**Sex**																	
	Female	25,399		25,424		Reference		Reference		Reference		Reference		Reference			
	Male	31,416		26,208		0.833 (0.814-0.854); <.001		1.011477787		<.001		–0.05		0.776 (0.750-0.803); <.001			
**Only child**																	
	No	13,653		15,639		Reference		Reference		Reference		Reference		Reference			
	Yes	43,162		35,993		0.728 (0.709-0.748); <.001		1.395246244		<.001		–0.023		0.896 (0.863-0.931); <.001			
**Which child of the mother**																	
	Third	3167		1723		0.580 (0.547-0.616); <.001		5.626435311		<.001		–0.015		0.857 (0.787-0.933); <.001			
**Regular lunch breaks**																	
	No	14,204		17,569		Reference		Reference		Reference		Reference		Reference			
	Yes	42,611		34,063		0.646 (0.630-0.664); <.001		1.039852719		<.001		–0.034		0.848 (0.815-0.882); <.001			
**The highest level of education of the child’s mother**																	
	Junior high school	10,893		7442		0.710 (0.687-0.733); <.001		2.293610452		<.001		–0.043		0.816 (0.776-0.859); <.001			
	High school/technical secondary school	12,812		10,331		0.859 (0.834-0.885); <.001		1.917866133		<.001		–0.014		0.921 (0.883-0.962); <.001			
**Soft muscles**																	
	No	44,217		29,051		0.367 (0.357-0.376); <.001		1.885		<.001		–0.042		0.890 (0.834-0.951); <.001			
**Little activity makes sweating easily**																	
	No	16,259		6777		0.377 (0.365-0.389); <.001		1.561942249		<.001		–0.046		0.803 (0.766-0.841); <.001			
**Having a cold or eating (drinking) cold food makes it easy to have diarrhea**																	
	Rarely	11,069		14,780		1.658 (1.611-1.705); *<.*001		1.574801614		<.001		–0.009		0.904 (0.866-0.943); <.001			
**Parental attitude about children’s picky eating**																	
	Do not worry	29,327		16,436		0.438 (0.427-0.449); <.001		1.534487075		<.001		–0.051		0.824 (0.792-0.857); <.001			

^a^GHRS: gastrointestinal heat retention syndrome.

^b^Variables with *P*<.01 retained in the final model.

^c^Variables with VIF<10 retained.

^d^VIF: variance inflation factor.

^e^Variables with *P*<.001 retained.

^f^LASSO: Least Absolute Shrinkage and Selection Operator.

^g^α=.001; variables with nonzero values are retained.

^h^Variables with *P*<.001 retained in the final model.

**Table 4:** Risk factors for GHRS^a^.

Features			Non-GHRS (n=56,815), n		GHRS (n=51,632), n		Univariate logistic regression, odds ratio (95% CI); *P* value^b^		Collinearity statistics VIF^c,d^		Stepwise regression Wald test (*P* value)^e^		LASSO^f^ regression analysis^g^		Multivariate logistic regression, odds ratio (95% CI); *P* value^h^		
**Age (years)**																	
	3	4137		4691		1.272 (1.218-1.329); <.001		1.940612827		<.001		0.038		1.264 (1.183-1.351); <.001			
	4	18,854		19,404		1.212 (1.182-1.243); <.001		2.727345876		<.001		0.022		1.149 (1.103-1.198); <.001			
**Fed formula milk powder within 6 months after birth**																	
	No	41,042		34,535		Reference		Reference		Reference		Reference		Reference			
	Yes	15,773		17,097		1.288 (1.255-1.322); <.001		8.048		<.001		0.029		1.155 (1.114-1.198); <.001			
**The earliest age of using antibiotics**																	
	1-2 years old	5389		6897		1.562 (1.503-1.623); <.001		1.117598504		<.001		0.025		1.135 (1.083-1.191); <.001			
**History of allergies**																	
	No	42,690		32,995		Reference		Reference		Reference		Reference		Reference			
	Yes	14,125		18,637		1.707 (1.663-1.752); <.001		5.320354458		<.001		0.032		1.109 (1.058-1.164); <.001			
**History of functional dyspepsia**																	
	No	56,357		50,224		Reference		Reference		Reference		Reference		Reference			
	Yes	458		1408		3.450 (3.102-3.836); <.001		4.367928488		<.001		0.022		1.458 (1.250-1.701); <.001			
**History of functional constipation**																	
	No	56,676		50,694		Reference		Reference		Reference		Reference		Reference			
	Yes	139		938		7.545 (6.311-9.019); <.001		2.635422558		<.001		0.1		3.909 (3.040-5.026); <.001			
**Activity habits**																	
	Less physical activity	1432		2839		2.237 (2.097-2.387); <.001		1.10115061		<.001		0.046		1.418 (1.288-1.561); <.001			
	Moderate physical activity	27,703		27,676		1.204 (1.175-1.233); <.001		1.151989396		<.001		0.021		1.144 (1.104-1.185); <.001			
**Food preference**																	
	Particularly fond of certain foods	11,662		14,687		1.539 (1.497-1.583); <.001		1.090151058		<.001		0.025		1.144 (1.096-1.193); <.001			
	Particularly dislikes certain foods	5080		8207		1.925 (1.854-1.998); <.001		1.090358017		<.001		0.035		1.182 (1.116-1.251); <.001			
**Has your child ever undergone or is currently receiving vision correction treatment?**																	
	No	52,352		46,252		Reference		Reference		Reference		Reference		Reference			
	Yes	2089		2062		1.090 (1.024-1.159); .007		4.196512643		<.001		0.007		1.017 (1.007-1.027); <.001			
**Self-control ability**																	
	Poor self-control, throws tantrums when disciplined by parents	2023		4348		2.471 (2.341-2.609); <.001		1.305673304		<.001		0.08		1.540 (1.411-1.681); <.001			
	Poor self-control, accepts and corrects behavior when disciplined by parents	38,363		37,685		1.311 (1.274-1.350); <.001		1.304251357		<.001		0.031		1.181 (1.129-1.235); <.001			
**Whether to start subject education**																	
	No	40,866		35,671		Reference		Reference		Reference		Reference		Reference			
	Yes	13,382		14,107		1.208 (1.175-1.241); <.001		1.118251948		<.001		0.015		1.070 (1.030-1.113); <.001			
**Easy to lose spirit**																	
	Rarely	13,825		19,667		1.913 (1.864-1.964); <.001		1.298836233		<.001		0.03		1.142 (1.097-1.189); <.001			
	Occasionally	1936		5179		3.160 (2.995-3.335); <.001		1.288093984		<.001		0.049		1.289 (1.189-1.397); <.001			
**Yellow complexion**																	
	Rarely	9126		12,851		1.732 (1.680-1.784); <.001		1.343615945		<.001		0.025		1.183 (1.129-1.240); <.001			
	Occasionally	3359		7634		2.761 (2.646-2.881); <.001		1.430157381		<.001		0.038		1.291 (1.206-1.381); <.001			
	Often	812		2987		4.235 (3.915-4.581); <.001		1.296788958		<.001		0.059		1.616 (1.439-1.814); <.001			
	Always	287		1274		4.983 (4.381-5.667); <.001		1.267201053		<.001		0.013		1.359 (1.133-1.630); <.001			
**Little activity makes sweating easily**																	
	Often	9275		12,911		1.709 (1.659-1.761); <.001		1.489884719		<.001		0.028		1.119 (1.070-1.170); <.001			
	Always	2861		5557		2.274 (2.171-2.383); <.001		1.262973292		<.001		0.067		1.403 (1.309-1.505); <.001			
**Skin and lips dry easily**																	
	Rarely	9964		15,866		2.086 (2.027-2.146); <.001		1.514803499		<.001		0.022		1.109 (1.060-1.161); <.001			
	Occasionally	2456		5732		2.764 (2.632-2.902); <.001		1.356677507		<.001		0.026		1.137 (1.056-1.224, *P*<.001)			
**Always excited**																	
	Rarely	14,798		14,841		1.145 (1.115-1.176); <.001		1.719067637		<.001		0.003		1.087 (1.038-1.138); <.001			
	Occasionally	12,004		16,779		1.797 (1.749-1.847); <.001		2.103025086		<.001		0.019		1.127 (1.073-1.185); <.001			
	Often	3229		5997		2.181 (2.086-2.280); <.001		1.740525547		<.001		0.035		1.259 (1.167-1.357); <.001			
	Always	536		1162		2.417 (2.181-2.680); <.001		1.396219081		<.001		0.019		1.464 (1.259-1.702); <.001			
**Likes to eat sweet or greasy things**																	
	Often	2312		5439		2.776 (2.640-2.919); <.001		1.355237648		<.001		0.044		1.228 (1.138-1.325); <.001			
**Snoring at night**																	
	Rarely	11,682		15,484		1.655 (1.610-1.701); <.001		1.248193959		<.001		0.021		1.098 (1.053-1.145); <.001			
	Occasionally	3046		5743		2.209 (2.111-2.312); <.001		1.228896402		<.001		0.032		1.141 (1.066-1.222); <.001			
**Thick and greasy tongue coating**																	
	Rarely	12,445		18,884		2.056 (2.002-2.112); <.001		1.47076567		<.001		0.063		1.316 (1.261-1.374); <.001			
	Occasionally	2576		7138		3.378 (3.224-3.540); <.001		1.46854109		<.001		0.1		1.578 (1.467-1.696); <.001			
	Often	276		1212		4.924 (4.318-5.615); <.001		1.133033849		<.001		0.074		2.059 (1.714-2.474); <.001			
**Always red eyes or easy to get gum**																	
	Rarely	11,347		17,349		2.028 (1.973-2.084); <.001		1.457274308		<.001		0.03		1.153 (1.103-1.215); <.001			
	Occasionally	2366		5998		3.025 (2.880-3.177); <.001		1.390754326		<.001		0.037		1.227 (1.138-1.324); <.001			
	Often	245		1065		4.863 (4.230-5.590); <.001		1.122434296		<.001		0.001		1.572 (1.293-1.912); <.001			
**Sweat is sticky**																	
	Rarely	11,610		16,932		1.900 (1.848-1.953); <.001		1.341798555		<.001		0.023		1.083 (1.037-1.131); <.001			
	Occasionally	2425		5962		2.928 (2.788-3.074); <.001		1.29236379		<.001		0.041		1.245 (1.155-1.342); <.001			
**Easy to lack self-confidence**																	
	Often	519		1825		3.975 (3.603-4.385); <.001		1.6577046		<.001		0.02		1.332 (1.144-1.550); <.001			
**Always crying when in trouble**																	
	Often	1639		4227		3.002 (2.832-3.182); <.001		1.506670936		<.001		0.03		1.176 (1.076-1.285); <.001			
**Easy to have nightmares or wake up from dreams**																	
	Rarely	11,733		17,410		1.955 (1.902-2.009); <.001		1.283090699		<.001		0.03		1.141 (1.094-1.190); <.001			
	Occasionally	2218		4874		2.566 (2.437-2.702); <.001		1.181515355		<.001		0.022		1.168 (1.082-1.261); <.001			
**Eat less than your peers**																	
	Often	3943		8772		2.744 (2.638-2.855); <.001		1.49849829		<.001		0.055		1.316 (1.236-1.400); <.001			
	Always	909		3157		4.005 (3.717-4.316); <.001		1.868829617		<.001		0.061		1.472 (1.301-1.665); <.001			
**Distraction from eating (watching TV, playing video games, etc)**																	
	Often	7512		11,906		1.967 (1.906-2.030); <.001		1.40294084		<.001		0.017		1.090 (1.038-1.145); <.001			
**Force or punish children to eat more**																	
	Occasionally	13,876		19,772		1.920 (1.871-1.971); <.001		1.362750409		<.001		0.025		1.103 (1.059-1.149); <.001			
**Child’s average meal time**																	
	25-45 minutes	28,558		29,197		1.288 (1.257-1.319); <.001		1.258280111		<.001		0.009		1.065 (1.027-1.104); <.001			

^a^GHRS: gastrointestinal heat retention syndrome.

^b^Variables with *P*<.01 retained in the final model.

^c^Variables with VIF<10 retained.

^d^VIF: variance inflation factor.

^e^Variables with *P*<.001 retained.

^f^LASSO: Least Absolute Shrinkage and Selection Operator.

^g^α=.001; variables with nonzero values are retained.

^h^Variables with *P*<.001 retained in the final model.

### Data Preprocessing and Feature Selection

Using the dataset comprising 108,447 samples, records with missing values were excluded, yielding a complete-case sample of 49,798. Significant differences were observed in 304 out of 467 (65.1%) variables between the included and excluded pediatric groups (Table S3 in Multimedia Appendix 1). After stratified random sampling at an 8:2 ratio to create the training and test sets, the training set contained 39,838 samples, and the test set contained 9960 samples. Subsequently, the feature selection pipeline was reestablished. In the training set, after univariate correlation screening using the Pearson correlation coefficient, a total of 162 features were retained (Table S4 in Multimedia Appendix 1). Following RFE and a 10×10 cross-validation process, the optimal feature subset for each model was obtained (Table 5).

**Table 5:** Number of optimal features for each model after recursive feature elimination.

Model	Optimal features, n
Extreme Gradient Boosting	158
Linear Support Vector Machine	128
Logistic regression	123
Random Forest	158
Decision Tree	10
Naive Bayes	162
K-Nearest Neighbors	154

### Sample Size Determination

Using the maximum number of optimal features among all models, the minimum required sample size for the training set was calculated to be 3122. With the training and test sets divided at an 8:2 ratio, the corresponding minimum required test set sample size was 625 cases. Therefore, the total minimum sample size required for model development and validation was 3747 cases.

### Model Development and Validation

**Figure 2:**
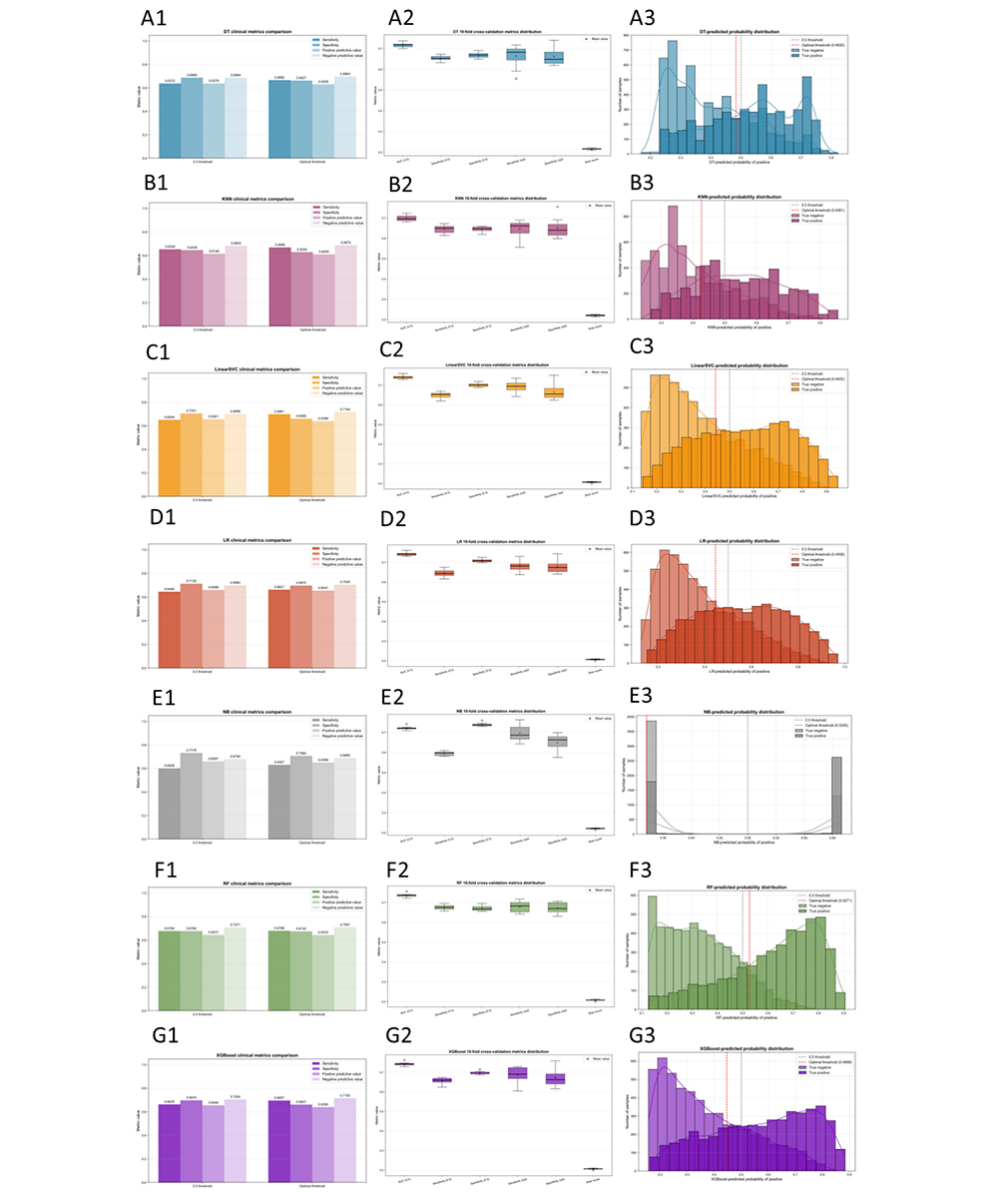
Internal validation results of seven machine learning models. A higher resolution version of this figure is included in Multimedia Appendix 4. Figures A1-G1 present the mean values of metrics including sensitivity and specificity for the DT, KNN, LinearSVC, LR, NB, RF, and XGBoost models during internal validation, respectively. Figures A2-G2 show box plots depicting the numerical distributions of AUC, sensitivity (0.5 threshold), specificity (0.5 threshold), sensitivity (optimal threshold), specificity (optimal threshold), and Brier Score for each model. Figures A3-G3 show the probability distribution diagrams for each model, presenting the number of positive and negative samples within each probability interval output by the models. These figures also display threshold lines for both the default 0.5 threshold and the optimal threshold to compare classification performance under different thresholds.

On the training set, we performed 10×10-fold internal cross-validation and hyperparameter grid search (Table S5 in Multimedia Appendix 1) to construct 7 ML models (Table 6, Figure 2). The results showed that XGBoost achieved the best AUC performance (mean 0.7417, SD 0.0086; 95% CI 0.7352-0.7482). Using 0.5 as the classification threshold, RF demonstrated the highest sensitivity (mean 0.6752, SD 0.0115; 95% CI 0.6666-0.6839), whereas NB exhibited the highest specificity (mean 0.7387, SD 0.0086; 95% CI 0.7323-0.7452). At the optimal threshold determined by the maximum Youden index, NB achieved the highest sensitivity (mean 0.6977, SD 0.0384; 95% CI 0.6688-0.7267), whereas LR demonstrated the highest specificity (mean 0.6783, SD 0.0304; 95% CI 0.6554-0.7013). For the Brier score, XGBoost exhibited the best performance (mean 0.206, SD 0.0031; 95% CI 0.2037-0.2084), with LR ranking second (mean 0.206, SD 0.0030; 95% CI 0.2037-0.2083).

**Figure 3:**
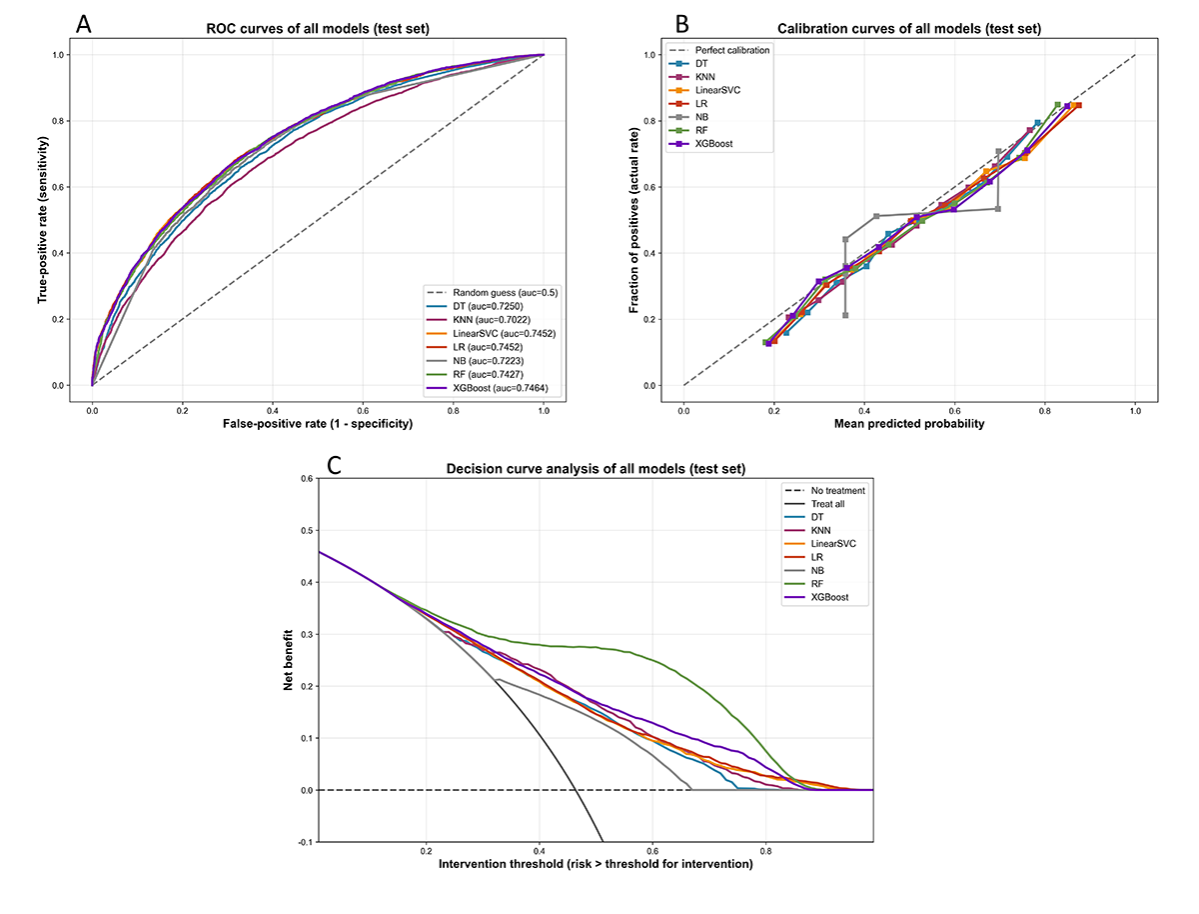
Validation results of machine learning models on the test set. Panel A displays the receiver operating characteristic (ROC) curve, with the area under the curve (AUC) representing model performance. Higher AUC values indicate stronger discriminatory power. Panel B shows the calibration curve. The horizontal coordinate indicates the predicted event incidence rate, while the vertical coordinate represents the observed actual event incidence rate. The dashed diagonal reference line indicates perfect calibration (output value = actual value). If the model output value exceeds the observed value, it indicates probability overestimation, and the calibration curve lies above the reference line. If the model output value is less than the observed value, it indicates probability underestimation, and the calibration curve lies below the reference line. The closer the calibration curve is to the reference line, the more closely the model-predicted event occurrence probability aligns with the observed actual event rate. Figure C presents the decision curve analysis (DCA), with the x-axis representing threshold probability and the y-axis representing net benefit, reflecting the model's utility across different thresholds. The black solid line and dashed line serve as reference lines, indicating the net benefits for intervening in all cases and no cases, respectively. The remaining curves depict the net benefits of using each model for assessment at various thresholds. At the same threshold probability, a higher net benefit indicates superior clinical utility of the model.

On the test set, we evaluated the discriminatory performance, calibration, and clinical utility of each model (Table 7, Figure 3). Among the discrimination metrics, the highest AUC was achieved by XGBoost; the highest specificity at the 0.5 threshold by RF; the highest sensitivity at both the 0.5 threshold and the optimal threshold by LinearSVC; and the highest specificity at the optimal threshold by NB. DeLong test results indicated statistically significant differences in AUC among the models (Table 8). Among the calibration metrics, the mean Brier scores for XGBoost, LR, and RF were closest to 0.20, with RF exhibiting the narrowest CI for the Brier score. This finding suggests smaller fluctuations in calibration performance and slightly greater stability across different sampling scenarios. Regarding the calibration slope and calibration intercept, LR and XGBoost performed slightly better than RF, indicating that RF exhibited mildly conservative calibration and a tendency to underestimate risk relative to these models. For clinical utility assessment, bootstrapped CIs for the net benefit derived from DCA (Table S6 in Multimedia Appendix 1), together with the decision curves, showed that within the threshold probability range of 0.01-0.90, the net benefit of the RF model exceeded those of both the “treat-all” and “treat-none” strategies. Within the threshold probability range of 0.13-0.85, the net benefit of the RF model was substantially higher than that of the other models, demonstrating superior clinical decision-making utility across the study’s threshold spectrum.

The above results indicate that, among the 7 ML models, the RF model did not outperform all other models across every performance metric. XGBoost and LR demonstrated slight advantages in certain discrimination or calibration metrics. According to the prespecified comprehensive utility-oriented selection criterion, RF was chosen as the primary analysis model because of its narrower CI for the Brier score, acceptable calibration performance, and relatively greater net benefit in DCA, indicating superior overall utility.

**Table 6:** Results of 10-round 10-fold internal cross-validation in the training set.

Model	Area under the receiver operating characteristic curve, mean (SD)	Sensitivity (0.5 threshold), mean (SD)	Sensitivity (optimal threshold), mean (SD)	Specificity (0.5 threshold), mean (SD)	Specificity (optimal threshold), mean (SD)	Brier score, mean (SD)
Decision Tree	0.7164 (0.0099)	0.653 (0.0122)	0.6637 (0.0512)	0.6676 (0.012)	0.6616 (0.0389)	0.215 (0.003)
K-Nearest Neighbors	0.6983 (0.0134)	0.6451 (0.0193)	0.6466 (0.0391)	0.6451 (0.0122)	0.6498 (0.0444)	0.22 (0.0037)
Linear Support Vector Machine	0.7407 (0.0084)	0.6505 (0.0149)	0.6935 (0.0292)	0.7009 (0.0092)	0.6648 (0.0361)	0.2064 (0.0029)
Logistic regression	0.7414 (0.0088)	0.6449 (0.0171)	0.6812 (0.0254)	0.7084 (0.0085)	0.6783 (0.0304)	0.206 (0.003)
Naive Bayes	0.7208 (0.0085)	0.5946 (0.0103)	0.6977 (0.0384)	0.7387 (0.0086)	0.6493 (0.0397)	0.2209 (0.0027)
Random Forest	0.7362 (0.0088)	0.6752 (0.0115)	0.6784 (0.0263)	0.6708 (0.0122)	0.6727 (0.0267)	0.208 (0.0031)
Extreme Gradient Boosting	0.7417 (0.0086)	0.657 (0.0146)	0.6876 (0.038)	0.6964 (0.008)	0.672 (0.0446)	0.206 (0.0031)

**Table 7:** Test set validation results.

Model	Area under the receiver operating characteristic curve, mean (95% CI)	Sensitivity (0.5 threshold), mean (95% CI)	Sensitivity (optimal threshold), mean (95% CI)	Specificity (0.5 threshold), mean (95% CI)	Specificity (optimal threshold), mean (95% CI)	Brier score, mean (95% CI)	Calibration slope, mean (95% CI)	Calibration intercept, mean (95% CI)
Decision Tree	0.7203 (0.7102 to 0.7296)	0.6560 (0.6425 to 0.6686)	0.7315 (0.7184 to 0.7434)	0.6633 (0.6510 to 0.6760)	0.5918 (0.5789 to 0.6047)	0.2135 (0.2105 to 0.2170)	0.7318 (0.4165 to 1.0488)	0.0548 (–0.0571 to 0.1696)
K-Nearest Neighbors	0.6892 (0.6799 to 0.6990)	0.6154 (0.6024 to 0.6287)	0.7477 (0.7366 to 0.7600)	0.6595 (0.6476 to 0.6711)	0.5317 (0.5187 to 0.5445)	0.2330 (0.2286 to 0.2374)	0.6037 (0.5742 to 0.6372)	0.1812 (0.1628 to 0.1999)
Linear Support Vector Machine	0.7454 (0.7352 to 0.7543)	0.3287 (0.3147 to 0.3421)	0.6857 (0.6735 to 0.6985)	0.9155 (0.9083 to 0.9232)	0.6776 (0.6652 to 0.6904)	0.2213 (0.2176 to 0.2256)	1.8790 (1.8337 to 1.9233)	–0.4791 (–0.5051 to –0.4526)
Logistic regression	0.7452 (0.7350 to 0.7540)	0.6445 (0.6315 to 0.6581)	0.6876 (0.6752 to 0.7006)	0.7131 (0.7013 to 0.7260)	0.6762 (0.6642 to 0.6891)	0.2047 (0.2015 to 0.2083)	1.0263 (0.9907 to 1.0575)	–0.0488 (–0.0659 to –0.028)
Naive Bayes	0.7285 (0.7181 to 0.7377)	0.5976 (0.5832 to 0.6120)	0.6886 (0.6744 to 0.7014)	0.7335 (0.7226 to 0.7452)	0.6564 (0.6442 to 0.6687)	0.3233 (0.3147 to 0.3329)	0.2649 (0.1556 to 0.3681)	0.3972 (0.3206 to 0.4753)
Random Forest	0.7421 (0.7324 to 0.7504)	0.6432 (0.6298 to 0.6569)	0.6985 (0.6850 to 0.7111)	0.7043 (0.6923 to 0.7172)	0.6599 (0.6481 to 0.6734)	0.2059 (0.2032 to 0.2089)	1.2010 (1.1698 to 1.2303)	–0.1097 (–0.1270 to –0.0911)
Extreme Gradient Boosting	0.7460 (0.7358 to 0.7545)	0.6196 (0.6063 to 0.6328)	0.6843 (0.6710 to 0.6974)	0.7312 (0.7203 to 0.7429)	0.6727 (0.6605 to 0.6858)	0.2033 (0.2003 to 0.2068)	1.0577 (1.0179 to 1.0933)	–0.0324 (–0.0498 to –0.0116)

**Table 8:** DeLong test comparison of AUC^a^ differences among models.

Model A	Model B	AUC A	AUC B	AUC Difference	DeLong *P* value
Decision Tree	K-Nearest Neighbors	0.7203	0.6892	0.0311	<.001
Decision Tree	Linear Support Vector Machine	0.7203	0.7454	–0.025	<.001
Decision Tree	Logistic regression	0.7203	0.7452	–0.0249	<.001
Decision Tree	Naive Bayes	0.7203	0.7285	–0.0081	<.001
Decision Tree	Random Forest	0.7203	0.7421	–0.0218	<.001
Decision Tree	Extreme Gradient Boosting	0.7203	0.746	–0.0257	<.001
K-Nearest Neighbors	Linear Support Vector Machine	0.6892	0.7454	–0.0562	<.001
K-Nearest Neighbors	Logistic regression	0.6892	0.7452	–0.056	<.001
K-Nearest Neighbors	Naive Bayes	0.6892	0.7285	–0.0392	<.001
K-Nearest Neighbors	Random Forest	0.6892	0.7421	–0.0529	<.001
K-Nearest Neighbors	Extreme Gradient Boosting	0.6892	0.746	–0.0568	<.001
Linear Support Vector Machine	Logistic regression	0.7454	0.7452	0.0002	<.001
Linear Support Vector Machine	Naive Bayes	0.7454	0.7285	0.0169	<.001
Linear Support Vector Machine	Random Forest	0.7454	0.7421	0.0033	<.001
Linear Support Vector Machine	Extreme Gradient Boosting	0.7454	0.746	–0.0006	<.001
Logistic regression	Naive Bayes	0.7452	0.7285	0.0168	<.001
Logistic regression	Random Forest	0.7452	0.7421	0.0031	<.001
Logistic regression	Extreme Gradient Boosting	0.7452	0.746	–0.0008	<.001
Naive Bayes	Random Forest	0.7285	0.7421	–0.0136	<.001
Naive Bayes	Extreme Gradient Boosting	0.7285	0.746	–0.0176	<.001
Random Forest	Extreme Gradient Boosting	0.7421	0.746	–0.0039	<.001

^a^AUC: area under the receiver operating characteristic curve.

### Model Description

To improve the interpretability and credibility of the established prediction model, we used the SHAP method to analyze the contribution of each variable to the model output. We also generated a probability distribution plot for the RF model on the test set to further support the reliability of its prevalence probability predictions.

**Figure 4:**
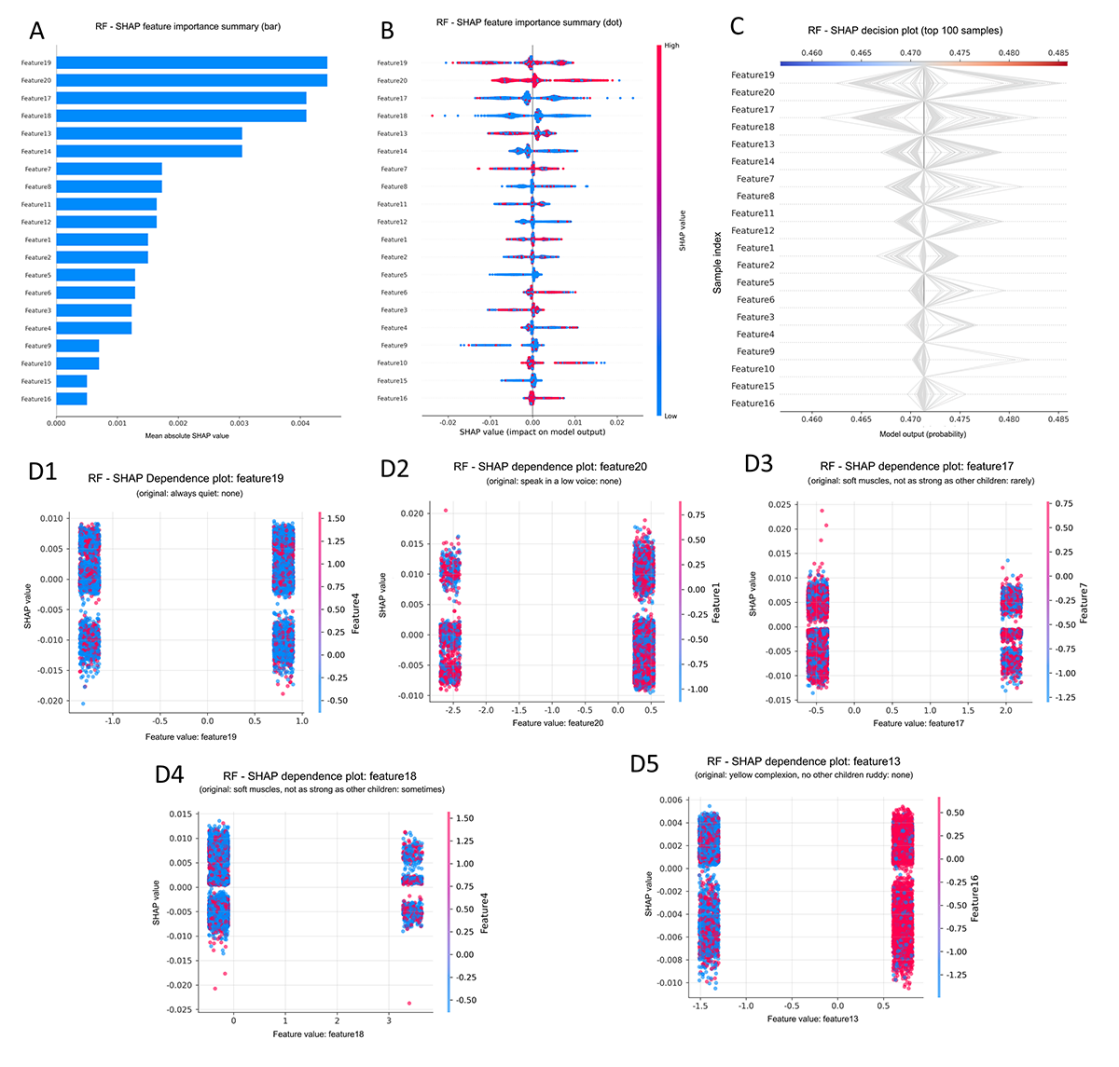
Model interpretation results using the SHAP method. (A) SHAP summary bar plots. This plot ranks average SHAP values in descending order to assess each feature's contribution to model output. (B) SHAP summary dot plots: The probability of GHRS increases with higher SHAP values of features. Each dot represents a patient's SHAP value for a given feature, with red indicating higher feature values and blue indicating lower feature values. (C) SHAP decision plots. This plot displays the decision process for the top 100 samples. The X-axis represents the model's output probability, while the Y-axis shows sample indices. Each line corresponds to a sample's decision path, with the central gray vertical line indicating the model's baseline decision value—the average predicted probability across all samples when no features are considered. Lines extending rightward from the baseline value indicate a positive SHAP value for that feature, increasing the sample's disease probability. Lines extending leftward from the baseline value indicate a negative SHAP value for that feature, decreasing the sample's disease probability. (D) SHAP dependence plots, where each plot illustrates how a single feature influences the model's output, with each point representing a patient.

The SHAP method provides model interpretability through both global and local explanations. Global interpretability characterizes overall model behavior and is illustrated by SHAP summary bar plots, SHAP summary dot plots, and SHAP decision plots, whereas local interpretability is represented by SHAP dependence plots. Given the large number of features and their lengthy names in this study, we created a feature ID-feature name mapping table (Table S7 in Multimedia Appendix 1) to facilitate simplified feature representation. For global interpretation, SHAP summary bar plots were used to evaluate feature contributions to the model based on mean absolute SHAP values and to display the top 20 contributing features in descending order (Figure 4A). SHAP summary dot plots were used to visually demonstrate the direction and magnitude of each feature’s influence on model outputs (Figure 4B). SHAP decision plots illustrated the decision-making process for the first 100 samples (Figure 4C). The 5 most influential features were Feature19 (always quiet: none), Feature20 (speak in a low voice: none), Feature17 (soft muscles, not as strong as other children: rarely), Feature18 (soft muscles, not as strong as other children: sometimes), and Feature13 (yellow complexion, not as ruddy as other children: none). Higher values for Feature19 (always quiet: none) and Feature20 (speak in a low voice: none), together with lower values for Feature17 (soft muscles, not as strong as other children: rarely), Feature18 (soft muscles, not as strong as other children: sometimes), and Feature13 (yellow complexion, not as ruddy as other children: none), were associated with a higher predicted probability of GHRS in the model output. For local interpretability, SHAP dependence plots were generated for these 5 features to further illustrate their individual effects on model predictions (Figure 4D).

**Figure 5:**
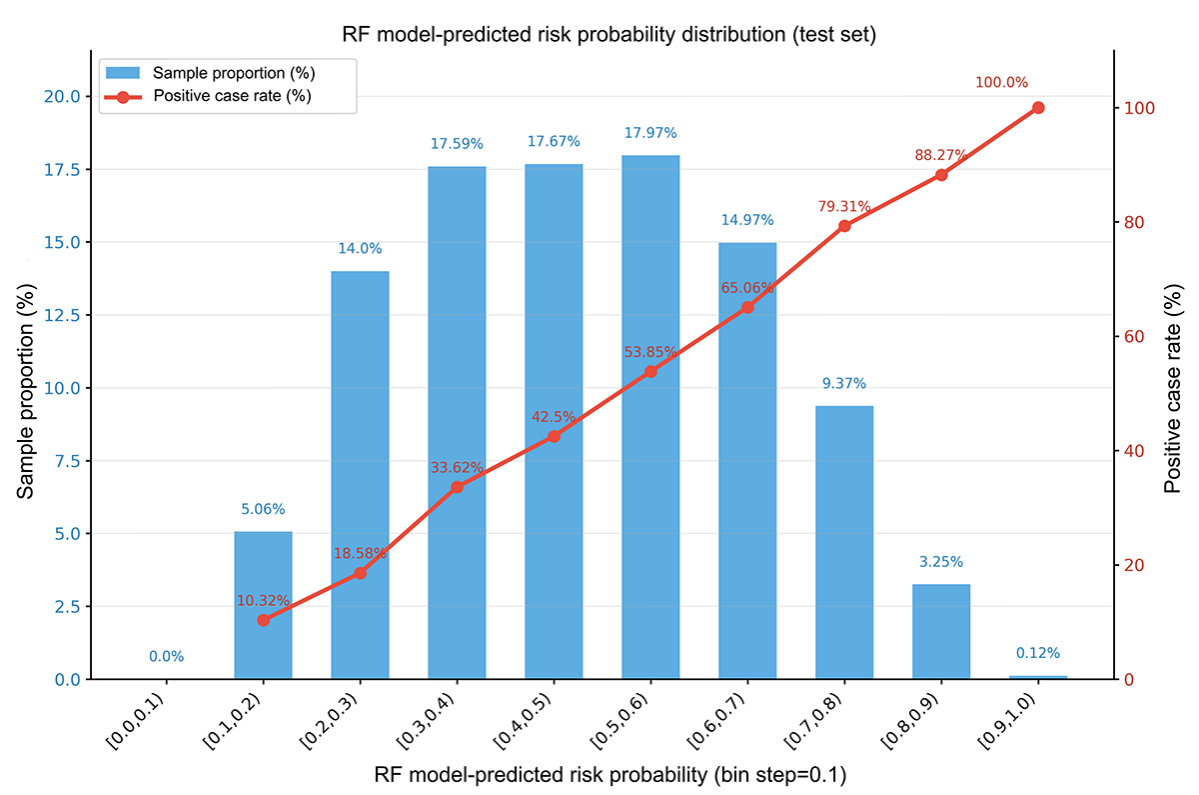
Probability distribution diagram of the random forest model. The horizontal axis of the figure denotes predicted probability bins (increment of 0.1 ranging from 0 to 1) generated by the RF model for meeting the study-specific GHRS scale criteria, representing the model-estimated probability range for each sample. Left vertical axis (blue bars): Sample Proportion (%), referring to the percentage of total samples falling within each probability bin. Right vertical axis (red line): Positive Case Rate (%), defined as the proportion of true positive samples among all samples within the corresponding probability bin.

The probability distribution plot was used to assess the agreement between the model-predicted sample distribution and the observed positive rate (Figure 5). Test-set samples were stratified into 3 subgroups according to their predicted probabilities of meeting the study-specific GHRS scale criteria: low probability (0%-30%), moderate probability (30%-60%), and high probability (60%-100%). The low-probability group (n=9960) consisted of 1898 (19.06%) test-set samples, including 311 positive cases, corresponding to an observed positive rate of 16.39%. This observed rate was substantially lower than the upper threshold of 30%, suggesting that the model was conservative within this probability range and could preliminarily identify children with a low likelihood of meeting the study-specific GHRS scale criteria. The moderate-probability group (n=9960) comprised 5302 (53.23%) test-set samples, including 2301 positive cases, corresponding to an observed positive rate of 43.41%. This finding suggests relatively good calibration performance within this probability range. The high-probability group (n=9960) included 2760 (27.71%) samples, of which 2008 were positive cases, corresponding to an observed positive rate of 72.75%. This result indicates that the RF model was able to appropriately stratify children with a high likelihood of meeting the study-specific GHRS scale criteria. Overall, the predicted probabilities of GHRS positivity generated by the RF model were consistent with the observed trend in positive rates, demonstrating satisfactory risk-stratification performance.

### Implementation of the Web Calculator

To facilitate public use, we integrated the RF model into a web application developed using the Streamlit framework for use in everyday settings. We established a definitive mapping between the 158 features included in the RF model and the questionnaire items used in the Streamlit application. The questionnaire content is consistent with the survey instrument used for data collection in this study, and all items can be understood and completed by a child’s guardian for the purpose of information collection (Table S8 in Multimedia Appendix 1). After the child’s guardian completes the electronic questionnaire consisting of 75 single-choice questions, the application automatically estimates the probability that the child currently meets the GHRS diagnostic criteria, outputs the corresponding disease probability category, and provides recommendations based on the results. This application can be accessed online [23].

## Discussion

This study analyzed independent correlates of childhood GHRS using data from a cross-sectional survey, investigated and compared 7 ML models, and developed a prevalence prediction model for childhood GHRS using the model with the greatest overall utility. The model was subsequently deployed online, enabling its use as a tool for exploratory self-assessment of the probability that a child meets the GHRS scale criteria. This tool allows caregivers to estimate the likelihood of GHRS in children based on sociodemographic characteristics, lifestyle factors, and daily manifestations.

Analysis of independent correlates revealed that male gender; only-child status; a mother’s third pregnancy; regular lunch consumption; a maternal education level of junior high school, senior high school, or technical secondary school; absence of muscle softness; absence of excessive sweating during light activity; infrequent diarrhea after consumption of raw or cold foods; and lack of parental concern about picky eating were protective factors. According to formulas used to estimate energy requirements in children and adolescents, boys have significantly higher daily total energy expenditure than girls of the same body weight. Consequently, high-calorie diets may have a weaker impact on boys than on girls [24]. A cross-sectional study conducted in Shanghai reported that children with siblings experience more childhood trauma and adverse parenting experiences, whereas children without siblings receive more attentive care [25]. Third-born children, benefiting from their later birth order, often receive increased attention, leading to more refined dietary control. Children of mothers with secondary education demonstrated a lower likelihood of GHRS than those of highly educated mothers, contradicting findings from French and Dutch studies [26,27]. This divergence may stem from regional dietary and cultural differences. Parents’ lack of concern about their children’s picky eating and consistent lunch habits indicates appropriate dietary management. The absence of muscle flaccidity and excessive sweating during minimal activity suggests that the child’s energy expenditure falls within normal limits [28,29]. Infrequent diarrhea after consuming raw or cold foods indicates a healthy digestive state [30]. Conversely, a history of functional constipation, frequent or occasional thick tongue coating, frequent sallow complexion, frequent eye redness or susceptibility to gum problems, poor self-control with tantrums during discipline, consistently eating less than peers, persistent excitability, a history of functional dyspepsia, and low activity levels were identified as major risk factors. A history of functional constipation, consistently eating less than peers, frequent sallow complexion, and a history of functional dyspepsia indicate an unhealthy gastrointestinal state or increased susceptibility to adverse factors. Research indicates that a thick, greasy tongue coating is closely associated with metabolic disorders, and its microbiota may promote gastritis and influence the development of various chronic diseases [31]. Frequent eye redness and gum discomfort represent manifestations of the body’s inflammatory response, whereas mental agitation and poor self-control may reflect excessive energy expenditure associated with excessive energy intake, thereby serving as risk factors for GHRS [32]. Analysis of independent correlates revealed associations among sociodemographic factors, children’s daily behaviors, lifestyle status, and GHRS, supporting the feasibility of developing a prevalence probability assessment model using relevant variables. To protect respondents’ privacy and respect their preferences, all items in the online questionnaire were optional, resulting in a relatively high proportion of missing data. To assess whether directly removing samples with missing values would degrade model performance, this study constructed 5 imputed datasets using multiple imputation by chained equations and combined model evaluation metrics according to Rubin’s rules for sensitivity analysis. The results showed that, after multiple imputation, the RF model exhibited AUC, sensitivity, specificity, and Brier score values comparable to those of the original-case model on the test set. Although potential selection bias persisted, the impact of excluding samples with missing values on the model conclusions remained within an acceptable range (Table S9 in Multimedia Appendix 1). Subsequently, we compared baseline characteristics across all variables between children included in and excluded from the original-case analysis. The results indicated significant differences in 304 of the 467 (65.1%) variables between the included and excluded pediatric groups (*P*<.01; Table S3 in Multimedia Appendix 1). Furthermore, we noted that the vast majority of variables in this study were binary. Following dataset imputation, some imputed values produced logical inconsistencies. For example, both “father’s first child” and “father’s third child” could be imputed as “yes” for the same participant, rendering the postimputation data clinically implausible and potentially leading to misleading model conclusions. Therefore, to ensure data validity during preprocessing, this study excluded all samples with missing values. As complete-case analysis may alter the distribution of the training set, we included SMOTETomek as a conservative resampling step in the primary analysis pipeline. However, the ratio of GHRS-positive to GHRS-negative cases in this study was nearly balanced. Thus, SMOTETomek was not required to correct severe outcome-class imbalance, and synthetic sampling could potentially affect probability calibration. We therefore conducted an additional RF sensitivity analysis without SMOTETomek. The results showed minimal differences between the 2 pipelines in AUC, Brier score, calibration slope, calibration intercept, and DCA, indicating that the main analysis conclusions were robust to the inclusion or exclusion of this resampling step (Table S9 in Multimedia Appendix 1). As the weight assigned to a given variable during model training did not fully align with the results of the multivariable logistic analysis, and because the optimal feature set varied across model types, this study did not directly use the variables identified in the independent correlates analysis as training features. Instead, we used the Pearson correlation coefficient and a 10×10-fold cross-validated RFE process to identify the optimal feature set for each model from all available variables. During model development and validation, the 7 ML models exhibited distinct performance characteristics. In the training set, performance was evaluated using 10×10-fold internal cross-validation. Model discrimination was assessed using AUC, sensitivity, and specificity, whereas calibration was preliminarily evaluated using the Brier score. The K-Nearest Neighbors model demonstrated relatively poor discriminatory performance, with an AUC below the acceptable threshold of 0.7. Among the remaining models, XGBoost exhibited slightly superior discriminatory performance and marginally better calibration. In the test set, in addition to AUC, sensitivity, specificity, and Brier score, we evaluated model performance using calibration slope, calibration intercept, calibration curves, and DCA. Although the XGBoost model showed slightly better AUC, Brier score, calibration slope, and calibration intercept values than the RF model, these differences were not statistically significant. The RF model exhibited a slightly narrower CI for the Brier score and yielded the highest net benefit in DCA, indicating superior calibration stability and greater clinical utility. Based on findings from both the training and test sets, we believe this may be attributable to the RF model generating a smoother probability distribution, with sensitivity and specificity exhibiting less fluctuation across different decision thresholds. As the intended application of this model is to assist children’s guardians in achieving greater clinical benefit through risk assessment, overall clinical utility is particularly important. Accordingly, the RF model demonstrated stable performance and greater practical value, and was ultimately selected as the optimal model. ML models are often regarded as “black boxes” because of their limited transparency and explainability, which can reduce users’ acceptance of and trust in their predictions [33]. In recent years, the demand for Explainable Artificial Intelligence (XAI), a subfield of artificial intelligence, has grown substantially. XAI aims to improve the transparency, interpretability, and accountability of model behavior. One strength of this study is the use of SHAP for model interpretation, which has become one of the most widely used XAI methods in recent years. Compared with other interpretation methods, such as Local Interpretable Model-agnostic Explanations and Gradient-weighted Class Activation Mapping, SHAP offers the advantage of providing both global and local interpretability and is better suited for tree-based models such as RF and XGBoost [34]. In this study, SHAP was used to effectively illustrate the functionality of the RF model and explain how the RF model evaluates children based on features contained in the input data. The SHAP results indicated that children who are restless, speak loudly, have nonsoft muscles, and exhibit a sallow complexion are assigned a higher probability of current GHRS by the RF model. These findings are consistent with the independent correlates analysis and reflect characteristics that guardians can readily assess through repeated observation. Compared with similar studies, the explainability methods used in this research are relatively limited [34,35]. This approach was adopted because the primary users of the model and the intended audience for its explainability analyses are children’s guardians. Considering the nonmedical background of the target users, detailed interpretation results may be relatively difficult to understand and may offer greater reference value than practical utility. Therefore, rather than incorporating additional explainability methods, we included probability distribution plots to visually demonstrate the consistency between the disease probabilities predicted by the RF model and the observed trend in positive rates, thereby enhancing confidence in the model’s output. Furthermore, using the Streamlit framework, we integrated the RF model into a user-friendly online assessment platform. The platform collects the required evaluation information through a series of single-choice questions, with validation prompts displayed for unanswered items to ensure complete and structured data entry and to prevent logical inconsistencies. All questions are completed by the child’s guardian based on subjective judgment, consistent with the data collection approach used during model training, making the platform suitable for widespread use in routine health monitoring settings. Upon submission of the completed questionnaire, the platform displays the estimated probability of GHRS and the corresponding risk classification. Lifestyle recommendations are also provided to guide adjustments to the child’s daily habits and diet. These features demonstrate the feasibility of the model for large-scale dissemination and practical application.

The limitations of this study primarily include the following. First, to protect participant privacy and respect respondents’ preferences, all questionnaire items were optional, resulting in a substantial number of samples with missing values. As most features in this study were binary categorical variables, missing-value imputation produced clinically implausible contradictions among certain variables. To ensure data completeness and clinical relevance, we were required to exclude more than half of the samples containing missing values. Although the final sample size still substantially exceeded the required sample size, this approach resulted in considerable data loss and may have introduced some degree of bias into the findings. Second, although the model was developed using data from children in Longgang District, Shenzhen, China, and its performance was evaluated through internal cross-validation and validation in an independent test set, the test set samples were collected during the same period and from the same geographic region. In the absence of external validation across different periods or regions, there is insufficient evidence to confirm the model’s generalizability to other populations, despite the advantage of a large sample size. Third, all features used to develop the model consisted of sociodemographic information and data readily available in daily life. Although this enhances the model’s potential for widespread use in routine settings, the limited predictive relevance of these features suggests that there remains room for improvement in discriminatory performance. Fourth, the prevalence probability assessment models developed in this study were trained exclusively using traditional ML approaches. No reinforcement learning techniques were applied to optimize model performance, which may represent a methodological limitation [20]. Fifth, the use of SMOTETomek warrants cautious interpretation. As the outcome classes in this study were nearly balanced, resampling was not necessary to address severe class imbalance. Although sensitivity analyses demonstrated no meaningful deterioration in model performance, synthetic samples may still influence probability calibration. Future external validation studies and prospective evaluations should further compare the performance of simplified model pipelines that do not incorporate resampling. Finally, an important limitation is that GHRS status was determined using a self-rated diagnostic scale developed by the research team and completed by children’s guardians, rather than using an independent clinical reference standard. Although items from the diagnostic scale were excluded from model training, both the outcome and candidate predictors were derived from the same questionnaire source. This may have introduced shared-method bias and a degree of circularity in construct definition. Therefore, this model should be interpreted as estimating the probability that a child meets the study-specific GHRS scale criteria, rather than as an independent diagnostic tool for a clinically validated disease entity. To date, our team has made meaningful progress in GHRS-related research, including its definition, clinical manifestations, associated diseases, diagnostic approaches, pathogenic mechanisms, and risk factors, thereby establishing a preliminary research framework. Future efforts should focus on identifying and incorporating features more strongly associated with GHRS, expanding datasets to include samples from diverse geographic regions and periods, and integrating advanced modeling approaches, such as ensemble boosting techniques, into model development and validation. These improvements may further enhance the model’s predictive accuracy and clinical utility. Despite these limitations, the assessment model developed in this study remains practically useful for household self-assessment.

In summary, we successfully developed an explainable ML model to estimate the prevalence probability of GHRS in children using sociodemographic and clinical questionnaire data. During internal validation, the RF model demonstrated moderate discriminatory performance and acceptable calibration. It may serve as an exploratory tool for guardians to assess the likelihood that a child meets the study-specific GHRS scale criteria. However, external validation, usability testing, and prospective evaluation are required before implementation in clinical, community, or public health settings. In addition, this study differs from traditional approaches that either develop diagnostic models based on disease-specific symptoms using small cross-sectional samples or construct future disease risk prediction models using longitudinal cohort data. Instead, we used a large cross-sectional dataset to develop a GHRS prevalence probability assessment model based on sociodemographic characteristics and observable daily manifestations beyond disease-specific signs and symptoms. The predicted probability generated by the model reflects association-based estimates derived from the cross-sectional dataset. Models developed using this approach reduce reliance on professionals and specialized equipment for assessing disease-specific symptoms while quantifying disease probability using readily obtainable sociodemographic and behavioral information. As such, they may support exploratory self-assessment and hypothesis-generating population-level evaluations in settings involving nonprofessional users. Nevertheless, the practical screening value of this tool requires further confirmation through external validation, usability testing, and prospective studies. Future research should include external validation across different geographic regions, periods, and independent populations, as well as evaluation of the model’s usability and real-world impact in household and primary health care settings.

## Appendix

Multimedia Appendix 1 Additional analysis.

Multimedia Appendix 2 TRIPOD+AI (Transparent Reporting of a Multivariable Prediction Model for Individual Prognosis or Diagnosis + Artificial Intelligence) abstract reporting checklist.

Multimedia Appendix 3 TRIPOD+AI (Transparent Reporting of a Multivariable Prediction Model for Individual Prognosis or Diagnosis + Artificial Intelligence) prediction model research report checklist.

Multimedia Appendix 4 High resolution version of Figure 2.

## Abbreviations

AUC: area under the receiver operating characteristic curve
DCA: decision curve analysis
EPV: events per variable
GHRS: gastrointestinal heat retention syndrome
Iba-1: ionized calcium-binding adapter molecule 1
IL: interleukin
ImbPipeline: Imbalanced Learning Pipeline
LASSO: Least Absolute Shrinkage and Selection Operator
LinearSVC: Linear Support Vector Machine
LR: logistic regression
ML: machine learning
NB: Naive Bayes
RF: Random Forest
RFE: recursive feature elimination
SHAP: Shapley Additive Explanations
SMOTETomek: Synthetic Minority Oversampling Technique Tomek Links
TRIPOD+AI: Transparent Reporting of a Multivariable Prediction Model for Individual Prognosis or Diagnosis + Artificial Intelligence
VIF: variance inflation factor
XAI: Explainable Artificial Intelligence
XGBoost: Extreme Gradient Boosting

## Data Sharing

The study protocol, analytical dataset, complete model code, and model files will be made available upon reasonable request to the corresponding author (XG), subject to approval.
