# Supplementary material for: Development and Validation of an Explainable Machine Learning Model to Assess the Prevalence Probability of Gastrointestinal Heat Retention Syndrome in Children: Cross-Sectional Study
Source: J Med Internet Res. 2026 Jul 2;28:e94775. doi: 10.2196/94775 (PMC13376857; doi:10.2196/94775)
Supplement: Multimedia Appendix 1 [file jmir_v28i1e94775_app1.docx]

# Supplementary Materials

Figure S1. Comparison of continuous variables before and after outlier processing. Page1

Table S1. Continuous Variable Processing Results and Distributions Page2

Table S2.Included variables and screening results of independent risk factors Page3

Table S3.Comparison of Baseline Characteristics Between Included vs. Excluded Children in Model Development Page33

Table S4.Results of Univariate Correlation Screening Using Pearson Correlation Coefficients Page106

Table S5.Hyperparameter Grid and Selected Values Page124

Table S6.Confidence Intervals for DCA Net Benefit Were Calculated Page125

Table S7.Mapping Table of Feature IDs to Feature Names in the RF Model Page135

Table S8.Mapping Relationship Between RF Model Features and Streamlit Application Questionnaire Items Page 139

Table S9.Results of MICE Imputation and Sensitivity Analysis Page147

Table S10.Tripod+AI Checklist Page148

Table S11. Tripod+AI Prediction Model Reporting Checklist Page149


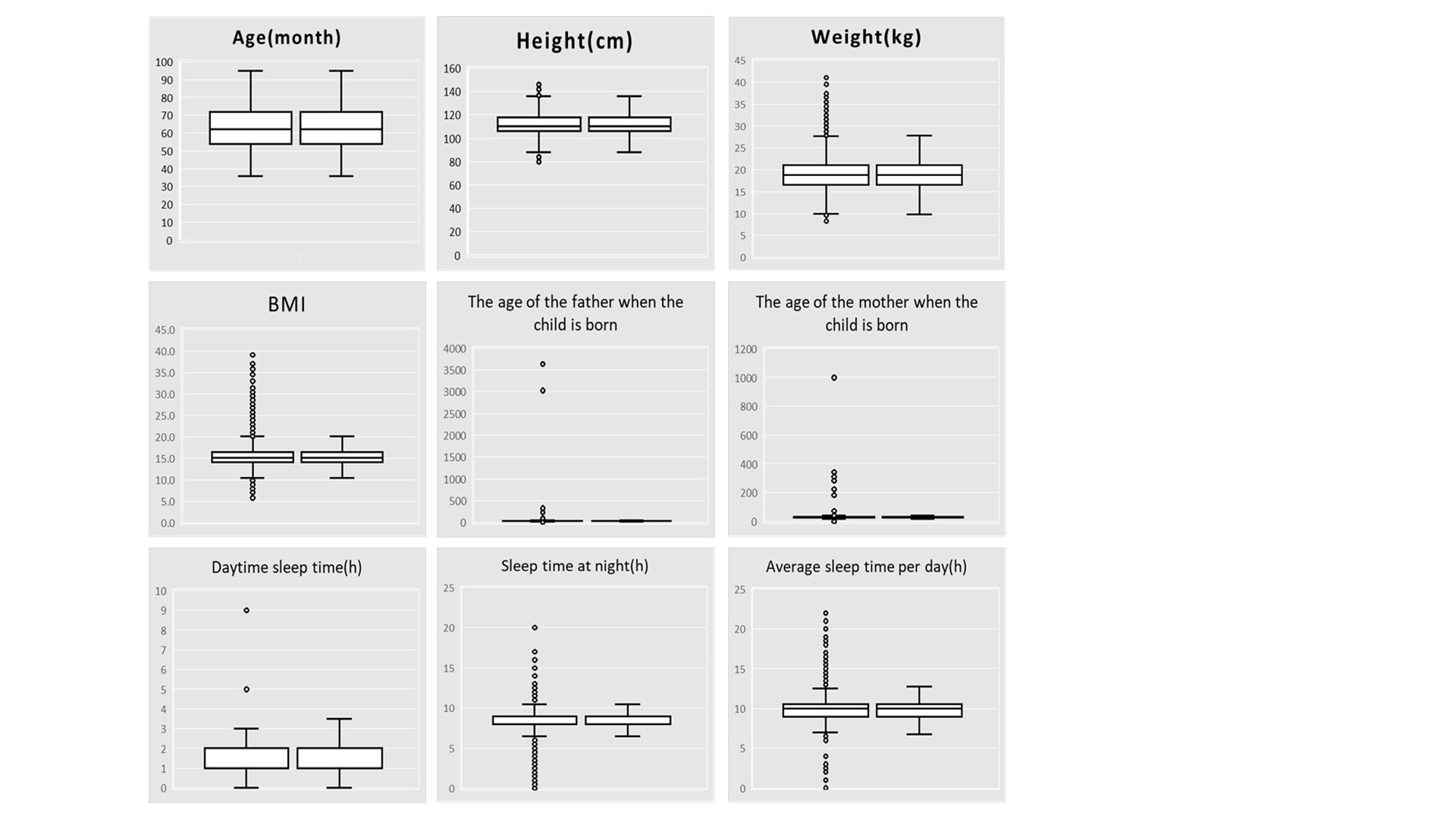


Figure S1. Comparison of continuous variables before and after outlier treatment. This figure illustrates the distribution of continuous variables before and after outlier processing. Outliers were defined as values exceeding the upper quartile (Q3) + 1.5 times the interquartile range (IQR), or falling below the lower quartile (Q1) - 1.5 times the IQR; During processing, such outliers were replaced with corresponding boundary values to align them more closely with the main data distribution. Box plots display the distributions of each variable before (Before) and after (After) treatment, including the median, interquartile range, and extremes, facilitating visual assessment of distribution changes following outlier treatment. Before treatment ( Left ); After treatment ( Right ).

**Table S1. Processing Results and Distribution of Continuous Variables**

|  | Q1 | Q3 | IQR | Lower bound | Upper bound | Median |
| --- | --- | --- | --- | --- | --- | --- |
| Age(Month) | 54 | 72 | 18 | 27 | 99 | 62 |
| Height(cm) | 106 | 118 | 12 | 88 | 136 | 110 |
| Weight(kg) | 16.5 | 21 | 4.5 | 9.75 | 27.75 | 18.8 |
| BMI | 14.04959 | 16.47359 | 2.424002 | 10.41358 | 20.10959 | 15.1 |
| The age of the father when the child is born | 28 | 35 | 7 | 17.5 | 45.5 | 31 |
| The age of the mother when the child is born | 26 | 32 | 6 | 17 | 41 | 29 |
| Daytime sleep time(h) | 1 | 2 | 1 | -0.5 | 3.5 | 2 |
| Sleep time at night(h) | 8 | 9 | 1 | 6.5 | 10.5 | 9 |
| Average sleep time per day(h) | 9 | 10.5 | 1.5 | 6.75 | 12.75 | 10 |

**Table S2. Included Variables and Independent Risk Factors Screening Results (Suffix '**' indicates excluded indicators)**

| Feature | None-GHRS,  N=56815 | GHRS,  N=51632 | OR (Univariate logistic)  (*P<*.01 retained) | Collinearity Statistics VIF (VIF<10 retained) | Stepwise Regression Wald Test  (*P<*.001 retained) | LASSO Regression Analysis (Alpha=0.001, variables with non-zero values retained) | OR (Multivariate logistic)  (*P<*.001 retained) |
| --- | --- | --- | --- | --- | --- | --- | --- |
| Categorical variables | | | | | | | |
| Grade | | | | | | | |
| Small class | 16126 | 17565 | 1.301(1.268-1.335,*P<*.001) | 91.717763** |  |  |  |
| Middle Class | 18329 | 17219 | 1.051(1.024-1.078,*P<*.001) | 93.19983226** |  |  |  |
| Senior Class | 22223 | 16726 | 0.746(0.727-0.765,*P<*.001) | 96.70606203** |  |  |  |
| Other | 137 | 122 | 0.980(0.767-1.251,*P=.*87)** |  |  |  |  |
| Gender | | | | | | | |
| Female | 25399 | 25424 | refer |  |  |  |  |
| Male | 31416 | 26208 | 0.833 (0.814-0.854)  *P<*.001） | 1.011477787 | *P<*.001 | -0.05 | 0.776(0.750-0.803,*P<*.001) |
| Age(year) | | | | | | | |
| 3 | 4137 | 4691 | 1.272(1.218-1.329,*P<*.001) | 1.940612827 | *P<*.001 | 0.038 | 1.264(1.183-1.351,*P<*.001) |
| 4 | 18854 | 19404 | 1.212(1.182-1.243,*P<*.001) | 2.727345876 | *P<*.001 | 0.022 | 1.149(1.103-1.198,*P<*.001) |
| 5 | 18049 | 15755 | 0.943(0.919-0.968,*P<*.001) | 1897000000000** |  |  |  |
| 6 | 15291 | 11483 | 0.777 (0.755–0.799, *P<* .001) | 2.405937227 | *P<*.001 | -0.013 | 0.930 (0.889–0.973, *P =* .002)** |
| 7 | 484 | 299 | 0.678 (0.587–0.783, *P<* .001) | 1.055110288 | *P=*.03** |  |  |
| Time of birth | | | | | | | |
| 1:00-3:00 | 4522 | 4082 | 0.993 (0.950–1.037, *P =* 7.46)** |  |  |  |  |
| 3:00-5:00 | 3643 | 3227 | 0.973 (0.927–1.022, *P =* .27)** |  |  |  |  |
| 5:00-7:00 | 4308 | 3711 | 0.944 (0.902–0.988, *P =* .01)** |  |  |  |  |
| 7:00-9:00 | 5793 | 5144 | 0.975 (0.937–1.014, *P =* .20)** |  |  |  |  |
| 9:00-11:00 | 9291 | 8069 | 0.947 (0.917–0.979, *P =* .001) | 1.027868913 | *P=.*16** |  |  |
| 11:00-13:00 | 5829 | 5264 | 0.993 (0.955–1.033, *P =* .73)** |  |  |  |  |
| 13:00-15:00 | 4861 | 4454 | 1.009 (0.967–1.053, *P =* .68)** |  |  |  |  |
| 15:00-17:00 | 5130 | 4829 | 1.040 (0.998–1.083, *P =* .07)** |  |  |  |  |
| 17:00-19:00 | 4150 | 3910 | 1.040(0.994-1.088,*P=*.09)** |  |  |  |  |
| 19:00-21:00 | 3601 | 3352 | 1.026(0.977-1.077,*P=*.30)** |  |  |  |  |
| 21:00-23:00 | 2967 | 2921 | 1.088(1.033-1.147,*P=*.002) | 1.017540661 | *P=*.154** |  |  |
| 23:00-1:00 | 2720 | 2669 | 1.084(1.026-1.145*,P=*.004) | 1.015844995 | *P=*.111** |  |  |
| Ethnic group | | | | | | | |
| Ethnic minority | 2697 | 2380 | refer |  |  |  |  |
| Han ethnicity | 54118 | 49252 | 1.031(0.975-1.091,*P=.*29)** |  |  |  |  |
| Only child | | | | | | | |
| No | 13653 | 15639 | refer |  |  |  |  |
| Yes | 43162 | 35993 | 0.728(0.709-0.748,*P<*.001) | 1.395246244 | *P<*.001 | -0.023 | 0.896(0.863-0.931,*P<*.001) |
| Term infant | | | | | | | |
| No | 2942 | 3031 | refer |  |  |  |  |
| Yes | 53873 | 48601 | 0.876(0.831-0.923,*P<*.001) | 1.220015467 | *P=*.09** |  |  |
| Caesarean birth | | | | | | | |
| No | 36467 | 33212 | refer |  |  |  |  |
| Yes | 20326 | 18403 | 0.994(0.970-1.019,*P=*.64)** |  |  |  |  |
| Birth weight | | | | | | | |
| 2.5-4kg | 50085 | 45891 | 1.074(1.035-1.115,*P<*.001) | 1.887399742 | *P=*.45** |  |  |
| <2.5kg | 4014 | 3694 | 1.014(0.968-1.062,*P=.*57)** |  |  |  |  |
| ＞4kg | 2716 | 2047 | 0.822(0.775-0.872,*P<*.001) | 1.712445983 | *P=.*007** |  |  |
| Which child of father | | | | | | | |
| First | 26808 | 29295 | 1.477(1.441-1.514,*P<*.001) | 11400000000000** |  |  |  |
| Second | 23364 | 18004 | 0.752(0.734-0.771,*P<*.001) | 11.81976138** |  |  |  |
| Third | 3292 | 1840 | 0.596(0.592-0.632,*P<*.001) | 5.666151162 | *P=*.51** |  |  |
| Fourth or more | 645 | 357 | 0.602(0.529-0.686,*P<*.001) | 2.301231362 | *P=.*33** |  |  |
| Which child of mother | | | | | | | |
| First | 27142 | 29701 | 1.492(1.456-1.529,*P<*.001) | .** |  |  |  |
| Second | 23277 | 17787 | 0.744(0.725-0.763,*P<*.001) | 11.79354658** |  |  |  |
| Third | 3167 | 1723 | 0.580(0.547-0.616,*P<*.001) | 5.626435311 | *P<*.001 | -0.015 | 0.857(0.787-0.933,*P<*.001) |
| Fourth or more | 580 | 311 | 0.584 (0.508–0.670, *P<* .001) | 2.274996933 | *P=*.004** |  |  |
| Exclusively breast fed within 6 months after birth | | | | | | | |
| No | 17367 | 18638 | refer |  |  |  |  |
| Yes | 39448 | 32994 | 0.779 (0.760–0.799, *P<* .001) | 12.94911128** |  |  |  |
| Subgroup: Fresh milk | | | | | | | |
| No | 56409 | 51291 | refer |  |  |  |  |
| Yes | 406 | 341 | 0.924 (0.799–1.067, *P =* .28)** |  |  |  |  |
| Subgroup: Fresh ewe milk | | | | | | | |
| No | 56623 | 51496 | refer |  |  |  |  |
| Yes | 192 | 136 | 0.779 (0.625–0.971, *P =* .03)** |  |  |  |  |
| Subgroup: Formula milk powder | | | | | | | |
| No | 41042 | 34535 | refer |  |  |  |  |
| Yes | 15773 | 17097 | 1.288 (1.255–1.322, *P<* .001) | 8.048 | *P<*.001 | 0.029 | 1.155 (1.114–1.198, *P<* .001) |
| Subgroup: Soymilk | | | | | | | |
| No | 56755 | 51596 | refer |  |  |  |  |
| Yes | 60 | 36 | 0.660 (0.437–0.998, *P =* .05)** |  |  |  |  |
| Subgroup: Ewe milk power | | | | | | | |
| No | 56291 | 51116 | refer |  |  |  |  |
| Yes | 524 | 516 | 1.084 (0.960–1.225, *P =* .19)** |  |  |  |  |
| Subgroup: Others | | | | | | | |
| No | 55357 | 50012 | refer |  |  |  |  |
| Yes | 1458 | 1620 | 1.230(1.145-1.321,*P<*.001) | 1.609720932 | *P=*.003** |  |  |
| Time of adding animal protein complementary food | | | | | | | |
| After 6 months | 45200 | 41619 | 1.068(1.037-1.100,*P<*.001) | . |  |  |  |
| 4-6 months | 10923 | 9547 | 0.953(0.924-0.983,*P=*.002) | 1.007555717 | *P=*.75** |  |  |
| Within 3 months | 692 | 466 | 0.739(0.656-0.831,*P<*.001) | 1.007779603 | *P<*.001=0.77** |  |  |
| Have been treated with antibiotics | | | | | | | |
| No | 35915 | 26481 | refer |  |  |  |  |
| Yes | 15773 | 17097 | 1.288 (1.255–1.322, *P<* .001) | 12.18493903** |  |  |  |
| The earliest age of using antibiotics | | | | | | | |
| Never used | 35915 | 26481 | 0.578(0.561-0.595,*P<*.001) | .** |  |  |  |
| Within 1 year | 3515 | 4999 | 1.718(1.642-1.798,*P<*.001) | .** |  |  |  |
| 1-2 years | 5389 | 6897 | 1.562(1.503-1.623,*P<*.001) | 1.117598504 | *P<*.001 | 0.025 | 1.135(1.083-1.191,*P<*.001) |
| After 3 years | 2981 | 3274 | 1.283(1.218-1.350,*P<*.001) | 1.072831216 | *P<*.001 | 0** |  |
| Frequency of antibiotic use within 1 year old | | | | | | | |
| 0 times | 44285 | 36652 | 0.585(0.554-0.617,*P<*.001) | .** |  |  |  |
| 1 time | 1352 | 1646 | 1.437(1.336-1.547,*P<*.001) | 1.041712693 | *P<*.001 | 0** |  |
| 2 times | 486 | 758 | 1.834(1.636-2.057,*P<*.001) | 1.019787733 | *P<*.001 | 0** |  |
| ≥3 times | 547 | 972 | 2.098(1.888-2.332,*P<*.001) | 1.035759364 | *P<*.001 | 0.002 | 1.228(1.080-1.397,*P=*.002)** |
| Children usually have the most contact with people outside kindergarten time | | | | | | | |
| Father | 5406 | 4553 | 0.920(0.882-0.959,*P<*.001) | 10.41374661** |  |  |  |
| Mother | 43687 | 38587 | 0.889(0.864-0.914,*P<*.001) | 20.74171103** |  |  |  |
| Father/Mother | 516 | 380 | 0.809(0.708-0.924,*P=*.002) | 1.874523283 | *P=*.10** |  |  |
| Grandparents (Paternal grandparents, Maternal grandparents) | 5477 | 6423 | 1.332 (1.282–1.384, *P<* .001) | 12.006738** |  |  |  |
| Mixed (parents + grandparents) | 1132 | 1160 | 1.131 (1.041–1.228, *P =* .004) | 3.103811654 | *P=*.39** |  |  |
| Other | 597 | 529 | 0.975 (0.867–1.096, *P =* .67)** |  |  |  |  |
| History of allergies | | | | | | | |
| No | 42690 | 32995 | refer |  |  |  |  |
| Yes | 14125 | 18637 | 1.707 (1.663–1.752, *P<* .001) | 5.320354458 | *P<*.001 | 0.032 | 1.109 (1.058–1.164, *P<* .001) |
| Subgroup: Eczema or atopic dermatitis | | | | | | | |
| No | 50412 | 42638 | refer |  |  |  |  |
| Yes | 6403 | 8994 | 1.661 (1.604–1.719, *P<* .001) | 2.486610432 | *P=*.001** |  |  |
| Subgroup: Allergic rhinitis | | | | | | | |
| No | 50733 | 42837 | refer |  |  |  |  |
| Yes | 6082 | 8795 | 1.713 (1.654–1.774, *P<* .001) | 2.418753498 | *P<*.001 | 0.001 | 1.012 (0.949–1.079, *P =* .72)** |
| Subgroup: Drug allergy | | | | | | | |
| No | 55791 | 50440 | refer |  |  |  |  |
| Yes | 1024 | 1192 | 1.288 (1.183–1.401, *P<* .001) | 1.161600891 | *P=*.91** |  |  |
| Subgroup: Food allergy | | | | | | | |
| No | 54110 | 48135 | refer |  |  |  |  |
| Yes | 2705 | 3497 | 1.453 (95% CI: 1.380-1.530; *P<* .001) | 1.409002535 | *P=*.12** |  |  |
| Subgroup: Other allergic history | | | | | | | |
| No | 55959 | 50491 | refer |  |  |  |  |
| Yes | 856 | 1141 | 1.477 (95% CI: 1.351-1.616; *P<* .001) | 1.213242246 | *P=*.78** |  |  |
| History of digestive system diseases | | | | | | | |
| No | 55527 | 48058 | refer |  |  |  |  |
| Yes | 1288 | 3574 | 3.206 (95% CI: 3.005-3.421; *P<* .001) | 12.02086725** |  |  |  |
| Subgroup: Functional dyspepsia | | | | | | | |
| No | 56357 | 50224 | refer |  |  |  |  |
| Yes | 458 | 1408 | 3.450 (95% CI: 3.102-3.836; *P<* .001) | 4.367928488 | *P<*.001 | 0.022 | 1.458 (95% CI: 1.250-1.701; *P<* .001) |
| Subgroup: Functional constipation | | | | | | | |
| No | 56676 | 50694 | refer |  |  |  |  |
| Yes | 139 | 938 | 7.545 (95% CI: 6.311-9.019; *P<* .001) | 2.635422558 | *P<*.001 | 0.1 | 3.909 (95% CI: 3.040-5.026; *P<* .001)** |
| Subgroup: Gastritis | | | | | | | |
| No | 56683 | 51378 | refer |  |  |  |  |
| Yes | 132 | 254 | 2.123 (95% CI: 1.720-2.621; *P<* .001) | 1.425311819 | *P=*.05** |  |  |
| Subgroup: Gastroesophageal reflux | | | | | | | |
| No | 56790 | 51570 | refer |  |  |  |  |
| Yes | 25 | 62 | 2.731(1.716-4.346,*P<*.001) | 1.132754785 | *P=*.21** |  |  |
| Subgroup: Peptic ulcer | | | | | | | |
| No | 56805 | 51606 | refer |  |  |  |  |
| Yes | 10 | 26 | 2.862(1.380-5.935,*P=*.01) | 1.012439217 | *P=*.75** |  |  |
| Subgroup: Mesenteric lymphadenitis | | | | | | | |
| No | 56352 | 50725 | refer |  |  |  |  |
| Yes | 463 | 907 | 2.176(1.945-2.436,*P<*.001) | 3.584629175 | *P<*.001 | 0** |  |
| Subgroup: Other digestive system diseases | | | | | | | |
| No | 56642 | 51172 | refer |  |  |  |  |
| Yes | 173 | 460 | 2.943(2.470-3.507,*P<*.001) | 2.360226253 | *P<*.001 | 0** |  |
| History of major diseases | | | | | | | |
| No | 55418 | 50421 | refer |  |  |  |  |
| Yes | 591 | 647 | 1.203(1.076-1.346,*P=*.001) | 1.007446597 | *P=*.021** |  |  |
| Activity habits | | | | | | | |
| Minimal activity | 14 | 42 | 3.283(1.793-6.011,*P<*.001) | 1.003943736 | *P=*.02** |  |  |
| Low activity level | 1432 | 2839 | 2.237(2.097-2.387,*P<*.001) | 1.10115061 | *P<*.001 | 0.046 | 1.418(1.288-1.561,*P<*.001) |
| Moderate activity level | 27703 | 27676 | 1.204(1.175-1.233,*P<*.001) | 1.151989396 | *P<*.001 | 0.021 | 1.144(1.104-1.185,*P<*.001) |
| Higher activity level | 26220 | 20074 | 0.730(0.713-0.748,*P<*.001) | .** |  |  |  |
| Physical balance and flexibility in the last year | | | | | | | |
| Acceptable flexibility | 685 | 1462 | 2.368(2.160-2.595,*P<*.001) | 1.042165536 | *P<*.001 | 0** |  |
| Good flexibility | 37440 | 36666 | 1.256(1.222-1.290,*P<*.001) | .** |  |  |  |
| Excellent flexibility | 16352 | 11825 | 0.723(0.703-0.743,*P<*.001) | 1.192420042 | *P<*.001 | 0** |  |
| Like music or not | | | | | | | |
| Strong dislike | 484 | 301 | 0.678 (0.587–0.783, *P<* .001) | 1.014266489 | *P=*.98** |  |  |
| Dislike | 257 | 204 | 0.867(0.721-1.042,*P=*.13)** |  |  |  |  |
| Neutral, no particular response to music | 14042 | 12514 | 0.965(0.939-0.992,*P=*.01)** |  |  |  |  |
| Like | 31162 | 29160 | 1.053(1.028-1.079,*P<*.001) | 1.018475704 | *P=*.040** |  |  |
| Strong preference | 9263 | 8346 | 0.981(0.950-1.014,*P=*.26)** |  |  |  |  |
| Regular lunch breaks | | | | | | | |
| No | 14204 | 17569 | refer |  |  |  |  |
| Yes | 42611 | 34063 | 0.646 (0.630-0.664, *P<* .001) | 1.039852719 | *P<*.001 | -0.034 | 0.848(0.815-0.882,*P<*.001) |
| Food preference | | | | | | | |
| No significant dietary preference | 40073 | 28738 | 0.524(0.511-0.538,*P<*.001) | .** |  |  |  |
| Strong preference for certain foods | 11662 | 14687 | 1.539(1.497-1.583,*P<*.001) | 1.090151058 | *P<*.001 | 0.025 | 1.144(1.096-1.193,*P<*.001) |
| Strong aversion to certain foods | 5080 | 8207 | 1.925(1.854-1.998,*P<*.001) | 1.090358017 | *P<*.001 | 0.035 | 1.182(1.116-1.251,*P<*.001) |
| Supplementing nutrients separately from daily diet | | | | | | | |
| No | 44156 | 36587 | refer |  |  |  |  |
| Yes | 12659 | 15045 | 1.434(1.396-1.474,*P<*.001) | 1.060012229 | *P<*.001 | 0.009 | 1.055(1.015-1.096,*P=*.007)** |
| With dental caries | | | | | | | |
| No | 24898 | 21499 | refer |  |  |  |  |
| Yes | 24868 | 24122 | 1.123(1.095-1.152,*P<*.001) | 1.431002746 | *P<*.001 | 0.003 | 1.047(1.012-1.084,*P=*.008)** |
| Dental caries have been treated | | | | | | | |
| Never oral treatment | 37877 | 34242 | 0.985(0.960-1.010,*P=*.23)** |  |  |  |  |
| Occasional oral treatment | 9350 | 8986 | 1.070(1.036-1.104,*P<*.001) | 1.399633879 | *P=*.01** |  |  |
| Frequent oral treatment | 1305 | 1400 | 1.186(1.098-1.280,*P<*.001) | 1.091307102 | *P=*.40** |  |  |
| Whether have abnormal vision | | | | | | | |
| No | 50428 | 44073 | refer |  |  |  |  |
| Yes | 6387 | 7559 | 1.354(1.307-1.403,*P<*.001) | 8.829929509 | *P=*.03** |  |  |
| Subgroup: Normal_vision | | | | | | | |
| No | 6387 | 7559 | refer |  |  |  |  |
| Yes | 50428 | 44073 | 0.738(0.713-0.765,*P<*.001) | .** |  |  |  |
| Subgroup: short sighted | | | | | | | |
| No | 56156 | 50946 | refer |  |  |  |  |
| Yes | 659 | 686 | 1.147(1.030-1.278,*P=*.01)** |  |  |  |  |
| Subgroup: Amblyopia | | | | | | | |
| No | 55341 | 50114 | refer |  |  |  |  |
| Yes | 1474 | 1518 | 1.137(1.058-1.223,*P<*.001) | 2.520190064 | *P=*.52** |  |  |
| Subgroup: Astigmatism | | | | | | | |
| No | 55496 | 50145 | refer |  |  |  |  |
| Yes | 1319 | 1487 | 1.248(1.157-1.345,*P<*.001) | 2.567336823 | *P=*.81** |  |  |
| Subgroup: Other visual abnormalities | | | | | | | |
| No | 54669 | 49205 | refer |  |  |  |  |
| Yes | 2146 | 2427 | 1.257(1.184-1.333,*P<*.001) | 5.369832928 | *P=*.99** |  |  |
| Has your child ever received or is currently receiving vision correction therapy? | | | | | | | |
| No | 52352 | 46252 | refer |  |  |  |  |
| Yes | 2089 | 2062 | 1.090(1.024-1.159,*P=*.01) | 4.196512643 | *P<*.001 | 0.007 | 1.017(1.007-1.027,*P<*.001) |
| Language comprehension and expression ability | | | | | | | |
| Can comprehend speech but not yet articulate clearly | 1936 | 2615 | 1.499(1.411-1.591,*P<*.001) | 1.061112516 | *P=*.004** |  |  |
| Can understand speech intonation and tone, and respond clearly | 29298 | 27224 | 1.029(1.004-1.054,*P=*.02)** |  |  |  |  |
| Comprehends conversational speech and responds using complex sentences | 23135 | 20035 | 0.906(0.884-0.929,*P<*.001) | 1.174361123 | *P=*.05** |  |  |
| Self control ability | | | | | | | |
| Exhibits poor self-control and throws tantrums during parental discipline | 2023 | 4348 | 2.471(2.341-2.609,*P<*.001) | 1.305673304 | *P<*.001 | 0.08 | 1.540(1.411-1.681,*P<*.001) |
| Accepts and corrects behavior during parental discipline despite limited self-control | 38363 | 37685 | 1.311(1.274-1.350,*P<*.001) | 1.304251357 | *P<*.001 | 0.031 | 1.181(1.129-1.235,*P<*.001) |
| Demonstrates exceptional self-control requiring minimal parental supervision | 12353 | 6421 | 0.499 (95% CI: 0.483-0.516; *P<* .001) | . |  |  |  |
| Whether to start subject education | | | | | | | |
| No | 40866 | 35671 | refer |  |  |  |  |
| Yes | 13382 | 14107 | 1.208 (95% CI: 1.175-1.241; *P<* .001) | 1.118251948 | *P<*.001 | 0.015 | 1.070 (95% CI: 1.030-1.113; *P<* .001) |
| Who educates children the longest in the family at ordinary times | | | | | | | |
| Father | 7817 | 6538 | 0.908 (95% CI: 0.877-0.941; *P<* .001) | 4.877482415 | *P=*.50** |  |  |
| Mother | 46901 | 43181 | 1.080 (95% CI: 1.046-1.115; *P<* .001) | 4.906098688 | *P=*.13** |  |  |
| Father/Mother | 586 | 535 | 1.004 (95% CI: 0.893-1.130; *P =* .94)** |  |  |  |  |
| Grandparents (Paternal grandparents, Maternal grandparents) | 1126 | 1046 | 1.022 (95% CI: 0.939-1.113; *P =* .61)** |  |  |  |  |
| Mixed (parents + grandparents) | 229 | 203 | 0.975 (95% CI: 0.807-1.178; *P =* .79)** |  |  |  |  |
| The highest education of the child's father | | | | | | | |
| Below primary education | 172 | 82 | 0.524 (95% CI: 0.403-0.682; *P<* .001) | 1.608403386 | *P=*.87** |  |  |
| Primary education | 518 | 408 | 0.866 (95% CI: 0.760-0.986; *P =* .03)** |  |  |  |  |
| Middle school education | 10154 | 7300 | 0.757 (95% CI: 0.732-0.782; *P<* .001) | 20.50524045** |  |  |  |
| High school/Technical secondary | 13339 | 10931 | 0.875(0.851-0.901,*P<*.001) | 28.19934729** |  |  |  |
| Associate degree | 13775 | 13140 | 1.067(1.038-1.096,*P<*.001) | 31.90291333** |  |  |  |
| Bachelor's degree | 16375 | 17287 | 1.243(1.211-1.275,*P<*.001) | 37.69382996** |  |  |  |
| Graduate degree | 2482 | 2484 | 1.106(1.045-1.171,*P<*.001) | 9.250198936 | *P=*.08** |  |  |
| The highest education of the child's mother | | | | | | | |
| Below primary education | 236 | 102 | 0.475(0.376-0.599,*P<*.001) | 1.378835829 | *P<*.001 | 0** |  |
| Primary education | 883 | 611 | 0.759(0.684-0.842,*P<*.001) | 1.178025905 | *P<*.001 | 0** |  |
| Middle school education | 10893 | 7442 | 0.710(0.687-0.733,*P<*.001) | 2.293610452 | *P<*.001 | -0.043 | 0.816(0.776-0.859,*P<*.001) |
| High school/Technical secondary | 12812 | 10331 | 0.859(0.834-0.885,*P<*.001) | 1.917866133 | *P<*.001 | -0.014 | 0.921(0.883-0.962,*P<*.001) |
| Associate degree | 16207 | 16055 | 1.131(1.102-1.161,*P<*.001) | 1.640822259 | *P=*.01** |  |  |
| Bachelor's degree | 14236 | 15518 | 1.285(1.251-1.320,*P<*.001) | . |  |  |  |
| Graduate degree | 1548 | 1573 | 1.122(1.045-1.205,*P=*.002) | 1.191151375 | *P=*.49** |  |  |
| Per capita monthly income of families | | | | | | | |
| Monthly per capita income <1000 RMB | 1004 | 742 | 0.807(0.734-0.888,*P<*.001) | 1.029911897 | *P=*.57** |  |  |
| 1000 ≤ Monthly per capita income <2000 RMB | 2149 | 1696 | 0.860(0.806-0.918,*P<*.001) | 1.060576827 | *P=*.14** |  |  |
| 2000 ≤ Monthly per capita income <5000 RMB | 9659 | 8454 | 0.951(0.921-0.982,*P=*.002) | 1.179503388 | *P=*.07** |  |  |
| 5000 ≤ Monthly per capita income <10000 RMB | 18389 | 16957 | 1.107(0.991-1.044,*P=*.21)** |  |  |  |  |
| 10000 ≤ Monthly per capita income < 20000 yuan | 14394 | 13607 | 1.051(1.022-1.080,*P<*.001) | 1.145072829 | *P=*.57** |  |  |
| Monthly per capita income > 20000 yuan | 6696 | 6244 | 1.026(0.988-1.064,*P=*.18)** |  |  |  |  |
| (TZ1)Easy to lose spirit | | | | | | | |
| Never | 40923 | 26415 | 0.407(0.397-0.417,*P<*.001) | 4667000000000** |  |  |  |
| Seldom | 13825 | 19667 | 1.913(1.864-1.964,*P<*.001) | 1.298836233 | *P<*.001 | 0.03 | 1.142(1.097-1.189,*P<*.001) |
| Occasionally | 1936 | 5179 | 3.160(2.995-3.335,*P<*.001) | 1.288093984 | *P<*.001 | 0.049 | 1.289(1.189-1.397,*P<*.001) |
| Frequently | 96 | 314 | 3.615(2.875-4.545,*P<*.001) | 1.054051047 | *P=*.02** |  |  |
| Always | 35 | 57 | 1.793(1.177-2.732,*P=*.01) | 1.032195194 | *P=*.21** |  |  |
| (TZ2)Yellow complexion | | | | | | | |
| Never | 43231 | 26886 | 0.341(0.333-0.350,*P<*.001) | -250300000  0000** |  |  |  |
| Seldom | 9126 | 12851 | 1.732(1.680-1.784,*P<*.001) | 1.343615945 | *P<*.001 | 0.025 | 1.183(1.129-1.240,*P<*.001) |
| Occasionally | 3359 | 7634 | 2.761(2.646-2.881,*P<*.001) | 1.430157381 | *P<*.001 | 0.038 | 1.291(1.206-1.381,*P<*.001) |
| Frequently | 812 | 2987 | 4.235(3.915-4.581,*P<*.001) | 1.296788958 | *P<*.001 | 0.059 | 1.616(1.439-1.814,*P<*.001) |
| Always | 287 | 1274 | 4.983(4.381-5.667,*P<*.001) | 1.267201053 | *P<*.001 | 0.013 | 1.359(1.133-1.630,*P<*.001) |
| (TZ3)Soft muscles | | | | | | | |
| Never | 44217 | 29051 | 0.367(0.357-0.376,*P<*.001) | 1.885 | *P<*.001 | -0.042 | 0.890(0.834-0.951,*P<*.001) |
| Seldom | 8715 | 12359 | 1.737(1.685-1.791,*P<*.001) | 1.346625713 | *P<*.001 | -0.006 | 0.964(0.901-1.032,*P=*.30)** |
| Occasionally | 2787 | 6257 | 2.673(2.552-2.800,*P<*.001) | 1.402030223 | *P<*.001 | 0** |  |
| Frequently | 806 | 2742 | 3.897(3.600-4.220,*P<*.001) | 1.2958173 | *P=*.17** |  |  |
| Always | 290 | 1223 | 4.129(4.158-5.378,*P<*.001) | 1.271767212 | *P=*.17** |  |  |
| (TZ4)Always quiet | | | | | | | |
| Never | 37602 | 27954 | 0.603(0.589-0.618,*P<*.001) | 3909000000000** |  |  |  |
| Seldom | 12013 | 14438 | 1.448(1.408-1.489,*P<*.001) | 1.304842378 | *P=*.65** |  |  |
| Occasionally | 6593 | 8222 | 1.443(1.393-1.494,*P<*.001) | 1.324825493 | *P=*.22** |  |  |
| Frequently | 493 | 860 | 1.935(1.731-2.163,*P<*.001) | 1.119843814 | *P=*.39** |  |  |
| Always | 114 | 158 | 1.527(1.200-1.943,*P<*.001) | 1.092918449 | *P=*.08** |  |  |
| (TZ5)Speak in a low voice | | | | | | | |
| Never | 51291 | 41509 | 0.442(0.426-0.457,*P<*.001) | 7792000000000** |  |  |  |
| Seldom | 4117 | 7129 | 2.050(1.969-2.135,*P<*.001) | 1.410504806 | *P=*.07** |  |  |
| Occasionally | 1205 | 2440 | 2.289(2.134-2.455,*P<*.001) | 1.28562872 | *P=*.04** |  |  |
| Frequently | 151 | 423 | 3.100(2.573-3.734,*P<*.001) | 1.167848347 | *P=*.83** |  |  |
| Always | 51 | 131 | 2.831(2.048-3.913,*P<*.001) | 1.171224437 | *P=*.62** |  |  |
| (TZ6)Not talkative | | | | | | | |
| Never | 49054 | 39970 | 0.542(0.525-0.560,*P<*.001) | .** |  |  |  |
| Seldom | 5540 | 7771 | 1.640(1.581-1.701,*P<*.001) | 1.393098992 | *P=*.13** |  |  |
| Occasionally | 1906 | 3235 | 1.926(1.817-2.040,*P<*.001) | 1.309090159 | *P=*.53** |  |  |
| Frequently | 221 | 486 | 2.433(2.075-2.854,*P<*.001) | 1.169679796 | *P=*.16** |  |  |
| Always | 94 | 170 | 1.993(1.549-2.565,*P<*.001) | 1.162825579 | *P=*.93** |  |  |
| (TZ7)Little activity makes sweating easily | | | | | | | |
| Never | 16259 | 6777 | 0.377(0.365-0.389,*P<*.001) | 1.561942249 | *P<*.001 | -0.046 | 0.803(0.766-0.841,*P<*.001) |
| Seldom | 14931 | 12349 | 0.882(0.858-0.906,*P<*.001) | 1.534975976 | *P=*.13** |  |  |
| Occasionally | 13489 | 14038 | 1.199(1.167-1.233,*P<*.001) | 5891000000000** |  |  |  |
| Frequently | 9275 | 12911 | 1.709(1.659-1.761,*P<*.001) | 1.489884719 | *P<*.001 | 0.028 | 1.119(1.070-1.170,*P<*.001) |
| Always | 2861 | 5557 | 2.274(2.171-2.383,*P<*.001) | 1.262973292 | *P<*.001 | 0.067 | 1.403(1.309-1.505,*P<*.001) |
| (TZ8)Hands and feet always lukewarm | | | | | | | |
| Never | 47149 | 33442 | 0.377(0.366-0.388,*P<*.001) | 5365000000000** |  |  |  |
| Seldom | 7415 | 12516 | 2.132(2.165-2.200,*P<*.001) | 1.508617945 | *P=*.002** |  |  |
| Occasionally | 1878 | 4539 | 2.820(2.668-2.979,*P<*.001) | 1.354262899 | *P<*.001 | 0.006 | 1.044 (0.964-1.130, *P =* 0.29)** |
| Frequently | 286 | 855 | 3.328(2.910-3.807,*P<*.001) | 1.130899087 | *P=*.05** |  |  |
| Always | 87 | 280 | 3.555(2.794-4.524,*P<*.001) | 1.098306263 | *P=*.59** |  |  |
| (TZ9)Resistant to summer but not to winter | | | | | | | |
| Never | 47334 | 34507 | 0.404(0.392-0.415,*P<*.001) | .** |  |  |  |
| Seldom | 6847 | 11068 | 1.991(1.927-2.058,*P<*.001) | 1.617847857 | *P=*.97** |  |  |
| Occasionally | 2180 | 4709 | 2.515(2.387-2.650,*P<*.001) | 1.493594185 | *P=*.77** |  |  |
| Frequently | 344 | 1017 | 3.298(2.917-3.730,*P<*.001) | 1.250139115 | *P=*.14** |  |  |
| Always | 110 | 331 | 3.326(2.680-4.128,*P<*.001) | 1.241188662 | *P=*.49** |  |  |
| (TZ10)Afraid of cold than other children of the same age | | | | | | | |
| Never | 49945 | 39055 | 0.427(0.414-0.441,*P<*.001) | .** |  |  |  |
| Seldom | 5305 | 8932 | 2.031(1.959-2.106,*P<*.001) | 1.480434847 | *P=*.07** |  |  |
| Occasionally | 1292 | 2816 | 2.479(2.318-2.651,*P<*.001) | 1.301179815 | *P=*.60** |  |  |
| Frequently | 213 | 612 | 3.188(2.726-3.727,*P<*.001) | 1.171917825 | *P=*.90** |  |  |
| Always | 60 | 217 | 3.992(2.999-5.315,*P<*.001) | 1.208657218 | *P=*.71** |  |  |
| (TZ11)Feel uncomfortable or afraid of eating(drinking)cold food | | | | | | | |
| Never | 46184 | 34803 | 0.476(0.463-0.490,*P<*.001) | .** |  |  |  |
| Seldom | 7711 | 11085 | 1.741(1.686-1.797,*P<*.001) | 1.516068108 | *P=*.69** |  |  |
| Occasionally | 2321 | 4297 | 2.131(2.023-2.245,*P<*.001) | 1.445953139 | *P=*.06** |  |  |
| Frequently | 441 | 1035 | 2.615(2.338-2.925,*P<*.001) | 1.288995288 | *P=*.36** |  |  |
| Always | 158 | 412 | 2.884 (2.400-3.466, *P<* 0.001) | 1.359219887 | *P=*.22** |  |  |
| (TZ12)Having cold or eating(drinking)cold food makes it easy to have diarrhea | | | | | | | |
| Never | 42592 | 31259 | 0.512 (0.499-0.526, *P<* 0.001) | .** |  |  |  |
| Seldom | 11069 | 14780 | 1.658 (1.611-1.705, *P<* 0.001) | 1.574801614 | *P<*.001 | -0.009 | 0.904 (0.866-0.943, *P<* 0.001) |
| Occasionally | 2714 | 4582 | 1.941 (1.848-2.039, *P<* 0.001) | 1.508947856 | *P<*.001 | 0** |  |
| Frequently | 316 | 775 | 2.725 (2.389-3.107, *P<* 0.001) | 1.283658428 | *P=*.03** |  |  |
| Always | 124 | 236 | 2.099 (1.689-2.610, *P<* 0.001) | 1.340116612 | *P<*.001 | 0** |  |
| (TZ13)The body and face are always very hot | | | | | | | |
| Never | 44883 | 30268 | 0.377 (0.367-0.387, *P<* 0.001) | .** |  |  |  |
| Seldom | 8785 | 14360 | 2.106 (2.045-2.170, *P<* 0.001) | 1.543105799 | *P=*.21** |  |  |
| Occasionally | 2429 | 5361 | 2.594 (2.469-2.726, *P<* 0.001) | 1.346952903 | *P=*.03** |  |  |
| Frequently | 579 | 1315 | 2.538 (2.300-2.801, *P<* 0.001) | 1.106778567 | *P=*.60** |  |  |
| Always | 139 | 328 | 2.607 (2.137-3.180, *P<* 0.001) | 1.058467003 | *P=*.59** |  |  |
| (TZ14)Skin and lips dry easily | | | | | | | |
| Never | 43886 | 28296 | 0.357(0.348-0.367,*P<*.001) | -473600000  0000** |  |  |  |
| Seldom | 9964 | 15866 | 2.086(2.027-2.146,*P<*.001) | 1.514803499 | *P<*.001 | 0.022 | 1.109(1.060-1.161,*P<*.001) |
| Occasionally | 2456 | 5732 | 2.764(2.632-2.902,*P<*.001) | 1.356677507 | *P<*.001 | 0.026 | 1.137(1.056-1.224,*P<*.001) |
| Frequently | 405 | 1379 | 3.822(3.419-4.273,*P<*.001) | 1.118746359 | *P<*.001 | 0.007 | 1.274 (1.093-1.485, *P =* .002)** |
| Always | 104 | 359 | 3.818(3.068-4.751,*P<*.001) | 1.090199004 | *P=*.111** |  |  |
| (TZ15)Always excited | | | | | | | |
| Never | 26248 | 12853 | 0.386(0.376-0.396,*P<*.001) | .** |  |  |  |
| Seldom | 14798 | 14841 | 1.145(1.115-1.176,*P<*.001) | 1.719067637 | *P<*.001 | 0.003 | 1.087 (1.038-1.138, *P<* .001) |
| Occasionally | 12004 | 16779 | 1.797(1.749-1.847,*P<*.001) | 2.103025086 | *P<*.001 | 0.019 | 1.127 (1.073–1.185, *P<* .001) |
| Frequently | 3229 | 5997 | 2.181 (2.086–2.280, *P<* .001) | 1.740525547 | *P<*.001 | 0.035 | 1.259 (1.167–1.357, *P<* .001) |
| Always | 536 | 1162 | 2.417 (2.181–2.680, *P<* .001) | 1.396219081 | *P<*.001 | 0.019 | 1.464 (1.259–1.702, *P<* .001) |
| (TZ16)Easily restless | | | | | | | |
| Never | 33854 | 19991 | 0.429 (0.418–0.439, *P<* .001) | .** |  |  |  |
| Seldom | 14100 | 16286 | 1.396 (1.359–1.433, *P<* .001) | 1.597539461 | *P=*.11** |  |  |
| Occasionally | 6690 | 10553 | 1.925 (1.862–1.990, *P<* .001) | 1.695218656 | *P=*.02** |  |  |
| Frequently | 1827 | 3876 | 2.443 (2.308–2.586, *P<* .001) | 1.456714486 | *P<*.001 | 0.01 | 1.056 (0.968–1.153, *P =* .22)** |
| Always | 344 | 926 | 2.998 (2.647–3.395, *P<* .001) | 1.346506835 | *P=*.02** |  |  |
| (TZ17)Being overweight and unwilling to exercise | | | | | | | |
| Never | 49994 | 42120 | 0.604 (0.584–0.625, *P<* .001) | .** |  |  |  |
| Seldom | 5043 | 6704 | 1.532(1.474-1.592,*P<*.001) | 1.30416531 | *P<*.001 | -0.006 | 0.921(0.869-0.975,*P=*.01)** |
| Occasionally | 1462 | 2238 | 1.715(1.604-1.834,*P<*.001) | 1.2185666 | *P<*.001 | 0** |  |
| Frequently | 252 | 450 | 1.973(1.691-2.304,*P<*.001) | 1.103964825 | *P=*.01** |  |  |
| Always | 64 | 120 | 2.066(1.525-2.798,*P<*.001) | 1.122535356 | *P<*.001 | 0** |  |
| (TZ18)Abdominal obesity is flabby and soft | | | | | | | |
| Never | 45612 | 36106 | 0.571(0.555-0.587,*P<*.001) | .** |  |  |  |
| Seldom | 7498 | 9141 | 1.415(1.369-1.463,*P<*.001) | 1.256818953 | *P=*.02** |  |  |
| Occasionally | 2659 | 4249 | 1.826(1.737-1.920,*P<*.001) | 1.235053735 | *P<*.001 | 0** |  |
| Frequently | 835 | 1561 | 2.090(1.920-2.275,*P<*.001) | 1.166444047 | *P<*.001 | 0** |  |
| Always | 211 | 575 | 3.021(2.579-3.539,*P<*.001) | 1.166141087 | *P<*.001 | 0** |  |
| (TZ19)Always sleepy | | | | | | | |
| Never | 51130 | 40550 | 0.407(0.393-0.421,*P<*.001) | .** |  |  |  |
| Seldom | 4813 | 8879 | 2.244(2.162-2.329,*P<*.001) | 1.394480608 | *P<*.001 | 0** |  |
| Occasionally | 794 | 1946 | 2.763(2.542-3.004,*P<*.001) | 1.177193328 | *P<*.001 | 0** |  |
| Frequently | 58 | 203 | 3.863(2.884-5.173,*P<*.001) | 1.035413696 | *P=*.31** |  |  |
| Always | 20 | 54 | 2.973(1.780-4.967,*P<*.001) | 1.057809969 | *P=*.31** |  |  |
| (TZ20)Like to eat sweet or greasy things | | | | | | | |
| Never | 27782 | 15315 | 0.441(0.430-0.452,*P<*.001) | .** |  |  |  |
| Seldom | 16903 | 16401 | 1.099(1.071-1.128,*P<*.001) | 1.477886219 | *P<*.001 | 0.003 | 1.041(0.998-1.086,*P=*.06)** |
| Occasionally | 9344 | 12955 | 1.702(1.652-1.753,*P<*.001) | 1.564393779 | *P<*.001 | 0.001 | 1.023(0.974-1.075,*P=*.35)** |
| Frequently | 2312 | 5439 | 2.776(2.640-2.919,*P<*.001) | 1.355237648 | *P<*.001 | 0.044 | 1.228(1.138-1.325,*P<*.001) |
| Always | 474 | 1522 | 3.610(3.254-4.005,*P<*.001) | 1.208641744 | *P<*.001 | 0** |  |
| (TZ21)Snoring at night | | | | | | | |
| Never | 41501 | 28880 | 0.468(0.457-0.480,*P<*.001) | .** |  |  |  |
| Seldom | 11682 | 15484 | 1.655(1.610-1.701,*P<*.001) | 1.248193959 | *P<*.001 | 0.021 | 1.098(1.053-1.145,*P<*.001) |
| Occasionally | 3046 | 5743 | 2.209(2.111-2.312,*P<*.001) | 1.228896402 | *P<*.001 | 0.032 | 1.141(1.066-1.222,*P<*.001) |
| Frequently | 474 | 1165 | 2.744(2.464-3.055,*P<*.001) | 1.07159933 | *P<*.001 | 0** |  |
| Always | 112 | 360 | 3.555(2.875-4.396,*P<*.001) | 1.082424855 | *P=*.19** |  |  |
| (TZ22)Thick and greasy tongue coating | | | | | | | |
| Never | 41478 | 24101 | 0.324(0.316-0.332,*P<*.001) | .** |  |  |  |
| Seldom | 12445 | 18884 | 2.056(2.002-2.112,*P<*.001) | 1.47076567 | *P<*.001 | 0.063 | 1.316(1.261-1.374,*P<*.001) |
| Occasionally | 2576 | 7138 | 3.378(3.224-3.540,*P<*.001) | 1.46854109 | *P<*.001 | 0.1 | 1.578(1.467-1.696,*P<*.001) |
| Frequently | 276 | 1212 | 4.924 (4.318–5.615, *P<* 0.001) | 1.133033849 | *P<*.001 | 0.074 | 2.059 (1.714–2.474, *P<* 0.001) |
| Always | 40 | 297 | 8.212 (5.902–11.426, *P<* 0.001) | 1.123793894 | *P<*.001 | 0** |  |
| (TZ23)Always red eyes,or easy to get gum | | | | | | | |
| Never | 42812 | 27017 | 0.359 (0.350–0.368, *P<* 0.001) | -4045000000  000* |  |  |  |
| Seldom | 11347 | 17349 | 2.028 (1.973–2.084, *P<* 0.001) | 1.457274308 | *P<*.001 | 0.03 | 1.153 (1.103–1.215, *P<* 0.001) |
| Occasionally | 2366 | 5998 | 3.025 (2.880–3.177, *P<* 0.001) | 1.390754326 | *P<*.001 | 0.037 | 1.227 (1.138–1.324, *P<* 0.001) |
| Frequently | 245 | 1065 | 4.863 (4.230–5.590, *P<* 0.001) | 1.122434296 | *P<*.001 | 0.001 | 1.572 (1.293–1.912, *P<* 0.001) |
| Always | 45 | 203 | 4.980 (3.605–6.879, *P<* 0.001) | 1.095603446 | *P=*.12** |  |  |
| (TZ24)Eczema is easy to grow on the body | | | | | | | |
| Never | 43612 | 31122 | 0.459 (0.447–0.472, *P<* 0.001) | .** |  |  |  |
| Seldom | 9119 | 12792 | 1.723(1.672-1.775,*P<*.001) | 1.273440458 | *P=*.04** |  |  |
| Occasionally | 3046 | 5333 | 2.033(1.941-2.129,*P<*.001) | 1.171773992 | *P<*.001 | 0.003 | 1.029(0.962-1.100,*P=*.41)** |
| Frequently | 796 | 1728 | 2.437(2.239-2.653,*P<*.001) | 1.07328838 | *P<*.001 | 0** |  |
| Always | 242 | 657 | 3.013(2.599-3.493,*P<*.001) | 1.063617203 | *P<*.001 | 0** |  |
| (TZ25)Like to eat spicy or fried barbecue food | | | | | | | |
| Never | 39145 | 28252 | 0.545(0.532-0.559,*P<*.001) | .** |  |  |  |
| Seldom | 12528 | 14248 | 1.347(1.311-1.385,*P<*.001) | 1.203051222 | *P=*.15** |  |  |
| Occasionally | 4361 | 6980 | 1.880(1.807-1.957,*P<*.001) | 1.190178612 | *P=*.01** |  |  |
| Frequently | 643 | 1673 | 2.925(2.669-3.207,*P<*.001) | 1.089929933 | *P<*.001 | 0** |  |
| Always | 138 | 479 | 3.846(3.181-4.649,*P<*.001) | 1.100983149 | *P=*.03** |  |  |
| (TZ26)Sweat is sticky | | | | | | | |
| Never | 42371 | 27316 | 0.383(0.373-0.393,*P<*.001) | .** |  |  |  |
| Seldom | 11610 | 16932 | 1.900(1.848-1.953,*P<*.001) | 1.341798555 | *P<*.001 | 0.023 | 1.083(1.037-1.131,*P<*.001) |
| Occasionally | 2425 | 5962 | 2.928(2.788-3.074,*P<*.001) | 1.29236379 | *P<*.001 | 0.041 | 1.245(1.155-1.342,*P<*.001) |
| Frequently | 328 | 1127 | 3.843(3.396-4.348,*P<*.001) | 1.081465071 | *P<*.001 | 0** |  |
| Always | 81 | 295 | 4.025(3.147-5.148,*P<*.001) | 1.072786731 | *P=*.02** |  |  |
| (TZ27)The skin always appears blue and purple ecchymosis unconsciously | | | | | | | |
| Never | 49200 | 37258 | 0.401(0.389-0.414,*P<*.001) | .** |  |  |  |
| Seldom | 5995 | 10217 | 2.091(2.021-2.165,*P<*.001) | 1.350246149 | *P<*.001 | 0.007 | 1.040(0.987-1.095,*P=*.14)** |
| Occasionally | 1354 | 3280 | 2.779(2.605-2.964,*P<*.001) | 1.234522401 | *P=*.003** |  |  |
| Frequently | 231 | 701 | 3.371(2.904-3.914,*P<*.001) | 1.078072264 | *P=*.28** |  |  |
| Always | 35 | 176 | 5.549(3.860-7.977,*P<*.001) | 1.113437162 | *P=*.24** |  |  |
| (TZ28)Where there is pain on the body | | | | | | | |
| Never | 50732 | 39790 | 0.403(0.390-0.417,*P<*.001) | .** |  |  |  |
| Seldom | 5103 | 9207 | 2.199(2.120-2.281,*P<*.001) | 1.413954235 | *P=*.95** |  |  |
| Occasionally | 884 | 2316 | 2.971(2.747-3.214,*P<*.001) | 1.244295464 | *P=*.56** |  |  |
| Frequently | 77 | 259 | 3.715(2.880-4.792,*P<*.001) | 1.06128319 | *P=*.58** |  |  |
| Always | 19 | 60 | 3.478(2.076-5.827,*P<*.001) | 1.076120699 | *P=*.75** |  |  |
| (TZ29)The skin on the body or the skin on the neck,armpit or thigh root is dark | | | | | | | |
| Never | 50051 | 39088 | 0.421(0.408-0.435,*P<*.001) | .** |  |  |  |
| Seldom | 5225 | 8702 | 2.001(1.929-2.076,*P<*.001) | 1.353827984 | *P=*.22** |  |  |
| Occasionally | 1144 | 2563 | 2.542(2.368-2.728,*P<*.001) | 1.198691324 | *P=*.21** |  |  |
| Frequently | 283 | 898 | 3.536(3.092-4.043,*P<*.001) | 1.083869431 | *P=*.07** |  |  |
| Always | 112 | 381 | 3.764(3.048-4.648,*P<*.001) | 1.09226808 | *P=*.31** |  |  |
| (TZ30)Be prone to dark circles | | | | | | | |
| Never | 43229 | 29679 | 0.425(0.414-0.436,*P<*.001) | .** |  |  |  |
| Seldom | 10078 | 13960 | 1.719(1.669-1.769,*P<*.001) | 1.256956216 | *P<*.001 | 0.011 | 1.056(1.010-1.103,*P=*.02)** |
| Occasionally | 2630 | 5300 | 2.357(2.246-2.474,*P<*.001) | 1.210864753 | *P<*.001 | 0.001 | 1.048(0.976-1.126,*P=*.20)** |
| Frequently | 672 | 1981 | 3.333(3.052-3.641,*P<*.001) | 1.097681576 | *P<*.001 | 0** |  |
| Always | 206 | 712 | 3.842(3.289-4.489,*P<*.001) | 1.091819665 | *P=*.01** |  |  |
| (TZ31)Blue or dark gums | | | | | | | |
| Never | 49844 | 39527 | 0.457(0.442-0.472,*P<*.001) | . |  |  |  |
| Seldom | 5346 | 8718 | 1.956(1.886-2.208,*P<*.001) | 1.321134462 | *P<*.001 | 0.004 | 1.038(0.981-1.097,*P=*.19)** |
| Occasionally | 1299 | 2509 | 2.183(2.039-2.337,*P<*.001) | 1.17137055 | *P<*.001 | 0** |  |
| Frequently | 262 | 614 | 2.598(2.247-3.003,*P<*.001) | 1.046005177 | *P<*.001 | 0** |  |
| Always | 64 | 264 | 4.557(3.467-5.990,*P<*.001) | 1.04631749 | *P<*.001 | 0** |  |
| (TZ32)Rough skin,like fish scales | | | | | | | |
| Never | 53723 | 45510 | 0.428(0.409-0.448,*P<*.001) | .** |  |  |  |
| Seldom | 2367 | 4471 | 2.181(2.072-2.296,*P<*.001) | 1.391139465 | *P=*.70** |  |  |
| Occasionally | 560 | 1165 | 2.319(2.095-2.567,*P<*.001) | 1.201397039 | *P=*.006** |  |  |
| Frequently | 124 | 346 | 3.084(2.511-3.788,*P<*.001) | 1.062098466 | *P=*.40** |  |  |
| Always | 41 | 140 | 3.765(2.658-5.333,*P<*.001) | 1.069354854 | *P=*.20** |  |  |
| (TZ33)Lips are dark or blue | | | | | | | |
| Never | 53949 | 44646 | 0.340(0.324-0.355,*P<*.001) | . |  |  |  |
| Seldom | 2407 | 5435 | 2.659(2.531-2.794,*P<*.001) | 1.514279736 | *P<*.001 | 0** |  |
| Occasionally | 375 | 1235 | 3.688(3.284-4.143,*P<*.001) | 1.277747905 | *P<*.001 | 0** |  |
| Frequently | 61 | 226 | 4.090(3.082-5.429,*P<*.001) | 1.064477431 | *P=*.006** |  |  |
| Always | 23 | 90 | 4.312(2.727-6.817,*P<*.001) | 1.078110071 | *P=*.08** |  |  |
| (TZ34)Always moping | | | | | | | |
| Never | 50372 | 39219 | 0.404(0.391-0.418,*P<*.001) | .** |  |  |  |
| Seldom | 5683 | 10179 | 2.209(2.133-2.288,*P<*.001) | 1.575929209 | *P=*.20** |  |  |
| Occasionally | 714 | 2053 | 3.254(2.985-3.546,*P<*.001) | 1.364635066 | *P=*.96** |  |  |
| Frequently | 33 | 151 | 5.047(3.462-7.357,*P<*.001) | 1.102153706 | *P=*.97** |  |  |
| Always | 13 | 30 | 2.540(1.325-4.870,*P=*.005) | 1.132700412 | *P=*.11** |  |  |
| (TZ35)Easily nervous | | | | | | | |
| Never | 46417 | 32758 | 0.389(0.378-0.400,*P<*.001) | .** |  |  |  |
| Seldom | 8535 | 13667 | 2.036(1.976-2.099,*P<*.001) | 1.806567398 | *P=*.23** |  |  |
| Occasionally | 1690 | 4468 | 3.090(2.918-3.272,*P<*.001) | 1.762439067 | *P=*.05** |  |  |
| Frequently | 145 | 623 | 4.773(3.983-5.721,*P<*.001) | 1.349276159 | *P=*.75** |  |  |
| Always | 28 | 116 | 4.567(3.022-6.901,*P<*.001) | 1.349081347 | *P=*.23** |  |  |
| (TZ36)Easy to lack self confidence | | | | | | | |
| Never | 36512 | 21822 | 0.407(0.397-0.417,*P<*.001) | .** |  |  |  |
| Seldom | 15175 | 18715 | 1.560(1.520-1.601,*P<*.001) | 1.936038572 | *P<*.001 | 0.007 | 1.046(1.000-1.094,*P=*.05)** |
| Occasionally | 4500 | 8876 | 2.413(2.323-2.507,*P<*.001) | 2.185121482 | *P<*.001 | 0.006 | 1.045(0.975-1.121,*P=*.22)** |
| Frequently | 519 | 1825 | 3.975(3.603-4.385,*P<*.001) | 1.6577046 | *P<*.001 | 0.02 | 1.332(1.144-1.550,*P<*.001) |
| Always | 109 | 394 | 4.000(3.235-4.947,*P<*.001) | 1.54751564 | *P=*.14** |  |  |
| (TZ37)Easily afraid or frightened | | | | | | | |
| Never | 37605 | 21896 | 0.376(0.367-0.386,*P<*.001) | .** |  |  |  |
| Seldom | 14360 | 18805 | 1.694(1.650-1.738,*P<*.001) | 1.922486513 | *P<*.001 | 0.006 | 1.033(0.987-1.081,*P=*.17)** |
| Occasionally | 4219 | 8723 | 2.534(2.438-2.635,*P<*.001) | 2.053840394 | *P<*.001 | 0.003 | 1.017(0.949-1.089,*P=*.64)** |
| Frequently | 527 | 1783 | 3.820(3.464-4.213,*P<*.001) | 1.50188754 | *P<*.001 | 0** |  |
| Always | 104 | 425 | 4.526(3.651-5.610,*P<*.001) | 1.434983903 | *P=*.002** |  |  |
| (TZ38)Always crying when in trouble | | | | | | | |
| Never | 28137 | 14583 | 0.401(0.391-0.411,*P<*.001) | .** |  |  |  |
| Seldom | 19254 | 19735 | 1.207(1.177-1.237,*P<*.001) | 1.738889767 | *P<*.001 | 0.001 | 1.012 (0.974–1.051, *P =* .55)** |
| Occasionally | 7423 | 11861 | 1.984 (1.922–2.049, *P<* .001) | 1.884509887 | *P<*.001 | 0** |  |
| Frequently | 1639 | 4227 | 3.002 (2.832–3.182, *P<* .001) | 1.506670936 | *P<*.001 | 0.03 | 1.176 (1.076–1.285, *P<* .001) |
| Always | 362 | 1226 | 3.793 (3.371–4.267, *P<* .001) | 1.335127085 | *P<*.001 | 0** |  |
| (TZ39)Easily shy or timid | | | | | | | |
| Never | 28530 | 15664 | 0.432 (0.421–0.443, *P<* .001) | .** |  |  |  |
| Seldom | 20271 | 20941 | 1.230 (1.200–1.261, *P<* .001) | 1.84040919 | *P<*.001 | 0** |  |
| Occasionally | 6784 | 11507 | 2.115 (2.047–2.185, *P<* .001) | 2.212104395 | *P<*.001 | 0.009 | 0.984 (0.932–1.039, *P =* .56)** |
| Frequently | 1045 | 2804 | 3.065 (2.852–3.294, *P<* .001) | 1.669773766 | *P<*.001 | 0.009 | 0.999 (0.890–1.122, *P =* .99)** |
| Always | 185 | 716 | 4.305(3.660-5.062,*P<*.001) | 1.569095917 | *P<*.001 | 0** |  |
| (TZ40)Always worried about a lot of things | | | | | | | |
| Never | 41624 | 27419 | 0.413(0.403-0.424,*P<*.001) | .** |  |  |  |
| Seldom | 11791 | 16231 | 1.751(1.703-1.800,*P<*.001) | 1.560517306 | *P=*.08** |  |  |
| Occasionally | 2926 | 6250 | 2.536(2.423-2.655,*P<*.001) | 1.612173332 | *P=*.42** |  |  |
| Frequently | 402 | 1353 | 3.776(3.376-4.224,*P<*.001) | 1.324167164 | *P=*.14** |  |  |
| Always | 72 | 379 | 5.828(4.528-7.500,*P<*.001) | 1.320119331 | *P=*.01** |  |  |
| (TZ41)More attached to you now than before | | | | | | | |
| Never | 33980 | 20326 | 0.436(0.426-0.447,*P<*.001) | .** |  |  |  |
| Seldom | 14937 | 17481 | 1.435(1.398-1.473,*P<*.001) | 1.357 | *P<*.001 | 0** |  |
| Occasionally | 6341 | 10293 | 1.982(1.916-2.050,*P<*.001) | 1.350596792 | *P<*.001 | 0.001 | 0.981(0.932-1.032,*P=*.45)** |
| Frequently | 1234 | 2801 | 2.584(2.414-2.766,*P<*.001) | 1.115534267 | *P<*.001 | 0.007 | 1.130(1.023-1.248,*P=*.02)** |
| Always | 323 | 731 | 2.512(2.202-2.865, *P<*0.001) | 1.056153315 | *P=*.001** |  |  |
| (TZ42)Sneez when don't have a cold | | | | | | | |
| Never | 37887 | 25833 | 0.500(0.488-0.513, *P<*0.001) | 2963000000000** |  |  |  |
| Seldom | 14874 | 18412 | 1.563(1.523-1.604, *P<*0.001) | 1.496205785 | *P=*.006** |  |  |
| Occasionally | 3469 | 5827 | 1.956(1.872-2.044, *P<*0.001) | 1.594217321 | *P=*.10** |  |  |
| Frequently | 472 | 1177 | 2.785(2.501-3.101, *P<*0.001) | 1.417172414 | *P=*.13** |  |  |
| Always | 113 | 383 | 3.750(3.039-4.627, *P<*0.001) | 1.57299202 | *P=*.009** |  |  |
| (TZ43)Without cold but have stuffy nose and runny nose | | | | | | | |
| Never | 43974 | 31865 | 0.471(0.458-0.483, *P<*0.001) | .** |  |  |  |
| Seldom | 9256 | 12637 | 1.665(1.616-1.716, *P<*0.001) | 1.470686505 | *P<*.001 | 0.002 | 1.081(1.009-1.158, *P=*0.03)** |
| Occasionally | 3001 | 5450 | 2.116(2.021-2.216, *P<*0.001) | 1.555242808 | *P<*.001 | 0.002 | 1.160(0.996-1.350, *P=*0.06)** |
| Frequently | 452 | 1308 | 3.241(2.910-3.609, *P<*0.001) | 1.434993294 | *P<*.001 | 0** |  |
| Always | 132 | 372 | 3.116(2.554-3.802,*P<*.001) | 1.572252889 | *P=*.03** |  |  |
| (TZ44)Cough and wheeze when the season changes,the temperature changes or smells strange smell | | | | | | | |
| Never | 49103 | 38143 | 0.444(0.431-0.458,*P<*.001) | 5510000000000** |  |  |  |
| Seldom | 6182 | 9741 | 1.905(1.840-1.971,*P<*.001) | 1.45651366 | *P=*.05** |  |  |
| Occasionally | 1302 | 3004 | 2.634(2.465-2.814,*P<*.001) | 1.36155614 | *P<*.001 | 0** |  |
| Frequently | 184 | 594 | 3.582(3.035-4.228,*P<*.001) | 1.186644154 | *P=*.005** |  |  |
| Always | 44 | 150 | 3.759(2.686-5.262,*P<*.001) | 1.202229434 | *P=*.13** |  |  |
| (TZ45)Susceptible to allergies (to drugs,food,smells,pollen or changing seasons and climate) | | | | | | | |
| Never | 48053 | 37219 | 0.471(0.457-0.485,*P<*.001) | -397400000  0000 |  |  |  |
| Seldom | 6040 | 9243 | 1.833(1.770-1.898,*P<*.001) | 1.454474308 | *P<*.001 | 0.001 | 1.019(0.966-1.075,*P=*.483)** |
| Occasionally | 2035 | 3587 | 2.010(1.901-2.125,*P<*.001) | 1.366420574 | *P=*.57** |  |  |
| Frequently | 516 | 1114 | 2.406(2.166-2.672,*P<*.001) | 1.269234236 | *P=*.93** |  |  |
| Always | 171 | 469 | 3.037(2.548-3.619,*P<*.001) | 1.300630134 | *P=*.095** |  |  |
| (TZ46)The skin is prone to itching and urticaria | | | | | | | |
| Never | 45437 | 33322 | 0.456(0.443-0.468,*P<*.001) | 4187000000000** |  |  |  |
| Seldom | 7921 | 11290 | 1.727(1.674-1.783,*P<*.001) | 1.370358718 | *P<*.001 | 0** |  |
| Occasionally | 2547 | 4804 | 2.186(2.080-2.297,*P<*.001) | 1.334188585 | *P<*.001 | 0** |  |
| Frequently | 692 | 1604 | 2.600(2.377-2.845,*P<*.001) | 1.238157779 | *P<*.001 | 0** |  |
| Always | 218 | 612 | 3.114(2.667-3.637,*P<*.001) | 1.268897023 | *P<*.001 | 0** |  |
| (TZ47)Purpura has occurred in the skin due to allergies | | | | | | | |
| Never | 54112 | 46411 | 0.444(0.423-0.466,*P<*.001) | 2476000000000** |  |  |  |
| Seldom | 2254 | 4193 | 2.140(2.030-2.255,*P<*.001) | 1.350701147 | *P<*.001 | 0** |  |
| Occasionally | 389 | 854 | 2.440(2.163-2.752,*P<*.001) | 1.191195632 | *P<*.001 | 0** |  |
| Frequently | 39 | 120 | 3.391(2.363-4.868,*P<*.001) | 1.050395171 | *P=*.51** |  |  |
| Always | 21 | 54 | 2.831(1.710-4.688,*P<*.001) | 1.084391584 | *P=*.40** |  |  |
| (TZ48)The skin turns red when scratched,and scratches appear | | | | | | | |
| Never | 45044 | 32996 | 0.463(0.450-0.475,*P<*.001) | .** |  |  |  |
| Seldom | 9067 | 13014 | 1.775(1.722-1.829,*P<*.001) | 1.348197621 | *P<*.001 | 0.005 | 1.054 (1.008–1.103, *P =* .022)** |
| Occasionally | 2041 | 4064 | 2.293 (2.171–2.421, *P<* .001) | 1.286773072 | *P<*.001 | 0** |  |
| Frequently | 460 | 1138 | 2.761 (2.476–3.079, *P<* .001) | 1.163327021 | *P<*.001 | 0** |  |
| Always | 203 | 420 | 2.287 (1.933–2.705, *P<* .001) | 1.19043824 | *P=*.66** |  |  |
| (TZ49)Always lively and strong | | | | | | | |
| Never | 17039 | 8139 | 0.437 (0.424–0.450, *P<* .001) | .** |  |  |  |
| Seldom | 6751 | 7263 | 1.214 (1.172–1.258, *P<* .001) | 1.954550524 | *P=*.004** |  |  |
| Occasionally | 9232 | 11626 | 1.498 (1.453–1.544, *P<* .001) | 2.412572298 | *P<*.001 | 0.002 | 1.006 (0.963–1.051, *P =* .78)** |
| Frequently | 15337 | 17055 | 1.334 (1.300–1.369, *P<* .001) | 3.111244163 | *P<*.001 | 0** |  |
| Always | 8456 | 7549 | 0.979 (0.947–1.013, *P =* .22)** |  |  |  |  |
| (TZ50)Can adapt to weather changes and new environment | | | | | | | |
| Never | 18308 | 8935 | 0.440 (0.428–0.453, *P<* .001) | 2.62077282 | *P<*.001 | 0.001 | 1.005(0.962-1.050,*P=*.83)** |
| Seldom | 3964 | 4984 | 1.425(1.364-1.488,*P<*.001) | 1.45770078 | *P=*.005** |  |  |
| Occasionally | 6147 | 8803 | 1.694(1.636-1.755,*P<*.001) | 1.395917058 | *P=*.11** |  |  |
| Frequently | 15273 | 17259 | 1.366(1.331-1.402,*P<*.001) | 2892000000000** |  |  |  |
| Always | 13123 | 11651 | 0.970(0.943-0.998,*P=*.04)** |  |  |  |  |
| (TZ51)Easy to have nightmares or wake up from dreams | | | | | | | |
| Never | 42608 | 28530 | 0.412(0.401-0.423,*P<*.001) | 3762000000000** |  |  |  |
| Seldom | 11733 | 17410 | 1.955(1.902-2.009,*P<*.001) | 1.283090699 | *P<*.001 | 0.03 | 1.141(1.094-1.190,*P<*.001) |
| Occasionally | 2218 | 4874 | 2.566(2.437-2.702,*P<*.001) | 1.181515355 | *P<*.001 | 0.022 | 1.168(1.082-1.261,*P<*.001) |
| Frequently | 193 | 629 | 3.618(3.078-4.253,*P<*.001) | 1.034110647 | *P<*.001 | 0** |  |
| Always | 63 | 189 | 3.310(2.488-4.403,*P<*.001) | 1.030660969 | *P<*.001 | 0** |  |
| (YSRT1)Eat less than your peers | | | | | | | |
| Never | 25275 | 13524 | 0.443(0.432-0.454,*P<*.001) | 1.886216711 | *P<*.001 | 0.012 | 0.964(0.926-1.004,*P=*.08)** |
| Occasionally | 26688 | 26179 | 1.161(1.134-1.189,*P<*.001) | .** |  |  |  |
| Frequently | 3943 | 8772 | 2.744(2.638-2.855,*P<*.001) | 1.49849829 | *P<*.001 | 0.055 | 1.316(1.236-1.400,*P<*.001) |
| Always | 909 | 3157 | 4.005(3.717-4.316,*P<*.001) | 1.868829617 | *P<*.001 | 0.061 | 1.472(1.301-1.665,*P<*.001) |
| (YSRT2)Eating slowly compared with peers | | | | | | | |
| Never | 21072 | 11041 | 0.461(0.449-0.474,*P<*.001) | 3845000000000** |  |  |  |
| Occasionally | 26139 | 22964 | 0.940(0.918-0.963,*P<*.001) | 2.238615758 | *P=*.63** |  |  |
| Frequently | 7820 | 12849 | 2.076(2.012-2.141,*P<*.001) | 2.579034431 | *P<*.001 | 0.019 | 1.073(1.019-1.129,*P=*.007)** |
| Always | 1784 | 4778 | 3.146(2.975-3.326,*P<*.001) | 2.385909383 | *P<*.001 | 0.017 | 1.023(0.925-1.131,*P=*.66)** |
| (YSRT3)Not interested in food | | | | | | | |
| Never | 31628 | 18120 | 0.431 (0.420-0.441, *P<* .001) | 6291000000000** |  |  |  |
| Occasionally | 22142 | 25301 | 1.505(1.469-1.541,*P<*.001) | 1.920441025 | *P=*.10** |  |  |
| Frequently | 2543 | 6329 | 2.982(2.843-3.127,*P<*.001) | 1.932303557 | *P=*.007** |  |  |
| Always | 502 | 1882 | 4.244(3.843-4.686,*P<*.001) | 1.831214511 | *P=*.06** |  |  |
| (YSRT4)Refuse certain foods for more than a month | | | | | | | |
| Never | 41221 | 27751 | 0.440(0.429-0.451,*P<*.001) | .** |  |  |  |
| Occasionally | 11359 | 14560 | 1.572(1.528-1.616,*P<*.001) | 1.431875954 | *P=*.002** |  |  |
| Frequently | 2834 | 5804 | 2.412(2.302-2.527,*P<*.001) | 1.538085929 | *P<*.001 | 0.009 | 1.048(0.975-1.125,*P=*.20)** |
| Always | 1401 | 3517 | 2.891(2.714-3.080,*P<*.001) | 1.672192162 | *P<*.001 | 0.018 | 1.104(0.997-1.223,*P=*.06)** |
| (YSRT5)Unwilling to try new food | | | | | | | |
| Never | 31274 | 18802 | 0.468(0.456-0.479,*P<*.001) | .** |  |  |  |
| Occasionally | 21017 | 23283 | 1.399(1.365-1.433,*P<*.001) | 1.619121529 | *P<*.001 | 0** |  |
| Frequently | 3499 | 6804 | 2.313(2.216-2.414,*P<*.001) | 1.694664438 | *P<*.001 | 0.009 | 1.077(1.009-1.150,*P=*.03)** |
| Always | 1025 | 2743 | 3.054(2.840-3.284,*P<*.001) | 1.68236078 | *P<*.001 | 0.004 | 1.161(1.035-1.303,*P=*.01)** |
| (YSRT6)Have a strong preference for certain textures or certain types of food | | | | | | | |
| Never | 27263 | 14200 | 0.411(0.401-0.422,*P<*.001) | .** |  |  |  |
| Occasionally | 21825 | 22541 | 1.242(1.212-1.273,*P<*.001) | 1.741275103 | *P<*.001 | 0** |  |
| Frequently | 6033 | 10957 | 2.267(2.192-2.346,*P<*.001) | 1.879176818 | *P<*.001 | 0.006 | 1.023(0.971-1.077,*P=*.40)** |
| Always | 1694 | 3934 | 2.684(2.532-2.845,*P<*.001) | 1.695278474 | *P<*.001 | 0** |  |
| (YSRT7)Distraction from eating(watching TV,playing video games,etc.) | | | | | | | |
| Never | 21021 | 11598 | 0.493(0.480-0.507,*P<*.001) | 1.561113636 | *P=*.004** |  |  |
| Occasionally | 26414 | 23422 | 0.956(0.933-0.979,*P<*.001) | .** |  |  |  |
| Frequently | 7512 | 11906 | 1.967(1.906-2.030,*P<*.001) | 1.40294084 | *P<*.001 | 0.017 | 1.090(1.038-1.145,*P<*.001) |
| Always | 1868 | 4706 | 2.950(2.792-3.117,*P<*.001) | 1.639841901 | *P<*.001 | 0.038 | 1.153(1.056-1.258,*P=*.001)** |
| (YSRT8)The dining place is not fixed | | | | | | | |
| Never | 33947 | 22992 | 0.541(0.528-0.554,*P<*.001) | .** |  |  |  |
| Occasionally | 20509 | 23671 | 1.499(1.463-1.536,*P<*.001) | 1.466261945 | *P<*.001 | 0.004 | 1.026(0.989-1.065,*P=*.16)** |
| Frequently | 1913 | 3808 | 2.285(2.160-2.417,*P<*.001) | 1.327781704 | *P=*.002** |  |  |
| Always | 446 | 1161 | 2.907(2.605-3.245,*P<*.001) | 1.311287218 | *P=*.79** |  |  |
| (YSFY1)Few emotional exchanges during meals | | | | | | | |
| Never | 30112 | 20495 | 0.584(0.570-0.598,*P<*.001) | .** |  |  |  |
| Occasionally | 22424 | 25762 | 1.527(1.491-1.565,*P<*.001) | 1.397065347 | *P<*.001 | 0.005 | 1.007(0.971-1.044,*P=*.72)** |
| Frequently | 3747 | 4620 | 1.392(1.331-1.456,*P<*.001) | 1.192296961 | *P=*.09** |  |  |
| Always | 532 | 755 | 1.570(1.404-1.755,*P<*.001) | 1.095817231 | *P=*.75** |  |  |
| (YSFY2)Force or punish children to eat more | | | | | | | |
| Never | 41607 | 27992 | 0.433(0.422-0.444,*P<*.001) | .** |  |  |  |
| Occasionally | 13876 | 19772 | 1.920(1.871-1.971,*P<*.001) | 1.362750409 | *P<*.001 | 0.025 | 1.103(1.059-1.149,*P<*.001) |
| Frequently | 1159 | 3235 | 3.210(2.998-3.436,*P<*.001) | 1.296431443 | *P<*.001 | 0.03 | 1.158(1.046-1.282,*P=*.005)** |
| Always | 173 | 633 | 4.064(3.433-4.810,*P<*.001) | 1.272105939 | *P<*.001 | 0** |  |
| (YSFY3)Induce children to eat(toys,TV,story rewards,etc.) | | | | | | | |
| Never | 34661 | 22315 | 0.487(0.475-0.498,*P<*.001) | .** |  |  |  |
| Occasionally | 19380 | 23169 | 1.572(1.534-1.611,*P<*.001) | 1.53677325 | *P<*.001 | 0.003 | 1.038(0.998-1.080,*P=*.06)** |
| Frequently | 2383 | 5074 | 2.489(2.367-2.618,*P<*.001) | 1.493021148 | *P<*.001 | 0.009 | 1.069(0.987-1.158,*P=*.10)** |
| Always | 391 | 1074 | 3.066(2.729-3.444,*P<*.001) | 1.400897168 | *P=*.11** |  |  |
| (YSFY4)Allow children to choose food at will | | | | | | | |
| Never | 24478 | 13684 | 0.476(0.464-0.489,*P<*.001) | 1.581993842 | *P<*.001 | 0** |  |
| Occasionally | 24361 | 26303 | 1.383(1.351-1.417,*P<*.001) | -477100000  0000** |  |  |  |
| Frequently | 6319 | 9287 | 1.753(1.693-1.814,*P<*.001) | 1.242097811 | *P<*.001 | 0.011 | 1.045(0.994-1.099,*P=*.08)** |
| Always | 1657 | 2358 | 1.593 (1.494–1.698, P < 0.001) | 1.194600676 | *P<*.001 | 0.004 | 1.069 (0.977–1.169, P = 0.14)** |
| (YSFY5)Children are rarely encouraged to taste new foods | | | | | | | |
| Never | 33595 | 24389 | 0.619 (0.604–0.634, P < 0.001) | .** |  |  |  |
| Occasionally | 15952 | 18617 | 1.444 (1.408–1.482, P < 0.001) | 1.398662184 | *P=*.75** |  |  |
| Frequently | 6016 | 7064 | 1.338 (1.290–1.388, P < 0.001) | 1.217327852 | *P=*.19** |  |  |
| Always | 1252 | 1562 | 1.384 (1.284–1.493, P < 0.001) | 1.097496694 | *P=*.20** |  |  |
| (YSFY6)Allow children to eat snacks if they want | | | | | | | |
| Never | 30035 | 21340 | 0.628 (0.613–0.643, P < 0.001) | 1.382219821 | *P=*.03** |  |  |
| Occasionally | 24771 | 27167 | 1.436 (1.402–1.471, P < 0.001) | 4037000000000** |  |  |  |
| Frequently | 1743 | 2689 | 1.736 (1.632–1.846, P < 0.001) | 1.158126782 | *P=*.72** |  |  |
| Always | 266 | 436 | 1.810 (1.554–2.110, P < 0.001) | 1.148779053 | *P=*.60** |  |  |
| (YSFY7)Allow children to dine and wander around | | | | | | | |
| Never | 45124 | 34924 | 0.542 (0.527–0.557, P < 0.001) | 4145000000000** |  |  |  |
| Occasionally | 10754 | 14494 | 1.672 (1.625–1.720, P < 0.001) | 1.346292541 | *P<*.001 | 0.003 | 1.013(0.9700-1.058,*P=*.57)** |
| Frequently | 786 | 1840 | 2.634(2.421-2.866,*P<*.001) | 1.237639504 | *P<*.001 | 0.005 | 1.141(1.010-1.289,*P=*.03)** |
| Always | 151 | 374 | 2.738(2.266-3.309,*P<*.001) | 1.190431727 | *P=*.09** |  |  |
| (YS1)child's average meal time | | | | | | | |
| ＜25min | 25292 | 17422 | 0.635(0.619-0.651,*P<*.001) | .** |  |  |  |
| 25-45min | 28558 | 29197 | 1.288(1.257-1.319,*P<*.001) | 1.258280111 | *P<*.001 | 0.009 | 1.065(1.027-1.104,*P<*.001) |
| ＞45min | 2965 | 5013 | 1.953(1.863-2.047,*P<*.001) | 1.342739748 | *P<*.001 | 0.011 | 1.096(1.016-1.182,*P=*.02)** |
| (YS2)Parental attitude about children's picky eating | | | | | | | |
| Worry | 25459 | 33191 | 2.217(2.163-2.272,*P<*.001) | .** |  |  |  |
| Do not worry | 29327 | 16436 | 0.438(0.427-0.449,*P<*.001) | 1.534487075 | *P<*.001 | -0.051 | 0.824 (95% CI: 0.792–0.857; *P<* .001) |
| Do not care | 2029 | 2005 | 1.091 (95% CI: 1.024–1.162; *P<* .001) | 1.055353996 | *P=*.98** |  |  |
| Continuous variables | | | | | | | |
| Age(Month),Median(IQR) | 62.00 (IQR: 54.00–72.00) | 61.00 (IQR: 53.00–70.00) | 0.986 (95% CI: 0.985–0.988; *P<* .001) | 2.22526152 | *P<*.001 | 0** |  |
| Height(cm),Median(IQR) | 112.00 (IQR: 106.00–118.00) | 110.00 (IQR: 105.00–116.00) | 0.978 (95% CI: 0.977–0.980; *P<* .001) | 36.26886935** |  |  |  |
| Weight(kg),Median(IQR) | 19.00 (IQR: 17.00–22.00) | 18.00 (IQR: 16.00–21.00) | 0.954 (95% CI: 0.951–0.957; *P<* .001) | 61.54928264** |  |  |  |
| BMI,Median(IQR) | 15.13 (IQR: 14.06–16.53) | 15.00 (IQR: 14.00–16.29) | 0.972(0.967-0.976,*P<*.001) | 37.41086856** |  |  |  |
| The age of the father when the child is born,Median(IQR) | 31.00(28.00,35.00) | 31.00(28.00,34.00) | 1.000(0.999-1.001,*P=*.99)** |  |  |  |  |
| The age of the mother when the child is born,Median(IQR) | 29.00(26.00,32.00) | 29.00(26.00,32.00) | 1.000(0.998-1.002,*P=*.81)** |  |  |  |  |
| Daytime sleep time(h),Median(IQR) | 2.00(1.00,2.00) | 1.50(1.00,2.00) | 0.965(0.955-0.975,*P<*.001) | 1.387087193 | *P<*.001 | -0.001 | 1.013(0.995-1.031,*P=*.16)** |
| Sleep time at night(h),Median(IQR) | 9.00(8.00,9.00) | 9.00(8.00,9.00) | 0.902(0.891-0.913,*P<*.001) | 1.682444033 | *P<*.001 | -0.004, 0.002 | 0.982(0.960-1.004,*P=*.10)** |
| Average sleep time per day(h),Median(IQR) | 10.00(9.00,11.00) | 10.00(9.00,10.00) | 0.888(0.879-0.898,*P<*.001) | 1.700549737 | *P<*.001 | 0.012 | 0.969(0.950-0.989,*P=*.002)** |

**Table S3. Comparison of baseline characteristics between children included in and excluded from model training**

| Variable | Data type | Included group (Mean ± SD)  SD | Excluded group (Mean ± SD)  SD | *P-value* | Included group (n, %) | Excluded group (n, %) |
| --- | --- | --- | --- | --- | --- | --- |
| Age_Month | Continuous variables | 62.55±  10.93 | 62.62±  10.94 | *P=*.31 |  |  |
| Height_cm | Continuous variables | 111.68±  7.97 | 111.52±  8.06 | *P=*.001 |  |  |
| Weight_kg | Continuous variables | 19.26±  3.55 | 19.23±  3.59 | *P=*.21 |  |  |
| BMI | Continuous variables | 15.36±  1.96 | 15.37±  2.00 | *P=*.21 |  |  |
| The_age_of_the_father  _when_the_child_is_born | Continuous variables | 31.39±  5.01 | 31.36±  5.09 | *P=*.25 |  |  |
| The_age_of_the_mother  _when_the_child_is_born | Continuous variables | 29.22±  4.47 | 29.12±  4.57 | *P=*.0012 |  |  |
| Daytime_sleep_time_h | Continuous variables | 1.56±  0.66 | 1.55±  0.69 | *P=*.0059 |  |  |
| Sleep_time_at_night_h | Continuous variables | 8.70±  0.79 | 8.68±  0.80 | *P<*.001 |  |  |
| Average_sleep_time_per  _day_h | Continuous variables | 9.92±  1.05 | 9.87±  1.06 | *P<*.001 |  |  |
| Grade_Kindergarten_class | Classification |  |  | *P<*.001 | Category 0: 33951 (68.2%); Category 1: 15847 (31.8%) | Category 0: 40805 (69.6%); Category 1: 17844 (30.4%) |
| Grade_Middle_class | Classification |  |  | *P=*.0015 | 0:33720(67.7%) 1:16078(32.3%) | 0:39179(66.8%) 1:19470(33.2%) |
| Grade_Senior_class | Classification |  |  | *P=*.12 | 0:32037(64.3%) 1:17761(35.7%) | 0:37461(63.9%) 1:21188(36.1%) |
| Grade_other | Classification |  |  | *P=*.42 | 0:49686(99.8%) 1:112(0.2%) | 0:58502(99.7%) 1:147(0.3%) |
| Gender | Classification |  |  | *P<*.001 | 0:23770(47.7%) 1:26028(52.3%) | 0:27053(46.1%) 1:31596(53.9%) |
| Age_3 | Classification |  |  | *P=*.41 | 0:45782(91.9%) 1:4016(8.1%) | 0:53837(91.8%) 1:4812(8.2%) |
| Age_4 | Classification |  |  | *P=*0.01 | 0:32010(64.3%) 1:17788(35.7%) | 0:38179(65.1%) 1:20470(34.9%) |
| Age_5 | Classification |  |  | *P=*0.01 | 0:34463(69.2%) 1:15335(30.8%) | 0:40180(68.5%) 1:18469(31.5%) |
| Age_6 | Classification |  |  | *P=*.67 | 0:37473(75.3%) 1:12325(24.7%) | 0:44200(75.4%) 1:14449(24.6%) |
| Age_7 | Classification |  |  | *P=*.07 | 0:49464(99.3%) 1:334(0.7%) | 0:58200(99.2%) 1:449(0.8%) |
| Time_of_birth_1 | Classification |  |  | *P=*.01 | 0:45957(92.3%) 1:3841(7.7%) | 0:53886(91.9%) 1:4763(8.1%) |
| Time_of_birth_2 | Classification |  |  | *P=*0.26 | 0:46689(93.8%) 1:3109(6.2%) | 0:54888(93.6%) 1:3761(6.4%) |
| Time_of_birth_3 | Classification |  |  | *P=*0.65 | 0:46136(92.6%) 1:3662(7.4%) | 0:54292(92.6%) 1:4357(7.4%) |
| Time_of_birth_4 | Classification |  |  | *P=*.93 | 0:44781(89.9%) 1:5017(10.1%) | 0:52729(89.9%) 1:5920(10.1%) |
| Time_of_birth_5 | Classification |  |  | *P=*.02 | 0:41688(83.7%) 1:8110(16.3%) | 0:49399(84.2%) 1:9250(15.8%) |
| Time_of_birth_6 | Classification |  |  | *P=*.79 | 0:44718(89.8%) 1:5080(10.2%) | 0:52636(89.7%) 1:6013(10.3%) |
| Time_of_birth_7 | Classification |  |  | *P=*1 | 0:45520(91.4%) 1:4278(8.6%) | 0:53612(91.4%) 1:5037(8.6%) |
| Time_of_birth_8 | Classification |  |  | *P=*.51 | 0:45257(90.9%) 1:4541(9.1%) | 0:53231(90.8%) 1:5418(9.2%) |
| Time_of_birth_9 | Classification |  |  | *P=*.12 | 0:46029(92.4%) 1:3769(7.6%) | 0:54358(92.7%) 1:4291(7.3%) |
| Time_of_birth_10 | Classification |  |  | *P=*.87 | 0:46598(93.6%) 1:3200(6.4%) | 0:54896(93.6%) 1:3753(6.4%) |
| Time_of_birth_11 | Classification |  |  | *P=*.07 | 0:47027(94.4%) 1:2771(5.6%) | 0:55532(94.7%) 1:3117(5.3%) |
| Time_of_birth_12 | Classification |  |  | *P=*.13 | 0:47378(95.1%) 1:2420(4.9%) | 0:55680(94.9%) 1:2969(5.1%) |
| Ethnic_group | Classification |  |  | *P=*.02 | 0:2252(4.5%) 1:47546(95.5%) | 0:2825(4.8%) 1:55824(95.2%) |
| Only_child | Classification |  |  | *P=*.001 | 0: 13,688 (27.5%) 1: 36,110 (72.5%) | 0: 15,604 (26.6%) 1: 43,045 (73.4%) |
| Term_infant | Classification |  |  | *P<*.001 | 0: 2,460 (4.9%) 1: 47,338 (95.1%) | 0: 3,513 (6.0%) 1: 55,136 (94.0%) |
| Caesarean_birth | Classification |  |  | *P=*.84 | 0: 5,404 (10.9%) 1: 44,394 (89.1%) | 0: 7,067 (12.0%) 1: 51,582 (88.0%) |
| Birth weight 2.5-4 kg | Classification |  |  | *P<*.001 | 0: 46,616 (93.6%) 1: 3,182 (6.4%) | 0: 54,123 (92.3%) 1: 4,526 (7.7%) |
| Birth_weight_Low_  <2.5 kg | Classification |  |  | *P<*.001 | 0: 47,576 (95.5%) 1: 2,222 (4.5%) | 0: 56,108 (95.7%) 1: 2,541 (4.3%) |
| Birth_weight_More_  than_4kg | Classification |  |  | *P=*.31 | 0:16015(32.2%) 1:33783(67.8%) | 0:19990(34.1%) 1:38659(65.9%) |
| Which_child_of_  father_first | Classification |  |  | *P<*.001 | 0:49481(99.4%) 1:317(0.6%) | 0:58219(99.3%) 1:430(0.7%) |
| Which_child_of_  father_second | Classification |  |  | *P<*.001 | 0:49649(99.7%) 1:149(0.3%) | 0:58470(99.7%) 1:179(0.3%) |
| Which_child_of_  father_third | Classification |  |  | *P<*.001 | 0:35052(70.4%) 1:14746(29.6%) | 0:40525(69.1%) 1:18124(30.9%) |
| Which_child_of_father_  Fourth_and_After_the_fourth | Classification |  |  | *P=*.005 | 0:49757(99.9%) 1:41(0.1%) | 0:58594(99.9%) 1:55(0.1%) |
| Which_child_of_mother  _First | Classification |  |  | *P<*.001 | 0:49347(99.1%) 1:451(0.9%) | 0:58,060 (99.0%) 1:589 (1.0%) |
| Which_child_of_mother  _Second | Classification |  |  | *P<*.001 | 0:48,540 (97.5%) 1:1,258 (2.5%) | 0:56,829 (96.9%) 1:1,820 (3.1%) |
| Which_child_of_mother  _Third | Classification |  |  | *P<*.001 | 0:9,802 (19.7%) 1:39,996 (80.3%) | 0:11,826 (20.2%) 1:46,823 (79.8%) |
| Which_child_of_mother  _Fourth_and_after_the_fourth | Classification |  |  | *P=*.002 | 0:40,455 (81.2%) 1:9,343 (18.8%) | 0:47,522 (81.0%) 1:11,127 (19.0%) |
| Exclusively_breast_fed_within  _6_months_after_birth | Classification |  |  | *P<*.001 | 0:49,339 (99.1%) 1:459 (0.9%) | 0:57,950 (98.8%) 1:699 (1.2%) |
| Feed_Fresh_milk | Classification |  |  | *P=*0.06 | 0:45,323 (91.0%) 1:4,475 (9.0%) | 0:53,165 (90.6%) 1:5,484 (9.4%) |
| Feed_Fresh_ewe_milk | Classification |  |  | *P=*0.90 | 0:11711(23.5%) 1:38087(76.5%) | 0:14462(24.7%) 1:44187(75.3%) |
| Feed_Formula_milk_powder | Classification |  |  | *P<*.001 | 0:49411(99.2%) 1:387(0.8%) | 0:58140(99.1%) 1:509(0.9%) |
| Feed_soymilk | Classification |  |  | *P=*0.60 | 0:44367(89.1%) 1:5431(10.9%) | 0:52180(89.0%) 1:6469(11.0%) |
| Feed_Ewe_milk_power | Classification |  |  | *P=*0.10 | 0:48841(98.1%) 1:957(1.9%) | 0:57314(97.7%) 1:1335(2.3%) |
| Feed_Others | Classification |  |  | *P<*.001 | 0:49337(99.1%) 1:461(0.9%) | 0:58241(99.3%) 1:408(0.7%) |
| Time_of_adding_animal  _protein_complementary  _food_After_6th_months | Classification |  |  | *P=*0.05 | 0:35385(71.1%) 1:14413(28.9%) | 0:40300(68.7%) 1:18349(31.3%) |
| Time_of_adding_animal  _protein_complementary  _food_between_4th_and_6thmonths | Classification |  |  | *P=*0.38 | 0:42874(86.1%) 1:6924(13.9%) | 0:50176(85.6%) 1:8473(14.4%) |
| Time_of_adding_animal  _protein_complementary  _food_within_3_months | Classification |  |  | *P<*.001 | 0:43285(86.9%) 1:6513(13.1%) | 0:50285(85.7%) 1:8364(14.3%) |
| Have_been_treated  _with_antibiotics | Classification |  |  | *P<*.001 | 0:48881(98.2%) 1:917(1.8%) | 0:57350(97.8%) 1:1299(2.2%) |
| The_earliest_age_of  _using_antibiotics_None | Classification |  |  | *P<*.001 | 0:47042(94.5%) 1:2756(5.5%) | 0:55203(94.1%) 1:3446(5.9%) |
| The_earliest_age_of  _using_antibiotics  _Within_1_age | Classification |  |  | *P<*.001 | 0:48964(98.3%) 1:834(1.7%) | 0:57486(98.0%) 1:1163(2.0%) |
| The_earliest_age_of  _using_antibiotics  _1__and_2_age | Classification |  |  | *P<*.001 | 0:47774(95.9%) 1:2024(4.1%) | 0:55811(95.2%) 1:2838(4.8%) |
| The_earliest_age_of_  using_antibiotics_  After_3_age | Classification |  |  | *P<*.001 | 0:49019(98.4%) 1:779(1.6%) | 0:57562(98.1%) 1:1087(1.9%) |
| Frequency_of_antibiotic_use  _within_1_year_old_None | Classification |  |  | *P<*.001 | 0:49355(99.1%) 1:443(0.9%) | 0:58015(98.9%) 1:634(1.1%) |
| Frequency_of_antibiotic_use  _within_1_year_old_Once | Classification |  |  | *P=*0.06 | 0:49634(99.7%) 1:164(0.3%) | 0:58427(99.6%) 1:222(0.4%) |
| Frequency_of_antibiotic_use  _within_1_year_old_Twice | Classification |  |  | *P=*0.03 | 0:49767(99.9%) 1:31(0.1%) | 0:58593(99.9%) 1:56(0.1%) |
| Frequency_of_antibiotic_use_  within_1_year_old_3_  Times_or_more | Classification |  |  | *P<*.001 | 0:49787(100.0%) 1:11(0.0%) | 0:58624(100.0%) \| 1:25(0.0%) |
| Children_usually_have_the_  most_contact_with_people_  outside_kindergarten_time_Father | Classification |  |  | *P=*0.04 | 0:49204(98.8%) 1:594(1.2%) | 0:57873(98.7%) 1:776(1.3%) |
| Children_usually_have_the_  most_contact_with_people_  outside_kindergarten_time_Mother | Classification |  |  | *P<*.001 | 0:49549(99.5%) 1:249(0.5%) | 0:58265(99.3%) 1:384(0.7%) |
| Children_usually_have_the_  most_contact_with_people_  outside_kindergarten_time_Father/Mother | Classification |  |  | *P=*0.11 | 0:13555(27.2%) 1:36243(72.8%) | 0:18218(31.1%) 1:40431(68.9%) |
| Children_usually_have_the_  most_contact_with_people_  outside_kindergarten_time_Grandparents | Classification |  |  | *P=*0.52 | 0:18519(37.2%) 1:31279(62.8%) | 0:21117(36.0%) 1:37532(64.0%) |
| Children_usually_have_the_  most_contact_with_people_  outside_kindergarten_time_  Parents_and_Grandparents | Classification |  |  | *P<*.001 | 0:37235(74.8%) 1:12563(25.2%) | 0:44863(76.5%) 1:13786(23.5%) |
| Children_usually_have_the_  most_contact_with_people_  outside_kindergarten_time_Others | Classification |  |  | *P<*.001 | 0:43842(88.0%) 1:5956(12.0%) | 0:51318(87.5%) 1:7331(12.5%) |
| History_of_allergies | Classification |  |  | *P<*.001 | 0:36609(73.5%) 1:13189(26.5%) | 0:44134(75.3%) 1:14515(24.7%) |
| Eczema_or_atopic_dermatitis | Classification |  |  | *P=*0.01 | 0:45927(92.2%) 1:3871(7.8%) | 0:48574(82.8%) 1:10075(17.2%) |
| Allergic_rhinitis | Classification |  |  | *P<*.001 | 0:3871(7.8%) 1:45927(92.2%) | 0:10075(17.2%) 1:48574(82.8%) |
| Drug_allergy | Classification |  |  | *P<*.001 | 0:49,186 (98.8%) 1:612 (1.2%) | 0:57,916 (98.8%) 1:733 (1.2%) |
| Food_allergy | Classification |  |  | *P=*0.02 | 0:48,404 (97.2%) 1:1,394 (2.8%) | 0:57,051 (97.3%) 1:1,598 (2.7%) |
| Other_allergic_history | Classification |  |  | *P<*.001 | 0:48,466 (97.3%) 1:1,332 (2.7%) | 0:57,175 (97.5%) 1:1,474 (2.5%) |
| History_of_digestive_system  _diseases | Classification |  |  | *P<*.001 | 0:47,661 (95.7%) 1:2,137 (4.3%) | 0:56,213 (95.8%) 1:2,436 (4.2%) |
| Functional_dyspepsia | Classification |  |  | *P<*.001 | 0:49,725 (99.9%) 1:73 (0.1%) | 0:52,846 (90.1%) 1:5,803 (9.9%) |
| Functional_constipation | Classification |  |  | *P=*0.002 | 0: 43,297 (86.9%) 1: 6,501 (13.1%) | 0: 50,795 (86.6%) 1: 7,854 (13.4%) |
| gastritis | Classification |  |  | *P=*0.19 | 0: 8,180 (16.4%) 1: 41,618 (83.6%) | 0: 10,185 (17.4%) 1: 48,464 (82.6%) |
| Gastroesophageal_reflux | Classification |  |  | *P=*0.07 | 0: 49,326 (99.1%) 1: 472 (0.9%) | 0: 58,000 (98.9%) 1: 649 (1.1%) |
| Peptic_ulcer | Classification |  |  | *P=*0.09 | 0: 48,879 (98.2%) 1: 919 (1.8%) | 0: 57,396 (97.9%) 1: 1,253 (2.1%) |
| Mesenteric_lymphadenitis | Classification |  |  | *P=*0.06 | 0: 49,622 (99.6%) 1: 176 (0.4%) | Class 0: 58,046 (99.0%) Class 1: 603 (1.0%) |
| Other_digestive_system  _diseases | Classification |  |  | *P=*0.001 | Class 0: 49,686 (99.8%) Class 1: 112 (0.2%) | Class 0: 58,476 (99.7%) Class 1: 173 (0.3%) |
| History_of_major_diseases | Classification |  |  | *P=*0.03 | Class 0: 49,723 (99.8%) Class 1: 75 (0.2%) | Class 0: 58,470 (99.7%) Class 1: 179 (0.3%) |
| Activity_habits_inactivity | Classification |  |  | *P=*0.20 | Class 0: 49,491 (99.4%) Class 1: 307 (0.6%) | Class 0: 58,030 (98.9%) Class 1: 619 (1.1%) |
| Activity_habits_Low_  activity_levels | Classification |  |  | *P<*.001 | Class 0: 42,668 (85.7%) Class 1: 7,130 (14.3%) | Class 0: 48,325 (82.4%) Class 1: 10,324 (17.6%) |
| Activity_habits_general  _activity_levels | Classification |  |  | *P<*.001 | Class 0: 39,124 (78.6%) Class 1: 10,674 (21.4%) | 0:45053(76.8%) 1:13596(23.2%) |
| Activity_habits_High  _activity_levels | Classification |  |  | *P<*.001 | 0:37205(74.7%) 1:12593(25.3%) | 0:44327(75.6%) 1:14322(24.4%) |
| Physical_balance_and_flexibility  _in_the_last_year_Acceptable | Classification |  |  | *P<*.001 | 0:33267(66.8%) 1:16531(33.2%) | 0:41518(70.8%) 1:17131(29.2%) |
| Physical_balance_and_flexibility  _in_the_last_year_Good | Classification |  |  | *P<*.001 | 0:47310(95.0%) 1:2488(5.0%) | 0:56171(95.8%) 1:2478(4.2%) |
| Physical_balance_and_flexibility  _in_the_last_year_Very_good | Classification |  |  | *P<*.001 | 0:49686(99.8%) 1:112(0.2%) | 0:58423(99.6%) 1:226(0.4%) |
| Like_music_or_not_Resistance | Classification |  |  | *P=*0.99 | 0:49303(99.0%) 1:495(1.0%) | 0:57650(98.3%) 1:999(1.7%) |
| Like_music_or_not_Dislike | Classification |  |  | *P=*0.03 | 0:42516(85.4%) 1:7282(14.6%) | 0:47596(81.2%) 1:11053(18.8%) |
| Like_music_or_not_Numb | Classification |  |  | *P<*.001 | 0:39386(79.1%) 1:10412(20.9%) | 0:45918(78.3%) 1:12731(21.7%) |
| Like_music_or_not_Like | Classification |  |  | *P=*0.47 | 0:34636(69.6%) 1:15162(30.4%) | 0:41549(70.8%) 1:17100(29.2%) |
| Like_music_or_not_Favorite | Classification |  |  | *P<*.001 | 0:35075(70.4%) 1:14723(29.6%) | 0:43618(74.4%) 1:15031(25.6%) |
| Regular_lunch_breaks | Classification |  |  | *P<*.001 | 0:48186(96.8%) 1:1612(3.2%) | 0:57140(97.4%) 1:1509(2.6%) |
| Food_preference_No_obvious | Classification |  |  | *P<*.001 | 0:18324(36.8%) 1:31474(63.2%) | 0:22785(38.8%) 1:35864(61.2%) |
| Food_preference_Have_much  _loved_food | Classification |  |  | *P<*.001 | 0:34762(69.8%) 1:15036(30.2%) | 0:40193(68.5%) 1:18456(31.5%) |
| Food_preference_Have_much  _hated_food | Classification |  |  | *P=*0.01 | 0:46721(93.8%) 1:3077(6.2%) | 0:54611(93.1%) 1:4038(6.9%) |
| Supplementing_nutrients_(suchasvitamins)  _separately_fromdaily_diet | Classification |  |  | *P<*.001 | 0:49624(99.7%) 1:174(0.3%) | 0:58413(99.6%) 1:236(0.4%) |
| With_dental_caries | Classification |  |  | *P<*.001 | 0:49761(99.9%) 1:37(0.1%) | 0:58594(99.9%) 1:55(0.1%) |
| Dental_caries_have_been  _treated_None | Classification |  |  | *P=*0.60 | 0:16811(33.8%) 1:32987(66.2%) | Class 0: 21,519 (36.7%) Class 1: 37,130 (63.3%) |
| Dental_caries_have_been  _treated_occasionally | Classification |  |  | *P=*0.65 | Class 0: 40,095 (80.5%) Class 1: 9,703 (19.5%) | Class 0: 46,375 (79.1%) Class 1: 12,274 (20.9%) |
| Dental_caries_have_been  _treated_usually | Classification |  |  | *P=*0.83 | Class 0: 45,006 (90.4%) Class 1: 4,792 (9.6%) | Class 0: 52,448 (89.4%) Class 1: 6,201 (10.6%) |
| Whether_there_is_  abnormal_vision | Classification |  |  | *P<*.001 | Class 0: 48,135 (96.7%) Class 1: 1,663 (3.3%) | Class 0: 56,513 (96.4%) Class 1: 2,136 (3.6%) |
| Normal_vision | Classification |  |  | *P<*.001 | Class 0: 49,145 (98.7%) Class 1: 653 (1.3%) | Class 0: 57,741 (98.5%) Class 1: 908 (1.5%) |
| short_sighted | Classification |  |  | *P=*0.78 | 0:15363(30.9%) 1:34435(69.1%) | 0:19816(33.8%) 1:38833(66.2%) |
| Amblyopia | Classification |  |  | *P=*0.47 | 0:40478(81.3%) 1:9320(18.7%) | 0:46895(80.0%) 1:11754(20.0%) |
| Astigmatism | Classification |  |  | *P=*0.10 | 0:45907(92.2%) 1:3891(7.8%) | 0:53496(91.2%) 1:5153(8.8%) |
| Other_visual_abnormalities | Classification |  |  | *P=*0.27 | 0:48277(96.9%) 1:1521(3.1%) | 0:56622(96.5%) 1:2027(3.5%) |
| unclear | Classification |  |  | *P<*.001 | 0:49167(98.7%) 1:631(1.3%) | 0:57767(98.5%)  1:882(1.5%) |
| Have_vision_correction  _therapy | Classification |  |  | *P=*0.07 | 0:19581(39.3%) 1:30217(60.7%) | 0:23310(39.7%) 1:35339(60.3%) |
| Language_comprehension_and  _expression_ability_  Expression_Not_yet_clear | Classification |  |  | *P<*.001 | 0:37702(75.7%) 1:12096(24.3%) | 0:44294(75.5%) 1:14355(24.5%) |
| Language_comprehension_  and_expression_ability  _Respond_clearly | Classification |  |  | *P<*.001 | 0:43055(86.5%) 1:6743(13.5%) | 0:50577(86.2%) 1:8072(13.8%) |
| Language_comprehension_and  _expression_ability_Respond  _with_complex_sentence | Classification |  |  | *P<*.001 | 0:49173(98.7%) 1:625(1.3%) | 0:57921(98.8%) 1:728(1.2%) |
| Self_control_ability_Rambunctious | Classification |  |  | *P<*.001 | 0:49681(99.8%) 1:117(0.2%) | 0:58494(99.7%) 1:155(0.3%) |
| Self_control_ability_Not_good  _at_selfcontrol_but_can  _accept_discipline | Classification |  |  | *P=*0.11 | 0:6,750 (13.6%) 1:43,048 (86.4%) | 0:8,897 (15.2%) 1:49,752 (84.8%) |
| Self_control_ability_Good  _at_self_control | Classification |  |  | *P<*.001 | 0:44,912 (90.2%) 1:4,886 (9.8%) | 0:52,289 (89.2%) 1:6,360 (10.8%) |
| Whether_to_start_subject_  education(such_as_studying_  mathematics_and_English_alone,etc.) | Classification |  |  | *P<*.001 | 0:48,251 (96.9%) 1:1,547 (3.1%) | 0:56,551 (96.4%) 1:2,098 (3.6%) |
| Who_educates_children_the_  longest_in_the_family_at_  ordinary_times_Father | Classification |  |  | *P=*0.10 | 0:49,552 (99.5%) 1:246 (0.5%) | 0:58,321 (99.4%) 1:328 (0.6%) |
| Who_educates_children_the  _longest_in_the_family_at  _ordinary_times_Mother | Classification |  |  | *P<*.001 | 0:49,727 (99.9%) 1:71 (0.1%) | 0:58,538 (99.8%) 1:111 (0.2%) |
| Who_educates_children_the  _longest_in_the_family_at_  ordinary_times_Parents | Classification |  |  | *P=*0.01 | 0:8,430 (16.9%) 1:41,368 (83.1%) | 0:10993(18.7%) 1:47656(81.3%) |
| Who_educates_children_  the_longest_in_the_family_  at_ordinary_times_Grandparents | Classification |  |  | *P<*.001 | 0:43977(88.3%) 1:5821(11.7%) | 0:51159(87.2%) 1:7490(12.8%) |
| Who_educates_children_  the_longest_in_the_family_  at_ordinary_times_  Parents_and_Grandparents | Classification |  |  | *P<*.001 | 0:47575(95.5%) 1:2223(4.5%) | 0:55731(95.0%) 1:2918(5.0%) |
| Who_educates_children_the  _longest_in_the_family_at  _ordinary_times_Others | Classification |  |  | *P=*0.03 | 0:49508(99.4%) 1:290(0.6%) | 0:58232(99.3%) 1:417(0.7%) |
| The_highest_education_of_the_child's_father_Below_elementary_school | Classification |  |  | *P<*.001 | 0:49702(99.8%) 1:96(0.2%) | 0:58481(99.7%) 1:168(0.3%) |
| The_highest_education_of_the  _child's_father_Elementary_school | Classification |  |  | *P<*.001 | 0:38795(77.9%) 1:11003(22.1%) | 0:46616(79.5%) 1:12033(20.5%) |
| The_highest_education_of_  the_child's_father_Junior_high_school | Classification |  |  | *P<*.001 | Category 0: 37,169 (74.6%); Category 1: 12,629 (25.4%) | Category 0: 43,998 (75.0%); Category 1: 14,651 (25.0%) |
| The_highest_education_of_the  _child's_father_High_school/Technical  _secondary_school | Classification |  |  | *P<*.001 | 0:37235(74.8%) 1:12563(25.2%) | Category 0: 43,685 (74.5%); Category 1: 14,964 (25.5%) |
| The_highest_education_of_the  _child's_father_Associate_degree | Classification |  |  | *P=*0.001 | Category 0: 39,835 (80.0%); Category 1: 9,963 (20.0%) | Category 0: 46,426 (79.2%); Category 1: 12,223 (20.8%) |
| The_highest_education_of_  the_child's_father_Bachelor_degree. | Classification |  |  | *P<*.001 | Category 0: 46,158 (92.7%); Category 1: 3,640 (7.3%) | Category 0: 53,871 (91.9%); Category 1: 4,778 (8.1%) |
| The_highest_education_of_  the_child's_father_Graduate_degree. | Classification |  |  | *P<*.001 | Category 0: 12,163 (24.4%); Category 1: 37,635 (75.6%) | Category 0: 15,693 (26.8%); Category 1: 42,956 (73.2%) |
| The_highest_education_of_  the_child's_mother_Below_  elementary_school | Classification |  |  | *P<*.001 | Category 0: 41,023 (82.4%); Category 1: 8,775 (17.6%) | Category 0: 47,493 (81.0%); Category 1: 11,156 (19.0%) |
| The_highest_education_of  _the_child's_mother_  Elementary_school | Classification |  |  | *P<*.001 | 0:47034(94.4%) 1:2764(5.6%) | 0:54996(93.8%) 1:3653(6.2%) |
| The_highest_education_of  _the_child's_mother  _Junior_high_school | Classification |  |  | *P<*.001 | 0:49306(99.0%) 1:492(1.0%) | 0:58000(98.9%) 1:649(1.1%) |
| The_highest_education_of  _the_child's_mother_High_school  /Technical_secondary_school | Classification |  |  | *P=*0.001 | 0:49666(99.7%) 1:132(0.3%) | 0:58414(99.6%) 1:235(0.4%) |
| The_highest_education_of_  the_child's_mother_Associate_degree | Classification |  |  | *P<*.001 | 0:11541(23.2%) 1:38257(76.8%) | 0:15065(25.7%) 1:43584(74.3%) |
| The_highest_education_of_  the_child's_mother_Bachelor_degree. | Classification |  |  | *P<*.001 | 0:41968(84.3%) 1:7830(15.7%) | 0:48564(82.8%) 1:10085(17.2%) |
| The_highest_education_of_  the_child's_mother_Graduate_degree. | Classification |  |  | *P<*.001 | 0:46831(94.0%) 1:2967(6.0%) | 0:54727(93.3%) 1:3922(6.7%) |
| Per_capita_monthly_income_of_  families_Lower_than_1000RMB | Classification |  |  | *P<*.001 | 0:49231(98.9%) 1:567(1.1%) | 0:57855(98.6%) 1:794(1.4%) |
| Per_capita_monthly_income_of_  families_Between_1000_and_2000RMB | Classification |  |  | *P<*.001 | 0:49621(99.6%) 1:177(0.4%) | 0:58385(99.5%) 1:264(0.5%) |
| Per_capita_monthly_income_of_  families_Between_2000_and_5000RMB | Classification |  |  | *P<*.001 | 0:8472(17.0%) 1:41326(83.0%) | 0:10975(18.7%) 1:47674(81.3%) |
| Per_capita_monthly_income_of_  families_Between_5000_and_10000RMB | Classification |  |  | *P=*0.003 | 0:43541(87.4%)1:6257(12.6%) | 0:50669(86.4%) 1:7980(13.6%) |
| Per_capita_monthly_income_of_  families_Between_10000_and_20000RMB | Classification |  |  | *P<*.001 | 0:48036(96.5%) 1:1762(3.5%) | 0:56303(96.0%) 1:2346(4.0%) |
| Per_capita_monthly_income_of_  families_Higher_than_20000RMB | Classification |  |  | *P<*.001 | 0:49454(99.3%) 1:344(0.7%) | 0:58168(99.2%) 1:481(0.8%) |
| TZ1_Easy_to_lose_spirit_1 | Classification |  |  | *P<*.001 | 0:49689(99.8%) 1:109(0.2%) | 0:58481(99.7%) 1:168(0.3%) |
| TZ1_Easy_to_lose_spirit_2 | Classification |  |  | *P<*.001 | 0:12146(24.4%) 1:37652(75.6%) | 0:15314(26.1%) 1:43335(73.9%) |
| TZ1_Easy_to_lose_spirit_3 | Classification |  |  | *P<*.001 | 0:41382(83.1%) 1:8416(16.9%) | 0:48269(82.3%) 1:10380(17.7%) |
| TZ1_Easy_to_lose_spirit_4 | Classification |  |  | *P=*0.17 | 0:46961(94.3%) 1:2837(5.7%) | 0:54868(93.6%) 1:3781(6.4%) |
| TZ1_Easy_to_lose_spirit_5 | Classification |  |  | *P=*0.32 | 0:49156(98.7%) 1:642(1.3%) | 0:57815(98.6%) 1:834(1.4%) |
| TZ2_Yellow_complexion,no_  other_children_ruddy_1 | Classification |  |  | *P<*.001 | 0:49547(99.5%) 1:251(0.5%) | 0:58330(99.5%) 1:319(0.5%) |
| TZ2_Yellow_complexion,no_  other_children_ruddy_2 | Classification |  |  | *P<*.001 | 0:15262(30.6%) 1:34536(69.4%) | 0:19334(33.0%) 1:39315(67.0%) |
| TZ2_Yellow_complexion,no  _other_children_ruddy_3 | Classification |  |  | *P<*.001 | 0:38310(76.9%) 1:11488(23.1%) | 0:44288(75.5%) 1:14361(24.5%) |
| TZ2_Yellow_complexion,no_  other_children_ruddy_4 | Classification |  |  | *P=*0.007 | 0:46621(93.6%) 1:3177(6.4%) | 0:54530(93.0%) 1:4119(7.0%) |
| TZ2_Yellow_complexion,no_  other_children_ruddy_5 | Classification |  |  | *P=*0.001 | 0:49360(99.1%) 1:438(0.9%) | 0:57996(98.9%) 1:653(1.1%) |
| TZ3_Soft_muscles,not_as_  strong_as_other_children_1 | Classification |  |  | *P<*.001 | 0:49639(99.7%) 1:159(0.3%) | 0:58448(99.7%)  1:201(0.3%) |
| TZ3_Soft_muscles,not_as_  strong_as_other_children_2 | Classification |  |  | *P<*.001 | 0:14722(29.6%) 1:35076(70.4%) | 0:18574(31.7%) 1:40075(68.3%) |
| TZ3_Soft_muscles,not_as_  strong_as_other_children_3 | Classification |  |  | *P<*.001 | 0:39535(79.4%) 1:10263(20.6%) | 0:45767(78.0%) 1:12882(22.0%) |
| TZ3_Soft_muscles,not_as_  strong_as_other_children_4 | Classification |  |  | *P<*.001 | 0:46439(93.3%) 1:3359(6.7%) | 0:54218(92.4%) 1:4431(7.6%) |
| TZ3_Soft_muscles,not_as_  strong_as_other_children_5 | Classification |  |  | *P=*0.001 | 0:48902(98.2%) 1:896(1.8%) | 0:57651(98.3%) 1:998(1.7%) |
| TZ4_Always_quiet_1 | Classification |  |  | *P=*0.16 | 0:49594(99.6%) 1:204(0.4%) | 0:58386(99.6%) 1:263(0.4%) |
| TZ4_Always_quiet_2 | Classification |  |  | *P=*0.48 | 0:15938(32.0%) 1:33860(68.0%) | 0:20327(34.7%) 1:38322(65.3%) |
| TZ4_Always_quiet_3 | Classification |  |  | *P=*0.29 | 0:38416(77.1%) 1:11382(22.9%) | 0:44201(75.4%) 1:14448(24.6%) |
| TZ4_Always_quiet_4 | Classification |  |  | *P=*0.86 | 0:46245(92.9%) 1:3553(7.1%) | 0:54014(92.1%) 1:4635(7.9%) |
| TZ4_Always_quiet_5 | Classification |  |  | *P=*0.37 | 0:48998(98.4%) 1:800(1.6%) | 0: 57,665 cases (98.3%) 1: 984 cases (1.7%) |
| TZ5_Speak_in_a_low_voice_1 | Classification |  |  | *P<*.001 | 0: 49,595 cases (99.6%) 1: 203 cases (0.4%) | 0: 58,389 cases (99.6%) 1: 260 cases (0.4%) |
| TZ5_Speak_in_a_low_voice_2 | Classification |  |  | *P<*.001 | 0: 31,443 cases (63.1%) 1: 18,355 cases (36.9%) | 0: 37,903 cases (64.6%) 1: 20,746 cases (35.4%) |
| TZ5_Speak_in_a_low_voice_3 | Classification |  |  | *P<*.001 | 0: 36,207 cases (72.7%) 1: 13,591 cases (27.3%) | 0: 42,601 cases (72.6%) 1: 16,048 cases (27.4%) |
| TZ5_Speak_in_a_low_voice_4 | Classification |  |  | *P=*0.15 | 0: 36,818 cases (73.9%) 1: 12,980 cases (26.1%) | 0: 42,846 cases (73.1%) 1: 15,803 cases (26.9%) |
| TZ5_Speak_in_a_low_voice_5 | Classification |  |  | *P=*0.07 | 0: 45,660 cases (91.7%) 1: 4,138 cases (8.3%) | Class 0: 53,561 (91.3%) Class 1: 5,088 (8.7%) |
| TZ6_Not_talkative_1 | Classification |  |  | *P<*.001 | Class 0: 49,064 (98.5%) Class 1: 734 (1.5%) | Class 0: 57,685 (98.4%) Class 1: 964 (1.6%) |
| TZ6_Not_talkative_2 | Classification |  |  | *P<*.001 | Class 0: 24,467 (49.1%) Class 1: 25,331 (50.9%) | Class 0: 30,135 (51.4%) Class 1: 28,514 (48.6%) |
| TZ6_Not_talkative_3 | Classification |  |  | *P<*.001 | Class 0: 35,809 (71.9%) Class 1: 13,989 (28.1%) | Class 0: 42,252 (72.0%) Class 1: 16,397 (28.0%) |
| TZ6_Not_talkative_4 | Classification |  |  | *P=*0.01 | Class 0: 42,230 (84.8%) Class 1: 7,568 (15.2%) | Class 0: 48,974 (83.5%) Class 1: 9,675 (16.5%) |
| TZ6_Not_talkative_5 | Classification |  |  | *P=*0.002 | Class 0: 47,386 (95.2%) Class 1: 2,412 (4.8%) | 0:55358(94.4%) 1:3291(5.6%) |
| TZ7_Little_activity_makes_  sweatingeasily_1 | Classification |  |  | *P<*.001 | 0:49300(99.0%) 1:498(1.0%) | 0:57877(98.7%) 1:772(1.3%) |
| TZ7_Little_activity_makes_  sweatingeasily_2 | Classification |  |  | *P=*0.15 | 0:7336(14.7%) 1:42462(85.3%) | 0:8997(15.3%) 1:49652(84.7%) |
| TZ7_Little_activity_makes_  sweatingeasily_3 | Classification |  |  | *P=*0.28 | 0:44505(89.4%) 1:5293(10.6%) | 0:52195(89.0%) 1:6454(11.0%) |
| TZ7_Little_activity_makes_  sweatingeasily_4 | Classification |  |  | *P<*.001 | 0:48134(96.7%) 1:1664(3.3%) | 0:56613(96.5%) 1:2036(3.5%) |
| TZ7_Little_activity_makes  _sweatingeasily_5 | Classification |  |  | *P<*.001 | 0:49492(99.4%) 1:306(0.6%) | Class 0: 58,253 (99.3%) Class 1: 396 (0.7%) |
| TZ8_Hands_and_feet_  always_lukewarm_1 | Classification |  |  | *P<*.001 | 0:49,725 (99.9%) 1:73 (0.1%) | Class 0: 58,538 (99.8%) Class 1: 111 (0.2%) |
| TZ8_Hands_and_feet_  always_lukewarm_2 | Classification |  |  | *P<*.001 | Class 0: 12,036 (24.2%) Class 1: 37,762 (75.8%) | Class 0: 14,693 (25.1%) Class 1: 43,956 (74.9%) |
| TZ8_Hands_and_feet_  always_lukewarm_3 | Classification |  |  | *P<*.001 | Class 0: 42,284 (84.9%) Class 1: 7,514 (15.1%) | Class 0: 49,524 (84.4%) Class 1: 9,125 (15.6%) |
| TZ8_Hands_and_feet_  always_lukewarm_4 | Classification |  |  | *P=*0.06 | Class 0: 46,712 (93.8%) Class 1: 3,086 (6.2%) | Class 0: 54,827 (93.5%) Class 1: 3,822 (6.5%) |
| TZ8_Hands_and_feet_  always_lukewarm_5 | Classification |  |  | *P<*.001 | Class 0: 48,704 (97.8%) Class 1: 1,094 (2.2%) | Class 0: 57,347 (97.8%) Class 1: 1,302 (2.2%) |
| TZ9_Resistant_to_summer_  but_not_to_winter_1 | Classification |  |  | *P<*.001 | Class 0: 49,456 (99.3%) Class 1: 342 (0.7%) | Class 0: 58,205 (99.2%) Class 1: 444 (0.8%) |
| TZ9_Resistant_to_summer_  but_not_to_winter_2 | Classification |  |  | *P<*.001 | Class 0: 7,341 (14.7%) Class 1: 42,457 (85.3%) | Class 0: 9,426 (16.1%) Class 1: 49,223 (83.9%) |
| TZ9_Resistant_to_summer_  but_not_to_winter_3 | Classification |  |  | *P<*.001 | Class 0: 43,746 (87.8%) Class 1: 6,052 (12.2%) | Class 0: 51,009 (87.0%) Class 1: 7,640 (13.0%) |
| TZ9_Resistant_to_summer_  but_not_to_winter_4 | Classification |  |  | *P=*0.002 | Class 0: 48,661 (97.7%) Class 1: 1,137 (2.3%) | Class 0: 57,046 (97.3%) Class 1: 1,603 (2.7%) |
| TZ9_Resistant_to_summer_  but_not_to_winter_5 | Classification |  |  | *P=*0.02 | Class 0: 49,682 (99.8%) Class 1: 116 (0.2%) | Class 0: 58,504 (99.8%) Class 1: 145 (0.2%) |
| TZ10_Afraid_of_cold_than_  other_children_of_  the_same_age_1 | Classification |  |  | *P<*.001 | Class 0: 49,762 (99.9%) Class 1: 36 (0.1%) | Class 0: 58,611 (99.9%) Class 1: 38 (0.1%) |
| TZ10_Afraid_of_cold_than_  other_children_of  _the_same_age_2 | Classification |  |  | *P<*.001 | 0:29340(58.9%) 1:20458(41.1%) | 0:36010(61.4%) 1:22639(38.6%) |
| TZ10_Afraid_of_cold_than_  other_children_of_  the_same_age_3 | Classification |  |  | *P<*.001 | 0:34654(69.6%) 1:15144(30.4%) | 0:40489(69.0%) 1:18160(31.0%) |
| TZ10_Afraid_of_cold_than_  other_children_of  _the_same_age_4 | Classification |  |  | *P=.*02 | 0:39858(80.0%) 1:9940(20.0%) | 0:46290(78.9%) 1:12359(21.1%) |
| TZ10_Afraid_of_cold_than_  other_children_of_  the_same_age_5 | Classification |  |  | *P=.*03 | 0:46395(93.2%) 1:3403(6.8%) | 0:54301(92.6%) 1:4348(7.4%) |
| TZ11_Feel_uncomfortable_  or_afraid_of_eating(drinking)  cold_food_1 | Classification |  |  | *P<*.001 | 0:48945(98.3%) 1:853(1.7%) | 0:57506(98.1%) 1:1143(1.9%) |
| TZ11_Feel_uncomfortable_  or_afraid_of_eating_cold_food_2 | Classification |  |  | *P<*.001 | 0:16856(33.8%) 1:32942(66.2%) | 0:21210(36.2%) 1:37439(63.8%) |
| TZ11_Feel_uncomfortable_  or_afraid_of_eating_cold_food_3 | Classification |  |  | *P<*.001 | 0:37763(75.8%) 1:12035(24.2%) | 0:43518(74.2%) 1:15131(25.8%) |
| TZ11_Feel_uncomfortable_  or_afraid_of_eating_cold_food_4 | Classification |  |  | *P=.*06 | 0:45880(92.1%) 1:3918(7.9%) | 0:53778(91.7%) 1:4871(8.3%) |
| TZ11_Feel_uncomfortable_  or_afraid_of_eating_cold_food_5 | Classification |  |  | *P=.*39 | 0:49063(98.5%) 1:735(1.5%) | 0:57745(98.5%) 1:904(1.5%) |
| TZ12_Having_cold_or_eating_  cold_food_makes_it_easy_to  _have_diarrhea_1 | Classification |  |  | *P<*.001 | 0:49630(99.7%) 1:168(0.3%) | 0:58345(99.5%) 1:304(0.5%) |
| TZ12_Having_cold_or_eating_  cold_food_makes_it_easy_to  _have_diarrhea_2 | Classification |  |  | *P<*.001 | 0:18847(37.8%) 1:30951(62.2%) | 0:24021(41.0%) 1:34628(59.0%) |
| TZ12_Having_cold_or_eating_  cold_food_makes_it_easy_to  _have_diarrhea_3 | Classification |  |  | *P<*.001 | 0:35919(72.1%) 1:13879(27.9%) | 0:41199(70.2%) 1:17450(29.8%) |
| TZ12_Having_cold_or_eating_  cold_food_makes_it_easy_to  _have_diarrhea_4 | Classification |  |  | *P<*.001 | 0:45637(91.6%) 1:4161(8.4%) | 0:53096(90.5%) 1:5553(9.5%) |
| TZ12_Having_cold_or_eating_  cold_food_makes_it_easy_to  _have_diarrhea_5 | Classification |  |  | *P=.*54 | 0:49127(98.7%) 1:671(1.3%) | 0:57832(98.6%) 1:817(1.4%) |
| TZ13_The_body_and_face_are  _always_very_hot_1 | Classification |  |  | *P<*.001 | 0:49662(99.7%) 1:136(0.3%) | 0:58448(99.7%) 1:201(0.3%) |
| TZ13_The_body_and_face_are  _always_very_hot_2 | Classification |  |  | *P<*.001 | 0:16861(33.9%) 1:32937(66.1%) | 0:21757(37.1%) 1:36892(62.9%) |
| TZ13_The_body_and_face_are  _always_very_hot_3 | Classification |  |  | *P<*.001 | 0:37187(74.7%) 1:12611(25.3%) | 0:42564(72.6%) 1:16085(27.4%) |
| TZ13_The_body_and_face_are  _always_very_hot_4 | Classification |  |  | *P=.*23 | 0:46209(92.8%) 1:3589(7.2%) | 0:53874(91.9%) 1:4775(8.1%) |
| TZ13_The_body_and_face_are  _always_very_hot_5 | Classification |  |  | *P=.*35 | 0:49241(98.9%) 1:557(1.1%) | 0:57896(98.7%) 1:753(1.3%) |
| TZ14_Skin_and_lips_  dry_easily_1 | Classification |  |  | *P<*.001 | 0:49694(99.8%) 1:104(0.2%) | 0:58505(99.8%) 1:144(0.2%) |
| TZ14_Skin_and_lips  _dry_easily_2 | Classification |  |  | *P<*.001 | 0:14788(29.7%) 1:35010(70.3%) | 0:18925(32.3%) 1:39724(67.7%) |
| TZ14_Skin_and_lips  _dry_easily_3 | Classification |  |  | *P<*.001 | 0:40293(80.9%) 1:9505(19.1%) | 0:46243(78.8%) 1:12406(21.2%) |
| TZ14_Skin_and_lips  _dry_easily_4 | Classification |  |  | *P=.*37 | 0:46018(92.4%) 1:3780(7.6%) | 0:54050(92.2%) 1:4599(7.8%) |
| TZ14_Skin_and_lips  _dry_easily_5 | Classification |  |  | *P=.*39 | 0:48673(97.7%) 1:1125(2.3%) | 0:57250(97.6%) 1:1399(2.4%) |
| TZ15_Always_excited_1 | Classification |  |  | *P<*.001 | 0:49420(99.2%) 1:378(0.8%) | 0:58128(99.1%) 1:521(0.9%) |
| TZ15_Always_excited_2 | Classification |  |  | *P=.*80 | 0:18047(36.2%) 1:31751(63.8%) | 0:23003(39.2%) 1:35646(60.8%) |
| TZ15_Always_excited_3 | Classification |  |  | *P=.*001 | 0:37872(76.1%) 1:11926(23.9%) | 0:43799(74.7%) 1:14850(25.3%) |
| TZ15_Always_excited_4 | Classification |  |  | *P=.*03 | 0:44881(90.1%) 1:4917(9.9%) | 0:52,225 (89.0%) 1:6,424 (11.0%) |
| TZ15_Always_excited_5 | Classification |  |  | *P=.*03 | 0:48,831 (98.1%) 1:967 (1.9%) | 0:57,300 (97.7%) 1:1,349 (2.3%) |
| TZ16_Easily_restless_1 | Classification |  |  | *P<*.001 | 0:49,561 (99.5%) 1:237 (0.5%) | 0:58,269 (99.4%) 1:380 (0.6%) |
| TZ16_Easily_restless_2 | Classification |  |  | *P=.*63 | 0:17,019 (34.2%) 1:32,779 (65.8%) | 0:21,741 (37.1%) 1:36,908 (62.9%) |
| TZ16_Easily_restless_3 | Classification |  |  | *P<*.001 | 0:37,155 (74.6%) 1:12,643 (25.4%) | 0:42,750 (72.9%) 1:15,899 (27.1%) |
| TZ16_Easily_restless_4 | Classification |  |  | *P<*.001 | 0:46,207 (92.8%) 1:3,591 (7.2%) | 0:53,853 (91.8%) 1:4,796 (8.2%) |
| TZ16_Easily_restless_5 | Classification |  |  | *P<*.001 | Class 0: 49,163 (98.7%) Class 1: 635 (1.3%) | Class 0: 57,829 (98.6%) Class 1: 820 (1.4%) |
| TZ17_Being_overweight_and  _unwilling_to_exercise_1 | Classification |  |  | *P=.*01 | Class 0: 49,648 (99.7%) Class 1: 150 (0.3%) | 0:58423(99.6%) 1:226(0.4%) |
| TZ17_Being_overweight_and  _unwilling_to_exercise_2 | Classification |  |  | *P=.*05 | Class 0: 9,565 (19.2%) Class 1: 40,233 (80.8%) | Class 0: 12,424 (21.2%) Class 1: 46,225 (78.8%) |
| TZ17_Being_overweight_and  _unwilling_to_exercise_3 | Classification |  |  | *P=.*25 | Class 0: 42,739 (85.8%) Class 1: 7,059 (14.2%) | Class 0: 49,496 (84.4%) Class 1: 9,153 (15.6%) |
| TZ17_Being_overweight_and  _unwilling_to_exercise_4 | Classification |  |  | *P=.*23 | Class 0: 47,797 (96.0%) Class 1: 2,001 (4.0%) | Class 0: 56,016 (95.5%) Class 1: 2,633 (4.5%) |
| TZ17_Being_overweight_and  _unwilling_to_exercise_5 | Classification |  |  | *P=.*10 | Class 0: 49,390 (99.2%) Class 1: 408 (0.8%) | Class 0: 58,125 (99.1%) Class 1: 524 (0.9%) |
| TZ18_Abdominal_obesity_is_  flabby_and_soft_1 | Classification |  |  | *P<*.001 | 0:49701(99.8%) 1:97(0.2%) | 0:58535(99.8%) 1:114(0.2%) |
| TZ18_Abdominal_obesity_is_  flabby_and_soft_2 | Classification |  |  | *P=.*03 | 0:7815(15.7%) 1:41983(84.3%) | 0:10110(17.2%) 1:48539(82.8%) |
| TZ18_Abdominal_obesity_is_  flabby_and_soft_3 | Classification |  |  | *P=.*03 | 0:43549(87.5%) 1:6249(12.5%) | 0:50588(86.3%) 1:8061(13.7%) |
| TZ18_Abdominal_obesity_is_  flabby_and_soft_4 | Classification |  |  | *P=.*81 | 0:48434(97.3%) 1:1364(2.7%) | 0:56813(96.9%) 1:1836(3.1%) |
| TZ18_Abdominal_obesity_is_  flabby_and_soft_5 | Classification |  |  | *P=.*19 | 0:49636(99.7%) 1:162(0.3%) | 0:58475(99.7%) 1:174(0.3%) |
| TZ19_Always_sleepy_1 | Classification |  |  | *P<*.001 | 0:49758(99.9%) 1:40(0.1%) | 0: 58,610 (99.9%) 1: 39 (0.1%) |
| TZ19_Always_sleepy_2 | Classification |  |  | *P<*.001 | 0: 8,419 (16.9%) 1: 41,379 (83.1%) | 0: 10,889 (18.6%) 1: 47,760 (81.4%) |
| TZ19_Always_sleepy_3 | Classification |  |  | *P<*.001 | Class 0: 43,746 (87.8%) Class 1: 6,052 (12.2%) | 0: 50,774 (86.6%) 1: 7,875 (13.4%) |
| TZ19_Always_sleepy_4 | Classification |  |  | *P=.*68 | 0: 48,172 (96.7%) 1: 1,626 (3.3%) | 0: 56,568 (96.5%) 1: 2,081 (3.5%) |
| TZ19_Always_sleepy_5 | Classification |  |  | *P=.*72 | 0: 49,267 (98.9%) 1: 531 (1.1%) | 0: 57,999 (98.9%) 1: 650 (1.1%) |
| TZ20_Like_to_eat_sweet_or  _greasy_things_1 | Classification |  |  | *P<*.001 | 0: 49,588 (99.6%) 1: 210 (0.4%) | 0: 58,366 (99.5%) 1: 283 (0.5%) |
| TZ20_Like_to_eat_sweet_or  _greasy_things_2 | Classification |  |  | *P=.*05 | 0: 15,760 (31.6%) 1: 34,038 (68.4%) | 0:19779(33.7%) 1:38870(66.3%) |
| TZ20_Like_to_eat_sweet_or  _greasy_things_3 | Classification |  |  | *P<*.001 | 0:39048(78.4%) 1:10750(21.6%) | 0:45361(77.3%) 1:13288(22.7%) |
| TZ20_Like_to_eat_sweet_or  _greasy_things_4 | Classification |  |  | *P<*.001 | 0:46337(93.0%) 1:3461(7.0%) | 0:54180(92.4%) 1:4469(7.6%) |
| TZ20_Like_to_eat_sweet_or  _greasy_things_5 | Classification |  |  | *P=.*004 | 0:48637(97.7%) 1:1161(2.3%) | 0:57157(97.5%) 1:1492(2.5%) |
| TZ21_Snoring_at_night_1 | Classification |  |  | *P<*.001 | 0:49410(99.2%) 1:388(0.8%) | 0:58119(99.1%) 1:530(0.9%) |
| TZ21_Snoring_at_night_2 | Classification |  |  | *P<*.001 | 0:8284(16.6%) 1:41514(83.4%) | 0:10792(18.4%) 1:47857(81.6%) |
| TZ21_Snoring_at_night_3 | Classification |  |  | *P=.*009 | 0:43685(87.7%) 1:6113(12.3%) | 0:50698(86.4%) 1:7951(13.6%) |
| TZ21_Snoring_at_night_4 | Classification |  |  | *P=.*39 | 0:48137(96.7%) 1:1661(3.3%) | 0:56502(96.3%) 1:2147(3.7%) |
| TZ21_Snoring_at_night_5 | Classification |  |  | *P<*.001 | 0:49417(99.2%) 1:381(0.8%) | 0:58154(99.2%) 1:495(0.8%) |
| TZ22_Thick_and_greasy_  tongue_coating_1 | Classification |  |  | *P<*.001 | 0:49669(99.7%) 1:129(0.3%) | 0:58450(99.7%) 1:199(0.3%) |
| TZ22_Thick_and_greasy_  tongue_coating_2 | Classification |  |  | *P<*.001 | 0:4024(8.1%) 1:45774(91.9%) | 0:5190(8.8%) 1:53459(91.2%) |
| TZ22_Thick_and_greasy_  tongue_coating_3 | Classification |  |  | *P<*.001 | 0:46809(94.0%) 1:2989(6.0%) | 0:54800(93.4%) 1:3849(6.6%) |
| TZ22_Thick_and_greasy_  tongue_coating_4 | Classification |  |  | *P=.*54 | 0:49046(98.5%) 1:752(1.5%) | 0:57676(98.3%) 1:973(1.7%) |
| TZ22_Thick_and_greasy_  tongue_coating_5 | Classification |  |  | *P=.*05 | 0: 49,588 (99.6%) 1: 210 (0.4%) | 0: 58,389 cases (99.6%) 1: 260 cases (0.4%) |
| TZ23_Always_red_eyes_or_  easy_to_get_gum_1 | Classification |  |  | *P<*.001 | 0:49,725 (99.9%) 1:73 (0.1%) | 0:58541(99.8%) 1:108(0.2%) |
| TZ23_Always_red_eyes_or_  easy_to_get_gum_2 | Classification |  |  | *P<*.001 | 0:4244(8.5%) 1:45554(91.5%) | 0:5608(9.6%) 1:53041(90.4%) |
| TZ23_Always_red_eyes_or_  easy_to_get_gum_3 | Classification |  |  | *P<*.001 | 0:46401(93.2%) 1:3397(6.8%) | 0:54204(92.4%) 1:4445(7.6%) |
| TZ23_Always_red_eyes_or_  easy_to_get_gum_4 | Classification |  |  | *P=.*01 | 0:49114(98.6%)1:684(1.4%) | 0:57723(98.4%) 1:926(1.6%) |
| TZ23_Always_red_eyes_or_  easy_to_get_gum_5 | Classification |  |  | *P=.*23 | 0:49680(99.8%) 1:118(0.2%) | 0:58,480 (99.7%) 1:169 (0.3%) |
| TZ24_Eczema_is_easy_to_grow  _on_the_body_1 | Classification |  |  | *P<*.001 | 0:49,753 (99.9%) 1:45 (0.1%) | 0:58,581 (99.9%) 1:68 (0.1%) |
| TZ24_Eczema_is_easy_to_grow  _on_the_body_2 | Classification |  |  | *P<*.001 | 0:8,022 (16.1%) 1:41,776 (83.9%) | 0:10,834 (18.5%) 1:47,815 (81.5%) |
| TZ24_Eczema_is_easy_to_grow  _on_the_body_3 | Classification |  |  | *P=.*13 | 0:43,004 (86.4%) 1:6,794 (13.6%) | 0:49,581 (84.5%) 1:9,068 (15.5%) |
| TZ24_Eczema_is_easy_to_grow  _on_the_body_4 | Classification |  |  | *P=.*18 | 0:48,666 (97.7%) 1:1,132 (2.3%) | 0:57,014 (97.2%) 1:1,635 (2.8%) |
| TZ24_Eczema_is_easy_to_grow  _on_the_body_5 | Classification |  |  | *P=.*02 | 0:49,720 (99.8%) 1:78 (0.2%) | 0:58,543 (99.8%) 1:106 (0.2%) |
| TZ25_Like_to_eat_spicy_or_fried  _barbecue_food_1 | Classification |  |  | *P<*.001 | 0:49780(100.0%) 1:18(0.0%) | 0:58624(100.0%) 1:25(0.0%) |
| TZ25_Like_to_eat_spicy_or_fried  _barbecue_food_2 | Classification |  |  | *P<*.001 | 0:12751(25.6%) 1:37047(74.4%) | 0:16521(28.2%) 1:42128(71.8%) |
| TZ25_Like_to_eat_spicy_or_fried  _barbecue_food_3 | Classification |  |  | *P<*.001 | 0:40004(80.3%) 1:9794(19.7%) | 0:46241(78.8%) 1:12408(21.2%) |
| TZ25_Like_to_eat_spicy_or_fried  _barbecue_food_4 | Classification |  |  | *P<*.001 | 0:47202(94.8%) 1:2596(5.2%) | 0:55087(93.9%) 1:3562(6.1%) |
| TZ25_Like_to_eat_spicy_or_fried  _barbecue_food_5 | Classification |  |  | *P<*.001 | 0:49493(99.4%) 1:305(0.6%) | 0:58186(99.2%) 1:463(0.8%) |
| TZ26_Sweat_is_sticky_1 | Classification |  |  | *P<*.001 | 0:49742(99.9%) 1:56(0.1%) | 0:58561(99.8%) 1:88(0.2%) |
| TZ26_Sweat_is_sticky_2 | Classification |  |  | *P<*.001 | 0:22224(44.6%) 1:27574(55.4%) | 0:27889(47.6%) 1:30760(52.4%) |
| TZ26_Sweat_is_sticky_3 | Classification |  |  | *P<*.001 | 0:34542(69.4%) 1:15256(30.6%) | 0:40015(68.2%) 1:18634(31.8%) |
| TZ26_Sweat_is_sticky_4 | Classification |  |  | *P=.*08 | 0:44006(88.4%) 1:5792(11.6%) | 0:51065(87.1%) 1:7584(12.9%) |
| TZ26_Sweat_is_sticky_5 | Classification |  |  | *P=.*02 | 0:48817(98.0%) 1:981(2.0%) | 0:57286(97.7%) 1:1363(2.3%) |
| TZ27_The_skin_always_appears_  blue_and_purple_ecchymosis  _unconsciously_1 | Classification |  |  | *P<*.001 | 0:49603(99.6%) 1:195(0.4%) | 0:58341(99.5%) 1:308(0.5%) |
| TZ27_The_skin_always_appears_  blue_and_purple_ecchymosis  _unconsciously_2 | Classification |  |  | *P<*.001 | 0:21569(43.3%) 1:28229(56.7%) | 0:27377(46.7%) 1:31272(53.3%) |
| TZ27_The_skin_always_appears_  blue_and_purple_ecchymosis  _unconsciously_3 | Classification |  |  | *P<*.001 | 0:35010(70.3%) 1:14788(29.7%) | 0:40272(68.7%) 1:18377(31.3%) |
| TZ27_The_skin_always_appears_  blue_and_purple_ecchymosis  _unconsciously_4 | Classification |  |  | *P=.*20 | 0:44215(88.8%) 1:5583(11.2%) | 0:51290(87.5%) 1:7359(12.5%) |
| TZ27_The_skin_always_appears_  blue_and_purple_ecchymosis  _unconsciously_5 | Classification |  |  | *P=*1 | 0:48789(98.0%) 1:1009(2.0%) | 0:57348(97.8%) 1:1301(2.2%) |
| TZ28_Where_there_is_pain  _on_the_body_1 | Classification |  |  | *P<*.001 | 0:49609(99.6%) 1:189(0.4%) | 0:58309(99.4%) 1:340(0.6%) |
| TZ28_Where_there_is_pain  _on_the_body_2 | Classification |  |  | *P<*.001 | 0:29423(59.1%) 1:20375(40.9%) | 0:36304(61.9%) 1:22345(38.1%) |
| TZ28_Where_there_is_pain  _on_the_body_3 | Classification |  |  | *P<*.001 | 0:32065(64.4%) 1:17733(35.6%) | 0:37393(63.8%) 1:21256(36.2%) |
| TZ28_Where_there_is_pain  _on_the_body_4 | Classification |  |  | *P=.*43 | 0:41214(82.8%) 1:8584(17.2%) | 0:47949(81.8%) 1:10700(18.2%) |
| TZ28_Where_there_is_pain  _on_the_body_5 | Classification |  |  | *P=.*47 | 0:47314(95.0%) 1:2484(5.0%) | 0:55267(94.2%) 1:3382(5.8%) |
| TZ29_The_skin_on_the_body_  or_the_skin_on_the_neck_armpit  _or_thigh_root_is_dark_1 | Classification |  |  | *P<*.001 | 0:49176(98.8%) 1:622(1.2%) | 0:57683(98.4%) 1:966(1.6%) |
| TZ29_The_skin_on_the_body_  or_the_skin_on_the_neck_armpit  _or_thigh_root_is_dark_2 | Classification |  |  | *P<*.001 | 0:28897(58.0%) 1:20901(42.0%) | 0:35356(60.3%) 1:23293(39.7%) |
| TZ29_The_skin_on_the_body_  or_the_skin_on_the_neck_armpit  _or_thigh_root_is_dark_3 | Classification |  |  | *P=.*01 | 0:30876(62.0%) 1:18922(38.0%) | 0:36359(62.0%) 1:22290(38.0%) |
| TZ29_The_skin_on_the_body_or_  the_skin_on_the_neck_armpit_  or_thigh_root_is_dark_4 | Classification |  |  | *P=.*53 | 0:41797(83.9%) 1:8001(16.1%) | 0:48359(82.5%) 1:10290(17.5%) |
| TZ29_The_skin_on_the_body_or  _the_skin_on_the_neck,armpit_  or_thigh_root_is_dark_5 | Classification |  |  | *P=.*15 | 0:48159(96.7%) 1:1639(3.3%) | 0:56439(96.2%) 1:2210(3.8%) |
| TZ30_Be_prone_to_dark_circles_1 | Classification |  |  | *P<*.001 | 0:49463(99.3%) 1:335(0.7%) | 0:58083(99.0%) 1:566(1.0%) |
| TZ30_Be_prone_to_dark_circles_2 | Classification |  |  | *P<*.001 | 0:17483(35.1%) 1:32315(64.9%) | 0:21921(37.4%) 1:36728(62.6%) |
| TZ30_Be_prone_to_dark_circles_3 | Classification |  |  | *P<*.001 | 0:45765(91.9%) 1:4033(8.1%) | 0:49,666 (84.7%) 1:8,983 (15.3%) |
| TZ30_Be_prone_to_dark_circles_4 | Classification |  |  | *P=.*03 | 0:45,765 (91.9%) 1:4,033 (8.1%) | 0:53,506 (91.2%) 1:5,143 (8.8%) |
| TZ30_Be_prone_to_dark_circles_5 | Classification |  |  | *P=.*03 | 0:49,040 (98.5%) 1:758 (1.5%) | 0:57,652 (98.3%) 1:997 (1.7%) |
| TZ31_Blue_or_dark_gums_1 | Classification |  |  | *P<*.001 | 0:49,621 (99.6%) 1:177 (0.4%) | 0:58,375 (99.5%) 1:274 (0.5%) |
| TZ31_Blue_or_dark_gums_2 | Classification |  |  | *P<*.001 | 0:24,206 (48.6%) 1:25,592 (51.4%) | 0:29,935 (51.0%) 1:28,714 (49.0%) |
| TZ31_Blue_or_dark_gums_3 | Classification |  |  | *P=.*04 | 0:35,120 (70.5%) 1:14,678 (29.5%) | 0:40,909 (69.8%) 1:17,740 (30.2%) |
| TZ31_Blue_or_dark_gums_4 | Classification |  |  | *P=.*16 | 0:42416(85.2%) 1:7382(14.8%) | 0:49397(84.2%) 1:9252(15.8%) |
| TZ31_Blue_or_dark_gums_5 | Classification |  |  | *P=.*02 | 0:48088(96.6%) 1:1710(3.4%) | 0:56324(96.0%) 1:2325(4.0%) |
| TZ32_Rough_skin_like_fish_scales_1 | Classification |  |  | *P<*.001 | 0:49362(99.1%) 1:436(0.9%) | 0:58031(98.9%) 1:618(1.1%) |
| TZ32_Rough_skin_like_fish_scales_2 | Classification |  |  | *P<*.001 | 0:19987(40.1%) 1:29811(59.9%) | 0:24740(42.2%) 1:33909(57.8%) |
| TZ32_Rough_skin_like_fish_scales_3 | Classification |  |  | *P=.*05 | 0:34878(70.0%) 1:14920(30.0%) | 0:40283(68.7%) 1:18366(31.3%) |
| TZ32_Rough_skin_like_fish_scales_4 | Classification |  |  | *P=.*62 | 0:45666(91.7%) 1:4132(8.3%) | 0:53485(91.2%) 1:5164(8.8%) |
| TZ32_Rough_skin_like_fish_scales_5 | Classification |  |  | *P=.*15 | 0:49069(98.5%) 1:729(1.5%) | 0:57729(98.4%) 1:920(1.6%) |
| TZ33_Lips_are_dark_or_blue_1 | Classification |  |  | *P<*.001 | 0:49592(99.6%) 1:206(0.4%) | 0:58359(99.5%) 1:290(0.5%) |
| TZ33_Lips_are_dark_or_blue_2 | Classification |  |  | *P<*.001 | 0:14355(28.8%) 1:35443(71.2%) | 0:18253(31.1%) 1:40396(68.9%) |
| TZ33_Lips_are_dark_or_blue_3 | Classification |  |  | *P=.*006 | 0:40075(80.5%) 1:9723(19.5%) | 0:46479(79.2%) 1:12170(20.8%) |
| TZ33_Lips_are_dark_or_blue_4 | Classification |  |  | *P=.*12 | 0:46140(92.7%) 1:3658(7.3%) | 0:53856(91.8%) 1:4793(8.2%) |
| TZ33_Lips_are_dark_or_blue_5 | Classification |  |  | *P=.*23 | 0:49,040 (98.5%) 1:758 (1.5%) | 0:57647(98.3%) 1:1002(1.7%) |
| TZ34_Always_moping_1 | Classification |  |  | *P<*.001 | 0:49582(99.6%) 1:216(0.4%) | 0:58361(99.5%) 1:288(0.5%) |
| TZ34_Always_moping_2 | Classification |  |  | *P<*.001 | 0:9081(18.2%) 1:40717(81.8%) | 0:12120(20.7%) 1:46529(79.3%) |
| TZ34_Always_moping_3 | Classification |  |  | *P<*.001 | 0:42931(86.2%) 1:6867(13.8%) | 0:49593(84.6%) 1:9056(15.4%) |
| TZ34_Always_moping_4 | Classification |  |  | *P=.*38 | 0:47994(96.4%) 1:1804(3.6%) | 0:56147(95.7%) 1:2502(4.3%) |
| TZ34_Always_moping_5 | Classification |  |  | *P=.*70 | 0:49468(99.3%) 1:330(0.7%) | 0:58201(99.2%) 1:448(0.8%) |
| TZ35_Easily_nervous_1 | Classification |  |  | *P<*.001 | 0:49718(99.8%) 1:80(0.2%) | 0:58535(99.8%) 1:114(0.2%) |
| TZ35_Easily_nervous_2 | Classification |  |  | *P<*.001 | 0:10089(20.3%) 1:39709(79.7%) | 0:13086(22.3%) 1:45563(77.7%) |
| TZ35_Easily_nervous_3 | Classification |  |  | *P<*.001 | 0:43154(86.7%) 1:6644(13.3%) | 0:50010(85.3%) 1:8639(14.7%) |
| TZ35_Easily_nervous_4 | Classification |  |  | *P<*.001 | 0:47324(95.0%) 1:2474(5.0%) | 0:55501(94.6%) 1:3148(5.4%) |
| TZ35_Easily_nervous_5 | Classification |  |  | *P=.*11 | 0:49100(98.6%) 1:698(1.4%) | 0:57717(98.4%) 1:932(1.6%) |
| TZ36_Easy_to_lack_self_  confidence_1 | Classification |  |  | *P<*.001 | 0:49525(99.5%) 1:273(0.5%) | 0:58282(99.4%) 1:367(0.6%) |
| TZ36_Easy_to_lack_self_  confidence_2 | Classification |  |  | *P<*.001 | 0:13059(26.2%) 1:36739(73.8%) | 0:16629(28.4%) 1:42020(71.6%) |
| TZ36_Easy_to_lack_self_  confidence_3 | Classification |  |  | *P<*.001 | 0:41306(82.9%) 1:8492(17.1%) | 0:47930(81.7%) 1:10719(18.3%) |
| TZ36_Easy_to_lack_self_  confidence_4 | Classification |  |  | *P<*.001 | 0:46552(93.5%) 1:3246(6.5%) | 0:54544(93.0%) 1:4105(7.0%) |
| TZ36_Easy_to_lack_self_  confidence_5 | Classification |  |  | *P=.*002 | 0:48806(98.0%) 1:992(2.0%) | 0:57345(97.8%) 1:1304(2.2%) |
| TZ37_Easily_afraid_or_frightened_1 | Classification |  |  | *P<*.001 | 0:49469(99.3%) 1:329(0.7%) | 0:58148(99.1%) 1:501(0.9%) |
| TZ37_Easily_afraid_or_frightened_2 | Classification |  |  | *P<*.001 | 0:3395(6.8%) 1:46403(93.2%) | 0:4529(7.7%) 1:54120(92.3%) |
| TZ37_Easily_afraid_or_frightened_3 | Classification |  |  | *P<*.001 | 0:47013(94.4%) 1:2785(5.6%) | 0:54987(93.8%) 1:3662(6.2%) |
| TZ37_Easily_afraid_or_frightened_4 | Classification |  |  | *P=.*03 | 0:49293(99.0%) 1:505(1.0%) | 0:57911(98.7%) 1:738(1.3%) |
| TZ37_Easily_afraid_or_frightened_5 | Classification |  |  | *P<*.001 | Class 0: 49,723 (99.8%) Class 1: 75 (0.2%) | 0:58565(99.9%) 1:84(0.1%) |
| TZ38_Always_crying_when  _in_trouble_1 | Classification |  |  | *P<*.001 | 0:49768(99.9%) 1:30(0.1%) | 0:58604(99.9%) 1:45(0.1%) |
| TZ38_Always_crying_when  _in_trouble_2 | Classification |  |  | *P=.*03 | 0:13176(26.5%) 1:36622(73.5%) | 0:17231(29.4%) 1:41418(70.6%) |
| TZ38_Always_crying_when  _in_trouble_3 | Classification |  |  | *P<*.001 | 0:40187(80.7%) 1:9611(19.3%) | 0:46179(78.7%) 1:12470(21.3%) |
| TZ38_Always_crying_when  _in_trouble_4 | Classification |  |  | *P<*.001 | 0:47190(94.8%) 1:2608(5.2%) | 0:55152(94.0%) 1:3497(6.0%) |
| TZ38_Always_crying_when  _in_trouble_5 | Classification |  |  | *P<*.001 | 0:49084(98.6%) 1:714(1.4%) | 0:57765(98.5%) 1:884(1.5%) |
| TZ39_Easily_shy_or_timid_1 | Classification |  |  | *P<*.001 | 0:49555(99.5%) 1:243(0.5%) | 0:58269(99.4%) 1:380(0.6%) |
| TZ39_Easily_shy_or_timid_2 | Classification |  |  | *P=.*98 | 0:38119(76.5%) 1:11679(23.5%) | 0:45150(77.0%) 1:13499(23.0%) |
| TZ39_Easily_shy_or_timid_3 | Classification |  |  | *P<*.001 | 0:43625(87.6%) 1:6173(12.4%) | 0:50808(86.6%) 1:7841(13.4%) |
| TZ39_Easily_shy_or_timid_4 | Classification |  |  | *P<*.001 | Absent: 40,545 (81.4%); Present: 9,253 (18.6%) | Absent: 47,044 (80.2%); Present: 11,605 (19.8%) |
| TZ39_Easily_shy_or_timid_5 | Classification |  |  | *P<*.001 | Absent: 34,773 (69.8%); Present: 15,025 (30.2%) | Absent: 41,282 (70.4%); Present: 17,367 (29.6%) |
| TZ40_Always_worried_about  _a_lot_of_things_1 | Classification |  |  | *P<*.001 | Absent: 42,130 (84.6%); Present: 7,668 (15.4%) | Absent: 50,312 (85.8%); Present: 8,337 (14.2%) |
| TZ40_Always_worried_about  _a_lot_of_things_2 | Classification |  |  | *P<*.001 | Absent: 37,133 (74.6%); Present: 12,665 (25.4%) | Absent: 44,071 (75.1%); Present: 14,578 (24.9%) |
| TZ40_Always_worried_about  _a_lot_of_things_3 | Classification |  |  | *P<*.001 | Absent: 46,063 (92.5%); Present: 3,735 (7.5%) | Absent: 53,436 (91.1%); Present: 5,213 (8.9%) |
| TZ40_Always_worried_about  _a_lot_of_things_4 | Classification |  |  | *P=.*02 | Absent: 43,412 (87.2%); Present: 6,386 (12.8%) | Category 0: 50,085 (85.4%); Category 1: 8,564 (14.6%) |
| TZ40_Always_worried_about  _a_lot_of_things_5 | Classification |  |  | *P=.*005 | Category 0: 34,682 (69.6%); Category 1: 15,116 (30.4%) | Category 0: 41,233 (70.3%); Category 1: 17,416 (29.7%) |
| TZ41_More_attached_to_  you_now_than_before_1 | Classification |  |  | *P<*.001 | Category 0: 37,902 (76.1%); Category 1: 11,896 (23.9%) | Category 0: 45,771 (78.0%); Category 1: 12,878 (22.0%) |
| TZ41_More_attached_to_  you_now_than_before_2 | Classification |  |  | *P=.*006 | Category 0: 16,423 (33.0%); Category 1: 33,375 (67.0%) | Category 0: 20,886 (35.6%); Category 1: 37,763 (64.4%) |
| TZ41_More_attached_to_  you_now_than_before_3 | Classification |  |  | *P<*.001 | Category 0: 36,885 (74.1%); Category 1: 12,913 (25.9%) | Category 0: 42,419 (72.3%); Category 1: 16,230 (27.7%) |
| TZ41_More_attached_to_  you_now_than_before_4 | Classification |  |  | *P<*.001 | Category 0: 46,738 (93.9%); Category 1: 3,060 (6.1%) | Category 0: 54,617 (93.1%); Category 1: 4,032 (6.9%) |
| TZ41_More_attached_to_  you_now_than_before_5 | Classification |  |  | *P=.*003 | 0: 49,439 (99.3%) 1: 359 (0.7%) | 0:58186(99.2%) 1:463(0.8%) |
| TZ42_Sneez_when_do_  not_have_a_cold_1 | Classification |  |  | *P<*.001 | 0: 49,707 (99.8%) 1: 91 (0.2%) | 0: 58,488 (99.7%) 1: 161 (0.3%) |
| TZ42_Sneez_when_do_  not_have_a_cold_2 | Classification |  |  | *P<*.001 | 0: 31,413 (63.1%) 1: 18,385 (36.9%) | 0: 38,235 (65.2%) 1: 20,414 (34.8%) |
| TZ42_Sneez_when_do_  not_have_a_cold_3 | Classification |  |  | *P=.*003 | 0: 25,799 (51.8%) 1: 23,999 (48.2%) | 0: 29,781 (50.8%) 1: 28,868 (49.2%) |
| TZ42_Sneez_when_do_  not_have_a_cold_4 | Classification |  |  | *P=.*17 | 0: 44,143 (88.6%) 1: 5,655 (11.4%) | 0: 51,589 (88.0%) 1: 7,060 (12.0%) |
| TZ42_Sneez_when_do_  not_have_a_cold_5 | Classification |  |  | *P=.*05 | 0: 48,039 (96.5%) 1: 1,759 (3.5%) | 0: 56,342 (96.1%) 1: 2,307 (3.9%) |
| TZ43_Without_cold_but_have  _stuffy_nose_and_runny_nose_1 | Classification |  |  | *P<*.001 | 0:34,667 (69.6%) 1:15,131 (30.4%) | 0:41,667 (71.0%) 1:16,982 (29.0%) |
| TZ43_Without_cold_but_have  _stuffy_nose_and_runny_nose_2 | Classification |  |  | *P<*.001 | 0:27,209 (54.6%) 1:22,589 (45.4%) | 0:32,135 (54.8%) 1:26,514 (45.2%) |
| TZ43_Without_cold_but_have  _stuffy_nose_and_runny_nose_3 | Classification |  |  | *P<*.001 | 0:40,562 (81.5%) 1:9,236 (18.5%) | 0:47,216 (80.5%) 1:11,433 (19.5%) |
| TZ43_Without_cold_but_have  _stuffy_nose_and_runny_nose_4 | Classification |  |  | *P=.*02 | 0:46,956 (94.3%) 1:2,842 (5.7%) | 0:54,929 (93.7%) 1:3,720 (6.3%) |
| TZ43_Without_cold_but_have  _stuffy_nose_and_runny_nose_5 | Classification |  |  | *P=.*18 | 0:26,340 (52.9%) 1:23,458 (47.1%) | 0:32,359 (55.2%) 1:26,290 (44.8%) |
| TZ44_Cough_and_wheeze_when_  the_season_changes_the_temperature  _changes_or_smells_strange_smell_1 | Classification |  |  | *P<*.001 | 0:28,395 (57.0%) 1:21,403 (43.0%) | 0:32609(55.6%) 1:26040(44.4%) |
| TZ44_Cough_and_wheeze_when_  the_season_changes_the_temperature_  changes_or_smells_strange_smell_2 | Classification |  |  | *P<*.001 | 0:45876(92.1%) 1:3922(7.9%) | 0:53699(91.6%) 1:4950(8.4%) |
| TZ44_Cough_and_wheeze_when_  the_season_changes_the_temperature_  changes_or_smells_strange_smell_3 | Classification |  |  | *P<*.001 | 0:48783(98.0%) 1:1015(2.0%) | 0:57280(97.7%) 1:1369(2.3%) |
| TZ44_Cough_and_wheeze_when_  the_season_changes_the_temperature_  changes_or_smells_strange_smell_4 | Classification |  |  | *P=.*05 | 0:17631(35.4%) 1:32167(64.6%) | 0:21844(37.2%) 1:36805(62.8%) |
| TZ44_Cough_and_wheeze_when_  the_season_changes_the_temperature_changes_or_smells_strange_smell_5 | Classification |  |  | *P=.*22 | 0:38214(76.7%) 1:11584(23.3%) | 0:44314(75.6%) 1:14335(24.4%) |
| TZ45_Susceptible_to_allergies_1 | Classification |  |  | *P<*.001 | 0:45880(92.1%) 1:3918(7.9%) | 0:53929(92.0%) 1:4720(8.0%) |
| TZ45_Susceptible_to_allergies_2 | Classification |  |  | *P<*.001 | 0:47669(95.7%) 1:2129(4.3%) | 0:55860(95.2%) 1:2789(4.8%) |
| TZ45_Susceptible_to_allergies_3 | Classification |  |  | *P=.*003 | 0:26481(53.2%) 1:23317(46.8%) | 0:31890(54.4%) 1:26759(45.6%) |
| TZ45_Susceptible_to_allergies_4 | Classification |  |  | *P=.*01 | 0:29632(59.5%) 1:20166(40.5%) | 0:34515(58.9%) 1:24134(41.1%) |
| TZ45_Susceptible_to_allergies_5 | Classification |  |  | *P=.*10 | 0:45127(90.6%) 1:4671(9.4%) | 0:53017(90.4%) 1:5632(9.6%) |
| TZ46_The_skin_is_prone_to_  itching_and_urticaria_1 | Classification |  |  | *P<*.001 | 0:48154(96.7%) 1:1644(3.3%) | 0:56525(96.4%) 1:2124(3.6%) |
| TZ46_The_skin_is_prone_to_  itching_and_urticaria_2 | Classification |  |  | *P<*.001 | 0:30299(60.8%) 1:19499(39.2%) | 0:36,685 (62.6%) and 1:21,964 (37.4%) |
| TZ46_The_skin_is_prone_to_  itching_and_urticaria_3 | Classification |  |  | *P=.*002 | 0:29,655 (59.6%) and 1:20,143 (40.4%) | 0:34,426 (58.7%) and 1:24,223 (41.3%) |
| TZ46_The_skin_is_prone_to_  itching_and_urticaria_4 | Classification |  |  | *P=.*009 | 0:42,133 (84.6%) and 1:7,665 (15.4%) | 0:49,324 (84.1%) and 1:9,325 (15.9%) |
| TZ46_The_skin_is_prone_to_  itching_and_urticaria_5 | Classification |  |  | *P<*.001 | 0:47,307 (95.0%) and 1:2,491 (5.0%) | 0:55,512 (94.7%) and 1:3,137 (5.3%) |
| TZ47_Purpura_has_occurred_  in_the_skin_due_to_allergies_1 | Classification |  |  | *P<*.001 | 0:34,075 (68.4%) and 1:15,723 (31.6%) | 0:41,753 (71.2%) and 1:16,896 (28.8%) |
| TZ47_Purpura_has_occurred_  in_the_skin_due_to_allergies_2 | Classification |  |  | *P<*.001 | 0:26,835 (53.9%) and 1:22,963 (46.1%) | 0:31,776 (54.2%) and 1:26,873 (45.8%) |
| TZ47_Purpura_has_occurred_  in_the_skin_due_to_allergies_3 | Classification |  |  | *P<*.001 | 0:41410(83.2%) 1:8388(16.8%) | 0:47619(81.2%) 1:11030(18.8%) |
| TZ47_Purpura_has_occurred_  in_the_skin_due_to_allergies_4 | Classification |  |  | *P=.*81 | 0:47074(94.5%) 1:2724(5.5%) | 0:54799(93.4%) 1:3850(6.6%) |
| TZ47_Purpura_has_occurred_  in_the_skin_due_to_allergies_5 | Classification |  |  | *P=.*36 | 0:22689(45.6%) 1:27109(54.4%) | 0:28819(49.1%) 1:29830(50.9%) |
| TZ48_The_skin_turns_red_when_  scratched_and_scratches_appear_1 | Classification |  |  | *P<*.001 | 0:30180(60.6%) 1:19618(39.4%) | 0:34087(58.1%) 1:24562(41.9%) |
| TZ48_The_skin_turns_red_when_  scratched_and_scratches_appear_2 | Classification |  |  | *P<*.001 | 0:47359(95.1%) 1:2439(4.9%) | 0:55367(94.4%) 1:3282(5.6%) |
| TZ48_The_skin_turns_red_when_  scratched_and_scratches_appear_3 | Classification |  |  | *P<*.001 | 0:49166(98.7%) 1:632(1.3%) | 0:57674(98.3%) 1:975(1.7%) |
| TZ48_The_skin_turns_red_when_  scratched_and_scratches_appear_4 | Classification |  |  | *P=.*33 | 0:25588(51.4%) 1:24210(48.6%) | 0:32252(55.0%) 1:26397(45.0%) |
| TZ48_The_skin_turns_red_when_  scratched_and_scratches_appear_5 | Classification |  |  | *P<*.001 | 0:28573(57.4%) 1:21225(42.6%) | 0:31688(54.0%) 1:26961(46.0%) |
| TZ49_Always_lively_and_strong_1 | Classification |  |  | *P=.*09 | 0:45992(92.4%) 1:3806(7.6%) | 0:54088(92.2%) 1:4561(7.8%) |
| TZ49_Always_lively_and_strong_2 | Classification |  |  | *P<*.001 | 0:49241(98.9%) 1:557(1.1%) | 0:57919(98.8%) 1:730(1.2%) |
| TZ49_Always_lively_and_strong_3 | Classification |  |  | *P<*.001 | 0:17012(34.2%) 1:32786(65.8%) | 0:21836(37.2%) 1:36813(62.8%) |
| TZ49_Always_lively_and_strong_4 | Classification |  |  | *P=.*05 | 0:35044(70.4%) 1:14754(29.6%) | 0:39755(67.8%) 1:18894(32.2%) |
| TZ49_Always_lively_and_strong_5 | Classification |  |  | *P<*.001 | 0:47874(96.1%) 1:1924(3.9%) | 0:56179(95.8%) 1:2470(4.2%) |
| TZ50_Can_adapt_to_weather  _changes_and_new_environment_1 | Classification |  |  | *P=.*03 | 0:49464(99.3%) 1:334(0.7%) | 0:58177(99.2%) 1:472(0.8%) |
| TZ50_Can_adapt_to_weather  _changes_and_new_environment_2 | Classification |  |  | *P<*.001 | 0:22965(46.1%) 1:26833(53.9%) | 0:28506(48.6%) 1:30143(51.4%) |
| TZ50_Can_adapt_to_weather  _changes_and_new_environment_3 | Classification |  |  | *P<*.001 | 0:30763(61.8%) 1:19035(38.2%) | 0:35135(59.9%) 1:23514(40.1%) |
| TZ50_Can_adapt_to_weather  _changes_and_new_environment_4 | Classification |  |  | *P=.*02 | 0:46486(93.3%) 1:3312(6.7%) | 0:54504(92.9%) 1:4145(7.1%) |
| TZ50_Can_adapt_to_weather  _changes_and_new_environment_5 | Classification |  |  | *P<*.001 | 0:49180(98.8%) 1:618(1.2%) | 0:57802(98.6%) 1:847(1.4%) |
| TZ51_Easy_to_have_nightmares_or  _wake_up_from_dreams_1 | Classification |  |  | *P<*.001 | 0:31895(64.0%) 1:17903(36.0%) | 0:38390(65.5%) 1:20259(34.5%) |
| TZ51_Easy_to_have_nightmares_or  _wake_up_from_dreams_2 | Classification |  |  | *P<*.001 | 0:26961(54.1%) 1:22837(45.9%) | 0:30822(52.6%) 1:27827(47.4%) |
| TZ51_Easy_to_have_nightmares_or  _wake_up_from_dreams_3 | Classification |  |  | *P<*.001 | 0:42601(85.5%) 1:7197(14.5%) | 0:50240(85.7%) 1:8409(14.3%) |
| TZ51_Easy_to_have_nightmares_or  _wake_up_from_dreams_4 | Classification |  |  | *P=.*21 | 0:47937(96.3%) 1:1861(3.7%) | 0:56495(96.3%) 1:2154(3.7%) |
| TZ51_Easy_to_have_nightmares_or  _wake_up_from_dreams_5 | Classification |  |  | *P=.*002 | 0:22445(45.1%) 1:27353(54.9%) | 0:28018(47.8%) 1:30631(52.2%) |
| YSRT1_Eat_less_than_your_peers_1 | Classification |  |  | *P<*.001 | 0:34,566 (69.4%) 1:15,232 (30.6%) | 0:39,312 (67.0%) 1:19,337 (33.0%) |
| YSRT1_Eat_less_than_your_peers_2 | Classification |  |  | *P<*.001 | 0:43,926 (88.2%) 1:5,872 (11.8%) | 0:51,441 (87.7%) 1:7,208 (12.3%) |
| YSRT1_Eat_less_than_your_peers_3 | Classification |  |  | *P<*.001 | 0:48,457 (97.3%) 1:1,341 (2.7%) | 0:57,176 (97.5%)  1:1,473 (2.5%) |
| YSRT1_Eat_less_than_your_peers_4 | Classification |  |  | *P<*.001 | 0:25,779 (51.8%) 1:24,019 (48.2%) | 0:31,293 (53.4%) 1:27,356 (46.6%) |
| YSRT2_Eating_slowly_compared  _with_peers_1 | Classification |  |  | *P<*.001 | 0:26,320 (52.9%) 1:23,478 (47.1%) | 0:30,189 (51.5%) 1:28,460 (48.5%) |
| YSRT2_Eating_slowly_compared  _with_peers_2 | Classification |  |  | *P=.*62 | 0:47,815 (96.0%) 1:1,983 (4.0%) | 0:56200(95.8%) 1:2449(4.2%) |
| YSRT2_Eating_slowly_compared  _with_peers_3 | Classification |  |  | *P<*.001 | 0:49480(99.4%) 1:318(0.6%) | 0:58265(99.3%) 1:384(0.7%) |
| YSRT2_Eating_slowly_compared  _with_peers_4 | Classification |  |  | *P<*.001 | 0:12552(25.2%) 1:37246(74.8%) | 0:15847(27.0%) 1:42802(73.0%) |
| YSRT3_Not_interested_in_food_1 | Classification |  |  | *P<*.001 | 0:38633(77.6%)1:11165(22.4%) | 0:44566(76.0%) 1:14083(24.0%) |
| YSRT3_Not_interested_in_food_2 | Classification |  |  | *P<*.001 | 0:48632(97.7%) 1:1166(2.3%) | 0:57189(97.5%) 1:1460(2.5%) |
| YSRT3_Not_interested_in_food_3 | Classification |  |  | *P<*.001 | 0:49577(99.6%) 1:221(0.4%) | 0:58345(99.5%) 1:304(0.5%) |
| YSRT3_Not_interested_in_food_4 | Classification |  |  | *P=.*001 | 0: 29,915 (60.1%) 1: 19,883 (39.9%) | 0: 35,818 (61.1%) 1: 22,831 (38.9%) |
| YSRT4_Refuse_certain_foods  _for_more_than_a_month_1 | Classification |  |  | *P<*.001 | 0: 23,308 (46.8%) 1: 26,490 (53.2%) | 0: 27,384 (46.7%) 1: 31,265 (53.3%) |
| YSRT4_Refuse_certain_foods  _for_more_than_a_month_2 | Classification |  |  | *P<*.001 | 0: 46,373 (93.1%) 1: 3,425 (6.9%) | 0: 54,096 (92.2%) 1: 4,553 (7.8%) |
| YSRT4_Refuse_certain_foods  _for_more_than_a_month_3 | Classification |  |  | *P=.*28 | 0: 23,042 (46.3%) 1: 26,756 (53.7%) | 0: 26,755 (45.6%) 1: 31,894 (54.4%) |
| YSRT4_Refuse_certain_foods  _for_more_than_a_month_4 | Classification |  |  | *P<*.001 | 0: 27,968 (56.2%) 1: 21,830 (43.8%) | 0: 34,716 (59.2%) 1: 23,933 (40.8%) |
| YSRT5_Unwilling_to_try  _new_food_1 | Classification |  |  | *P<*.001 | 0: 48,586 (97.6%) 1: 1,212 (2.4%) | 0: 55,827 (95.2%) 1: 2,822 (4.8%) |
| YSRT5_Unwilling_to_try  _new_food_2 | Classification |  |  | *P=.*03 | Category 0: 33951 (68.2%); Category 1: 15847 (31.8%) | Category 0: 40805 (69.6%); Category 1: 17844 (30.4%) |
| YSRT5_Unwilling_to_try  _new_food_3 | Classification |  |  | *P=.*22 | 0: 33,720 (67.7%) 1: 16,078 (32.3%) | 0: 39,179 (66.8%) 1: 19,470 (33.2%) |
| YSRT5_Unwilling_to_try  _new_food_4 | Classification |  |  | *P=.*004 | 0: 32,037 (64.3%) 1: 17,761 (35.7%) | 0:37461(63.9%) 1:21188(36.1%) |
| YSRT6_Have_a_strong_preference  _for_certain_textures_  or_certain_types_of_food_1 | Classification |  |  | *P<*.001 | 0:49686(99.8%) 1:112(0.2%) | 0:58502(99.7%) 1:147(0.3%) |
| YSRT6_Have_a_strong_preference  _for_certain_textures_  or_certain_types_of_food_2 | Classification |  |  | *P=.*005 | 0:23770(47.7%) 1:26028(52.3%) | 0: 27,053 (46.1%) 1: 31,596 (53.9%) |
| YSRT6_Have_a_strong_preference  _for_certain_textures_  or_certain_types_of_food_3 | Classification |  |  | *P=.*02 | 0: 45,782 (91.9%) 1: 4,016 (8.1%) | 0: 53,837 (91.8%) 1: 4,812 (8.2%) |
| YSRT6_Have_a_strong_preference  _for_certain_textures_  or_certain_types_of_food_4 | Classification |  |  | *P=.*01 | 0:32010(64.3%) 1:17788(35.7%) | 0: 38,179 (65.1%) 1: 20,470 (34.9%) |
| YSRT7_Distraction_from_eating_1 | Classification |  |  | *P<*.001 | 0:34463(69.2%) 1:15335(30.8%) | 0:40180(68.5%) 1:18469(31.5%) |
| YSRT7_Distraction_from_eating_2 | Classification |  |  | *P=.*34 | 0:37473(75.3%) 1:12325(24.7%) | 0:44200(75.4%) 1:14449(24.6%) |
| YSRT7_Distraction_from_eating_3 | Classification |  |  | *P<*.001 | 0:49464(99.3%) 1:334(0.7%) | 0: 58,200 (99.2%) 1: 449 (0.8%) |
| YSRT7_Distraction_from_eating_4 | Classification |  |  | *P<*.001 | 0: 45,957 (92.3%) 1: 3,841 (7.7%) | 0:53886(91.9%) 1:4763(8.1%) |
| YSRT8_The_dining_place_is_not_fixed_1 | Classification |  |  | *P<*.001 | 0:46689(93.8%) 1:3109(6.2%) | 0:54888(93.6%) 1:3761(6.4%) |
| YSRT8_The_dining_place_is_not_fixed_2 | Classification |  |  | *P<*.001 | 0:46136(92.6%) 1:3662(7.4%) | 0:54292(92.6%) 1:4357(7.4%) |
| YSRT8_The_dining_place_is_not_fixed_3 | Classification |  |  | *P<*.001 | 0:44781(89.9%) 1:5017(10.1%) | 0:52729(89.9%) 1:5920(10.1%) |
| YSRT8_The_dining_place_is_not_fixed_4 | Classification |  |  | *P<*.001 | 0:41688(83.7%) 1:8110(16.3%) | 0:49399(84.2%) 1:9250(15.8%) |
| YSFY1_Few_emotional_exchanges  _during_meals_1 | Classification |  |  | *P<*.001 | 0:44718(89.8%) 1:5080(10.2%) | 0:52636(89.7%)  1:6013(10.3%) |
| YSFY1_Few_emotional_exchanges  _during_meals_2 | Classification |  |  | *P<*.001 | 0:45520(91.4%) 1:4278(8.6%) | 0:53612(91.4%) 1:5037(8.6%) |
| YSFY1_Few_emotional_exchanges  _during_meals_3 | Classification |  |  | *P=.*42 | 0:45257(90.9%) 1:4541(9.1%) | 0: 53,231 (90.8%) 1: 5,418 (9.2%) |
| YSFY1_Few_emotional_exchanges  _during_meals_4 | Classification |  |  | *P=.*06 | 0: 46,029 (92.4%) 1: 3,769 (7.6%) | 0:54358(92.7%) 1:4291(7.3%) |
| YSFY2_Force_or_punish_  children_to_eat_more_1 | Classification |  |  | *P<*.001 | 0:46598(93.6%) 1:3200(6.4%) | 0: 54,896 (93.6%) 1: 3,753 (6.4%) |
| YSFY2_Force_or_punish_  children_to_eat_more_2 | Classification |  |  | *P<*.001 | 0:47027(94.4%) 1:2771(5.6%) | 0: 55,532 (94.7%) 1: 3,117 (5.3%) |
| YSFY2_Force_or_punish_  children_to_eat_more_3 | Classification |  |  | *P=.*004 | 0: 47,378 (95.1%) 1: 2,420 (4.9%) | 0:55680(94.9%) 1:2969(5.1%) |
| YSFY2_Force_or_punish_  children_to_eat_more_4 | Classification |  |  | *P=.*01 | 0:2252(4.5%) 1:47546(95.5%) | 0: 2,825 (4.8%) 1: 55,824 (95.2%) |
| YSFY3_Induce_children_to_eat_1 | Classification |  |  | *P<*.001 | 0: 13,688 (27.5%) 1: 36,110 (72.5%) | 0: 15,604 (26.6%) 1: 43,045 (73.4%) |
| YSFY3_Induce_children_to_eat_2 | Classification |  |  | *P<*.001 | 0: 2,460 (4.9%) 1: 47,338 (95.1%) | 0: 3,513 (6.0%) 1: 55,136 (94.0%) |
| YSFY3_Induce_children_to_eat_3 | Classification |  |  | *P=.*007 | 0: 5,404 (10.9%) 1: 44,394 (89.1%) | 0: 7,067 (12.0%) 1: 51,582 (88.0%) |
| YSFY3_Induce_children_to_eat_4 | Classification |  |  | *P=.*004 | 0: 46,616 (93.6%) 1: 3,182 (6.4%) | 0: 54,123 (92.3%) 1: 4,526 (7.7%) |
| YSFY4_Allow_children_to_  choose_food_at_will_1 | Classification |  |  | *P<*.001 | 0: 47,576 (95.5%) 1: 2,222 (4.5%) | 0: 56,108 (95.7%) 1: 2,541 (4.3%) |
| YSFY4_Allow_children_to_  choose_food_at_will_2 | Classification |  |  | *P<*.001 | 0:16015(32.2%) 1:33783(67.8%) | 0:19990(34.1%) 1:38659(65.9%) |
| YSFY4_Allow_children_to_  choose_food_at_will_3 | Classification |  |  | *P=.*60 | 0:49481(99.4%) 1:317(0.6%) | 0:58219(99.3%) 1:430(0.7%) |
| YSFY4_Allow_children_to_  choose_food_at_will_4 | Classification |  |  | *P=.*59 | 0:49649(99.7%) 1:149(0.3%) | Class 0: 58,470 (99.7%) Class 1: 179 (0.3%) |
| YSFY5_Children_are_rarely_  encouraged_to_taste_new_foods_1 | Classification |  |  | *P<*.001 | 0:35052(70.4%) 1:14746(29.6%) | 0:40525(69.1%) 1:18124(30.9%) |
| YSFY5_Children_are_rarely_  encouraged_to_taste_new_foods_2 | Classification |  |  | *P<*.001 | 0:49757(99.9%) 1:41(0.1%) | 0:58594(99.9%) 1:55(0.1%) |
| YSFY5_Children_are_rarely_encouraged_to_taste_new_foods_3 | Classification |  |  | *P=.*01 | 0:49347(99.1%) 1:451(0.9%) | 0:58060(99.0%) 1:589(1.0%) |
| YSFY5_Children_are_rarely_  encouraged_to_taste_new_foods_4 | Classification |  |  | *P=.*06 | 0:48540(97.5%) 1:1258(2.5%) | 0:56829(96.9%) 1:1820(3.1%) |
| YSFY6_Allow_children_to_eat_  snacks_if_they_want_1 | Classification |  |  | *P<*.001 | 0:9802(19.7%) 1:39996(80.3%) | 0: 11,826 (20.2%) 1: 46,823 (79.8%) |
| YSFY6_Allow_children_to_eat_  snacks_if_they_want_2 | Classification |  |  | *P<*.001 | 0: 40,455 (81.2%) 1: 9,343 (18.8%) | 0:47,522 (81.0%) 1:11,127 (19.0%) |
| YSFY6_Allow_children_to_eat_  snacks_if_they_want_3 | Classification |  |  | *P=.*11 | 0: 49,339 (99.1%) 1: 459 (0.9%) | 0: 57,950 (98.8%) 1: 699 (1.2%) |
| YSFY6_Allow_children_to_eat_  snacks_if_they_want_4 | Classification |  |  | *P=.*77 | 0:45,323 (91.0%) 1:4,475 (9.0%) | 0:53,165 (90.6%) 1:5,484 (9.4%) |
| YSFY7_Allow_children_to_dine_  and_wander_around_1 | Classification |  |  | *P<*.001 | 0: 11,711 (23.5%) 1: 38,087 (76.5%) | 0: 14,462 (24.7%) 1: 44,187 (75.3%) |
| YSFY7_Allow_children_to_dine_  and_wander_around_2 | Classification |  |  | *P<*.001 | 0: 49,411 (99.2%) 1: 387 (0.8%) | 0: 58,140 (99.1%) 1: 509 (0.9%) |
| YSFY7_Allow_children_to_dine_  and_wander_around_3 | Classification |  |  | *P=.*12 | 0:44367(89.1%) 1:5431(10.9%) | 0: 52,180 (89.0%) 1: 6,469 (11.0%) |
| YSFY7_Allow_children_to_dine_  and_wander_around_4 | Classification |  |  | *P=.*09 | 0: 48,841 (98.1%) 1: 957 (1.9%) | 0:57314(97.7%) 1:1335(2.3%) |
| YS1_Your_child_average  _meal_time_1 | Classification |  |  | *P=.*0008 | 0: 49,337 (99.1%) 1: 461 (0.9%) | 0:58241(99.3%) 1:408(0.7%) |
| YS1_Your_child_average  _meal_time_2 | Classification |  |  | *P=.*71 | 0:35385(71.1%) 1:14413(28.9%) | 0:40,300 (68.7%) 1:18,349 (31.3%) |
| YS1_Your_child_average  _meal_time_3 | Classification |  |  | *P<*.001 | 0:42,874 (86.1%) 1:6,924 (13.9%) | 0:50176(85.6%) 1:8473(14.4%) |
| YS2_Do_you_think_your_child  _has_a_picky_eater_problem_1 | Classification |  |  | *P=.*03 | 0:43,285 (86.9%) 1:6,513 (13.1%) | 0:50285(85.7%) 1:8364(14.3%) |
| YS2_Do_you_think_your_child  _has_a_picky_eater_problem_2 | Classification |  |  | *P<*.001 | 0:48881(98.2%) 1:917(1.8%) | 0:57,350 (97.8%) 1:1,299 (2.2%) |
| YS2_Do_you_think_your_child  _has_a_picky_eater_problem_3 | Classification |  |  | *P<*.001 | 0:47,042 (94.5%) 1:2,756 (5.5%) | 0:55203(94.1%) 1:3446(5.9%) |

**Table S4. Results of univariate correlation-based filtering using Pearson correlation coefficients**

| Feature Name | Pearson Correlation | Absolute Correlation | Above Threshold | Selection Result |  |  |  |  |
| --- | --- | --- | --- | --- | --- | --- | --- | --- |
| TZ22 Thick and greasy tongue coating :Never | -0.265308322 | 0.265308322 | TRUE | Retained |  |  |  |  |
| TZ2 Yellow complexion,no other children ruddy :Never | -0.250676899 | 0.250676899 | TRUE | Retained |  |  |  |  |
| TZ23 Always red eyes or easy to get gum :Never | -0.242735296 | 0.242735296 | TRUE | Retained |  |  |  |  |
| TZ14 Skin and lips dry easily :Never | -0.23683845 | 0.23683845 | TRUE | Retained |  |  |  |  |
| TZ37 Easily afraid or frightened :Never | -0.236808421 | 0.236808421 | TRUE | Retained |  |  |  |  |
| TZ3 Soft muscles,not as strong as other children :Never | -0.228282703 | 0.228282703 | TRUE | Retained |  |  |  |  |
| TZ26 Sweat is sticky :Never | -0.225608106 | 0.225608106 | TRUE | Retained |  |  |  |  |
| TZ13 The body and face are always very hot :Never | -0.224467494 | 0.224467494 | TRUE | Retained |  |  |  |  |
| TZ15 Always excited :Never | -0.220636838 | 0.220636838 | TRUE | Retained |  |  |  |  |
| TZ36 Easy to lack self confidence :Never | -0.21978167 | 0.21978167 | TRUE | Retained |  |  |  |  |
| TZ1 Easy to lose spirit :Never | -0.215852716 | 0.215852716 | TRUE | Retained |  |  |  |  |
| TZ40 Always worried about a lot of things :Never | -0.212863986 | 0.212863986 | TRUE | Retained |  |  |  |  |
| TZ38 Always crying when in trouble :Never | -0.212380745 | 0.212380745 | TRUE | Retained |  |  |  |  |
| TZ16 Easily restless :Never | -0.208527654 | 0.208527654 | TRUE | Retained |  |  |  |  |
| TZ8 Hands and feet always lukewarm :Never | -0.20775322 | 0.20775322 | TRUE | Retained |  |  |  |  |
| TZ35 Easily nervous :Never | -0.205181849 | 0.205181849 | TRUE | Retained |  |  |  |  |
| TZ51 Easy to have nightmares or wake up from dreams :Never | -0.205116287 | 0.205116287 | TRUE | Retained |  |  |  |  |
| YSRT6 Have a strong preference for certain textures or certain types of food :Never | -0.204342856 | 0.204342856 | TRUE | Retained |  |  |  |  |
| YSRT3 Not interested in food :Never | -0.202818317 | 0.202818317 | TRUE | Retained |  |  |  |  |
| TZ41 More attached to you now than before :Never | -0.201169375 | 0.201169375 | TRUE | Retained |  |  |  |  |
| TZ39 Easily shy or timid :Never | -0.199512414 | 0.199512414 | TRUE | Retained |  |  |  |  |
| YSFY2 Force or punish children to eat more :Never | -0.198905363 | 0.198905363 | TRUE | Retained |  |  |  |  |
| TZ30 Be prone to dark circles :Never | -0.197511123 | 0.197511123 | TRUE | Retained |  |  |  |  |
| TZ20 Like to eat sweet or greasy things :Never | -0.19722965 | 0.19722965 | TRUE | Retained |  |  |  |  |
| YS2 Do you think your child has a picky eater problem:Do not worry | -0.19721001 | 0.19721001 | TRUE | Retained |  |  |  |  |
| YSRT4 Refuse certain foods for more than a month :Never | -0.19564513 | 0.19564513 | TRUE | Retained |  |  |  |  |
| YS2 Do you think your child has a picky eater problem:Worry | 0.193437668 | 0.193437668 | TRUE | Retained |  |  |  |  |
| TZ9 Resistant to summer but not to winter :Never | -0.189921222 | 0.189921222 | TRUE | Retained |  |  |  |  |
| TZ7 Little activity makes sweating easily :Never | -0.189003324 | 0.189003324 | TRUE | Retained |  |  |  |  |
| YSRT1 Eat less than your peers :Never | -0.188262866 | 0.188262866 | TRUE | Retained |  |  |  |  |
| YSRT5 Unwilling to try new food :Never | -0.182520126 | 0.182520126 | TRUE | Retained |  |  |  |  |
| TZ21 Snoring at night :Never | -0.179807683 | 0.179807683 | TRUE | Retained |  |  |  |  |
| TZ27 The skin always appears blue and purple ecchymosis unconsciously :Never | -0.178335471 | 0.178335471 | TRUE | Retained |  |  |  |  |
| TZ24 Eczema is easy to grow on the body :Never | -0.177590934 | 0.177590934 | TRUE | Retained |  |  |  |  |
| TZ46 The skin is prone to itching and urticaria :Never | -0.17568685 | 0.17568685 | TRUE | Retained |  |  |  |  |
| YSFY3 Induce children to eat :Never | -0.174525937 | 0.174525937 | TRUE | Retained |  |  |  |  |
| TZ48 The skin turns red when scratched and scratches appear :Never | -0.172695208 | 0.172695208 | TRUE | Retained |  |  |  |  |
| TZ43 Without cold but have stuffy nose and runny nose :Never | -0.1726403 | 0.1726403 | TRUE | Retained |  |  |  |  |
| TZ42 Sneez when do not have a cold :Never | -0.168058916 | 0.168058916 | TRUE | Retained |  |  |  |  |
| TZ50 Can adapt to weather changes and new environment :Never | -0.167199375 | 0.167199375 | TRUE | Retained |  |  |  |  |
| YSFY4 Allow children to choose food at will :Never | -0.167048633 | 0.167048633 | TRUE | Retained |  |  |  |  |
| TZ49 Always lively and strong :Never | -0.166970302 | 0.166970302 | TRUE | Retained |  |  |  |  |
| TZ28 Where there is pain on the body :Never | -0.166755123 | 0.166755123 | TRUE | Retained |  |  |  |  |
| YSRT2 Eating slowly compared with peers :Never | -0.166613274 | 0.166613274 | TRUE | Retained |  |  |  |  |
| TZ19 Always sleepy :Never | -0.16403187 | 0.16403187 | TRUE | Retained |  |  |  |  |
| TZ29 The skin on the body or the skin on the neck armpit or thigh root is dark :Never | -0.163748848 | 0.163748848 | TRUE | Retained |  |  |  |  |
| TZ34 Always moping :Never | -0.163133665 | 0.163133665 | TRUE | Retained |  |  |  |  |
| TZ22 Thick and greasy tongue coating :Rarely | 0.162892594 | 0.162892594 | TRUE | Retained |  |  |  |  |
| TZ11 Feel uncomfortable or afraid of eating(drinking)cold food :Never | -0.161612661 | 0.161612661 | TRUE | Retained |  |  |  |  |
| TZ44 Cough and wheeze when the season changes the temperature changes or smells strange smell :Never | -0.160607739 | 0.160607739 | TRUE | Retained |  |  |  |  |
| TZ23 Always red eyes or easy to get gum :Rarely | 0.159438733 | 0.159438733 | TRUE | Retained |  |  |  |  |
| TZ10 Afraid of cold than other children of the same age :Never | -0.15866987 | 0.15866987 | TRUE | Retained |  |  |  |  |
| TZ45 Susceptible to allergies :Never | -0.158049779 | 0.158049779 | TRUE | Retained |  |  |  |  |
| TZ22 Thick and greasy tongue coating :Sometimes | 0.157459404 | 0.157459404 | TRUE | Retained |  |  |  |  |
| TZ12 Having cold or eating cold food makes it easy to have diarrhea :Never | -0.156368054 | 0.156368054 | TRUE | Retained |  |  |  |  |
| TZ14 Skin and lips dry easily :Rarely | 0.155148987 | 0.155148987 | TRUE | Retained |  |  |  |  |
| YSRT1 Eat less than your peers :Often | 0.154856795 | 0.154856795 | TRUE | Retained |  |  |  |  |
| TZ13 The body and face are always very hot :Rarely | 0.154689018 | 0.154689018 | TRUE | Retained |  |  |  |  |
| Food preference No obvious | -0.153356485 | 0.153356485 | TRUE | Retained |  |  |  |  |
| YSRT7 Distraction from eating :Never | -0.152781628 | 0.152781628 | TRUE | Retained |  |  |  |  |
| YSFY2 Force or punish children to eat more :Sometimes | 0.152378954 | 0.152378954 | TRUE | Retained |  |  |  |  |
| TZ1 Easy to lose spirit :Rarely | 0.151396786 | 0.151396786 | TRUE | Retained |  |  |  |  |
| TZ33 Lips are dark or blue :Never | -0.150502162 | 0.150502162 | TRUE | Retained |  |  |  |  |
| YSRT8 The dining place is not fixed :Never | -0.149947573 | 0.149947573 | TRUE | Retained |  |  |  |  |
| YSRT6 Have a strong preference for certain textures or certain types of food :Often | 0.149061797 | 0.149061797 | TRUE | Retained |  |  |  |  |
| TZ51 Easy to have nightmares or wake up from dreams :Rarely | 0.148644648 | 0.148644648 | TRUE | Retained |  |  |  |  |
| TZ25 Like to eat spicy or fried barbecue food :Never | -0.146560199 | 0.146560199 | TRUE | Retained |  |  |  |  |
| TZ8 Hands and feet always lukewarm :Rarely | 0.14648964 | 0.14648964 | TRUE | Retained |  |  |  |  |
| TZ35 Easily nervous :Rarely | 0.146024386 | 0.146024386 | TRUE | Retained |  |  |  |  |
| YSRT2 Eating slowly compared with peers :Often | 0.145790549 | 0.145790549 | TRUE | Retained |  |  |  |  |
| TZ37 Easily afraid or frightened :Sometimes | 0.144927027 | 0.144927027 | TRUE | Retained |  |  |  |  |
| TZ2 Yellow complexion,no other children ruddy :Sometimes | 0.144383572 | 0.144383572 | TRUE | Retained |  |  |  |  |
| TZ31 Blue or dark gums :Never | -0.143573634 | 0.143573634 | TRUE | Retained |  |  |  |  |
| YSRT3 Not interested in food :Often | 0.143203635 | 0.143203635 | TRUE | Retained |  |  |  |  |
| TZ26 Sweat is sticky :Rarely | 0.142203763 | 0.142203763 | TRUE | Retained |  |  |  |  |
| TZ5 Speak in a low voice :Never | -0.141546879 | 0.141546879 | TRUE | Retained |  |  |  |  |
| TZ23 Always red eyes or easy to get gum :Sometimes | 0.14035391 | 0.14035391 | TRUE | Retained |  |  |  |  |
| TZ36 Easy to lack self confidence :Sometimes | 0.140121541 | 0.140121541 | TRUE | Retained |  |  |  |  |
| TZ19 Always sleepy :Rarely | 0.138275145 | 0.138275145 | TRUE | Retained |  |  |  |  |
| TZ26 Sweat is sticky :Sometimes | 0.136904353 | 0.136904353 | TRUE | Retained |  |  |  |  |
| TZ34 Always moping :Rarely | 0.135129042 | 0.135129042 | TRUE | Retained |  |  |  |  |
| TZ28 Where there is pain on the body :Rarely | 0.1347602 | 0.1347602 | TRUE | Retained |  |  |  |  |
| TZ1 Easy to lose spirit :Sometimes | 0.133434772 | 0.133434772 | TRUE | Retained |  |  |  |  |
| YSFY7 Allow children to dine and wander around :Never | -0.133288911 | 0.133288911 | TRUE | Retained |  |  |  |  |
| TZ39 Easily shy or timid :Sometimes | 0.133232793 | 0.133232793 | TRUE | Retained |  |  |  |  |
| Self control ability Good at self control | -0.131330211 | 0.131330211 | TRUE | Retained |  |  |  |  |
| YSFY1 Few emotional exchanges during meals :Never | -0.131156893 | 0.131156893 | TRUE | Retained |  |  |  |  |
| TZ27 The skin always appears blue and purple ecchymosis unconsciously :Rarely | 0.130218275 | 0.130218275 | TRUE | Retained |  |  |  |  |
| TZ20 Like to eat sweet or greasy things :Often | 0.128890964 | 0.128890964 | TRUE | Retained |  |  |  |  |
| YSRT2 Eating slowly compared with peers :Always | 0.128425497 | 0.128425497 | TRUE | Retained |  |  |  |  |
| TZ14 Skin and lips dry easily :Sometimes | 0.12813304 | 0.12813304 | TRUE | Retained |  |  |  |  |
| TZ3 Soft muscles,not as strong as other children :Sometimes | 0.127577092 | 0.127577092 | TRUE | Retained |  |  |  |  |
| TZ2 Yellow complexion,no other children ruddy :Often | 0.127231757 | 0.127231757 | TRUE | Retained |  |  |  |  |
| TZ40 Always worried about a lot of things :Sometimes | 0.125968497 | 0.125968497 | TRUE | Retained |  |  |  |  |
| TZ40 Always worried about a lot of things :Rarely | 0.125968497 | 0.125968497 | TRUE | Retained |  |  |  |  |
| YSRT7 Distraction from eating :Often | 0.125619335 | 0.125619335 | TRUE | Retained |  |  |  |  |
| History of allergies | 0.125292896 | 0.125292896 | TRUE | Retained |  |  |  |  |
| TZ38 Always crying when in trouble :Sometimes | 0.125227971 | 0.125227971 | TRUE | Retained |  |  |  |  |
| YSRT5 Unwilling to try new food :Often | 0.125206625 | 0.125206625 | TRUE | Retained |  |  |  |  |
| TZ33 Lips are dark or blue :Rarely | 0.125128447 | 0.125128447 | TRUE | Retained |  |  |  |  |
| TZ9 Resistant to summer but not to winter :Rarely | 0.124926401 | 0.124926401 | TRUE | Retained |  |  |  |  |
| TZ15 Always excited :Sometimes | 0.124447363 | 0.124447363 | TRUE | Retained |  |  |  |  |
| TZ38 Always crying when in trouble :Often | 0.123908457 | 0.123908457 | TRUE | Retained |  |  |  |  |
| TZ48 The skin turns red when scratched and scratches appear :Rarely | 0.122775567 | 0.122775567 | TRUE | Retained |  |  |  |  |
| TZ37 Easily afraid or frightened :Rarely | 0.122333958 | 0.122333958 | TRUE | Retained |  |  |  |  |
| YSRT7 Distraction from eating :Always | 0.120340332 | 0.120340332 | TRUE | Retained |  |  |  |  |
| TZ32 Rough skin like fish scales :Never | -0.119917082 | 0.119917082 | TRUE | Retained |  |  |  |  |
| YSRT4 Refuse certain foods for more than a month :Often | 0.119916873 | 0.119916873 | TRUE | Retained |  |  |  |  |
| TZ13 The body and face are always very hot :Sometimes | 0.119781988 | 0.119781988 | TRUE | Retained |  |  |  |  |
| TZ10 Afraid of cold than other children of the same age :Rarely | 0.118806464 | 0.118806464 | TRUE | Retained |  |  |  |  |
| TZ35 Easily nervous :Sometimes | 0.118279819 | 0.118279819 | TRUE | Retained |  |  |  |  |
| TZ4 Always quiet :Never | -0.117808944 | 0.117808944 | TRUE | Retained |  |  |  |  |
| TZ6 Not talkative :Never | -0.11747929 | 0.11747929 | TRUE | Retained |  |  |  |  |
| TZ29 The skin on the body or the skin on the neck armpit or thigh root is dark :Rarely | 0.116831384 | 0.116831384 | TRUE | Retained |  |  |  |  |
| TZ18 Abdominal obesity is flabby and soft :Never | -0.116401871 | 0.116401871 | TRUE | Retained |  |  |  |  |
| TZ41 More attached to you now than before :Sometimes | 0.115943871 | 0.115943871 | TRUE | Retained |  |  |  |  |
| TZ16 Easily restless :Sometimes | 0.115629252 | 0.115629252 | TRUE | Retained |  |  |  |  |
| TZ44 Cough and wheeze when the season changes the temperature changes or smells strange smell :Rarely | 0.11526948 | 0.11526948 | TRUE | Retained |  |  |  |  |
| The earliest age of using antibiotics None | -0.114824102 | 0.114824102 | TRUE | Retained |  |  |  |  |
| Have been treated with antibiotics | 0.114824102 | 0.114824102 | TRUE | Retained |  |  |  |  |
| YSFY6 Allow children to eat snacks if they want :Never | -0.114379311 | 0.114379311 | TRUE | Retained |  |  |  |  |
| TZ3 Soft muscles,not as strong as other children :Often | 0.11408936 | 0.11408936 | TRUE | Retained |  |  |  |  |
| YSRT1 Eat less than your peers :Always | 0.11390348 | 0.11390348 | TRUE | Retained |  |  |  |  |
| TZ8 Hands and feet always lukewarm :Sometimes | 0.113792113 | 0.113792113 | TRUE | Retained |  |  |  |  |
| TZ30 Be prone to dark circles :Rarely | 0.113631706 | 0.113631706 | TRUE | Retained |  |  |  |  |
| TZ31 Blue or dark gums :Rarely | 0.112242828 | 0.112242828 | TRUE | Retained |  |  |  |  |
| TZ2 Yellow complexion,no other children ruddy :Rarely | 0.111894182 | 0.111894182 | TRUE | Retained |  |  |  |  |
| TZ47 Purpura has occurred in the skin due to allergies :Never | -0.111890672 | 0.111890672 | TRUE | Retained |  |  |  |  |
| TZ15 Always excited :Often | 0.111271208 | 0.111271208 | TRUE | Retained |  |  |  |  |
| TZ3 Soft muscles,not as strong as other children :Rarely | 0.11059764 | 0.11059764 | TRUE | Retained |  |  |  |  |
| YSFY5 Children are rarely encouraged to taste new foods :Never | -0.110444721 | 0.110444721 | TRUE | Retained |  |  |  |  |
| YSFY3 Induce children to eat :Sometimes | 0.110154929 | 0.110154929 | TRUE | Retained |  |  |  |  |
| TZ12 Having cold or eating cold food makes it easy to have diarrhea :Rarely | 0.109990913 | 0.109990913 | TRUE | Retained |  |  |  |  |
| YSFY3 Induce children to eat :Often | 0.109869298 | 0.109869298 | TRUE | Retained |  |  |  |  |
| TZ9 Resistant to summer but not to winter :Sometimes | 0.109701682 | 0.109701682 | TRUE | Retained |  |  |  |  |
| TZ21 Snoring at night :Rarely | 0.109488208 | 0.109488208 | TRUE | Retained |  |  |  |  |
| TZ45 Susceptible to allergies :Rarely | 0.109238121 | 0.109238121 | TRUE | Retained |  |  |  |  |
| TZ7 Little activity makes sweating easily :Often | 0.108308974 | 0.108308974 | TRUE | Retained |  |  |  |  |
| TZ5 Speak in a low voice :Rarely | 0.108267208 | 0.108267208 | TRUE | Retained |  |  |  |  |
| TZ46 The skin is prone to itching and urticaria :Rarely | 0.108169841 | 0.108169841 | TRUE | Retained |  |  |  |  |
| TZ7 Little activity makes sweating easily :Always | 0.108093091 | 0.108093091 | TRUE | Retained |  |  |  |  |
| TZ30 Be prone to dark circles :Sometimes | 0.107792148 | 0.107792148 | TRUE | Retained |  |  |  |  |
| TZ24 Eczema is easy to grow on the body :Rarely | 0.107737778 | 0.107737778 | TRUE | Retained |  |  |  |  |
| History of digestive system diseases | 0.107721224 | 0.107721224 | TRUE | Retained |  |  |  |  |
| YSFY7 Allow children to dine and wander around :Sometimes | 0.107705697 | 0.107705697 | TRUE | Retained |  |  |  |  |
| TZ11 Feel uncomfortable or afraid of eating cold food :Rarely | 0.107617115 | 0.107617115 | TRUE | Retained |  |  |  |  |
| TZ21 Snoring at night :Sometimes | 0.107054388 | 0.107054388 | TRUE | Retained |  |  |  |  |
| YSRT6 Have a strong preference for certain textures or certain types of food :Always | 0.106703311 | 0.106703311 | TRUE | Retained |  |  |  |  |
| TZ51 Easy to have nightmares or wake up from dreams :Sometimes | 0.106429845 | 0.106429845 | TRUE | Retained |  |  |  |  |
| YSRT4 Refuse certain foods for more than a month :Always | 0.106201784 | 0.106201784 | TRUE | Retained |  |  |  |  |
| TZ36 Easy to lack self confidence :Rarely | 0.106051102 | 0.106051102 | TRUE | Retained |  |  |  |  |
| YSFY2 Force or punish children to eat more :Often | 0.105643826 | 0.105643826 | TRUE | Retained |  |  |  |  |
| TZ42 Sneez when do not have a cold :Rarely | 0.105476201 | 0.105476201 | TRUE | Retained |  |  |  |  |
| YSFY1 Few emotional exchanges during meals :Sometimes | 0.10500438 | 0.10500438 | TRUE | Retained |  |  |  |  |
| Food preference Have much hated food | 0.104675674 | 0.104675674 | TRUE | Retained |  |  |  |  |
| TZ43 Without cold but have stuffy nose and runny nose :Rarely | 0.104261507 | 0.104261507 | TRUE | Retained |  |  |  |  |
| TZ20 Like to eat sweet or greasy things :Sometimes | 0.104238937 | 0.104238937 | TRUE | Retained |  |  |  |  |
| YS1 Your child average meal time:<25min | -0.103846451 | 0.103846451 | TRUE | Retained |  |  |  |  |
| Regular lunch breaks | -0.102232372 | 0.102232372 | TRUE | Retained |  |  |  |  |
| Which child of mother First | 0.102123048 | 0.102123048 | TRUE | Retained |  |  |  |  |
| TZ39 Easily shy or timid :Often | 0.101298698 | 0.101298698 | TRUE | Retained |  |  |  |  |
| YSRT3 Not interested in food :Sometimes | 0.100374827 | 0.100374827 | TRUE | Retained |  |  |  |  |
| Which child of father first | 0.099670855 | 0.099670855 | FALSE | Excluded |  |  |  |  |
| TZ43 Without cold but have stuffy nose and runny nose :Sometimes | 0.099459072 | 0.099459072 | FALSE | Excluded |  |  |  |  |
| Self control ability Rambunctious | 0.099423359 | 0.099423359 | FALSE | Excluded |  |  |  |  |
| YSFY4 Allow children to choose food at will :Often | 0.098823741 | 0.098823741 | FALSE | Excluded |  |  |  |  |
| TZ32 Rough skin like fish scales :Rarely | 0.09809945 | 0.09809945 | FALSE | Excluded |  |  |  |  |
| YSRT8 The dining place is not fixed :Sometimes | 0.097648445 | 0.097648445 | FALSE | Excluded |  |  |  |  |
| TZ47 Purpura has occurred in the skin due to allergies :Rarely | 0.096434259 | 0.096434259 | FALSE | Excluded |  |  |  |  |
| YSRT5 Unwilling to try new food :Always | 0.096345031 | 0.096345031 | FALSE | Excluded |  |  |  |  |
| TZ16 Easily restless :Often | 0.096274649 | 0.096274649 | FALSE | Excluded |  |  |  |  |
| Eczema or atopic dermatitis | 0.094684911 | 0.094684911 | FALSE | Excluded |  |  |  |  |
| Allergic rhinitis | 0.094609629 | 0.094609629 | FALSE | Excluded |  |  |  |  |
| TZ46 The skin is prone to itching and urticaria :Sometimes | 0.094525867 | 0.094525867 | FALSE | Excluded |  |  |  |  |
| TZ17 Being overweight and unwilling to exercise :Never | -0.094353971 | 0.094353971 | FALSE | Excluded |  |  |  |  |
| YSRT4 Refuse certain foods for more than a month :Sometimes | 0.094181642 | 0.094181642 | FALSE | Excluded |  |  |  |  |
| TZ24 Eczema is easy to grow on the body :Sometimes | 0.093928438 | 0.093928438 | FALSE | Excluded |  |  |  |  |
| YSRT8 The dining place is not fixed :Often | 0.093431042 | 0.093431042 | FALSE | Excluded |  |  |  |  |
| TZ36 Easy to lack self confidence :Often | 0.093015112 | 0.093015112 | FALSE | Excluded |  |  |  |  |
| TZ37 Easily afraid or frightened :Often | 0.092888731 | 0.092888731 | FALSE | Excluded |  |  |  |  |
| Food preference Have much loved food | 0.09244636 | 0.09244636 | FALSE | Excluded |  |  |  |  |
| TZ25 Like to eat spicy or fried barbecue food :Sometimes | 0.092388186 | 0.092388186 | FALSE | Excluded |  |  |  |  |
| TZ27 The skin always appears blue and purple ecchymosis unconsciously :Sometimes | 0.092045886 | 0.092045886 | FALSE | Excluded |  |  |  |  |
| YSRT3 Not interested in food :Always | 0.091826426 | 0.091826426 | FALSE | Excluded |  |  |  |  |
| TZ42 Sneez when do not have a cold :Sometimes | 0.090145838 | 0.090145838 | FALSE | Excluded |  |  |  |  |
| TZ44 Cough and wheeze when the season changes the temperature changes or smells strange smell :Sometimes | 0.089279647 | 0.089279647 | FALSE | Excluded |  |  |  |  |
| YSFY6 Allow children to eat snacks if they want :Sometimes | 0.088377299 | 0.088377299 | FALSE | Excluded |  |  |  |  |
| Weight kg | -0.088102641 | 0.088102641 | FALSE | Excluded |  |  |  |  |
| TZ6 Not talkative :Rarely | 0.087498612 | 0.087498612 | FALSE | Excluded |  |  |  |  |
| TZ41 More attached to you now than before :Rarely | 0.087184406 | 0.087184406 | FALSE | Excluded |  |  |  |  |
| TZ41 More attached to you now than before :Often | 0.087099586 | 0.087099586 | FALSE | Excluded |  |  |  |  |
| TZ11 Feel uncomfortable or afraid of eating cold food :Sometimes | 0.085676173 | 0.085676173 | FALSE | Excluded |  |  |  |  |
| TZ48 The skin turns red when scratched and scratches appear :Sometimes | 0.085564889 | 0.085564889 | FALSE | Excluded |  |  |  |  |
| TZ22 Thick and greasy tongue coating :Often | 0.085142953 | 0.085142953 | FALSE | Excluded |  |  |  |  |
| TZ30 Be prone to dark circles :Often | 0.084461981 | 0.084461981 | FALSE | Excluded |  |  |  |  |
| TZ28 Where there is pain on the body :Sometimes | 0.082736401 | 0.082736401 | FALSE | Excluded |  |  |  |  |
| TZ34 Always moping :Sometimes | 0.082661426 | 0.082661426 | FALSE | Excluded |  |  |  |  |
| TZ16 Easily restless :Rarely | 0.081773593 | 0.081773593 | FALSE | Excluded |  |  |  |  |
| TZ12 Having cold or eating cold food makes it easy to have diarrhea :Sometimes | 0.081471407 | 0.081471407 | FALSE | Excluded |  |  |  |  |
| YSFY5 Children are rarely encouraged to taste new foods :Sometimes | 0.081290181 | 0.081290181 | FALSE | Excluded |  |  |  |  |
| YS1 Your child average meal time:>45min | 0.080422044 | 0.080422044 | FALSE | Excluded |  |  |  |  |
| TZ10 Afraid of cold than other children of the same age :Sometimes | 0.080376274 | 0.080376274 | FALSE | Excluded |  |  |  |  |
| TZ50 Can adapt to weather changes and new environment :Often | 0.079696031 | 0.079696031 | FALSE | Excluded |  |  |  |  |
| Height cm | -0.079055544 | 0.079055544 | FALSE | Excluded |  |  |  |  |
| The earliest age of using antibiotics 1 and 2 age | 0.078586788 | 0.078586788 | FALSE | Excluded |  |  |  |  |
| TZ40 Always worried about a lot of things :Often | 0.078418736 | 0.078418736 | FALSE | Excluded |  |  |  |  |
| TZ29 The skin on the body or the skin on the neck armpit or thigh root is dark :Sometimes | 0.077905399 | 0.077905399 | FALSE | Excluded |  |  |  |  |
| Activity habits Low activity levels | 0.077849154 | 0.077849154 | FALSE | Excluded |  |  |  |  |
| TZ50 Can adapt to weather changes and new environment :Sometimes | 0.077329203 | 0.077329203 | FALSE | Excluded |  |  |  |  |
| TZ2 Yellow complexion,no other children ruddy :Always | 0.077212686 | 0.077212686 | FALSE | Excluded |  |  |  |  |
| TZ25 Like to eat spicy or fried barbecue food :Often | 0.077183048 | 0.077183048 | FALSE | Excluded |  |  |  |  |
| Supplementing nutrients (such as vitamins) separately from daily diet | 0.077182255 | 0.077182255 | FALSE | Excluded |  |  |  |  |
| TZ45 Susceptible to allergies :Sometimes | 0.077006354 | 0.077006354 | FALSE | Excluded |  |  |  |  |
| Activity habits High activity levels | -0.076891914 | 0.076891914 | FALSE | Excluded |  |  |  |  |
| TZ14 Skin and lips dry easily :Often | 0.076851976 | 0.076851976 | FALSE | Excluded |  |  |  |  |
| YSRT5 Unwilling to try new food :Sometimes | 0.076103028 | 0.076103028 | FALSE | Excluded |  |  |  |  |
| TZ3 Soft muscles,not as strong as other children :Always | 0.075362886 | 0.075362886 | FALSE | Excluded |  |  |  |  |
| Functional dyspepsia | 0.075183908 | 0.075183908 | FALSE | Excluded |  |  |  |  |
| Which child of mother Second | -0.075002734 | 0.075002734 | FALSE | Excluded |  |  |  |  |
| TZ20 Like to eat sweet or greasy things :Always | 0.074906454 | 0.074906454 | FALSE | Excluded |  |  |  |  |
| YSFY4 Allow children to choose food at will :Sometimes | 0.074662118 | 0.074662118 | FALSE | Excluded |  |  |  |  |
| Functional constipation | 0.073989271 | 0.073989271 | FALSE | Excluded |  |  |  |  |
| TZ23 Always red eyes or easy to get gum :Often | 0.073766844 | 0.073766844 | FALSE | Excluded |  |  |  |  |
| TZ17 Being overweight and unwilling to exercise :Rarely | 0.07316932 | 0.07316932 | FALSE | Excluded |  |  |  |  |
| Which child of father second | -0.07307491 | 0.07307491 | FALSE | Excluded |  |  |  |  |
| TZ4 Always quiet :Rarely | 0.07302708 | 0.07302708 | FALSE | Excluded |  |  |  |  |
| Only child | -0.072906811 | 0.072906811 | FALSE | Excluded |  |  |  |  |
| TZ5 Speak in a low voice :Sometimes | 0.072758197 | 0.072758197 | FALSE | Excluded |  |  |  |  |
| TZ19 Always sleepy :Sometimes | 0.072692546 | 0.072692546 | FALSE | Excluded |  |  |  |  |
| Average sleep time per day h | -0.072232792 | 0.072232792 | FALSE | Excluded |  |  |  |  |
| YSFY7 Allow children to dine and wander around :Often | 0.071950574 | 0.071950574 | FALSE | Excluded |  |  |  |  |
| TZ33 Lips are dark or blue :Sometimes | 0.071875936 | 0.071875936 | FALSE | Excluded |  |  |  |  |
| The highest education of the child's mother Junior high school | -0.07173963 | 0.07173963 | FALSE | Excluded |  |  |  |  |
| TZ43 Without cold but have stuffy nose and runny nose :Often | 0.070854094 | 0.070854094 | FALSE | Excluded |  |  |  |  |
| TZ49 Always lively and strong :Sometimes | 0.069916068 | 0.069916068 | FALSE | Excluded |  |  |  |  |
| TZ49 Always lively and strong :Often | 0.069673971 | 0.069673971 | FALSE | Excluded |  |  |  |  |
| TZ26 Sweat is sticky :Often | 0.069437873 | 0.069437873 | FALSE | Excluded |  |  |  |  |
| Self control ability Not good at selfcontrol but can accept discipline | 0.069323034 | 0.069323034 | FALSE | Excluded |  |  |  |  |
| TZ25 Like to eat spicy or fried barbecue food :Rarely | 0.068648424 | 0.068648424 | FALSE | Excluded |  |  |  |  |
| Physical balance and flexibility in the last year Very good | -0.068411138 | 0.068411138 | FALSE | Excluded |  |  |  |  |
| TZ38 Always crying when in trouble :Always | 0.06834833 | 0.06834833 | FALSE | Excluded |  |  |  |  |
| TZ18 Abdominal obesity is flabby and soft :Sometimes | 0.06741946 | 0.06741946 | FALSE | Excluded |  |  |  |  |
| TZ46 The skin is prone to itching and urticaria :Often | 0.067118346 | 0.067118346 | FALSE | Excluded |  |  |  |  |
| TZ24 Eczema is easy to grow on the body :Often | 0.066177831 | 0.066177831 | FALSE | Excluded |  |  |  |  |
| The highest education of the child's mother Bachelor degree. | 0.06505782 | 0.06505782 | FALSE | Excluded |  |  |  |  |
| TZ29 The skin on the body or the skin on the neck armpit or thigh root is dark :Often | 0.064969048 | 0.064969048 | FALSE | Excluded |  |  |  |  |
| Age Month | -0.064947401 | 0.064947401 | FALSE | Excluded |  |  |  |  |
| Sleep time at night h | -0.064168378 | 0.064168378 | FALSE | Excluded |  |  |  |  |
| Frequency of antibiotic use within 1 year old None | -0.064066179 | 0.064066179 | FALSE | Excluded |  |  |  |  |
| The earliest age of using antibiotics Within 1 age | 0.064066179 | 0.064066179 | FALSE | Excluded |  |  |  |  |
| TZ31 Blue or dark gums :Sometimes | 0.063151954 | 0.063151954 | FALSE | Excluded |  |  |  |  |
| TZ4 Always quiet :Sometimes | 0.063139993 | 0.063139993 | FALSE | Excluded |  |  |  |  |
| TZ9 Resistant to summer but not to winter :Often | 0.062631049 | 0.062631049 | FALSE | Excluded |  |  |  |  |
| TZ21 Snoring at night :Often | 0.062476416 | 0.062476416 | FALSE | Excluded |  |  |  |  |
| Feed Formula milk powder | 0.061794234 | 0.061794234 | FALSE | Excluded |  |  |  |  |
| Exclusively breast fed within 6 months after birth | -0.061642053 | 0.061642053 | FALSE | Excluded |  |  |  |  |
| TZ18 Abdominal obesity is flabby and soft :Rarely | 0.061525771 | 0.061525771 | FALSE | Excluded |  |  |  |  |
| YS1 Your child average meal time:25-45min | 0.061136548 | 0.061136548 | FALSE | Excluded |  |  |  |  |
| TZ6 Not talkative :Sometimes | 0.061020385 | 0.061020385 | FALSE | Excluded |  |  |  |  |
| YSRT8 The dining place is not fixed :Always | 0.060747867 | 0.060747867 | FALSE | Excluded |  |  |  |  |
| TZ13 The body and face are always very hot :Often | 0.06071559 | 0.06071559 | FALSE | Excluded |  |  |  |  |
| Grade Senior class | -0.060340984 | 0.060340984 | FALSE | Excluded |  |  |  |  |
| TZ8 Hands and feet always lukewarm :Often | 0.058547411 | 0.058547411 | FALSE | Excluded |  |  |  |  |
| TZ11 Feel uncomfortable or afraid of eating cold food :Often | 0.05826646 | 0.05826646 | FALSE | Excluded |  |  |  |  |
| TZ27 The skin always appears blue and purple ecchymosis unconsciously :Often | 0.057966205 | 0.057966205 | FALSE | Excluded |  |  |  |  |
| YSFY6 Allow children to eat snacks if they want :Often | 0.057714338 | 0.057714338 | FALSE | Excluded |  |  |  |  |
| TZ48 The skin turns red when scratched and scratches appear :Often | 0.05752855 | 0.05752855 | FALSE | Excluded |  |  |  |  |
| TZ42 Sneez when do not have a cold :Often | 0.057291942 | 0.057291942 | FALSE | Excluded |  |  |  |  |
| TZ45 Susceptible to allergies :Often | 0.05694877 | 0.05694877 | FALSE | Excluded |  |  |  |  |
| Which child of mother Third | -0.056900333 | 0.056900333 | FALSE | Excluded |  |  |  |  |
| The highest education of the child's father Bachelor degree. | 0.056752085 | 0.056752085 | FALSE | Excluded |  |  |  |  |
| TZ30 Be prone to dark circles :Always | 0.056350041 | 0.056350041 | FALSE | Excluded |  |  |  |  |
| Physical balance and flexibility in the last year Acceptable | 0.056237619 | 0.056237619 | FALSE | Excluded |  |  |  |  |
| Which child of father third | -0.055600363 | 0.055600363 | FALSE | Excluded |  |  |  |  |
| TZ35 Easily nervous :Often | 0.055501613 | 0.055501613 | FALSE | Excluded |  |  |  |  |
| TZ39 Easily shy or timid :Always | 0.055479482 | 0.055479482 | FALSE | Excluded |  |  |  |  |
| TZ39 Easily shy or timid :Rarely | 0.055477574 | 0.055477574 | FALSE | Excluded |  |  |  |  |
| YSFY3 Induce children to eat :Always | 0.055055634 | 0.055055634 | FALSE | Excluded |  |  |  |  |
| TZ51 Easy to have nightmares or wake up from dreams :Often | 0.054971279 | 0.054971279 | FALSE | Excluded |  |  |  |  |
| TZ44 Cough and wheeze when the season changes the temperature changes or smells strange smell :Often | 0.054068501 | 0.054068501 | FALSE | Excluded |  |  |  |  |
| YSFY2 Force or punish children to eat more :Always | 0.053817345 | 0.053817345 | FALSE | Excluded |  |  |  |  |
| TZ16 Easily restless :Always | 0.053428666 | 0.053428666 | FALSE | Excluded |  |  |  |  |
| Grade Kindergarten class | 0.052470856 | 0.052470856 | FALSE | Excluded |  |  |  |  |
| TZ18 Abdominal obesity is flabby and soft :Often | 0.052082224 | 0.052082224 | FALSE | Excluded |  |  |  |  |
| The highest education of the child's father Junior high school | -0.052079332 | 0.052079332 | FALSE | Excluded |  |  |  |  |
| TZ15 Always excited :Always | 0.051646372 | 0.051646372 | FALSE | Excluded |  |  |  |  |
| Physical balance and flexibility in the last year Good | 0.050833328 | 0.050833328 | FALSE | Excluded |  |  |  |  |
| Age 6 | -0.050619931 | 0.050619931 | FALSE | Excluded |  |  |  |  |
| TZ32 Rough skin like fish scales :Sometimes | 0.050588577 | 0.050588577 | FALSE | Excluded |  |  |  |  |
| TZ12 Having cold or eating cold food makes it easy to have diarrhea :Often | 0.050393297 | 0.050393297 | FALSE | Excluded |  |  |  |  |
| TZ10 Afraid of cold than other children of the same age :Often | 0.049807428 | 0.049807428 | FALSE | Excluded |  |  |  |  |
| Children usually have the most contact with people outside kindergarten time Grandparents | 0.048188225 | 0.048188225 | FALSE | Excluded |  |  |  |  |
| TZ46 The skin is prone to itching and urticaria :Always | 0.047918868 | 0.047918868 | FALSE | Excluded |  |  |  |  |
| TZ18 Abdominal obesity is flabby and soft :Always | 0.047478643 | 0.047478643 | FALSE | Excluded |  |  |  |  |
| TZ47 Purpura has occurred in the skin due to allergies :Sometimes | 0.047334911 | 0.047334911 | FALSE | Excluded |  |  |  |  |
| TZ24 Eczema is easy to grow on the body :Always | 0.047173891 | 0.047173891 | FALSE | Excluded |  |  |  |  |
| TZ38 Always crying when in trouble :Rarely | 0.047108446 | 0.047108446 | FALSE | Excluded |  |  |  |  |
| Activity habits general activity levels | 0.046724962 | 0.046724962 | FALSE | Excluded |  |  |  |  |
| YSRT6 Have a strong preference for certain textures or certain types of food :Sometimes | 0.046224793 | 0.046224793 | FALSE | Excluded |  |  |  |  |
| Gender | -0.046044985 | 0.046044985 | FALSE | Excluded |  |  |  |  |
| Frequency of antibiotic use within 1 year old 3 Times or more | 0.04565494 | 0.04565494 | FALSE | Excluded |  |  |  |  |
| TZ50 Can adapt to weather changes and new environment :Rarely | 0.04550538 | 0.04550538 | FALSE | Excluded |  |  |  |  |
| TZ17 Being overweight and unwilling to exercise :Sometimes | 0.045308382 | 0.045308382 | FALSE | Excluded |  |  |  |  |
| Whether to start subject education(such as studying mathematics and English alone,etc.) | 0.045099682 | 0.045099682 | FALSE | Excluded |  |  |  |  |
| YSFY5 Children are rarely encouraged to taste new foods :Often | 0.045009489 | 0.045009489 | FALSE | Excluded |  |  |  |  |
| YSFY1 Few emotional exchanges during meals :Often | 0.044821082 | 0.044821082 | FALSE | Excluded |  |  |  |  |
| Daytime sleep time h | -0.044458655 | 0.044458655 | FALSE | Excluded |  |  |  |  |
| BMI | -0.043370709 | 0.043370709 | FALSE | Excluded |  |  |  |  |
| YSFY4 Allow children to choose food at will :Always | 0.043276934 | 0.043276934 | FALSE | Excluded |  |  |  |  |
| TZ40 Always worried about a lot of things :Always | 0.043239982 | 0.043239982 | FALSE | Excluded |  |  |  |  |
| TZ22 Thick and greasy tongue coating :Always | 0.043148552 | 0.043148552 | FALSE | Excluded |  |  |  |  |
| TZ37 Easily afraid or frightened :Always | 0.042789081 | 0.042789081 | FALSE | Excluded |  |  |  |  |
| TZ25 Like to eat spicy or fried barbecue food :Always | 0.042756453 | 0.042756453 | FALSE | Excluded |  |  |  |  |
| TZ5 Speak in a low voice :Often | 0.042445068 | 0.042445068 | FALSE | Excluded |  |  |  |  |
| TZ14 Skin and lips dry easily :Always | 0.042245212 | 0.042245212 | FALSE | Excluded |  |  |  |  |
| Age 4 | 0.041738863 | 0.041738863 | FALSE | Excluded |  |  |  |  |
| TZ31 Blue or dark gums :Often | 0.041724833 | 0.041724833 | FALSE | Excluded |  |  |  |  |
| Food allergy | 0.041690738 | 0.041690738 | FALSE | Excluded |  |  |  |  |
| YSRT1 Eat less than your peers :Sometimes | 0.041416974 | 0.041416974 | FALSE | Excluded |  |  |  |  |
| TZ29 The skin on the body or the skin on the neck,armpit or thigh root is dark :Always | 0.041384791 | 0.041384791 | FALSE | Excluded |  |  |  |  |
| TZ36 Easy to lack self confidence :Always | 0.040333896 | 0.040333896 | FALSE | Excluded |  |  |  |  |
| TZ41 More attached to you now than before :Always | 0.040102542 | 0.040102542 | FALSE | Excluded |  |  |  |  |
| TZ45 Susceptible to allergies :Always | 0.04001728 | 0.04001728 | FALSE | Excluded |  |  |  |  |
| TZ1 Easy to lose spirit :Often | 0.037735202 | 0.037735202 | FALSE | Excluded |  |  |  |  |
| Frequency of antibiotic use within 1 year old Twice | 0.037381107 | 0.037381107 | FALSE | Excluded |  |  |  |  |
| TZ6 Not talkative :Often | 0.03730661 | 0.03730661 | FALSE | Excluded |  |  |  |  |
| TZ11 Feel uncomfortable or afraid of eating cold food :Always | 0.037291884 | 0.037291884 | FALSE | Excluded |  |  |  |  |
| TZ7 Little activity makes sweating easily :Sometimes | 0.036817617 | 0.036817617 | FALSE | Excluded |  |  |  |  |
| TZ4 Always quiet :Often | 0.036575347 | 0.036575347 | FALSE | Excluded |  |  |  |  |
| Other digestive system diseases | 0.036257497 | 0.036257497 | FALSE | Excluded |  |  |  |  |
| TZ42 Sneez when do not have a cold :Always | 0.036045735 | 0.036045735 | FALSE | Excluded |  |  |  |  |
| TZ49 Always lively and strong :Rarely | 0.036032627 | 0.036032627 | FALSE | Excluded |  |  |  |  |
| The highest education of the child's father High school/Technical secondary school | -0.035889531 | 0.035889531 | FALSE | Excluded |  |  |  |  |
| The highest education of the child's mother High school/Technical secondary school | -0.035868729 | 0.035868729 | FALSE | Excluded |  |  |  |  |
| TZ32 Rough skin like fish scales :Often | 0.035792176 | 0.035792176 | FALSE | Excluded |  |  |  |  |
| Mesenteric lymphadenitis | 0.035420585 | 0.035420585 | FALSE | Excluded |  |  |  |  |
| TZ28 Where there is pain on the body :Often | 0.035260265 | 0.035260265 | FALSE | Excluded |  |  |  |  |
| TZ26 Sweat is sticky :Always | 0.034850419 | 0.034850419 | FALSE | Excluded |  |  |  |  |
| TZ27 The skin always appears blue and purple ecchymosis unconsciously :Always | 0.034713666 | 0.034713666 | FALSE | Excluded |  |  |  |  |
| TZ9 Resistant to summer but not to winter :Always | 0.034443798 | 0.034443798 | FALSE | Excluded |  |  |  |  |
| TZ43 Without cold but have stuffy nose and runny nose :Always | 0.03420124 | 0.03420124 | FALSE | Excluded |  |  |  |  |
| TZ19 Always sleepy :Often | 0.033577003 | 0.033577003 | FALSE | Excluded |  |  |  |  |
| TZ15 Always excited :Rarely | 0.033383769 | 0.033383769 | FALSE | Excluded |  |  |  |  |
| TZ31 Blue or dark gums :Always | 0.03261315 | 0.03261315 | FALSE | Excluded |  |  |  |  |
| TZ23 Always red eyes or easy to get gum :Always | 0.03242776 | 0.03242776 | FALSE | Excluded |  |  |  |  |
| TZ21 Snoring at night :Always | 0.031999947 | 0.031999947 | FALSE | Excluded |  |  |  |  |
| TZ33 Lips are dark or blue :Often | 0.031695433 | 0.031695433 | FALSE | Excluded |  |  |  |  |
| YSFY7 Allow children to dine and wander around :Always | 0.031197319 | 0.031197319 | FALSE | Excluded |  |  |  |  |
| TZ8 Hands and feet always lukewarm :Always | 0.031151661 | 0.031151661 | FALSE | Excluded |  |  |  |  |
| Language comprehension and expression ability Expression Not yet clear | 0.030785921 | 0.030785921 | FALSE | Excluded |  |  |  |  |
| TZ34 Always moping :Often | 0.030366999 | 0.030366999 | FALSE | Excluded |  |  |  |  |
| The earliest age of using antibiotics After 3 age | 0.030363031 | 0.030363031 | FALSE | Excluded |  |  |  |  |
| TZ44 Cough and wheeze when the season changes the temperature changes or smells strange smell :Always | 0.03005838 | 0.03005838 | FALSE | Excluded |  |  |  |  |
| Frequency of antibiotic use within 1 year old Once | 0.029806241 | 0.029806241 | FALSE | Excluded |  |  |  |  |
| Which child of mother Fourth and after the fourth | -0.029730623 | 0.029730623 | FALSE | Excluded |  |  |  |  |
| TZ17 Being overweight and unwilling to exercise :Often | 0.029408485 | 0.029408485 | FALSE | Excluded |  |  |  |  |
| TZ20 Like to eat sweet or greasy things :Rarely | 0.028536183 | 0.028536183 | FALSE | Excluded |  |  |  |  |
| Age 3 | 0.027998271 | 0.027998271 | FALSE | Excluded |  |  |  |  |
| With dental caries | 0.027664072 | 0.027664072 | FALSE | Excluded |  |  |  |  |
| TZ13 The body and face are always very hot :Always | 0.027349545 | 0.027349545 | FALSE | Excluded |  |  |  |  |
| TZ47 Purpura has occurred in the skin due to allergies :Often | 0.027220354 | 0.027220354 | FALSE | Excluded |  |  |  |  |
| TZ10 Afraid of cold than other children of the same age :Always | 0.027093732 | 0.027093732 | FALSE | Excluded |  |  |  |  |
| TZ35 Easily nervous :Always | 0.0264642 | 0.0264642 | FALSE | Excluded |  |  |  |  |
| Children usually have the most contact with people outside kindergarten time Mother | -0.02623185 | 0.02623185 | FALSE | Excluded |  |  |  |  |
| TZ48 The skin turns red when scratched and scratches appear :Always | 0.026173205 | 0.026173205 | FALSE | Excluded |  |  |  |  |
| TZ51 Easy to have nightmares or wake up from dreams :Always | 0.025261626 | 0.025261626 | FALSE | Excluded |  |  |  |  |
| Which child of father Fourth and After the fourth | -0.024734321 | 0.024734321 | FALSE | Excluded |  |  |  |  |
| gastritis | 0.02455352 | 0.02455352 | FALSE | Excluded |  |  |  |  |
| TZ32 Rough skin like fish scales :Always | 0.023303994 | 0.023303994 | FALSE | Excluded |  |  |  |  |
| Other allergic history | 0.022335851 | 0.022335851 | FALSE | Excluded |  |  |  |  |
| YSFY6 Allow children to eat snacks if they want :Always | 0.021993947 | 0.021993947 | FALSE | Excluded |  |  |  |  |
| Normal vision | -0.021442402 | 0.021442402 | FALSE | Excluded |  |  |  |  |
| Whether there is abnormal vision | 0.021442402 | 0.021442402 | FALSE | Excluded |  |  |  |  |
| The highest education of the child's mother Associate degree | 0.021058295 | 0.021058295 | FALSE | Excluded |  |  |  |  |
| Per capita monthly income of families Between 1000 and 2000RMB | -0.02087485 | 0.02087485 | FALSE | Excluded |  |  |  |  |
| TZ7 Little activity makes sweating easily :Rarely | -0.020778808 | 0.020778808 | FALSE | Excluded |  |  |  |  |
| Other visual abnormalities | 0.02063046 | 0.02063046 | FALSE | Excluded |  |  |  |  |
| Drug allergy | 0.020571371 | 0.020571371 | FALSE | Excluded |  |  |  |  |
| TZ12 Having cold or eating cold food makes it easy to have diarrhea :Always | 0.020166668 | 0.020166668 | FALSE | Excluded |  |  |  |  |
| Feed Others | 0.020149894 | 0.020149894 | FALSE | Excluded |  |  |  |  |
| Language comprehension and expression ability Respond with complex sentence | -0.020017685 | 0.020017685 | FALSE | Excluded |  |  |  |  |
| Birth weight More than 4kg | -0.020009098 | 0.020009098 | FALSE | Excluded |  |  |  |  |
| YSRT2 Eating slowly compared with peers :Sometimes | -0.019743117 | 0.019743117 | FALSE | Excluded |  |  |  |  |
| TZ33 Lips are dark or blue :Always | 0.018934192 | 0.018934192 | FALSE | Excluded |  |  |  |  |
| Like music or not Like | 0.018446808 | 0.018446808 | FALSE | Excluded |  |  |  |  |
| TZ19 Always sleepy :Always | 0.018431926 | 0.018431926 | FALSE | Excluded |  |  |  |  |
| YSFY5 Children are rarely encouraged to taste new foods :Always | 0.018414508 | 0.018414508 | FALSE | Excluded |  |  |  |  |
| TZ28 Where there is pain on the body :Always | 0.0176925 | 0.0176925 | FALSE | Excluded |  |  |  |  |
| Time of adding animal protein complementary food within 3 months | -0.017651628 | 0.017651628 | FALSE | Excluded |  |  |  |  |
| TZ5 Speak in a low voice :Always | 0.017149041 | 0.017149041 | FALSE | Excluded |  |  |  |  |
| History of major diseases | 0.017128228 | 0.017128228 | FALSE | Excluded |  |  |  |  |
| Birth weight 2.5-4 kg | 0.017018079 | 0.017018079 | FALSE | Excluded |  |  |  |  |
| The highest education of the child's mother Below elementary school | -0.016956066 | 0.016956066 | FALSE | Excluded |  |  |  |  |
| Astigmatism | 0.016771387 | 0.016771387 | FALSE | Excluded |  |  |  |  |
| The highest education of the child's mother Graduate degree. | 0.016684829 | 0.016684829 | FALSE | Excluded |  |  |  |  |
| Who educates children the longest in the family at ordinary times Mother | 0.016410479 | 0.016410479 | FALSE | Excluded |  |  |  |  |
| YSFY1 Few emotional exchanges during meals :Always | 0.016332644 | 0.016332644 | FALSE | Excluded |  |  |  |  |
| Activity habits inactivity | 0.016200248 | 0.016200248 | FALSE | Excluded |  |  |  |  |
| Dental caries have been treated usually | 0.016165269 | 0.016165269 | FALSE | Excluded |  |  |  |  |
| The highest education of the child's mother Elementary school | -0.016069147 | 0.016069147 | FALSE | Excluded |  |  |  |  |
| Gastroesophageal reflux | 0.015533661 | 0.015533661 | FALSE | Excluded |  |  |  |  |
| Who educates children the longest in the family at ordinary times Father | -0.015329247 | 0.015329247 | FALSE | Excluded |  |  |  |  |
| Term infant | -0.01524078 | 0.01524078 | FALSE | Excluded |  |  |  |  |
| Per capita monthly income of families Between 10000 and 20000RMB | 0.01507372 | 0.01507372 | FALSE | Excluded |  |  |  |  |
| The highest education of the child's father Graduate degree. | 0.013128591 | 0.013128591 | FALSE | Excluded |  |  |  |  |
| Like music or not Resistance | -0.012746555 | 0.012746555 | FALSE | Excluded |  |  |  |  |
| Children usually have the most contact with people outside kindergarten time Father/Mother | -0.01260874 | 0.01260874 | FALSE | Excluded |  |  |  |  |
| Dental caries have been treated None | -0.012189988 | 0.012189988 | FALSE | Excluded |  |  |  |  |
| Time of birth: 21:00-23:00 | 0.011729309 | 0.011729309 | FALSE | Excluded |  |  |  |  |
| TZ17 Being overweight and unwilling to exercise :Always | 0.01172651 | 0.01172651 | FALSE | Excluded |  |  |  |  |
| TZ6 Not talkative :Always | 0.011451152 | 0.011451152 | FALSE | Excluded |  |  |  |  |
| TZ4 Always quiet :Always | 0.011422798 | 0.011422798 | FALSE | Excluded |  |  |  |  |
| Amblyopia | 0.011093552 | 0.011093552 | FALSE | Excluded |  |  |  |  |
| Like music or not Numb | -0.011080961 | 0.011080961 | FALSE | Excluded |  |  |  |  |
| Age 5 | -0.010954405 | 0.010954405 | FALSE | Excluded |  |  |  |  |
| Time of birth 9:00-11:00 | -0.010636348 | 0.010636348 | FALSE | Excluded |  |  |  |  |
| Grade Middle class | 0.010231348 | 0.010231348 | FALSE | Excluded |  |  |  |  |
| TZ47 Purpura has occurred in the skin due to allergies :Always | 0.009987045 | 0.009987045 | FALSE | Excluded |  |  |  |  |
| Children usually have the most contact with people outside kindergarten time Father | -0.009936075 | 0.009936075 | FALSE | Excluded |  |  |  |  |
| Per capita monthly income of families Lower than 1000RMB | -0.009840262 | 0.009840262 | FALSE | Excluded |  |  |  |  |
| Per capita monthly income of families Between 2000 and 5000RMB | -0.009283602 | 0.009283602 | FALSE | Excluded |  |  |  |  |
| Have vision correction therapy | 0.009173651 | 0.009173651 | FALSE | Excluded |  |  |  |  |
| YS2 Do you think your child has a picky eater problem:Do not care | 0.009108812 | 0.009108812 | FALSE | Excluded |  |  |  |  |
| The highest education of the child's father Associate degree | 0.008911428 | 0.008911428 | FALSE | Excluded |  |  |  |  |
| Age 7 | -0.008837725 | 0.008837725 | FALSE | Excluded |  |  |  |  |
| The age of the father when the child is born | -0.008785348 | 0.008785348 | FALSE | Excluded |  |  |  |  |
| TZ1 Easy to lose spirit :Always | 0.008628895 | 0.008628895 | FALSE | Excluded |  |  |  |  |
| Language comprehension and expression ability Respond clearly | 0.008466076 | 0.008466076 | FALSE | Excluded |  |  |  |  |
| Time of adding animal protein complementary food After 6th months | 0.007930176 | 0.007930176 | FALSE | Excluded |  |  |  |  |
| Like music or not Favorite | -0.007655465 | 0.007655465 | FALSE | Excluded |  |  |  |  |
| Feed Fresh ewe milk | -0.007452436 | 0.007452436 | FALSE | Excluded |  |  |  |  |
| The highest education of the child's father Below elementary school | -0.007046561 | 0.007046561 | FALSE | Excluded |  |  |  |  |
| Caesarean birth | -0.007045664 | 0.007045664 | FALSE | Excluded |  |  |  |  |
| short sighted | 0.006997627 | 0.006997627 | FALSE | Excluded |  |  |  |  |
| Who educates children the longest in the family at ordinary times Others | -0.00675547 | 0.00675547 | FALSE | Excluded |  |  |  |  |
| Grade other | -0.00675547 | 0.00675547 | FALSE | Excluded |  |  |  |  |
| YSRT7 Distraction from eating :Sometimes | -0.006749713 | 0.006749713 | FALSE | Excluded |  |  |  |  |
| Time of birth: 23:00-1:00 | 0.006474124 | 0.006474124 | FALSE | Excluded |  |  |  |  |
| unclear | 0.006464017 | 0.006464017 | FALSE | Excluded |  |  |  |  |
| Time of birth 5:00-7:00 | -0.00624594 | 0.00624594 | FALSE | Excluded |  |  |  |  |
| Like music or not Dislike | -0.006167176 | 0.006167176 | FALSE | Excluded |  |  |  |  |
| Dental caries have been treated occasionally | 0.005986674 | 0.005986674 | FALSE | Excluded |  |  |  |  |
| Peptic ulcer | 0.00514308 | 0.00514308 | FALSE | Excluded |  |  |  |  |
| Time of birth: 3:00-5:00 | -0.004992665 | 0.004992665 | FALSE | Excluded |  |  |  |  |
| Time of birth: 13:00-15:00 | 0.004857622 | 0.004857622 | FALSE | Excluded |  |  |  |  |
| Birth weight lower than 2.5 kg | -0.004750966 | 0.004750966 | FALSE | Excluded |  |  |  |  |
| Time of birth 17:00-19:00 | 0.003940963 | 0.003940963 | FALSE | Excluded |  |  |  |  |
| TZ50 Can adapt to weather changes and new environment :Always | -0.003921849 | 0.003921849 | FALSE | Excluded |  |  |  |  |
| Who educates children the longest in the family at ordinary times Parents and Grandparents | -0.003821027 | 0.003821027 | FALSE | Excluded |  |  |  |  |
| Time of adding animal protein complementary food between 4th and 6th months | -0.003755696 | 0.003755696 | FALSE | Excluded |  |  |  |  |
| Time of birth :7:00-9:00 | -0.003605974 | 0.003605974 | FALSE | Excluded |  |  |  |  |
| Ethnic group | 0.003586782 | 0.003586782 | FALSE | Excluded |  |  |  |  |
| TZ34 Always moping :Always | 0.003498212 | 0.003498212 | FALSE | Excluded |  |  |  |  |
| Who educates children the longest in the family at ordinary times Parents | -0.003291494 | 0.003291494 | FALSE | Excluded |  |  |  |  |
| Feed Fresh milk | -0.003052566 | 0.003052566 | FALSE | Excluded |  |  |  |  |
| Feed Ewe milk power | 0.002898907 | 0.002898907 | FALSE | Excluded |  |  |  |  |
| The highest education of the child's father Elementary school | -0.002773284 | 0.002773284 | FALSE | Excluded |  |  |  |  |
| Time of birth: 19:00-21:00 | 0.002593903 | 0.002593903 | FALSE | Excluded |  |  |  |  |
| Children usually have the most contact with people outside kindergarten time Others | -0.002025887 | 0.002025887 | FALSE | Excluded |  |  |  |  |
| Per capita monthly income of families Higher than 20000RMB | 0.001862628 | 0.001862628 | FALSE | Excluded |  |  |  |  |
| Time of birth: 11:00-13:00 | 0.001839456 | 0.001839456 | FALSE | Excluded |  |  |  |  |
| Children usually have the most contact with people outside kindergarten time Parents and Grandparents | 0.001798025 | 0.001798025 | FALSE | Excluded |  |  |  |  |
| Per capita monthly income of families Between 5000 and 10000RMB | 0.001754845 | 0.001754845 | FALSE | Excluded |  |  |  |  |
| TZ49 Always lively and strong :Always | -0.000839457 | 0.000839457 | FALSE | Excluded |  |  |  |  |
| The age of the mother when the child is born | -0.000789221 | 0.000789221 | FALSE | Excluded |  |  |  |  |
| Time of birth: 15:00-17:00 | 0.000394668 | 0.000394668 | FALSE | Excluded |  |  |  |  |
| Who educates children the longest in the family at ordinary times Grandparents | -0.000372462 | 0.000372462 | FALSE | Excluded |  |  |  |  |
| Time of birth: 1:00-3:00 | 0.00022506 | 0.00022506 | FALSE | Excluded |  |  |  |  |
| Feed soymilk | -0.000023 | 0.000023 | FALSE | Excluded |  |  |  |  |

**Table S5. Hyperparameter grid and selected values**

| Model name | Hyperparameter search grid range | Optimal hyperparameters selected during training |
| --- | --- | --- |
| DT | {'model__max_depth': [4, 6, 8, 10], 'model__min_samples_split': [2, 5, 10], 'model__min_samples_leaf': [1, 2, 4], 'model__max_features': ['sqrt', 'log2', None], 'model__criterion': ['gini', 'entropy']} | {'model__criterion': 'gini', 'model__max_depth': 8, 'model__max_features': 'sqrt', 'model__min_samples_leaf': 1, 'model__min_samples_split': 5} |
| KNN | {'model__n_neighbors': [3, 5, 7, 9], 'model__weights': ['uniform', 'distance'], 'model__p': [1, 2]} | {'model__n_neighbors': 9, 'model__p': 2, 'model__weights': 'uniform'} |
| LinearSVC | {'model__C': [0.01, 0.1, 1, 10], 'model__penalty': ['l1', 'l2'], 'model__loss': ['squared_hinge']} | {'model__C': 0.01, 'model__loss': 'squared_hinge', 'model__penalty': 'l1'} |
| LR | {'model__C': [0.01, 0.1, 1, 10], 'model__penalty': ['l1', 'l2'], 'model__solver': ['saga']} | {'model__C': 0.01, 'model__penalty': 'l2', 'model__solver': 'saga'} |
| NB | No hyperparameter search grid | {} |
| RF | {'model__n_estimators': [50, 100, 200], 'model__max_depth': [6, 8, 10], 'model__min_samples_split': [2, 5]} | {'model__max_depth': 10, 'model__min_samples_split': 5, 'model__n_estimators': 200} |
| XGBoost | {'model__n_estimators': [50, 100], 'model__max_depth': [3, 5, 7], 'model__learning_rate': [0.01, 0.1]} | {'model__learning_rate': 0.1, 'model__max_depth': 3, 'model__n_estimators': 100} |

**Table S6. Confidence Intervals for DCA Net Benefit**

| Model | Threshold | Net  Benefit | NB 95CI Lower | NB  95CI  Upper | Model | Threshold | Net  Benefit | NB 95CI Lower | NB  95CI  Upper |
| --- | --- | --- | --- | --- | --- | --- | --- | --- | --- |
| DT | 0.01 | 0.4584 | 0 | 0.4473 | KNN | 0.01 | 0.4584 | 0 | 0.4473 |
| DT | 0.02 | 0.4529 | 0 | 0.4473 | KNN | 0.02 | 0.4529 | 0 | 0.4473 |
| DT | 0.03 | 0.4473 | 0 | 0.4473 | KNN | 0.03 | 0.4473 | 0 | 0.4473 |
| DT | 0.04 | 0.4415 | 0 | 0.4473 | KNN | 0.04 | 0.4415 | 0 | 0.4473 |
| DT | 0.05 | 0.4356 | 0 | 0.4473 | KNN | 0.05 | 0.4356 | 0 | 0.4474 |
| DT | 0.06 | 0.4296 | 0 | 0.4473 | KNN | 0.06 | 0.4296 | 0 | 0.4529 |
| DT | 0.07 | 0.4235 | 0 | 0.4474 | KNN | 0.07 | 0.4235 | 0 | 0.4473 |
| DT | 0.08 | 0.4172 | 0 | 0.4473 | KNN | 0.08 | 0.4172 | 0 | 0.4473 |
| DT | 0.09 | 0.4108 | 0 | 0.4473 | KNN | 0.09 | 0.4108 | 0 | 0.4473 |
| DT | 0.1 | 0.4043 | 0 | 0.4473 | KNN | 0.1 | 0.4043 | 0 | 0.4473 |
| DT | 0.11 | 0.3976 | 0 | 0.4473 | KNN | 0.11 | 0.3976 | 0 | 0.4473 |
| DT | 0.12 | 0.3907 | 0 | 0.4529 | KNN | 0.12 | 0.3907 | 0 | 0.4473 |
| DT | 0.13 | 0.3837 | 0 | 0.4529 | KNN | 0.13 | 0.3837 | 0 | 0.4473 |
| DT | 0.14 | 0.3766 | 0 | 0.4473 | KNN | 0.14 | 0.3766 | 0 | 0.4529 |
| DT | 0.15 | 0.3692 | 0 | 0.4473 | KNN | 0.15 | 0.3692 | 0 | 0.4473 |
| DT | 0.16 | 0.3617 | 0 | 0.4529 | KNN | 0.16 | 0.3617 | 0 | 0.4473 |
| DT | 0.17 | 0.3541 | 0 | 0.4473 | KNN | 0.17 | 0.354 | 0 | 0.4529 |
| DT | 0.18 | 0.3462 | 0 | 0.4473 | KNN | 0.18 | 0.3462 | 0 | 0.4473 |
| DT | 0.19 | 0.3383 | 0 | 0.4473 | KNN | 0.19 | 0.3381 | 0 | 0.4473 |
| DT | 0.2 | 0.3302 | 0 | 0.4529 | KNN | 0.2 | 0.3298 | 0 | 0.4473 |
| DT | 0.21 | 0.3218 | 0 | 0.4415 | KNN | 0.21 | 0.3213 | 0 | 0.4473 |
| DT | 0.22 | 0.3133 | 0 | 0.4473 | KNN | 0.22 | 0.3126 | 0 | 0.4473 |
| DT | 0.23 | 0.3044 | 0 | 0.4473 | KNN | 0.23 | 0.3037 | 0 | 0.4473 |
| DT | 0.24 | 0.3039 | 0 | 0.4473 | KNN | 0.24 | 0.3043 | 0 | 0.4529 |
| DT | 0.25 | 0.296 | 0 | 0.4473 | KNN | 0.25 | 0.2954 | 0 | 0.4473 |
| DT | 0.26 | 0.2876 | 0 | 0.4415 | KNN | 0.26 | 0.2915 | 0 | 0.4473 |
| DT | 0.27 | 0.2871 | 0 | 0.4473 | KNN | 0.27 | 0.2824 | 0 | 0.4473 |
| DT | 0.28 | 0.2795 | 0 | 0.4473 | KNN | 0.28 | 0.2799 | 0 | 0.4415 |
| DT | 0.29 | 0.2742 | 0 | 0.4473 | KNN | 0.29 | 0.2777 | 0 | 0.4529 |
| DT | 0.3 | 0.2665 | 0 | 0.4473 | KNN | 0.3 | 0.2689 | 0 | 0.4529 |
| DT | 0.31 | 0.2621 | 0 | 0.4473 | KNN | 0.31 | 0.2729 | 0 | 0.4473 |
| DT | 0.32 | 0.2559 | 0 | 0.4473 | KNN | 0.32 | 0.2646 | 0 | 0.4473 |
| DT | 0.33 | 0.2509 | 0 | 0.4473 | KNN | 0.33 | 0.2647 | 0 | 0.4529 |
| DT | 0.34 | 0.246 | 0 | 0.4473 | KNN | 0.34 | 0.2605 | 0 | 0.4473 |
| DT | 0.35 | 0.2399 | 0 | 0.4473 | KNN | 0.35 | 0.2538 | 0 | 0.4473 |
| DT | 0.36 | 0.2339 | 0 | 0.4417 | KNN | 0.36 | 0.2496 | 0 | 0.4473 |
| DT | 0.37 | 0.2275 | 0 | 0.4473 | KNN | 0.37 | 0.2422 | 0 | 0.4415 |
| DT | 0.38 | 0.2207 | 0 | 0.4473 | KNN | 0.38 | 0.2419 | 0 | 0.4473 |
| DT | 0.39 | 0.2153 | 0 | 0.4473 | KNN | 0.39 | 0.2359 | 0 | 0.4415 |
| DT | 0.4 | 0.2086 | 0 | 0.4529 | KNN | 0.4 | 0.2321 | 0 | 0.4415 |
| DT | 0.41 | 0.2033 | 0 | 0.4473 | KNN | 0.41 | 0.2271 | 0 | 0.4473 |
| DT | 0.42 | 0.1964 | 0 | 0.4529 | KNN | 0.42 | 0.221 | 0 | 0.4473 |
| DT | 0.43 | 0.1901 | 0 | 0.4415 | KNN | 0.43 | 0.2104 | 0 | 0.4473 |
| DT | 0.44 | 0.184 | 0 | 0.4415 | KNN | 0.44 | 0.2045 | 0 | 0.4473 |
| DT | 0.45 | 0.178 | 0 | 0.4473 | KNN | 0.45 | 0.1987 | 0 | 0.4473 |
| DT | 0.46 | 0.1734 | 0 | 0.4417 | KNN | 0.46 | 0.1918 | 0 | 0.4473 |
| DT | 0.47 | 0.1685 | 0 | 0.4473 | KNN | 0.47 | 0.1862 | 0 | 0.4415 |
| DT | 0.48 | 0.1641 | 0 | 0.4473 | KNN | 0.48 | 0.1792 | 0 | 0.4473 |
| DT | 0.49 | 0.1578 | 0 | 0.4473 | KNN | 0.49 | 0.1703 | 0 | 0.4473 |
| DT | 0.5 | 0.1529 | 0 | 0.4473 | KNN | 0.5 | 0.1656 | 0 | 0.4473 |
| DT | 0.51 | 0.1477 | 0 | 0.4473 | KNN | 0.51 | 0.158 | 0 | 0.4473 |
| DT | 0.52 | 0.1413 | 0 | 0.4473 | KNN | 0.52 | 0.1517 | 0 | 0.4415 |
| DT | 0.53 | 0.1337 | 0 | 0.4473 | KNN | 0.53 | 0.1473 | 0 | 0.4473 |
| DT | 0.54 | 0.1267 | 0 | 0.4473 | KNN | 0.54 | 0.14 | 0 | 0.4415 |
| DT | 0.55 | 0.1201 | 0 | 0.4473 | KNN | 0.55 | 0.1365 | 0 | 0.4529 |
| DT | 0.56 | 0.1161 | 0 | 0.4473 | KNN | 0.56 | 0.1324 | 0 | 0.4473 |
| DT | 0.57 | 0.1093 | 0 | 0.4417 | KNN | 0.57 | 0.1205 | 0 | 0.4473 |
| DT | 0.58 | 0.1038 | 0 | 0.4473 | KNN | 0.58 | 0.1165 | 0 | 0.4415 |
| DT | 0.59 | 0.0986 | 0 | 0.4473 | KNN | 0.59 | 0.1085 | 0 | 0.4473 |
| DT | 0.6 | 0.0949 | 0 | 0.4415 | KNN | 0.6 | 0.1026 | 0 | 0.4473 |
| DT | 0.61 | 0.0888 | 0 | 0.4473 | KNN | 0.61 | 0.0983 | 0 | 0.4473 |
| DT | 0.62 | 0.0829 | 0 | 0.4473 | KNN | 0.62 | 0.0919 | 0 | 0.4473 |
| DT | 0.63 | 0.077 | 0 | 0.4473 | KNN | 0.63 | 0.0901 | 0 | 0.4473 |
| DT | 0.64 | 0.0724 | 0 | 0.4415 | KNN | 0.64 | 0.0842 | 0 | 0.4473 |
| DT | 0.65 | 0.0672 | 0 | 0.4529 | KNN | 0.65 | 0.0787 | 0 | 0.4473 |
| DT | 0.66 | 0.062 | 0 | 0.4473 | KNN | 0.66 | 0.0749 | 0 | 0.4473 |
| DT | 0.67 | 0.0592 | 0 | 0.4473 | KNN | 0.67 | 0.0679 | 0 | 0.4473 |
| DT | 0.68 | 0.0548 | 0 | 0.4415 | KNN | 0.68 | 0.0659 | 0 | 0.4473 |
| DT | 0.69 | 0.051 | 0 | 0.4415 | KNN | 0.69 | 0.0556 | 0 | 0.4473 |
| DT | 0.7 | 0.0441 | 0 | 0.4473 | KNN | 0.7 | 0.0537 | 0 | 0.4473 |
| DT | 0.71 | 0.0377 | 0 | 0.4473 | KNN | 0.71 | 0.0471 | 0 | 0.4473 |
| DT | 0.72 | 0.0322 | 0 | 0.4473 | KNN | 0.72 | 0.0433 | 0 | 0.4473 |
| DT | 0.73 | 0.0192 | 0 | 0.4473 | KNN | 0.73 | 0.0398 | 0 | 0.4473 |
| DT | 0.74 | 0.0152 | 0 | 0.4529 | KNN | 0.74 | 0.0327 | 0 | 0.4473 |
| DT | 0.75 | 0.0035 | 0 | 0.4529 | KNN | 0.75 | 0.0308 | 0 | 0.4473 |
| DT | 0.76 | 0.0031 | 0 | 0.4415 | KNN | 0.76 | 0.0253 | 0 | 0.4473 |
| DT | 0.77 | 0.0028 | 0 | 0.4529 | KNN | 0.77 | 0.0227 | 0 | 0.4529 |
| DT | 0.78 | 0.0024 | 0 | 0.4529 | KNN | 0.78 | 0.0169 | 0 | 0.4473 |
| DT | 0.79 | 0.0009 | 0 | 0.4473 | KNN | 0.79 | 0.015 | 0 | 0.4473 |
| DT | 0.8 | 0.0007 | 0 | 0.4473 | KNN | 0.8 | 0.0104 | 0 | 0.4473 |
| DT | 0.81 | 0.0004 | 0 | 0.4473 | KNN | 0.81 | 0.0091 | 0 | 0.4473 |
| DT | 0.82 | 0.0004 | 0 | 0.4417 | KNN | 0.82 | 0.0072 | 0 | 0.4473 |
| DT | 0.83 | 0 | 0 | 0.4529 | KNN | 0.83 | 0.0034 | 0 | 0.4473 |
| DT | 0.84 | 0 | 0 | 0.4473 | KNN | 0.84 | 0.0034 | 0 | 0.4473 |
| DT | 0.85 | 0 | 0 | 0.4474 | KNN | 0.85 | 0.0016 | 0 | 0.4529 |
| DT | 0.86 | 0 | 0 | 0.4417 | KNN | 0.86 | 0 | 0 | 0.4473 |
| DT | 0.87 | 0 | 0 | 0.4415 | KNN | 0.87 | 0 | 0 | 0.4473 |
| DT | 0.88 | 0 | 0 | 0.4473 | KNN | 0.88 | 0 | 0 | 0.4473 |
| DT | 0.89 | 0 | 0 | 0.4529 | KNN | 0.89 | 0 | 0 | 0.4473 |
| DT | 0.9 | 0 | 0 | 0.4473 | KNN | 0.9 | 0 | 0 | 0.4473 |
| DT | 0.91 | 0 | 0 | 0.4473 | KNN | 0.91 | 0 | 0 | 0.4473 |
| DT | 0.92 | 0 | 0 | 0.4473 | KNN | 0.92 | 0 | 0 | 0.4415 |
| DT | 0.93 | 0 | 0 | 0.4529 | KNN | 0.93 | 0 | 0 | 0.4415 |
| DT | 0.94 | 0 | 0 | 0.4473 | KNN | 0.94 | 0 | 0 | 0.4473 |
| DT | 0.95 | 0 | 0 | 0.4473 | KNN | 0.95 | 0 | 0 | 0.4415 |
| DT | 0.96 | 0 | 0 | 0.4473 | KNN | 0.96 | 0 | 0 | 0.4473 |
| DT | 0.97 | 0 | 0 | 0.4415 | KNN | 0.97 | 0 | 0 | 0.4473 |
| DT | 0.98 | 0 | 0 | 0.4473 | KNN | 0.98 | 0 | 0 | 0.4473 |
| DT | 0.99 | 0 | 0 | 0.4473 | KNN | 0.99 | 0 | 0 | 0.4473 |
|  |  |  |  |  |  |  |  |  |  |
| LinearSVC | 0.01 | 0.4584 | 0 | 0.4473 | LR | 0.01 | 0.4584 | 0.0001 | 0.4417 |
| LinearSVC | 0.02 | 0.4529 | 0 | 0.4473 | LR | 0.02 | 0.4529 | 0.0005 | 0.4473 |
| LinearSVC | 0.03 | 0.4473 | 0 | 0.4415 | LR | 0.03 | 0.4473 | 0.0001 | 0.4473 |
| LinearSVC | 0.04 | 0.4415 | 0 | 0.4473 | LR | 0.04 | 0.4415 | 0.0001 | 0.4473 |
| LinearSVC | 0.05 | 0.4356 | 0 | 0.4529 | LR | 0.05 | 0.4356 | 0.0005 | 0.4473 |
| LinearSVC | 0.06 | 0.4296 | 0 | 0.4473 | LR | 0.06 | 0.4296 | 0.0001 | 0.4415 |
| LinearSVC | 0.07 | 0.4235 | 0 | 0.4473 | LR | 0.07 | 0.4235 | 0.0005 | 0.4473 |
| LinearSVC | 0.08 | 0.4172 | 0 | 0.4474 | LR | 0.08 | 0.4172 | 0.0005 | 0.4473 |
| LinearSVC | 0.09 | 0.4108 | 0 | 0.4473 | LR | 0.09 | 0.4108 | 0.0001 | 0.4473 |
| LinearSVC | 0.1 | 0.4043 | 0 | 0.4473 | LR | 0.1 | 0.4043 | 0 | 0.4473 |
| LinearSVC | 0.11 | 0.3976 | 0 | 0.4473 | LR | 0.11 | 0.3976 | 0.0001 | 0.4529 |
| LinearSVC | 0.12 | 0.3907 | 0 | 0.4473 | LR | 0.12 | 0.3907 | 0.0005 | 0.4473 |
| LinearSVC | 0.13 | 0.3837 | 0 | 0.4473 | LR | 0.13 | 0.3838 | 0.0001 | 0.4529 |
| LinearSVC | 0.14 | 0.3772 | 0 | 0.4473 | LR | 0.14 | 0.3766 | 0.0001 | 0.4529 |
| LinearSVC | 0.15 | 0.3704 | 0 | 0.4473 | LR | 0.15 | 0.3704 | 0.0005 | 0.4473 |
| LinearSVC | 0.16 | 0.3643 | 0 | 0.4473 | LR | 0.16 | 0.3635 | 0.0001 | 0.4473 |
| LinearSVC | 0.17 | 0.358 | 0 | 0.4473 | LR | 0.17 | 0.3577 | 0.0001 | 0.4529 |
| LinearSVC | 0.18 | 0.3512 | 0 | 0.4473 | LR | 0.18 | 0.3511 | 0.0001 | 0.4529 |
| LinearSVC | 0.19 | 0.3443 | 0 | 0.4529 | LR | 0.19 | 0.344 | 0 | 0.4473 |
| LinearSVC | 0.2 | 0.3379 | 0 | 0.4417 | LR | 0.2 | 0.3374 | 0.0001 | 0.4473 |
| LinearSVC | 0.21 | 0.3321 | 0 | 0.4473 | LR | 0.21 | 0.3311 | 0.0001 | 0.4473 |
| LinearSVC | 0.22 | 0.3265 | 0 | 0.4474 | LR | 0.22 | 0.325 | 0 | 0.4473 |
| LinearSVC | 0.23 | 0.3192 | 0 | 0.4473 | LR | 0.23 | 0.3183 | 0.0001 | 0.4473 |
| LinearSVC | 0.24 | 0.3123 | 0 | 0.4473 | LR | 0.24 | 0.3121 | 0.0001 | 0.4473 |
| LinearSVC | 0.25 | 0.3064 | 0 | 0.4415 | LR | 0.25 | 0.3058 | 0.0001 | 0.4417 |
| LinearSVC | 0.26 | 0.2987 | 0 | 0.4473 | LR | 0.26 | 0.2993 | 0.0001 | 0.4473 |
| LinearSVC | 0.27 | 0.2921 | 0 | 0.4473 | LR | 0.27 | 0.2931 | 0.0001 | 0.4473 |
| LinearSVC | 0.28 | 0.2861 | 0 | 0.4473 | LR | 0.28 | 0.286 | 0.0001 | 0.4473 |
| LinearSVC | 0.29 | 0.28 | 0 | 0.4473 | LR | 0.29 | 0.2784 | 0.0001 | 0.4473 |
| LinearSVC | 0.3 | 0.2745 | 0 | 0.4415 | LR | 0.3 | 0.2729 | 0 | 0.4417 |
| LinearSVC | 0.31 | 0.2663 | 0 | 0.4473 | LR | 0.31 | 0.266 | 0.0001 | 0.4473 |
| LinearSVC | 0.32 | 0.2585 | 0 | 0.4473 | LR | 0.32 | 0.2602 | 0.0005 | 0.4417 |
| LinearSVC | 0.33 | 0.2522 | 0 | 0.4474 | LR | 0.33 | 0.2531 | 0.0005 | 0.4473 |
| LinearSVC | 0.34 | 0.2453 | 0 | 0.4473 | LR | 0.34 | 0.2471 | 0.0001 | 0.4473 |
| LinearSVC | 0.35 | 0.2387 | 0 | 0.4473 | LR | 0.35 | 0.2416 | 0.0005 | 0.4474 |
| LinearSVC | 0.36 | 0.2337 | 0 | 0.4473 | LR | 0.36 | 0.2342 | 0.0001 | 0.4473 |
| LinearSVC | 0.37 | 0.2276 | 0 | 0.4474 | LR | 0.37 | 0.2291 | 0 | 0.4415 |
| LinearSVC | 0.38 | 0.2216 | 0 | 0.4473 | LR | 0.38 | 0.2223 | 0.0001 | 0.4529 |
| LinearSVC | 0.39 | 0.2142 | 0 | 0.4473 | LR | 0.39 | 0.2168 | 0.0001 | 0.4473 |
| LinearSVC | 0.4 | 0.2085 | 0 | 0.4473 | LR | 0.4 | 0.2104 | 0.0005 | 0.4529 |
| LinearSVC | 0.41 | 0.2003 | 0 | 0.4474 | LR | 0.41 | 0.2033 | 0.0005 | 0.4473 |
| LinearSVC | 0.42 | 0.1947 | 0 | 0.4415 | LR | 0.42 | 0.197 | 0.0001 | 0.4473 |
| LinearSVC | 0.43 | 0.1886 | 0 | 0.4473 | LR | 0.43 | 0.1904 | 0.0001 | 0.4415 |
| LinearSVC | 0.44 | 0.183 | 0 | 0.4473 | LR | 0.44 | 0.1846 | 0.0015 | 0.4473 |
| LinearSVC | 0.45 | 0.1764 | 0 | 0.4473 | LR | 0.45 | 0.1795 | 0 | 0.4415 |
| LinearSVC | 0.46 | 0.1702 | 0 | 0.4473 | LR | 0.46 | 0.1725 | 0.0001 | 0.4417 |
| LinearSVC | 0.47 | 0.1658 | 0 | 0.4473 | LR | 0.47 | 0.1655 | 0.0001 | 0.4473 |
| LinearSVC | 0.48 | 0.1594 | 0 | 0.4473 | LR | 0.48 | 0.1588 | 0 | 0.4473 |
| LinearSVC | 0.49 | 0.1533 | 0 | 0.4474 | LR | 0.49 | 0.1516 | 0.0001 | 0.4473 |
| LinearSVC | 0.5 | 0.1462 | 0 | 0.4473 | LR | 0.5 | 0.1459 | 0.0001 | 0.4473 |
| LinearSVC | 0.51 | 0.1404 | 0 | 0.4473 | LR | 0.51 | 0.1418 | 0.0001 | 0.4473 |
| LinearSVC | 0.52 | 0.1337 | 0 | 0.4529 | LR | 0.52 | 0.1358 | 0.0001 | 0.4473 |
| LinearSVC | 0.53 | 0.1307 | 0 | 0.4473 | LR | 0.53 | 0.1309 | 0.0001 | 0.4417 |
| LinearSVC | 0.54 | 0.1255 | 0 | 0.4473 | LR | 0.54 | 0.1265 | 0.0005 | 0.4415 |
| LinearSVC | 0.55 | 0.1197 | 0 | 0.4473 | LR | 0.55 | 0.1214 | 0.0005 | 0.4529 |
| LinearSVC | 0.56 | 0.1151 | 0 | 0.4415 | LR | 0.56 | 0.1153 | 0 | 0.4529 |
| LinearSVC | 0.57 | 0.1113 | 0 | 0.4473 | LR | 0.57 | 0.111 | 0.0001 | 0.4473 |
| LinearSVC | 0.58 | 0.1049 | 0 | 0.4529 | LR | 0.58 | 0.1079 | 0.0001 | 0.4473 |
| LinearSVC | 0.59 | 0.0994 | 0 | 0.4473 | LR | 0.59 | 0.1061 | 0.0001 | 0.4473 |
| LinearSVC | 0.6 | 0.0947 | 0 | 0.4415 | LR | 0.6 | 0.1025 | 0.0005 | 0.4473 |
| LinearSVC | 0.61 | 0.0931 | 0 | 0.4529 | LR | 0.61 | 0.0974 | 0.0005 | 0.4415 |
| LinearSVC | 0.62 | 0.0885 | 0 | 0.4529 | LR | 0.62 | 0.0939 | 0.0001 | 0.4473 |
| LinearSVC | 0.63 | 0.0863 | 0 | 0.4473 | LR | 0.63 | 0.0894 | 0.0001 | 0.4473 |
| LinearSVC | 0.64 | 0.0815 | 0 | 0.4415 | LR | 0.64 | 0.0835 | 0.0001 | 0.4473 |
| LinearSVC | 0.65 | 0.0769 | 0 | 0.4473 | LR | 0.65 | 0.0803 | 0.0005 | 0.4473 |
| LinearSVC | 0.66 | 0.0691 | 0 | 0.4473 | LR | 0.66 | 0.0767 | 0.0005 | 0.4473 |
| LinearSVC | 0.67 | 0.0684 | 0 | 0.4473 | LR | 0.67 | 0.0729 | 0.0005 | 0.4529 |
| LinearSVC | 0.68 | 0.0643 | 0 | 0.4473 | LR | 0.68 | 0.0674 | 0.0001 | 0.4473 |
| LinearSVC | 0.69 | 0.0584 | 0 | 0.4473 | LR | 0.69 | 0.0638 | 0 | 0.4473 |
| LinearSVC | 0.7 | 0.0549 | 0 | 0.4473 | LR | 0.7 | 0.0633 | 0.0001 | 0.4473 |
| LinearSVC | 0.71 | 0.0496 | 0 | 0.4415 | LR | 0.71 | 0.0574 | 0.0005 | 0.4529 |
| LinearSVC | 0.72 | 0.047 | 0 | 0.4473 | LR | 0.72 | 0.0521 | 0.0005 | 0.4473 |
| LinearSVC | 0.73 | 0.0428 | 0 | 0.4473 | LR | 0.73 | 0.0502 | 0.0005 | 0.4473 |
| LinearSVC | 0.74 | 0.0409 | 0 | 0.4473 | LR | 0.74 | 0.0454 | 0 | 0.4473 |
| LinearSVC | 0.75 | 0.0385 | 0 | 0.4473 | LR | 0.75 | 0.0432 | 0.0001 | 0.4473 |
| LinearSVC | 0.76 | 0.037 | 0 | 0.4473 | LR | 0.76 | 0.039 | 0.0001 | 0.4417 |
| LinearSVC | 0.77 | 0.0355 | 0 | 0.4529 | LR | 0.77 | 0.0365 | 0.0001 | 0.4473 |
| LinearSVC | 0.78 | 0.0346 | 0 | 0.4473 | LR | 0.78 | 0.0328 | 0.0001 | 0.4473 |
| LinearSVC | 0.79 | 0.03 | 0 | 0.4473 | LR | 0.79 | 0.0285 | 0.0001 | 0.4473 |
| LinearSVC | 0.8 | 0.0269 | 0 | 0.4473 | LR | 0.8 | 0.027 | 0.0005 | 0.4415 |
| LinearSVC | 0.81 | 0.0237 | 0 | 0.4473 | LR | 0.81 | 0.0267 | 0.0001 | 0.4473 |
| LinearSVC | 0.82 | 0.0204 | 0 | 0.4415 | LR | 0.82 | 0.0234 | 0.0001 | 0.4529 |
| LinearSVC | 0.83 | 0.0199 | 0 | 0.4473 | LR | 0.83 | 0.0242 | 0.0001 | 0.4473 |
| LinearSVC | 0.84 | 0.019 | 0 | 0.4473 | LR | 0.84 | 0.0224 | 0.0005 | 0.4473 |
| LinearSVC | 0.85 | 0.0164 | 0 | 0.4473 | LR | 0.85 | 0.0202 | 0.0001 | 0.4473 |
| LinearSVC | 0.86 | 0.015 | 0 | 0.4473 | LR | 0.86 | 0.0176 | 0 | 0.4529 |
| LinearSVC | 0.87 | 0.0138 | 0 | 0.4415 | LR | 0.87 | 0.0168 | 0.0001 | 0.4473 |
| LinearSVC | 0.88 | 0.011 | 0 | 0.4473 | LR | 0.88 | 0.0149 | 0.0001 | 0.4473 |
| LinearSVC | 0.89 | 0.0095 | 0 | 0.4473 | LR | 0.89 | 0.0138 | 0.0005 | 0.4473 |
| LinearSVC | 0.9 | 0.0075 | 0 | 0.4473 | LR | 0.9 | 0.0111 | 0.0001 | 0.4473 |
| LinearSVC | 0.91 | 0.0041 | 0 | 0.4473 | LR | 0.91 | 0.0086 | 0.0001 | 0.4473 |
| LinearSVC | 0.92 | 0.0026 | 0 | 0.4529 | LR | 0.92 | 0.0061 | 0.0001 | 0.4473 |
| LinearSVC | 0.93 | 0.0015 | 0 | 0.4415 | LR | 0.93 | 0.0038 | 0.0001 | 0.4474 |
| LinearSVC | 0.94 | 0.0006 | 0 | 0.4415 | LR | 0.94 | 0.003 | 0.0001 | 0.4473 |
| LinearSVC | 0.95 | 0 | 0 | 0.4473 | LR | 0.95 | 0.0015 | 0.0001 | 0.4473 |
| LinearSVC | 0.96 | 0 | 0 | 0.4415 | LR | 0.96 | 0.0005 | 0.0005 | 0.4473 |
| LinearSVC | 0.97 | 0 | 0 | 0.4473 | LR | 0.97 | 0.0001 | 0 | 0.4529 |
| LinearSVC | 0.98 | 0 | 0 | 0.4473 | LR | 0.98 | 0 | 0.0001 | 0.4415 |
| LinearSVC | 0.99 | 0 | 0 | 0.4473 | LR | 0.99 | 0 | 0.0001 | 0.4473 |
|  |  |  |  |  |  |  |  |  |  |
| NB | 0.01 | 0.4584 | 0 | 0.4473 | RF | 0.01 | 0.4584 | 0 | 0.4473 |
| NB | 0.02 | 0.4529 | 0 | 0.4473 | RF | 0.02 | 0.4529 | 0 | 0.4415 |
| NB | 0.03 | 0.4473 | 0 | 0.4473 | RF | 0.03 | 0.4473 | 0 | 0.4473 |
| NB | 0.04 | 0.4415 | 0 | 0.4415 | RF | 0.04 | 0.4415 | 0 | 0.4473 |
| NB | 0.05 | 0.4356 | 0 | 0.4415 | RF | 0.05 | 0.4356 | 0 | 0.4473 |
| NB | 0.06 | 0.4296 | 0 | 0.4473 | RF | 0.06 | 0.4296 | 0 | 0.4473 |
| NB | 0.07 | 0.4235 | 0 | 0.4529 | RF | 0.07 | 0.4235 | 0 | 0.4473 |
| NB | 0.08 | 0.4172 | 0 | 0.4417 | RF | 0.08 | 0.4172 | 0 | 0.4473 |
| NB | 0.09 | 0.4108 | 0 | 0.4474 | RF | 0.09 | 0.4108 | 0 | 0.4415 |
| NB | 0.1 | 0.4043 | 0 | 0.4529 | RF | 0.1 | 0.4043 | 0 | 0.4473 |
| NB | 0.11 | 0.3976 | 0 | 0.4473 | RF | 0.11 | 0.3976 | 0 | 0.4415 |
| NB | 0.12 | 0.3907 | 0 | 0.4415 | RF | 0.12 | 0.3907 | 0 | 0.4417 |
| NB | 0.13 | 0.3837 | 0 | 0.4415 | RF | 0.13 | 0.3847 | 0 | 0.4474 |
| NB | 0.14 | 0.3766 | 0 | 0.4473 | RF | 0.14 | 0.3787 | 0 | 0.4415 |
| NB | 0.15 | 0.3692 | 0 | 0.4473 | RF | 0.15 | 0.3729 | 0 | 0.4473 |
| NB | 0.16 | 0.3617 | 0 | 0.4473 | RF | 0.16 | 0.3675 | 0 | 0.4474 |
| NB | 0.17 | 0.354 | 0 | 0.4473 | RF | 0.17 | 0.3615 | 0 | 0.4473 |
| NB | 0.18 | 0.3462 | 0 | 0.4417 | RF | 0.18 | 0.3557 | 0 | 0.4473 |
| NB | 0.19 | 0.3381 | 0 | 0.4529 | RF | 0.19 | 0.3499 | 0 | 0.4474 |
| NB | 0.2 | 0.3298 | 0 | 0.4473 | RF | 0.2 | 0.3459 | 0 | 0.4529 |
| NB | 0.21 | 0.3213 | 0 | 0.4473 | RF | 0.21 | 0.3399 | 0 | 0.4473 |
| NB | 0.22 | 0.3126 | 0 | 0.4473 | RF | 0.22 | 0.3349 | 0 | 0.4473 |
| NB | 0.23 | 0.3037 | 0 | 0.4473 | RF | 0.23 | 0.33 | 0 | 0.4473 |
| NB | 0.24 | 0.2945 | 0 | 0.4473 | RF | 0.24 | 0.3252 | 0 | 0.4473 |
| NB | 0.25 | 0.2851 | 0 | 0.4417 | RF | 0.25 | 0.3209 | 0 | 0.4529 |
| NB | 0.26 | 0.2755 | 0 | 0.4473 | RF | 0.26 | 0.317 | 0 | 0.4415 |
| NB | 0.27 | 0.2656 | 0 | 0.4473 | RF | 0.27 | 0.3129 | 0 | 0.4473 |
| NB | 0.28 | 0.2554 | 0 | 0.4417 | RF | 0.28 | 0.3089 | 0 | 0.4473 |
| NB | 0.29 | 0.2449 | 0 | 0.4473 | RF | 0.29 | 0.3024 | 0 | 0.4415 |
| NB | 0.3 | 0.2341 | 0 | 0.4473 | RF | 0.3 | 0.2989 | 0 | 0.4529 |
| NB | 0.31 | 0.223 | 0 | 0.4415 | RF | 0.31 | 0.2954 | 0 | 0.4529 |
| NB | 0.32 | 0.2116 | 0 | 0.4473 | RF | 0.32 | 0.2939 | 0 | 0.4529 |
| NB | 0.33 | 0.2127 | 0 | 0.4473 | RF | 0.33 | 0.2908 | 0 | 0.4473 |
| NB | 0.34 | 0.2078 | 0 | 0.4473 | RF | 0.34 | 0.2893 | 0 | 0.4473 |
| NB | 0.35 | 0.2036 | 0 | 0.4473 | RF | 0.35 | 0.2854 | 0 | 0.4473 |
| NB | 0.36 | 0.1994 | 0 | 0.4473 | RF | 0.36 | 0.2843 | 0 | 0.4415 |
| NB | 0.37 | 0.1953 | 0 | 0.4529 | RF | 0.37 | 0.283 | 0 | 0.4473 |
| NB | 0.38 | 0.1917 | 0 | 0.4529 | RF | 0.38 | 0.2814 | 0 | 0.4473 |
| NB | 0.39 | 0.1874 | 0 | 0.4473 | RF | 0.39 | 0.2799 | 0 | 0.4473 |
| NB | 0.4 | 0.1834 | 0 | 0.4473 | RF | 0.4 | 0.2795 | 0 | 0.4473 |
| NB | 0.41 | 0.1786 | 0 | 0.4473 | RF | 0.41 | 0.2777 | 0 | 0.4473 |
| NB | 0.42 | 0.1747 | 0 | 0.4415 | RF | 0.42 | 0.2766 | 0 | 0.4473 |
| NB | 0.43 | 0.1699 | 0 | 0.4417 | RF | 0.43 | 0.2767 | 0 | 0.4529 |
| NB | 0.44 | 0.1649 | 0 | 0.4473 | RF | 0.44 | 0.2765 | 0 | 0.4417 |
| NB | 0.45 | 0.1602 | 0 | 0.4473 | RF | 0.45 | 0.2754 | 0 | 0.4474 |
| NB | 0.46 | 0.1556 | 0 | 0.4473 | RF | 0.46 | 0.275 | 0 | 0.4415 |
| NB | 0.47 | 0.1507 | 0 | 0.4473 | RF | 0.47 | 0.2756 | 0 | 0.4415 |
| NB | 0.48 | 0.1455 | 0 | 0.4474 | RF | 0.48 | 0.2753 | 0 | 0.4473 |
| NB | 0.49 | 0.1403 | 0 | 0.4473 | RF | 0.49 | 0.2746 | 0 | 0.4415 |
| NB | 0.5 | 0.1344 | 0 | 0.4529 | RF | 0.5 | 0.275 | 0 | 0.4473 |
| NB | 0.51 | 0.1291 | 0 | 0.4415 | RF | 0.51 | 0.2721 | 0 | 0.4473 |
| NB | 0.52 | 0.1232 | 0 | 0.4415 | RF | 0.52 | 0.2712 | 0 | 0.4473 |
| NB | 0.53 | 0.1171 | 0 | 0.4473 | RF | 0.53 | 0.2706 | 0 | 0.4473 |
| NB | 0.54 | 0.1108 | 0 | 0.4473 | RF | 0.54 | 0.2686 | 0 | 0.4473 |
| NB | 0.55 | 0.1039 | 0 | 0.4473 | RF | 0.55 | 0.2658 | 0 | 0.4529 |
| NB | 0.56 | 0.0966 | 0 | 0.4473 | RF | 0.56 | 0.2653 | 0 | 0.4473 |
| NB | 0.57 | 0.089 | 0 | 0.4473 | RF | 0.57 | 0.2606 | 0 | 0.4473 |
| NB | 0.58 | 0.0811 | 0 | 0.4473 | RF | 0.58 | 0.2576 | 0 | 0.4473 |
| NB | 0.59 | 0.0744 | 0 | 0.4415 | RF | 0.59 | 0.2532 | 0 | 0.4473 |
| NB | 0.6 | 0.0664 | 0 | 0.4473 | RF | 0.6 | 0.2497 | 0 | 0.4529 |
| NB | 0.61 | 0.0573 | 0 | 0.4473 | RF | 0.61 | 0.2454 | 0 | 0.4473 |
| NB | 0.62 | 0.0481 | 0 | 0.4529 | RF | 0.62 | 0.2403 | 0 | 0.4473 |
| NB | 0.63 | 0.0396 | 0 | 0.4474 | RF | 0.63 | 0.2336 | 0 | 0.4473 |
| NB | 0.64 | 0.0295 | 0 | 0.4473 | RF | 0.64 | 0.229 | 0 | 0.4473 |
| NB | 0.65 | 0.019 | 0 | 0.4474 | RF | 0.65 | 0.2206 | 0 | 0.4474 |
| NB | 0.66 | 0.0121 | 0 | 0.4473 | RF | 0.66 | 0.2146 | 0 | 0.4473 |
| NB | 0.67 | 0 | 0 | 0.4529 | RF | 0.67 | 0.2071 | 0 | 0.4473 |
| NB | 0.68 | 0 | 0 | 0.4473 | RF | 0.68 | 0.1994 | 0 | 0.4529 |
| NB | 0.69 | 0 | 0 | 0.4473 | RF | 0.69 | 0.1922 | 0 | 0.4529 |
| NB | 0.7 | 0 | 0 | 0.4473 | RF | 0.7 | 0.1838 | 0 | 0.4473 |
| NB | 0.71 | 0 | 0 | 0.4417 | RF | 0.71 | 0.1741 | 0 | 0.4473 |
| NB | 0.72 | 0 | 0 | 0.4529 | RF | 0.72 | 0.166 | 0 | 0.4473 |
| NB | 0.73 | 0 | 0 | 0.4473 | RF | 0.73 | 0.1557 | 0 | 0.4473 |
| NB | 0.74 | 0 | 0 | 0.4473 | RF | 0.74 | 0.1435 | 0 | 0.4473 |
| NB | 0.75 | 0 | 0 | 0.4473 | RF | 0.75 | 0.1356 | 0 | 0.4473 |
| NB | 0.76 | 0 | 0 | 0.4473 | RF | 0.76 | 0.1247 | 0 | 0.4473 |
| NB | 0.77 | 0 | 0 | 0.4473 | RF | 0.77 | 0.1138 | 0 | 0.4473 |
| NB | 0.78 | 0 | 0 | 0.4473 | RF | 0.78 | 0.1014 | 0 | 0.4473 |
| NB | 0.79 | 0 | 0 | 0.4473 | RF | 0.79 | 0.0887 | 0 | 0.4473 |
| NB | 0.8 | 0 | 0 | 0.4529 | RF | 0.8 | 0.0752 | 0 | 0.4473 |
| NB | 0.81 | 0 | 0 | 0.4529 | RF | 0.81 | 0.062 | 0 | 0.4473 |
| NB | 0.82 | 0 | 0 | 0.4417 | RF | 0.82 | 0.0509 | 0 | 0.4529 |
| NB | 0.83 | 0 | 0 | 0.4473 | RF | 0.83 | 0.0385 | 0 | 0.4417 |
| NB | 0.84 | 0 | 0 | 0.4473 | RF | 0.84 | 0.0283 | 0 | 0.4473 |
| NB | 0.85 | 0 | 0 | 0.4529 | RF | 0.85 | 0.0208 | 0 | 0.4473 |
| NB | 0.86 | 0 | 0 | 0.4473 | RF | 0.86 | 0.0132 | 0 | 0.4473 |
| NB | 0.87 | 0 | 0 | 0.4473 | RF | 0.87 | 0.0077 | 0 | 0.4473 |
| NB | 0.88 | 0 | 0 | 0.4473 | RF | 0.88 | 0.004 | 0 | 0.4473 |
| NB | 0.89 | 0 | 0 | 0.4529 | RF | 0.89 | 0.0016 | 0 | 0.4473 |
| NB | 0.9 | 0 | 0 | 0.4473 | RF | 0.9 | 0.0002 | 0 | 0.4473 |
| NB | 0.91 | 0 | 0 | 0.4358 | RF | 0.91 | 0 | 0 | 0.4474 |
| NB | 0.92 | 0 | 0 | 0.4529 | RF | 0.92 | 0 | 0 | 0.4529 |
| NB | 0.93 | 0 | 0 | 0.4473 | RF | 0.93 | 0 | 0 | 0.4474 |
| NB | 0.94 | 0 | 0 | 0.4473 | RF | 0.94 | 0 | 0 | 0.4415 |
| NB | 0.95 | 0 | 0 | 0.4473 | RF | 0.95 | 0 | 0 | 0.4473 |
| NB | 0.96 | 0 | 0 | 0.4473 | RF | 0.96 | 0 | 0 | 0.4415 |
| NB | 0.97 | 0 | 0 | 0.4473 | RF | 0.97 | 0 | 0 | 0.4473 |
| NB | 0.98 | 0 | 0 | 0.4474 | RF | 0.98 | 0 | 0 | 0.4473 |
| NB | 0.99 | 0 | 0 | 0.4473 | RF | 0.99 | 0 | 0 | 0.4529 |
|  |  |  |  |  |  |  |  |  |  |
| XGBoost | 0.01 | 0.4584 | 0 | 0.4415 |  |  |  |  |  |
| XGBoost | 0.02 | 0.4529 | 0 | 0.4473 |  |  |  |  |  |
| XGBoost | 0.03 | 0.4473 | 0 | 0.4529 |  |  |  |  |  |
| XGBoost | 0.04 | 0.4415 | 0 | 0.4417 |  |  |  |  |  |
| XGBoost | 0.05 | 0.4356 | 0 | 0.4473 |  |  |  |  |  |
| XGBoost | 0.06 | 0.4296 | 0 | 0.4473 |  |  |  |  |  |
| XGBoost | 0.07 | 0.4235 | 0 | 0.4473 |  |  |  |  |  |
| XGBoost | 0.08 | 0.4172 | 0 | 0.4473 |  |  |  |  |  |
| XGBoost | 0.09 | 0.4108 | 0 | 0.4473 |  |  |  |  |  |
| XGBoost | 0.1 | 0.4043 | 0 | 0.4473 |  |  |  |  |  |
| XGBoost | 0.11 | 0.3976 | 0 | 0.4529 |  |  |  |  |  |
| XGBoost | 0.12 | 0.3907 | 0 | 0.4473 |  |  |  |  |  |
| XGBoost | 0.13 | 0.3837 | 0 | 0.4473 |  |  |  |  |  |
| XGBoost | 0.14 | 0.3766 | 0 | 0.4473 |  |  |  |  |  |
| XGBoost | 0.15 | 0.3692 | 0 | 0.4417 |  |  |  |  |  |
| XGBoost | 0.16 | 0.3626 | 0 | 0.4473 |  |  |  |  |  |
| XGBoost | 0.17 | 0.3564 | 0 | 0.4473 |  |  |  |  |  |
| XGBoost | 0.18 | 0.3512 | 0 | 0.4473 |  |  |  |  |  |
| XGBoost | 0.19 | 0.3459 | 0 | 0.4473 |  |  |  |  |  |
| XGBoost | 0.2 | 0.3392 | 0 | 0.4417 |  |  |  |  |  |
| XGBoost | 0.21 | 0.3334 | 0 | 0.4473 |  |  |  |  |  |
| XGBoost | 0.22 | 0.3274 | 0 | 0.4473 |  |  |  |  |  |
| XGBoost | 0.23 | 0.3209 | 0 | 0.4417 |  |  |  |  |  |
| XGBoost | 0.24 | 0.3154 | 0 | 0.4417 |  |  |  |  |  |
| XGBoost | 0.25 | 0.3098 | 0 | 0.4473 |  |  |  |  |  |
| XGBoost | 0.26 | 0.3042 | 0 | 0.4473 |  |  |  |  |  |
| XGBoost | 0.27 | 0.2971 | 0 | 0.4415 |  |  |  |  |  |
| XGBoost | 0.28 | 0.2904 | 0 | 0.4473 |  |  |  |  |  |
| XGBoost | 0.29 | 0.2852 | 0 | 0.4356 |  |  |  |  |  |
| XGBoost | 0.3 | 0.2784 | 0 | 0.4529 |  |  |  |  |  |
| XGBoost | 0.31 | 0.2733 | 0 | 0.4473 |  |  |  |  |  |
| XGBoost | 0.32 | 0.2669 | 0 | 0.4474 |  |  |  |  |  |
| XGBoost | 0.33 | 0.2604 | 0 | 0.4473 |  |  |  |  |  |
| XGBoost | 0.34 | 0.254 | 0 | 0.4473 |  |  |  |  |  |
| XGBoost | 0.35 | 0.2492 | 0 | 0.4473 |  |  |  |  |  |
| XGBoost | 0.36 | 0.2431 | 0 | 0.4473 |  |  |  |  |  |
| XGBoost | 0.37 | 0.2392 | 0 | 0.4474 |  |  |  |  |  |
| XGBoost | 0.38 | 0.2339 | 0 | 0.4473 |  |  |  |  |  |
| XGBoost | 0.39 | 0.2297 | 0 | 0.4473 |  |  |  |  |  |
| XGBoost | 0.4 | 0.223 | 0 | 0.4473 |  |  |  |  |  |
| XGBoost | 0.41 | 0.2189 | 0 | 0.4473 |  |  |  |  |  |
| XGBoost | 0.42 | 0.2131 | 0 | 0.4473 |  |  |  |  |  |
| XGBoost | 0.43 | 0.2076 | 0 | 0.4473 |  |  |  |  |  |
| XGBoost | 0.44 | 0.2026 | 0 | 0.4529 |  |  |  |  |  |
| XGBoost | 0.45 | 0.1965 | 0 | 0.4529 |  |  |  |  |  |
| XGBoost | 0.46 | 0.1902 | 0 | 0.4473 |  |  |  |  |  |
| XGBoost | 0.47 | 0.1845 | 0 | 0.4473 |  |  |  |  |  |
| XGBoost | 0.48 | 0.1782 | 0 | 0.4473 |  |  |  |  |  |
| XGBoost | 0.49 | 0.1742 | 0 | 0.4415 |  |  |  |  |  |
| XGBoost | 0.5 | 0.1697 | 0 | 0.4473 |  |  |  |  |  |
| XGBoost | 0.51 | 0.1643 | 0 | 0.4417 |  |  |  |  |  |
| XGBoost | 0.52 | 0.1612 | 0 | 0.4473 |  |  |  |  |  |
| XGBoost | 0.53 | 0.1557 | 0 | 0.4415 |  |  |  |  |  |
| XGBoost | 0.54 | 0.1529 | 0 | 0.4473 |  |  |  |  |  |
| XGBoost | 0.55 | 0.1482 | 0 | 0.4473 |  |  |  |  |  |
| XGBoost | 0.56 | 0.1432 | 0 | 0.4473 |  |  |  |  |  |
| XGBoost | 0.57 | 0.1389 | 0 | 0.4473 |  |  |  |  |  |
| XGBoost | 0.58 | 0.1368 | 0 | 0.4473 |  |  |  |  |  |
| XGBoost | 0.59 | 0.1329 | 0 | 0.4473 |  |  |  |  |  |
| XGBoost | 0.6 | 0.1291 | 0 | 0.4529 |  |  |  |  |  |
| XGBoost | 0.61 | 0.1242 | 0 | 0.4415 |  |  |  |  |  |
| XGBoost | 0.62 | 0.1204 | 0 | 0.4473 |  |  |  |  |  |
| XGBoost | 0.63 | 0.115 | 0 | 0.4473 |  |  |  |  |  |
| XGBoost | 0.64 | 0.1102 | 0 | 0.4473 |  |  |  |  |  |
| XGBoost | 0.65 | 0.1071 | 0 | 0.4415 |  |  |  |  |  |
| XGBoost | 0.66 | 0.1036 | 0 | 0.4529 |  |  |  |  |  |
| XGBoost | 0.67 | 0.0988 | 0 | 0.4473 |  |  |  |  |  |
| XGBoost | 0.68 | 0.0969 | 0 | 0.4415 |  |  |  |  |  |
| XGBoost | 0.69 | 0.0921 | 0 | 0.4473 |  |  |  |  |  |
| XGBoost | 0.7 | 0.0881 | 0 | 0.4415 |  |  |  |  |  |
| XGBoost | 0.71 | 0.084 | 0 | 0.4473 |  |  |  |  |  |
| XGBoost | 0.72 | 0.0832 | 0 | 0.4415 |  |  |  |  |  |
| XGBoost | 0.73 | 0.0795 | 0 | 0.4473 |  |  |  |  |  |
| XGBoost | 0.74 | 0.0757 | 0 | 0.4473 |  |  |  |  |  |
| XGBoost | 0.75 | 0.0743 | 0 | 0.4473 |  |  |  |  |  |
| XGBoost | 0.76 | 0.0702 | 0 | 0.4473 |  |  |  |  |  |
| XGBoost | 0.77 | 0.0622 | 0 | 0.4473 |  |  |  |  |  |
| XGBoost | 0.78 | 0.0586 | 0 | 0.4473 |  |  |  |  |  |
| XGBoost | 0.79 | 0.0514 | 0 | 0.4473 |  |  |  |  |  |
| XGBoost | 0.8 | 0.0437 | 0 | 0.4529 |  |  |  |  |  |
| XGBoost | 0.81 | 0.038 | 0 | 0.4415 |  |  |  |  |  |
| XGBoost | 0.82 | 0.032 | 0 | 0.4473 |  |  |  |  |  |
| XGBoost | 0.83 | 0.0254 | 0 | 0.4415 |  |  |  |  |  |
| XGBoost | 0.84 | 0.0174 | 0 | 0.4415 |  |  |  |  |  |
| XGBoost | 0.85 | 0.0116 | 0 | 0.4473 |  |  |  |  |  |
| XGBoost | 0.86 | 0.0071 | 0 | 0.4473 |  |  |  |  |  |
| XGBoost | 0.87 | 0.0028 | 0 | 0.4529 |  |  |  |  |  |
| XGBoost | 0.88 | 0.0005 | 0 | 0.4473 |  |  |  |  |  |
| XGBoost | 0.89 | 0 | 0 | 0.4473 |  |  |  |  |  |
| XGBoost | 0.9 | 0 | 0 | 0.4473 |  |  |  |  |  |
| XGBoost | 0.91 | 0 | 0 | 0.4473 |  |  |  |  |  |
| XGBoost | 0.92 | 0 | 0 | 0.4474 |  |  |  |  |  |
| XGBoost | 0.93 | 0 | 0 | 0.4415 |  |  |  |  |  |
| XGBoost | 0.94 | 0 | 0 | 0.4529 |  |  |  |  |  |
| XGBoost | 0.95 | 0 | 0 | 0.4473 |  |  |  |  |  |
| XGBoost | 0.96 | 0 | 0 | 0.4415 |  |  |  |  |  |
| XGBoost | 0.97 | 0 | 0 | 0.4473 |  |  |  |  |  |
| XGBoost | 0.98 | 0 | 0 | 0.4529 |  |  |  |  |  |
| XGBoost | 0.99 | 0 | 0 | 0.4473 |  |  |  |  |  |

**Table S7. RF Model 'Feature ID-Feature Name' Reference Table**

| Feature ID | Feature Name |
| --- | --- |
| Feature1 | Which child of mother:First |
| Feature2 | Have been treated with antibiotics |
| Feature3 | The earliest age of using antibiotics: None |
| Feature4 | History of allergies |
| Feature5 | History of digestive system diseases |
| Feature6 | Regular lunch breaks |
| Feature7 | Food preference: No obvious |
| Feature8 | Food preference: Have much hated food |
| Feature9 | Self control ability: Good at self control |
| Feature10 | TZ1 Easy to lose spirit: None |
| Feature11 | TZ1 Easy to lose spirit: Rarely |
| Feature12 | TZ1 Easy to lose spirit: Sometimes |
| Feature13 | TZ2 Yellow complexion,no other children ruddy: None |
| Feature14 | TZ2 Yellow complexion,no other children ruddy: Rarely |
| Feature15 | TZ2 Yellow complexion,no other children ruddy: Sometimes |
| Feature16 | TZ3 Soft muscles,not as strong as other children: None |
| Feature17 | TZ3 Soft muscles,not as strong as other children: Rarely |
| Feature18 | TZ3 Soft muscles,not as strong as other children: Sometimes |
| Feature19 | TZ4 Always quiet: None |
| Feature20 | TZ5 Speak in a low voice: None |
| Feature21 | TZ5 Speak in a low voice: Rarely |
| Feature22 | TZ6 Not talkative : None |
| Feature23 | TZ7 Little activity makes sweating easily : None |
| Feature24 | TZ7 Little activity makes sweating easily : Often |
| Feature25 | TZ7 Little activity makes sweating easily : Always |
| Feature26 | TZ8 Hands and feet always lukewarm : None |
| Feature27 | TZ8 Hands and feet always lukewarm : Rarely |
| Feature28 | TZ8 Hands and feet always lukewarm : Sometimes |
| Feature29 | TZ9 Resistant to summer but not to winter : None |
| Feature30 | TZ9 Resistant to summer but not to winter : Rarely |
| Feature31 | TZ9 Resistant to summer but not to winter : Sometimes |
| Feature32 | TZ10 Afraid of cold than other children of the same age : None |
| Feature33 | TZ10 Afraid of cold than other children of the same age : Rarely |
| Feature34 | TZ11 Feel uncomfortable or afraid of eating(drinking)cold food : None |
| Feature35 | TZ11 Feel uncomfortable or afraid of eating cold food : Rarely |
| Feature36 | TZ12 Having cold or eating cold food makes it easy to have diarrhea : None |
| Feature37 | TZ12 Having cold or eating cold food makes it easy to have diarrhea : Rarely |
| Feature38 | TZ13 The body and face are always very hot : None |
| Feature39 | TZ13 The body and face are always very hot : Rarely |
| Feature40 | TZ13 The body and face are always very hot : Sometimes |
| Feature41 | TZ14 Skin and lips dry easily : None |
| Feature42 | TZ14 Skin and lips dry easily : Rarely |
| Feature43 | TZ14 Skin and lips dry easily : Sometimes |
| Feature44 | TZ15 Always excited : None |
| Feature45 | TZ15 Always excited : Sometimes |
| Feature46 | TZ15 Always excited : Often |
| Feature47 | TZ16 Easily restless : None |
| Feature48 | TZ16 Easily restless : Sometimes |
| Feature49 | TZ18 Abdominal obesity is flabby and soft : None |
| Feature50 | TZ19 Always sleepy : None |
| Feature51 | TZ19 Always sleepy : Rarely |
| Feature52 | TZ20 Like to eat sweet or greasy things : None |
| Feature53 | TZ20 Like to eat sweet or greasy things : Sometimes |
| Feature54 | TZ20 Like to eat sweet or greasy things : Often |
| Feature55 | TZ21 Snoring at night : None |
| Feature56 | TZ21 Snoring at night : Rarely |
| Feature57 | TZ21 Snoring at night : Sometimes |
| Feature58 | TZ22 Thick and greasy tongue coating : None |
| Feature59 | TZ22 Thick and greasy tongue coating : Rarely |
| Feature60 | TZ22 Thick and greasy tongue coating : Sometimes |
| Feature61 | TZ23 Always red eyes or easy to get gum : None |
| Feature62 | TZ23 Always red eyes or easy to get gum : Rarely |
| Feature63 | TZ23 Always red eyes or easy to get gum : Sometimes |
| Feature64 | TZ24 Eczema is easy to grow on the body : None |
| Feature65 | TZ24 Eczema is easy to grow on the body : Rarely |
| Feature66 | TZ25 Like to eat spicy or fried barbecue food : None |
| Feature67 | TZ26 Sweat is sticky : None |
| Feature68 | TZ26 Sweat is sticky : Rarely |
| Feature69 | TZ26 Sweat is sticky : Sometimes |
| Feature70 | TZ27 The skin always appears blue and purple ecchymosis unconsciously : None |
| Feature71 | TZ27 The skin always appears blue and purple ecchymosis unconsciously : Rarely |
| Feature72 | TZ28 Where there is pain on the body : None |
| Feature73 | TZ28 Where there is pain on the body : Rarely |
| Feature74 | TZ29 The skin on the body or the skin on the neck armpit or thigh root is dark : None |
| Feature75 | TZ29 The skin on the body or the skin on the neck armpit or thigh root is dark : Rarely |
| Feature76 | TZ30 Be prone to dark circles : None |
| Feature77 | TZ30 Be prone to dark circles : Rarely |
| Feature78 | TZ30 Be prone to dark circles : Sometimes |
| Feature79 | TZ31 Blue or dark gums : None |
| Feature80 | TZ31 Blue or dark gums : Rarely |
| Feature81 | TZ32 Rough skin like fish scales : None |
| Feature82 | TZ33 Lips are dark or blue : None |
| Feature83 | TZ33 Lips are dark or blue : Rarely |
| Feature84 | TZ34 Always moping : None |
| Feature85 | TZ34 Always moping : Rarely |
| Feature86 | TZ35 Easily nervous : None |
| Feature87 | TZ35 Easily nervous : Rarely |
| Feature88 | TZ36 Easy to lack self confidence : None |
| Feature89 | TZ36 Easy to lack self confidence : Rarely |
| Feature90 | TZ36 Easy to lack self confidence : Sometimes |
| Feature91 | TZ37 Easily afraid or frightened : None |
| Feature92 | TZ37 Easily afraid or frightened : Rarely |
| Feature93 | TZ37 Easily afraid or frightened : Sometimes |
| Feature94 | TZ38 Always crying when in trouble : None |
| Feature95 | TZ38 Always crying when in trouble : Sometimes |
| Feature96 | TZ38 Always crying when in trouble : Often |
| Feature97 | TZ39 Easily shy or timid : None |
| Feature98 | TZ39 Easily shy or timid : Sometimes |
| Feature99 | TZ40 Always worried about a lot of things : None |
| Feature100 | TZ40 Always worried about a lot of things : Rarely |
| Feature101 | TZ40 Always worried about a lot of things : Sometimes |
| Feature102 | TZ41 More attached to you now than before : None |
| Feature103 | TZ41 More attached to you now than before : Sometimes |
| Feature104 | TZ42 Sneez when do not have a cold : None |
| Feature105 | TZ42 Sneez when do not have a cold : Rarely |
| Feature106 | TZ43 Without cold but have stuffy nose and runny nose : None |
| Feature107 | TZ43 Without cold but have stuffy nose and runny nose : Rarely |
| Feature108 | TZ44 Cough and wheeze when the season changes the temperature changes or smells strange smell : None |
| Feature109 | TZ44 Cough and wheeze when the season changes the temperature changes or smells strange smell : Rarely |
| Feature110 | TZ45 Susceptible to allergies : None |
| Feature111 | TZ45 Susceptible to allergies : Rarely |
| Feature112 | TZ46 The skin is prone to itching and urticaria : None |
| Feature113 | TZ46 The skin is prone to itching and urticaria : Rarely |
| Feature114 | TZ47 Purpura has occurred in the skin due to allergies : None |
| Feature115 | TZ48 The skin turns red when scratched and scratches appear : None |
| Feature116 | TZ48 The skin turns red when scratched and scratches appear : Rarely |
| Feature117 | TZ49 Always lively and strong : None |
| Feature118 | TZ50 Can adapt to weather changes and new environment : None |
| Feature119 | TZ51 Easy to have nightmares or wake up from dreams : None |
| Feature120 | TZ51 Easy to have nightmares or wake up from dreams : Rarely |
| Feature121 | TZ51 Easy to have nightmares or wake up from dreams : Sometimes |
| Feature122 | YSRT1 Eat less than your peers : None |
| Feature123 | YSRT1 Eat less than your peers : Often |
| Feature124 | YSRT1 Eat less than your peers : Always |
| Feature125 | YSRT2 Eating slowly compared with peers : None |
| Feature126 | YSRT2 Eating slowly compared with peers : Often |
| Feature127 | YSRT2 Eating slowly compared with peers : Always |
| Feature128 | YSRT3 Not interested in food : None |
| Feature129 | YSRT3 Not interested in food : Sometimes |
| Feature130 | YSRT3 Not interested in food : Often |
| Feature131 | YSRT4 Refuse certain foods for more than a month : None |
| Feature132 | YSRT4 Refuse certain foods for more than a month : Often |
| Feature133 | YSRT4 Refuse certain foods for more than a month : Always |
| Feature134 | YSRT5 Unwilling to try new food : None |
| Feature135 | YSRT5 Unwilling to try new food : Often |
| Feature136 | YSRT6 Have a strong preference for certain textures or certain types of food : None |
| Feature137 | YSRT6 Have a strong preference for certain textures or certain types of food : Often |
| Feature138 | YSRT6 Have a strong preference for certain textures or certain types of food : Always |
| Feature139 | YSRT7 Distraction from eating : None |
| Feature140 | YSRT7 Distraction from eating : Often |
| Feature141 | YSRT7 Distraction from eating : Always |
| Feature142 | YSRT8 The dining place is not fixed : None |
| Feature143 | YSFY1 Few emotional exchanges during meals : None |
| Feature144 | YSFY1 Few emotional exchanges during meals : Sometimes |
| Feature145 | YSFY2 Force or punish children to eat more : None |
| Feature146 | YSFY2 Force or punish children to eat more : Sometimes |
| Feature147 | YSFY2 Force or punish children to eat more : Often |
| Feature148 | YSFY3 Induce children to eat : None |
| Feature149 | YSFY3 Induce children to eat : Sometimes |
| Feature150 | YSFY3 Induce children to eat : Often |
| Feature151 | YSFY4 Allow children to choose food at will : None |
| Feature152 | YSFY5 Children are rarely encouraged to taste new foods : None |
| Feature153 | YSFY6 Allow children to eat snacks if they want : None |
| Feature154 | YSFY7 Allow children to dine and wander around : None |
| Feature155 | YSFY7 Allow children to dine and wander around : Sometimes |
| Feature156 | YS1 Your child average meal time : None |
| Feature157 | YS2 Do you think your child has a picky eater problem : None |
| Feature158 | YS2 Do you think your child has a picky eater problem : Sometimes |

**Table S8. Mapping Reference Table Between RF Model Features and Streamlit Application Questionnaire Items**

| **Questionnaire Item** | **Answer Options and Assigned Values Upon Selection** | **Corresponding RF Model Feature** | **RF Model Feature ID** |
| --- | --- | --- | --- |
| 1.Is your child the mother 's firstborn? | Yes:1,0;No:0,1; | Which_child_of_mother_First | 1 |
| 2.Has your child ever been treated with antibiotics? | Yes:1,0;No:0,1; | Have_been_treated_with_antibiotics | 2 |
| 3.At what age did your child first use antibiotics? | Never use:1,0,0,0;Within 1 year old:0,0,0,0; 1 to 2 years old:0,0,0,0; 3 years old and above:0,0,0,0 | The_earliest_age_of_using_antibiotics_None | 3 |
| 4.Does your child have any history of allergies? | Yes:1,0;No:0,1; | History_of_allergies | 4 |
| 5.Does your child have digestive system disorders? | Yes:1,0;No:0,1; | History_of _digestive_system_diseases | 5 |
| 6.Does your child have regular lunches? | Yes:1,0;No:0,1; | Regular_lunch_breaks | 6 |
| 7.Does your child have any food preferences? | No obvious:1,0,0;Have much hated food:0,1,0;Have much loved food:0,0,0 | Food_preference_No_obvious | 7 |
|  |  | Food_preference_Have_much_hated_food | 8 |
| 8.How is your child's self control ability? | Rambunctious:0,0,0;Not good at selfcontrol but can accept discipline:0,0,0;Good at self control:0,0,1 | Self_control_ability_Good_at_self_control | 9 |
| 9.Do your children lose spirit easily? | Never:1,0,0,0,0;Rarely:0,1,0,0,0;Sometimes:0,0,1,0,0;Often:0,0,0,0,0;Always:0,0,0,0,0 | TZ1_Easy_to_lose_spirit_1 | 10 |
|  |  | TZ1_Easy_to_lose_spirit_2 | 11 |
|  |  | TZ1_Easy_to_lose_spirit_3 | 12 |
| 10.Does your child have a yellowish complexion and not look as rosy as other children? | Never:1,0,0,0,0;Rarely:0,1,0,0,0;Sometimes:0,0,1,0,0;Often:0,0,0,0,0;Always:0,0,0,0,0 | TZ2_Yellow_complexion,no_other_children_ruddy_1 | 13 |
|  |  | TZ2_Yellow_complexion,no_other_children_ruddy_2 | 14 |
|  |  | TZ2_Yellow_complexion,no_other_children_ruddy_3 | 15 |
| 11.Is your child's muscles flabby and not as strong as other children's? | Never:1,0,0,0,0;Rarely:0,1,0,0,0;Sometimes:0,0,1,0,0;Often:0,0,0,0,0;Always:0,0,0,0,0 | TZ3_Soft_muscles,not_as_strong_as_other_children_1 | 16 |
|  |  | TZ3_Soft_muscles,not_as_strong_as_other_children_2 | 17 |
|  |  | TZ3_Soft_muscles,not_as_strong_as_other_children_3 | 18 |
| 12.Is your child always very quiet? | Never:1,0,0,0,0;Rarely:0,0,0,0,0;Sometimes:0,0,0,0,0;Often:0,0,0,0,0;Always:0,0,0,0,0 | TZ4_Always_quiet_1 | 19 |
| 13.Does your child speak weakly or softly? | Never:1,0,0,0,0;Rarely:0,1,0,0,0;Sometimes:0,0,0,0,0;Often:0,0,0,0,0;Always:0,0,0,0,0 | TZ5_Speak_in_a_low_voice_1 | 20 |
|  |  | TZ5_Speak_in_a_low_voice_2 | 21 |
| 14.Does your child not like to talk? | Never:1,0,0,0,0;Rarely:0,0,0,0,0;Sometimes:0,0,0,0,0;Often:0,0,0,0,0;Always:0,0,0,0,0 | TZ6_Not_talkative_1 | 22 |
| 15.Does your child sweat easily even with a little activity? | Never:1,0,0,0,0;Rarely:0,0,0,0,0;Sometimes:0,0,0,0,0;Often:0,0,0,1,0;Always:0,0,0,0,1 | TZ7_Little_activity_makes_sweating easily_1 | 23 |
|  |  | TZ7_Little_activity_makes_sweating easily_4 | 24 |
|  |  | TZ7_Little_activity_makes_sweating easily_5 | 25 |
| 16.Are your child's hands and feet always cold? | Never:1,0,0,0,0;Rarely:0,1,0,0,0;Sometimes:0,0,1,0,0;Often:0,0,0,0,0;Always:0,0,0,0,0 | TZ8_Hands_and_feet_always_lukewarm_1 | 26 |
|  |  | TZ8_Hands_and_feet_always_lukewarm_2 | 27 |
|  |  | TZ8_Hands_and_feet_always_lukewarm_3 | 28 |
| 17.Is your child tolerant of summer but not winter? | Never:1,0,0,0,0;Rarely:0,1,0,0,0;Sometimes:0,0,1,0,0;Often:0,0,0,0,0;Always:0,0,0,0,0 | TZ9_Resistant_to_summer_but_not_to_winter_1 | 29 |
|  |  | TZ9_Resistant_to_summer_but_not_to_winter_2 | 30 |
|  |  | TZ9_Resistant_to_summer_but_not_to_winter_3 | 31 |
| 18.Is your child more sensitive to cold than other children of the same age (for example, afraid of air conditioning, fans, winter, etc.)? | Never:1,0,0,0,0;Rarely:0,1,0,0,0;Sometimes:0,0,0,0,0;Often:0,0,0,0,0;Always:0,0,0,0,0 | TZ10_Afraid_of_cold_than_other_children_of_the_same_age_1 | 32 |
|  |  | TZ10_Afraid_of_cold_than_other_children_of_the_same_age_2 | 33 |
| 19.Does your child feel uncomfortable or reluctant to eat (or drink) cold things? | Never:1,0,0,0,0;Rarely:0,1,0,0,0;Sometimes:0,0,0,0,0;Often:0,0,0,0,0;Always:0,0,0,0,0 | TZ11_Feel_uncomfortable_or_afraid_of_eating(drinking)cold_food_1 | 34 |
|  |  | TZ11_Feel_uncomfortable_or_afraid_of_eating_cold_food_2 | 35 |
| 20.Does your child often get diarrhea after catching a cold or consuming cold food or drinks? | Never:1,0,0,0,0;Rarely:0,1,0,0,0;Sometimes:0,0,0,0,0;Often:0,0,0,0,0;Always:0,0,0,0,0 | TZ12_Having_cold_or_eating_cold_food_makes_it_easy_to_have_diarrhea_1 | 36 |
|  |  | TZ12_Having_cold_or_eating_cold_food_makes_it_easy_to_have_diarrhea_2 | 37 |
| 21.Does your child often feel hot, especially in the body and face?Does your child often feel hot, especially in the body and face? | Never:1,0,0,0,0;Rarely:0,1,0,0,0;Sometimes:0,0,1,0,0;Often:0,0,0,0,0;Always:0,0,0,0,0 | TZ13_The_body_and_face_are_always_very_hot_1 | 38 |
|  |  | TZ13_The_body_and_face_are_always_very_hot_2 | 39 |
|  |  | TZ13_The_body_and_face_are_always_very_hot_3 | 40 |
| 22.Does your child’s skin and lips get dry easily? | Never:1,0,0,0,0;Rarely:0,1,0,0,0;Sometimes:0,0,1,0,0;Often:0,0,0,0,0;Always:0,0,0,0,0 | TZ14_Skin_and_lips_dry_easily_1 | 41 |
|  |  | TZ14_Skin_and_lips_dry_easily_2 | 42 |
|  |  | TZ14_Skin_and_lips_dry_easily_3 | 43 |
| 23.Is your child always very excited? | Never:1,0,0,0,0;Rarely:0,0,0,0,0;Sometimes:0,0,1,0,0;Often:0,0,0,1,0;Always:0,0,0,0,0 | TZ15_Always_excited_1 | 44 |
|  |  | TZ15_Always_excited_3 | 45 |
|  |  | TZ15_Always_excited_4 | 46 |
| 24.Is your child easily restless? | Never:1,0,0,0,0;Rarely:0,0,0,0,0;Sometimes:0,0,1,0,0;Often:0,0,0,0,0;Always:0,0,0,0,0 | TZ16_Easily_restless_1 | 47 |
|  |  | TZ16_Easily_restless_3 | 48 |
| 25.Is your child’s abdomen bloated? | Never:1,0,0,0,0;Rarely:0,0,0,0,0;Sometimes:0,0,0,0,0;Often:0,0,0,0,0;Always:0,0,0,0,0 | TZ18_Abdominal_obesity_is_flabby_and_soft_1 | 49 |
| 26.Is your child always feeling sleepy? | Never:1,0,0,0,0;Rarely:0,1,0,0,0;Sometimes:0,0,0,0,0;Often:0,0,0,0,0;Always:0,0,0,0,0 | TZ19_Always_sleepy_1 | 50 |
|  |  | TZ19_Always_sleepy_2 | 51 |
| 27.Does your child like to eat sweet or greasy foods? | Never:1,0,0,0,0;Rarely:0,0,0,0,0;Sometimes:0,0,1,0,0;Often:0,0,0,1,0;Always:0,0,0,0,0 | TZ20_Like_to_eat_sweet_or_greasy_things_1 | 52 |
|  |  | TZ20_Like_to_eat_sweet_or_greasy_things_3 | 53 |
|  |  | TZ20_Like_to_eat_sweet_or_greasy_things_4 | 54 |
| 28.Does your child snore at night? | Never:1,0,0,0,0;Rarely:0,1,0,0,0;Sometimes:0,0,1,0,0;Often:0,0,0,0,0;Always:0,0,0,0,0 | TZ21_Snoring_at_night_1 | 55 |
|  |  | TZ21_Snoring_at_night_2 | 56 |
|  |  | TZ21_Snoring_at_night_3 | 57 |
| 29.Does your child have a thick coating on their tongue? | Never:1,0,0,0,0;Rarely:0,1,0,0,0;Sometimes:0,0,1,0,0;Often:0,0,0,0,0;Always:0,0,0,0,0 | TZ22_Thick_and_greasy_tongue_coating_1 | 58 |
|  |  | TZ22_Thick_and_greasy_tongue_coating_2 | 59 |
|  |  | TZ22_Thick_and_greasy_tongue_coating_3 | 60 |
| 30.Does your child often have red eyes or get eye discharge easily? | Never:1,0,0,0,0;Rarely:0,1,0,0,0;Sometimes:0,0,1,0,0;Often:0,0,0,0,0;Always:0,0,0,0,0 | TZ23_Always_red_eyes_or_easy_to_get_gum_1 | 61 |
|  |  | TZ23_Always_red_eyes_or_easy_to_get_gum_2 | 62 |
|  |  | TZ23_Always_red_eyes_or_easy_to_get_gum_3 | 63 |
| 31.Is your child prone to developing eczema? | Never:1,0,0,0,0;Rarely:0,1,0,0,0;Sometimes:0,0,0,0,0;Often:0,0,0,0,0;Always:0,0,0,0,0 | TZ24_Eczema_is_easy_to_grow_on_the_body_1 | 64 |
|  |  | TZ24_Eczema_is_easy_to_grow_on_the_body_2 | 65 |
| 32.Does your child like spicy or fried barbecue foods? | Never:1,0,0,0,0;Rarely:0,0,0,0,0;Sometimes:0,0,0,0,0;Often:0,0,0,0,0;Always:0,0,0,0,0 | TZ25_Like_to_eat_spicy_or_fried_barbecue_food_1 | 66 |
| 33.Is your child's sweat sticky? | Never:1,0,0,0,0;Rarely:0,1,0,0,0;Sometimes:0,0,1,0,0;Often:0,0,0,0,0;Always:0,0,0,0,0 | TZ26_Sweat_is_sticky_1 | 67 |
|  |  | TZ26_Sweat_is_sticky_2 | 68 |
|  |  | TZ26_Sweat_is_sticky_3 | 69 |
| 34.Does your child often develop bruises on the skin without knowing how they got them? | Never:1,0,0,0,0;Rarely:0,1,0,0,0;Sometimes:0,0,0,0,0;Often:0,0,0,0,0;Always:0,0,0,0,0 | TZ27_The_skin_always_appears_blue_and_purple_ecchymosis_unconsciously_1 | 70 |
|  |  | TZ27_The_skin_always_appears_blue_and_purple_ecchymosis_unconsciously_2 | 71 |
| 35.Does your child have any painful areas? | Never:1,0,0,0,0;Rarely:0,1,0,0,0;Sometimes:0,0,0,0,0;Often:0,0,0,0,0;Always:0,0,0,0,0 | TZ28_Where_there_is_pain_on_the_body_1 | 72 |
|  |  | TZ28_Where_there_is_pain_on_the_body_2 | 73 |
| 36.Is the skin on your child's body, or on their neck, armpits, or the crease of their thighs, darker than normal? | Never:1,0,0,0,0;Rarely:0,1,0,0,0;Sometimes:0,0,0,0,0;Often:0,0,0,0,0;Always:0,0,0,0,0 | TZ29_The_skin_on_the_body_or_the_skin_on_the_neck_armpit_or_thigh_root_is_dark_1 | 74 |
|  |  | TZ29_The_skin_on_the_body_or_the_skin_on_the_neck_armpit_or_thigh_root_is_dark_2 | 75 |
| 37.Does your child easily get dark circles under their eyes? | Never:1,0,0,0,0;Rarely:0,1,0,0,0;Sometimes:0,0,1,0,0;Often:0,0,0,0,0;Always:0,0,0,0,0 | TZ30_Be_prone_to_dark_circles_1 | 76 |
|  |  | TZ30_Be_prone_to_dark_circles_2 | 77 |
|  |  | TZ30_Be_prone_to_dark_circles_3 | 78 |
| 38.Are your child's gums bluish or darker than normal? | Never:1,0,0,0,0;Rarely:0,1,0,0,0;Sometimes:0,0,0,0,0;Often:0,0,0,0,0;Always:0,0,0,0,0 | TZ31_Blue_or_dark_gums_1 | 79 |
|  |  | TZ31_Blue_or_dark_gums_2 | 80 |
| 39.Does your child have rough, fish scale-like skin? | Never:1,0,0,0,0;Rarely:0,0,0,0,0;Sometimes:0,0,0,0,0;Often:0,0,0,0,0;Always:0,0,0,0,0 | TZ32_Rough_skin_like_fish_scales_1 | 81 |
| 40.Are your child's lips dark or bluish? | Never:1,0,0,0,0;Rarely:0,1,0,0,0;Sometimes:0,0,0,0,0;Often:0,0,0,0,0;Always:0,0,0,0,0 | TZ33_Lips_are_dark_or_blue_1 | 82 |
|  |  | TZ33_Lips_are_dark_or_blue_2 | 83 |
| 41.Is your child always feeling down? | Never:1,0,0,0,0;Rarely:0,1,0,0,0;Sometimes:0,0,0,0,0;Often:0,0,0,0,0;Always:0,0,0,0,0 | TZ34_Always_moping_1 | 84 |
|  |  | TZ34_Always_moping_2 | 85 |
| 42.Is your child easily anxious or restless? | Never:1,0,0,0,0;Rarely:0,1,0,0,0;Sometimes:0,0,0,0,0;Often:0,0,0,0,0;Always:0,0,0,0,0 | TZ35_Easily_nervous_1 | 86 |
|  |  | TZ35_Easily_nervous_2 | 87 |
| 43.Does your child tend to lack self-confidence? | Never:1,0,0,0,0;Rarely:0,1,0,0,0;Sometimes:0,0,1,0,0;Often:0,0,0,0,0;Always:0,0,0,0,0 | TZ36_Easy_to_lack_self_confidence_1 | 88 |
|  |  | TZ36_Easy_to_lack_self_confidence_2 | 89 |
|  |  | TZ36_Easy_to_lack_self_confidence_3 | 90 |
| 44.Is your child easily scared or startled? | Never:1,0,0,0,0;Rarely:0,1,0,0,0;Sometimes:0,0,1,0,0;Often:0,0,0,0,0;Always:0,0,0,0,0 | TZ37_Easily_afraid_or_frightened_1 | 91 |
|  |  | TZ37_Easily_afraid_or_frightened_2 | 92 |
|  |  | TZ37_Easily_afraid_or_frightened_3 | 93 |
| 45.Does your child always cry when facing difficulties? | Never:1,0,0,0,0;Rarely:0,0,0,0,0;Sometimes:0,0,1,0,0;Often:0,0,1,0,0;Always:0,0,0,0,0 | TZ38_Always_crying_when_in_trouble_1 | 94 |
|  |  | TZ38_Always_crying_when_in_trouble_3 | 95 |
|  |  | TZ38_Always_crying_when_in_trouble_4 | 96 |
| 46.Is your child shy or timid? | Never:1,0,0,0,0;Rarely:0,0,0,0,0;Sometimes:0,0,1,0,0;Often:0,0,0,0,0;Always:0,0,0,0,0 | TZ39_Easily_shy_or_timid_1 | 97 |
|  |  | TZ39_Easily_shy_or_timid_3 | 98 |
| 47.Is your child always worried about a lot of things? | Never:1,0,0,0,0;Rarely:0,1,0,0,0;Sometimes:0,0,1,0,0;Often:0,0,0,0,0;Always:0,0,0,0,0 | TZ40_Always_worried_about_a_lot_of_things_1 | 99 |
|  |  | TZ40_Always_worried_about_a_lot_of_things_2 | 100 |
|  |  | TZ40_Always_worried_about_a_lot_of_things_3 | 101 |
| 48.Is your child more attached to you now than before? | Never:1,0,0,0,0;Rarely:0,0,0,0,0;Sometimes:0,0,1,0,0;Often:0,0,0,0,0;Always:0,0,0,0,0 | TZ41_More_attached_to_you _now_than_before_1 | 102 |
|  |  | TZ41_More_attached_to_you _now_than_before_3 | 103 |
| 49.Does your child sneeze even when they don't have a cold? | Never:1,0,0,0,0;Rarely:0,1,0,0,0;Sometimes:0,0,0,0,0;Often:0,0,0,0,0;Always:0,0,0,0,0 | TZ42_Sneez_when_do_not_have_a_cold_1 | 104 |
|  |  | TZ42_Sneez_when_do_not_have_a_cold_2 | 105 |
| 50.Does your child have a stuffy nose or runny nose even when they don't have a cold? | Never:1,0,0,0,0;Rarely:0,1,0,0,0;Sometimes:0,0,0,0,0;Often:0,0,0,0,0;Always:0,0,0,0,0 | TZ43_Without_cold_but_have_stuffy_nose_and_runny_nose_1 | 106 |
|  |  | TZ43_Without_cold_but_have_stuffy_nose_and_runny_nose_2 | 107 |
| 51.Does your child cough or wheeze during seasonal changes, temperature changes, or when smelling unusual odors? | Never:1,0,0,0,0;Rarely:0,1,0,0,0;Sometimes:0,0,0,0,0;Often:0,0,0,0,0;Always:0,0,0,0,0 | TZ44_Cough_and_wheeze_when_the_season_changes_the_temperature_changes_or_smells_strange_smell_1 | 108 |
|  |  | TZ44_Cough_and_wheeze_when_the_season_changes_the_temperature_changes_or_smells_strange_smell_2 | 109 |
| 52.Is your child prone to allergies (to medications, foods, scents, pollen, or during seasonal changes and climate shifts)? | Never:1,0,0,0,0;Rarely:0,1,0,0,0;Sometimes:0,0,0,0,0;Often:0,0,0,0,0;Always:0,0,0,0,0 | TZ45_Susceptible_to_allergies_1 | 110 |
|  |  | TZ45_Susceptible_to_allergies_2 | 111 |
| 53.Does your child’s skin get itchy or develop hives easily? | Never:1,0,0,0,0;Rarely:0,1,0,0,0;Sometimes:0,0,0,0,0;Often:0,0,0,0,0;Always:0,0,0,0,0 | TZ46_The_skin_is_prone_to_itching_and_urticaria_1 | 112 |
|  |  | TZ46_The_skin_is_prone_to_itching_and_urticaria_2 | 113 |
| 54.Has your child's skin ever developed purpura (purplish-red spots or bruises) due to allergies? | Never:1,0,0,0,0;Rarely:0,0,0,0,0;Sometimes:0,0,0,0,0;Often:0,0,0,0,0;Always:0,0,0,0,0 | TZ47_Purpura_has_occurred_in_the_skin_due_to_allergies_1 | 114 |
| 55.Does your child's skin turn red and show scratch marks when scratched? | Never:1,0,0,0,0;Rarely:0,1,0,0,0;Sometimes:0,0,0,0,0;Often:0,0,0,0,0;Always:0,0,0,0,0 | TZ48_The_skin_turns_red_when_scratched_and_scratches_appear_1 | 115 |
|  |  | TZ48_The_skin_turns_red_when_scratched_and_scratches_appear_2 | 116 |
| 56.Is your child always lively and strong? | Never:1,0,0,0,0;Rarely:0,0,0,0,0;Sometimes:0,0,0,0,0;Often:0,0,0,0,0;Always:0,0,0,0,0 | TZ49_Always_lively_and_strong_1 | 117 |
| 57.Can your child adapt to changes in the weather and new environments (such as kindergarten)? | Never:1,0,0,0,0;Rarely:0,0,0,0,0;Sometimes:0,0,0,0,0;Often:0,0,0,0,0;Always:0,0,0,0,0 | TZ50_Can_adapt_to_weather_changes_and_new_environment_1 | 118 |
| 58.Does your child often have nightmares or wake up frightened from dreams? | Never:1,0,0,0,0;Rarely:0,1,0,0,0;Sometimes:0,0,1,0,0;Often:0,0,0,0,0;Always:0,0,0,0,0 | TZ51_Easy_to_have_nightmares_or_wake_up_from_dreams_1 | 119 |
|  |  | TZ51_Easy_to_have_nightmares_or_wake_up_from_dreams_2 | 120 |
|  |  | TZ51_Easy_to_have_nightmares_or_wake_up_from_dreams_3 | 121 |
| 59.Does your child eat less compared to other children of the same age? | Never:1,0,0,0;Sometimes:0,0,0,0;Often:0,0,1,0;Always:0,0,0,1 | YSRT1_Eat_less_than_your_peers_1 | 122 |
|  |  | YSRT1_Eat_less_than_your_peers_3 | 123 |
|  |  | YSRT1_Eat_less_than_your_peers_4 | 124 |
| 60.Does your child eat more slowly compared to peers? | Never:1,0,0,0;Sometimes:0,0,0,0;Often:0,0,1,0;Always:0,0,0,1 | YSRT2_Eating_slowly_compared_with_peers_1 | 125 |
|  |  | YSRT2_Eating_slowly_compared_with_peers_3 | 126 |
|  |  | YSRT2_Eating_slowly_compared_with_peers_4 | 127 |
| 61.Is your child not interested in food? | Never:1,0,0,0;Sometimes:0,1,0,0;Often:0,0,1,0;Always:0,0,0,0 | YSRT3_Not_interested_in_food_1 | 128 |
|  |  | YSRT3_Not_interested_in_food_2 | 129 |
|  |  | YSRT3_Not_interested_in_food_3 | 130 |
| 62.Has your child refused certain foods for more than a month? | Never:1,0,0,0;Sometimes:0,0,0,0;Often:0,0,1,0;Always:0,0,0,1 | YSRT4_Refuse_certain_foods_for_more_than_a_month_1 | 131 |
|  |  | YSRT4_Refuse_certain_foods_for_more_than_a_month_3 | 132 |
|  |  | YSRT4_Refuse_certain_foods_for_more_than_a_month_4 | 133 |
| 63.Is your child unwilling to try new foods? | Never:1,0,0,0;Sometimes:0,0,0,0;Often:0,0,1,0;Always:0,0,0,0 | YSRT5_Unwilling_to_try_new_food_1 | 134 |
|  |  | YSRT5_Unwilling_to_try_new_food_3 | 135 |
| 64.Strong preference for certain textures or types of food | Never:1,0,0,0;Sometimes:0,0,0,0;Often:0,0,1,0;Always:0,0,0,1 | YSRT6_Have_a_strong_preference_for_certain_textures_or_certain_types_of_food_1 | 136 |
|  |  | YSRT6_Have_a_strong_preference_for_certain_textures_or_certain_types_of_food_3 | 137 |
|  |  | YSRT6_Have_a_strong_preference_for_certain_textures_or_certain_types_of_food_4 | 138 |
| 65.Is your child distracted during meals (watching TV, playing video games, etc.)? | Never:1,0,0,0;Sometimes:0,0,0,0;Often:0,0,1,0;Always:0,0,0,1 | YSRT7_Distraction_from_eating_1 | 139 |
|  |  | YSRT7_Distraction_from_eating_3 | 140 |
|  |  | YSRT7_Distraction_from_eating_4 | 141 |
| 66.Does your child have an irregular eating schedule? | Never:1,0,0,0;Sometimes:0,0,0,0;Often:0,0,0,0;Always:0,0,0,0 | YSRT8_The_dining_place_is_not_fixed_1 | 142 |
| 67.Do you have little emotional exchange when eating with your child? | Never:1,0,0,0;Sometimes:0,1,0,0;Often:0,0,0,0;Always:0,0,0,0 | YSFY1_Few_emotional_exchanges_during_meals_1 | 143 |
|  |  | YSFY1_Few_emotional_exchanges_during_meals_2 | 144 |
| 68.Do you force or punish your child to eat more during meals? | Never:1,0,0,0;Sometimes:0,1,0,0;Often:0,0,1,0;Always:0,0,0,0 | YSFY2_Force_or_punish_children_to_eat_more_1 | 145 |
|  |  | YSFY2_Force_or_punish_children_to_eat_more_2 | 146 |
|  |  | YSFY2_Force_or_punish_children_to_eat_more_3 | 147 |
| 69.Do you use inducements to get your child to eat during meals (toys, TV, stories, rewards, etc.)? | Never:1,0,0,0;Sometimes:0,1,0,0;Often:0,0,1,0;Always:0,0,0,0 | YSFY3_Induce_children_to_eat_1 | 148 |
|  |  | YSFY3_Induce_children_to_eat_2 | 149 |
|  |  | YSFY3_Induce_children_to_eat_3 | 150 |
| 70.Do you allow children to choose their own food when eating with them? | Never:1,0,0,0;Sometimes:0,0,0,0;Often:0,0,0,0;Always:0,0,0,0 | YSFY4_Allow_children_to_choose_food_at_will_1 | 151 |
| 71.Do you rarely encourage your child to try new foods when eating together? | Never:1,0,0,0;Sometimes:0,0,0,0;Often:0,0,0,0;Always:0,0,0,0 | YSFY5_Children_are_rarely_encouraged_to_taste_new_foods_1 | 152 |
| 72.Do you allow children to eat snacks whenever they want? | Never:1,0,0,0;Sometimes:0,0,0,0;Often:0,0,0,0;Always:0,0,0,0 | YSFY6_Allow_children_to_eat_snacks_if_they_want_1 | 153 |
| 73.Do you allow children to roam around while eating? | Never:1,0,0,0;Sometimes:0,1,0,0;Often:0,0,0,0;Always:0,0,0,0 | YSFY7_Allow_children_to_dine_and_wander_around_1 | 154 |
|  |  | YSFY7_Allow_children_to_dine_and_wander_around_2 | 155 |
| 74.What is your child's average mealtime? | <25min:0,0,0;25min-45min:0,1,0;>45min:0,0,0 | YS1_Your_child_average_meal_time_1 | 156 |
| 75.Do you think your child has a picky eater problem? | Yes:1,0,0;No:0,1,0;Unclear:0,0,0 | YS2_Do_you_think_your_child_has_a_picky_eater_problem_1 | 157 |
|  |  | YS2_Do_you_think_your_child_has_a_picky_eater_problem_2 | 158 |

**Table S9. Results of MICE Imputation and Sensitivity Analysis**

| Imputed Dataset | AUC | Sensitivity | Specificity | Brier |
| --- | --- | --- | --- | --- |
| Dataset 1 | 0.7298 | 0.6256 | 0.7120 | 0.2108 |
| Dataset 2 | 0.7303 | 0.6274 | 0.7075 | 0.2105 |
| Dataset 3 | 0.7302 | 0.6274 | 0.7098 | 0.2105 |
| Dataset 4 | 0.7318 | 0.6244 | 0.7086 | 0.2101 |
| Dataset 5 | 0.7293 | 0.6277 | 0.7096 | 0.2108 |
| Mean ± standard deviation | 0.7303  ± 0.0009 | 0.6265  ± 0.0014 | 0.7095  ± 0.0017 | 0.2105  ±0.0003 |
