# Supplementary material for: Development and Validation of an Explainable Machine Learning Model to Assess the Prevalence Probability of Gastrointestinal Heat Retention Syndrome in Children: Cross-Sectional Study
Source: J Med Internet Res. 2026 Jul 2;28:e94775. doi: 10.2196/94775 (PMC13376857; doi:10.2196/94775)
Supplement: Multimedia Appendix 2 [file jmir_v28i1e94775_app2.docx]

| **Section/Topic** | **Item Content** | **Relevant Content Included** |
| --- | --- | --- |
| Title | 1 Explicitly state whether the study constitutes development or validation of a multivariable prediction model, the target population, and the outcome to be predicted | Yes |
| Background | 2 Briefly describe the clinical context and rationale for developing or validating the model(s) | Yes |
| Objectives | 3 State the research objectives, specifying whether the study involves model development, validation, or both | Yes |
| Methods | 4 Description of data sources | Yes |
|  | 5 Description of inclusion and exclusion criteria and data collection settings | Yes |
|  | 6 Specification of model-predicted outcomes; for prognostic models, the timeframe for prediction must be specified | Yes |
|  | 7 Specification of model type, outlining of modeling procedures and internal validation methods | Yes |
|  | 8 Specification of measures for evaluating model performance (e.g., discrimination, calibration, clinical utility) | Yes |
| Results | 9 Reporting of participant numbers and outcome events | Yes |
|  | 10 Overview of predictor variables in the final model | Yes |
|  | 11 Reporting of model performance evaluation results (including confidence intervals) | Yes |
| Discussion | 12 Overall interpretation of primary findings | Yes |
| Study Registration | 13 Listing of study registration number and registry name | No |
