# Supplementary material for: Development and Validation of an Explainable Machine Learning Model to Assess the Prevalence Probability of Gastrointestinal Heat Retention Syndrome in Children: Cross-Sectional Study
Source: J Med Internet Res. 2026 Jul 2;28:e94775. doi: 10.2196/94775 (PMC13376857; doi:10.2196/94775)
Supplement: Multimedia Appendix 3 [file jmir_v28i1e94775_app3.docx]

| Section/Topic | Item Number | Development/Evaluation | Item Content | Presence of Relevant Content and Explanation |
| --- | --- | --- | --- | --- |
| Title | 1 | D,E | Specify whether the study aims to develop a multivariable prediction model or evaluate its performance, define the target population, and identify the predicted outcome. | Yes |
| Abstract | 2 | D,E | Refer to the TRIPOD+AI checklist for abstracts | Yes |
| Introduction |  |  |  |  |
| Background | 3a | D,E | Describe the background in the relevant medical field (including whether it pertains to diagnostic or prognostic models) and the rationale for developing or evaluating the prediction model, with citations of existing models. | Yes |
|  | 3b | D,E | Describe the target population and the intended use of the prediction model in the healthcare setting, including its intended users (e.g., healthcare professionals, patients, general public). | Yes |
|  | 3c | D,E | Describing known health inequalities among different sociodemographic groups | Yes |
| Objective | 4 | D,E | Clearly state the research objectives, including whether the study focuses on predictive model development, validation, or both | Yes |
| Methods |  |  |  | Yes |
| Data | 5a | D,E | Describe the data sources for development and evaluation datasets (e.g., randomized trials, cohort studies, routine healthcare data, or registry data), providing the rationale for their selection and data representativeness | Yes |
|  | 5b | D,E | Specify participant data collection dates, including recruitment start and end dates; where applicable, indicate the follow-up end date | Yes |
|  | 6a | D,E | Describe key elements of the study setting (e.g., primary care, secondary care, general population), including the number and location of participating centers | Yes |
| Participants | 6b | D,E | Describe the inclusion and exclusion criteria for study participants | Yes |
|  | 6c | D,E | Specify any treatments received by participants and how these were managed during model development or evaluation (if applicable) | No ( This cross-sectional study assessed current disease probability without involving pharmaceutical/clinical treatments; therefore, treatment factors required no management ) |
| Data preprocessing | 7 | D,E | Describe all data preprocessing and quality checks performed, including the consistency of these procedures across relevant sociodemographic groups | Yes |
| Outcome | 8a | D,E | Clearly define the predicted outcome and its temporal scope, including assessment methods and time points, rationale for outcome selection, and consistency of outcome assessment approaches across sociodemographic groups | Yes |
|  | 8b | D | If outcome assessment requires subjective interpretation, describe the evaluators' qualifications and demographic characteristics | Yes |
|  | 8c | D | Report all blinding procedures implemented for outcome assessment pertaining to the predicted outcomes | Yes |
| Predictor variables | 9a | D | Describe the basis for selecting initial predictor variables (e.g., based on literature, existing models, all available predictors) and any prescreening process of predictors conducted prior to modeling | Yes |
|  | 9b | D,E | Clearly define all predictor variables, including measurement methods and timing (along with blinding procedures applied during assessment of both outcome predictors and other predictors) | Yes |
|  | 9c | D | If predictor variable measurement requires subjective interpretation, describe the evaluators' qualifications and demographic characteristics | Yes |
| Sample Size | 10 | D,E | Describe the method for determining the required sample size (specified separately for model development and evaluation), and justify that the sample size is sufficient to address the research question. This section should include all details relevant to sample size calculation. | Yes |
| Missing Data | 11 | D,E | Describe the approach for handling missing data. Provide the rationale for deletion of any data. | Yes |
| Analytical Methods | 12a | D | Describe how data were utilized in the analyses (e.g., for model development versus evaluation), including whether data partitioning was implemented (based on sample size requirements). | Yes |
|  | 12b | D | Depending on the model type, describe how predictor variables were processed in the analyses (e.g., functional form, rescaling, transformation, or standardization). | Yes |
|  | 12c | D | Specify the model type, design rationale, all modeling steps (including any hyperparameter tuning), and internal validation methods. | Yes |
|  | 12d | D,E | Describe whether and how heterogeneity in model parameter estimates and performance across different subsets (e.g., hospitals, countries) was addressed and quantified. Refer to the TRIPOD-Cluster guidelines‡ for additional considerations. | Yes |
|  | 12e | D,E | Specify all metrics and plots—along with their rationale—used to evaluate model performance (e.g., discrimination, calibration, clinical utility). If multiple models were compared, describe the methods employed for model comparison. | Yes |
|  | 12f | E | Describe any model updates arising from the evaluation process (e.g., recalibration), including overall updates or those specific to particular sociodemographic groups/settings. | No ( This study involved initial model development and internal validation; no recalibration—overall or for sociodemographic subgroups—was conducted during test set validation ) |
|  | 12g | E | For model evaluation, describe how the predicted values are calculated (e.g., formula, code, object, API, or model). | Yes |
| Class imbalance | 13 | D,E | If methods for handling class imbalance were employed, specify the rationale and specific approaches, along with any subsequent methods used for recalibrating the model or its predictions. | Yes |
| Fairness | 14 | D,E | Describe any methods used to address model fairness concerns and their theoretical basis. | No (this study treated sociodemographic variables as covariates; all sociodemographic subgroups received no differential treatment; furthermore, no specialized algorithmic optimizations for model fairness were conducted). |
| Model output | 15 | D | Specify the output format of the predictive model (e.g., probability values, classification results). Provide detailed information on categorization criteria and the methodology for determining classification thresholds | Yes |
| Training and Evaluation | 16 | D,E | Indicate any differences in clinical setting, eligibility criteria, outcomes, and predictors between data used for model development versus evaluation | Yes |
| Ethical Approval | 17 | D,E | Specify the name of the Institutional Review Board or Ethics Committee that approved the study, and state whether participant informed consent was obtained or waived | Yes |
| Open Science |  |  |  |  |
| Funding | 18a | D,E | Report the study's funding sources and the funders' involvement in the study | Yes |
| Conflicts of Interest | 18b | D,E | Declare all authors' conflicts of interest and financial disclosures | Yes |
| Study Protocol | 18c | D,E | Indicate whether the study protocol is accessible and the means of access, or state that no protocol was prepared | Yes |
| Study Registration | 18d | D,E | Provide study registration information (including registry name and registration number), or declare that the study was not registered | No |
| Data Sharing | 18e | D,E | Provide detailed information on access to the study data | Yes |
| Code Sharing | 18f | D,E | Provide detailed information on access to the analysis code § | Yes |
| Patient and Public Involvement | 19 | D,E | Provide details of any patient and public involvement in study design, conduct, result reporting, interpretation, or dissemination, or declare no patient and public involvement | Yes |
| Results |  |  |  |  |
| Participants | 20a | D,E | Describe participant status at each study stage, including numbers of participants with and without outcomes, and (if applicable) a summary of follow-up duration | Yes |
|  | 20b | D,E | Report overall data characteristics, and separately report characteristics for each dataset or environment, including key health issues, key predictor variables (including demographics), treatments received, sample size, number of outcome events, follow-up duration, and volume of missing data. Tables may be helpful. Report any differences in key demographic characteristics. | Yes |
|  | 20c | E | For model evaluation, compare the distributions of important predictor variables (demographics, predictors, and outcome variables) in the validation dataset side-by-side with those in the development dataset. | No (this study constitutes internal validation, with consistent data distribution between the test and training sets) |
| Model development | 21 | D | Specify the number of participants and outcome events in each analysis (e.g., during model development, tuning, or evaluation phases) | Yes |
| Model specifications | 22 | D | Provide comprehensive details of the complete prediction model (e.g., formulas, code, objects, API) to enable predictions for new individuals and support third-party evaluation and implementation, including any accessibility or reuse restrictions (e.g., free access, proprietary models) | Yes |
| Model performance | 23a | D,E | Report model performance estimates with confidence intervals, including subgroup results (e.g., for different socio-demographic groups). Consider using charts to facilitate results presentation | Yes |
|  | 23b | D,E | If relevant analyses were performed, report model performance correlation results between clusters. Refer to the TRIPOD-Cluster guidelines | No (This study was conducted exclusively in Longgang District, Shenzhen; inter-cluster correlation analyses were not performed) |
| Model updating | 24 | E | Report all model updating results, including updated models and their subsequent performance | No (This study represents initial model development; no model updates were implemented) |
| Discussion |  |  |  |  |
| Interpretation of results | 25 | D,E | Interpret primary findings comprehensively in accordance with research objectives and existing literature, including discussion of fairness considerations | Yes |
| Limitations | 26 | D,E | Discuss study limitations (e.g., sample representativeness, sample size, overfitting, missing data) and their impact on bias, statistical uncertainty, and generalizability | Yes |
| Utility of the model within the current medical setting | 27a | D | Describe how poor-quality or unavailable input data (e.g., predictor variable values) were evaluated and handled during predictive model implementation | Yes |
|  | 27b | D | Specify whether user interaction is required during input data processing or model application; and state any professional qualifications expected of users | Yes |
|  | 27c | D,E | Discuss any next steps for future research, with particular attention to the model's applicability and generalizability | Yes |
