# Supplementary figures and images for: Development and Validation of an Explainable Machine Learning Model to Assess the Prevalence Probability of Gastrointestinal Heat Retention Syndrome in Children: Cross-Sectional Study
Source: J Med Internet Res. 2026 Jul 2;28:e94775. doi: 10.2196/94775 (PMC13376857; doi:10.2196/94775)

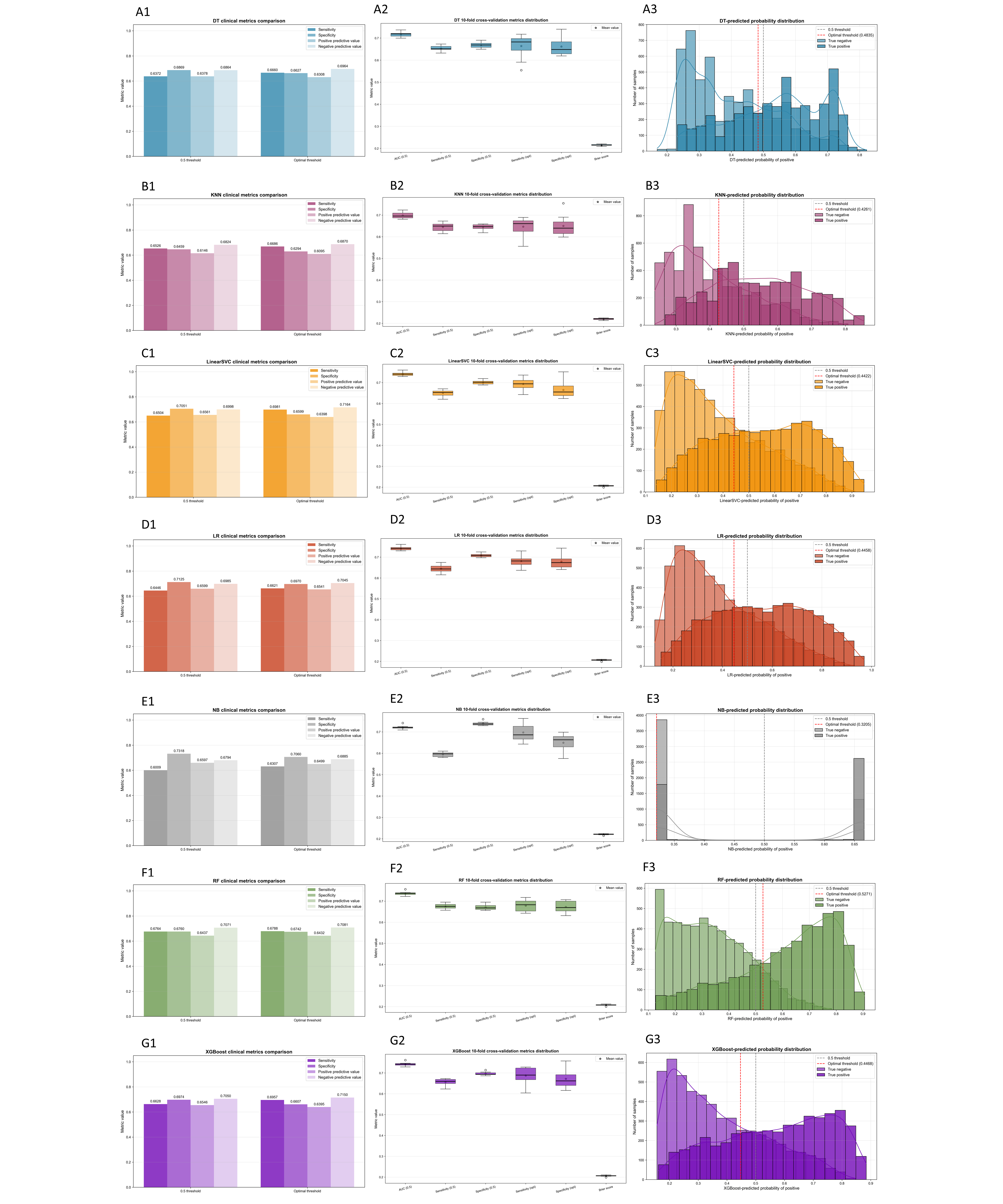

Supplement: Multimedia Appendix 4 [file jmir_v28i1e94775_app4.png]
